# Photoinduced Multicomponent Difluoromethylation of Imines via Iron-Mediated Ligand-to-Metal Charge Transfer

**DOI:** 10.1021/acs.orglett.6c01025

**Published:** 2026-03-26

**Authors:** Hyungwoo Choi, Jinwoo Lee, Seok Beom Lee, Sangmyung Han, Joonseok Jang, Suckchang Hong

**Affiliations:** † Research Institute of Pharmaceutical Sciences, College of Pharmacy, 26725 Seoul National University, Seoul 08826, Republic of Korea; ‡ Natural Products Research Institute, College of Pharmacy, Seoul National University, Seoul 08826, Republic of Korea

## Abstract

α-CF_2_H substituted amines exhibit improved physicochemical properties and biological performance in pharmaceutically relevant molecules; however, to date, radical difluoromethylation has required expensive preactivated CF_2_H reagents and photocatalysts. Herein, we report an iron-mediated ligand-to-metal charge-transfer (LMCT) strategy that enables the direct difluoromethylation of imines generated in situ. Using earth-abundant iron catalysis and inexpensive difluoroacetate salts as a cost-effective CF_2_H source, this method affords pharmaceutically relevant α-CF_2_H amine structures under mild conditions with decagram-scale scalability.

## Graphical abstract

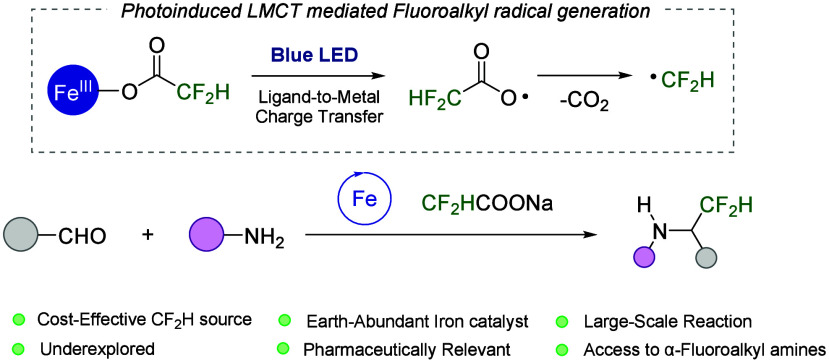

As a hydrogen bond donor, the difluoromethyl (CF_2_H) group is an important bioisostere of alcohols, thiols, and amines. The unique electronic characteristics of the CF_2_H group also improve molecular properties such as lipophilicity, membrane permeability, metabolic stability, and p*K*_a_ values.^1–7^ Notably, α-CF_2_H substituted amines, which are found in various pharmaceutical agents, including eflornithine and inavolisib, combine the pharmacologically advantageous properties of both amine moieties and difluoromethyl substituents (Figure 1A).^8–10^ Because this structure can be efficiently accessed through difluoromethylation of imines, developing reliable difluoromethylation methods for imines is essential for drug discovery.

**Figure 1:**
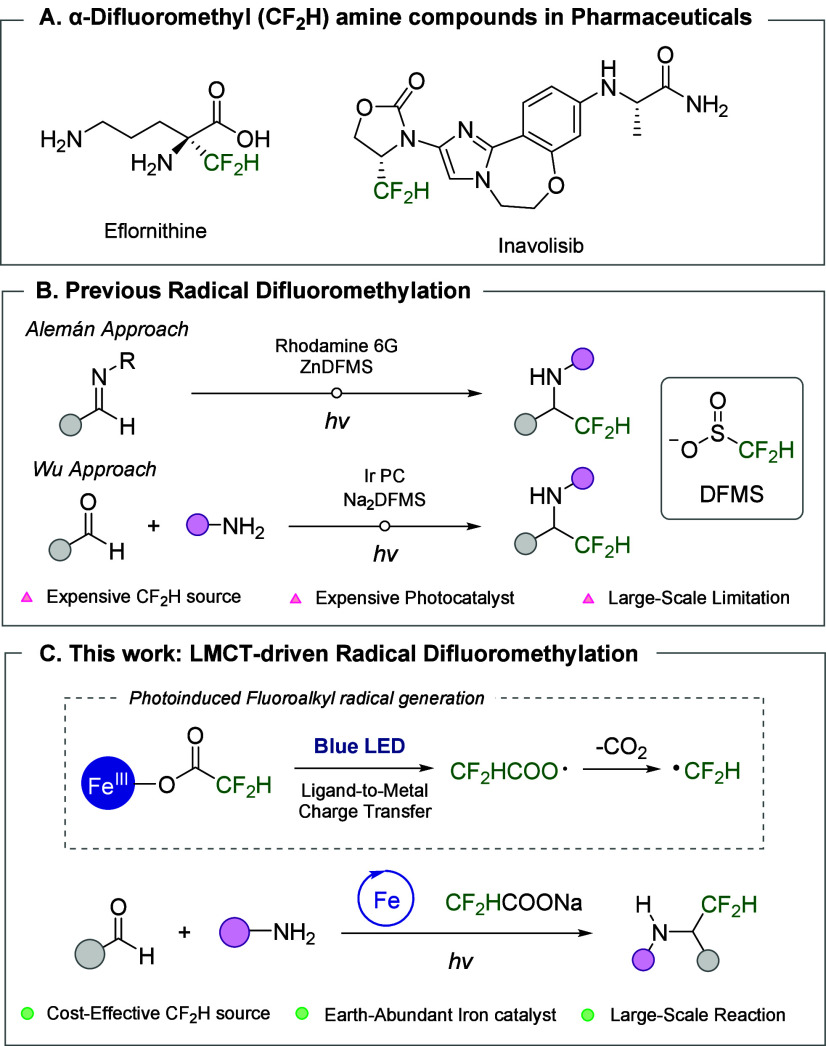
Development of direct difluoromethylation of imines.

Initial approaches to direct difluoromethylation relied on strongly nucleophilic difluoromethyl precursors that underwent nucleophilic addition to preformed imines.^11,12^ These two-electron pathways, however, rely on harsh basic conditions or multistep reduction, resulting in limited functional group compatibility. To address these limitations, Alemán et al. reported mild radical difluoromethylation of imines using a photocatalyst and single-electron-transfer (SET) activation of the CF_2_H source DFMS (zinc difluorosulfinate) (Figure 1B).^13^ Wu et al. developed a multicomponent difluoromethylation reaction in which an imine is formed in situ.^14^ Despite these advancements, both approaches still rely on relatively expensive difluoromethanesulfinate salts as the CF_2_H source and expensive photocatalysts, limiting large-scale synthesis and pharmaceutical applications.

To overcome these limitations, we employed difluoroacetate as a CF_2_H radical precursor within an iron-mediated ligand-to-metal charge transfer (LMCT) decarboxylative difluoromethylation strategy (Figure 1C). Although difluoroacetic acid is among the most commercially accessible difluoroalkyl sources, fluoroalkyl carboxylic acids have exceptionally high oxidation potentials, rendering them difficult to activate via conventional SET using photocatalysts.^15–17^ In contrast, LMCT proceeds through an inner-sphere electron-transfer mechanism that does not require redox potential matching or long-lived excited states.^18–21^ Fe­(III)-carboxylate complexes have been extensively investigated for LMCT-mediated activation of (fluoro)­alkyl carboxylates, establishing this platform as a powerful tool for radical functionalization.^19–27^ Moreover, compared with precious heavy metals typically used in photocatalysts, iron is earth-abundant, inexpensive, and has more favorable environmental and biological safety profiles.^26,27^

We report a multicomponent radical difluoromethylation of imines using difluoroacetate as the CF_2_H source. By generating CF_2_H radicals via iron-mediated LMCT and incorporating them into imines formed in situ from aldehydes and amines, this method provides a cost-effective, sustainable, and pharmaceutically relevant approach for synthesizing α-CF_2_H amines.

We explored difluoromethylation conditions using p-tolualdehyde and aniline as model substrates with sodium difluoroacetate and iron­(III) nitrate nonahydrate under 390 nm LEDs. In an iron-mediated LMCT process, the iron center is reduced from Fe­(III) to Fe­(II), concomitantly generating a CF_2_H radical from sodium difluoroacetate (top scheme of Table 1). The CF_2_H radical subsequently undergoes radical addition to the imine, generating an N-centered radical intermediate. We anticipated that this N-centered radical species would exhibit a high reduction potential and would therefore be capable of oxidizing Fe­(II) back to Fe­(III) while itself being reduced to an N-amide anion, thus establishing the iron catalytic cycle. We chose TMSOTf as an additive for two reasons: (i) to serve as a water scavenger that promotes imine formation and (ii) to generate acidic conditions that facilitate rapid protonation of the N-amide anion to the desired product while suppressing coordination of the Lewis-basic aniline to the iron catalyst. Unexpectedly, early screening revealed that stoichiometric iron was required, indicating inefficient catalytic turnover. To probe whether the catalytic cycle could still operate at lower iron loading, we reduced the amount of iron and found that full conversion was achieved even with 50 mol % Fe­(NO_3_)_3_·9H_2_O. This result supports the feasibility of catalytic turnover, while indicating the need for a plausible explanation for the inefficient turnover observed at lower iron catalyst loadings. We attributed this to insufficient concentrations of the N-centered radical at the initial stage of the reaction, which likely limited reoxidation of Fe­(II) to Fe­(III). To restore catalytic turnover, we introduced K_2_S_2_O_8_ as an oxidant additive and found that catalytic amounts of K_2_S_2_O_8_ enabled efficient turnover of the iron catalyst.

**Table 1:**
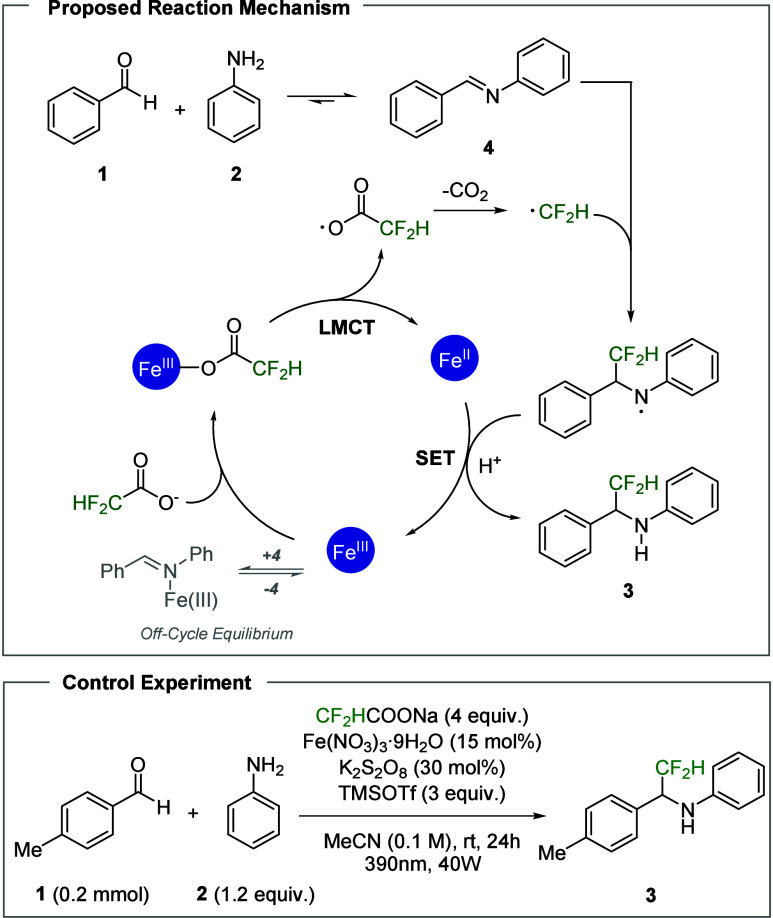
Proposed Mechanism and Control Reactions^a^

Entry	Deviation from standard conditions	Yield (%)^b^
1	None	99 (84)
2	FeCl_3_·6H_2_O, Fe(OTf)_3_, Fe(acac)_3_	66, 60, 12
3	Na_2_S_2_O_8_, Oxone, Bz_2_O_2_, H_2_O_2_	54, 35, 44, ND
4	TMSOTf 2 equiv, 1 equiv, no TMSOTf	64, 37, 18
5	DCM, DMSO, EA, THF	ND, 45, 62, ND
6	0.2 M, 0.05 M	40, 78
7	427 nm, 456 nm	46, 19
8	30 W, 20 W, No light	40, 22, ND
9	No iron salt	ND
10	Under Air	ND

a The reaction was performed on a 0.2 mmol scale.

b Yields were determined by^1^H NMR, and the yield in parentheses corresponds to the isolated yield.

The optimal conditions were identified through systematic screening. Under a standard set of conditions—Fe­(NO_3_)_3_·9H_2_O (15 mol %), CF_2_HCOONa (4.0 equiv), TMSOTf (3.0 equiv), and K_2_S_2_O_8_ (30 mol %) in MeCN (0.1 M) under 390 nm light irradiation and an Ar atmosphere—the desired product was obtained in an isolated yield of 84% (Table 1, entry 1). Control experiments were conducted to assess the effect of each reaction parameter on the yield. Among the iron salts examined, Fe­(NO_3_)_3_·9H_2_O was the most effective catalyst (Table 1, entry 2), and K_2_S_2_O_8_ was identified as the optimal catalytic oxidant additive (Table 1, entry 3). Reducing the amount of TMSOTf substantially decreased the yield (Table 1, entry 4), highlighting the crucial role of sufficiently acidic conditions. With respect to the solvent effect, the reaction proceeded only in coordinating solvents such as DMSO and ethyl acetate, consistent with previous reports on LMCT processes (Table 1, entry 5).^22,28^ Reaction concentration significantly affected the yield, which decreased considerably as the reaction mixture became more concentrated (Table 1, entry 6). The efficiency of the LMCT process also decreased substantially when irradiation at wavelengths above 400 nm was employed, resulting in significantly lower product yields (Table 1, entry 7). Moreover, no reaction occurred in the absence of the external light source or the iron salt, nor did the reaction proceed in air (Table 1, entries 8–10).

On the basis of the optimized conditions, we explored the substrate scope and synthesized a range of α-CF_2_H amines (Scheme 1). With respect to the aldehyde series, unsubstituted benzaldehyde (**3aa**) yielded the desired product in very high yield. Similarly, methyl-substituted substrates (**3ba**–**3da**) resulted in high yields at all three positions (ortho, meta, and para), and the para-*tert*-butyl-substituted aldehyde (**3ea**) was also well tolerated. In contrast, mesitaldehyde (**3fa**) markedly reduced the yield because of steric hindrance, which impairs imine formation. For a representative strongly electron-donating substituent, a para-methoxy group, no isolable product was obtained under the standard conditions (see SI, Section 3.D). However, the methoxy substituent itself was tolerated at the meta position. 3-Methoxybenzaldehyde (**3ga**) gave the desired product in a moderate yield. 4-Acetoxybenzaldehyde (**3ha**), bearing a moderately electron-donating substituent, afforded the desired product in moderate yield. These results suggest that the present reaction system is compatible with moderately electron-donating substituents, but not with strongly electron-donating ones. Halogen substituents (**3ia**–**3ka**, **3na**) resulted in high yields regardless of the identity of the halogen (F, Cl, Br, and I), and the reaction proceeded smoothly with both 2-bromo- and 3-bromobenzaldehydes (**3la**, **3ma**), allowing further diversification of the products via cross-coupling. Electron-withdrawing groups, including trifluoromethyl (**3oa**), methyl ester (**3pa**), nitrile (**3qa**), and sulfonate (**3ra**), were also tolerated, resulting in moderate to high yields. Notably, the pinacol boronic ester (**3sa**) was compatible with the reaction conditions, suggesting the potential for further elaboration by Suzuki coupling.

**Scheme 1:**
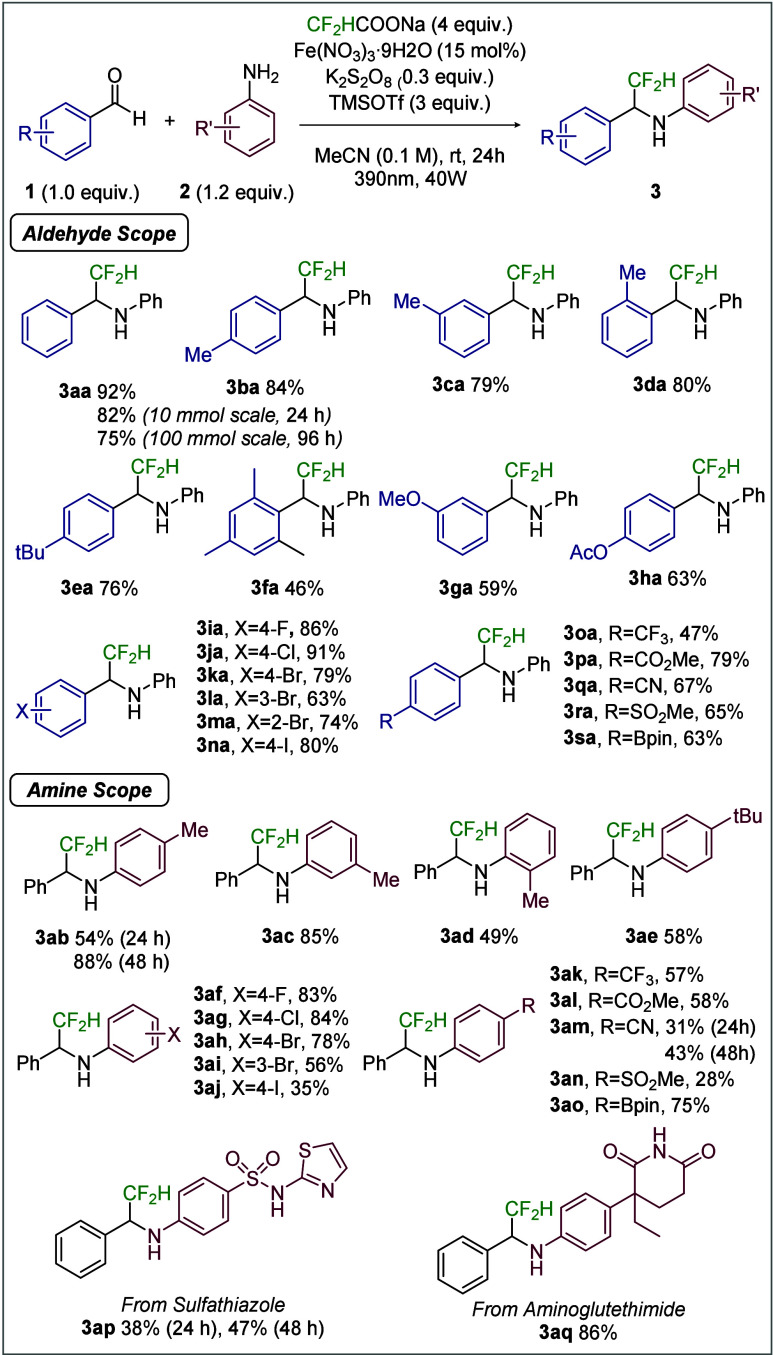
Substrate Scope and Applications^a^

a Reactions were performed on a 0.2 mmol scale with aryl aldehyde (0.20 mmol), aryl amine (0.24 mmol, 1.2 equiv), sodium difluoroacetate (4.0 equiv), iron nitrate nonahydrate (15 mol %), potassium persulfate (30 mol %), trimethylsilyl trifluoromethanesulfonate (3.0 equiv), and acetonitrile (2 mL, 0.1 M for aldehyde). The reactions were irradiated with 390 nm Kessil LEDs for 24 h at 100% intensity (40 W) under an ethanol coolant (20 °C). All yields are the isolated yields.

With respect to the amine series, the para-methyl aniline (**3ab**) did not reach complete conversion after 24 h. Increasing the reaction time to 48 h resulted in a substantial increase in the yield, indicating that the transformation proceeded more slowly for this substrate. The meta-methyl aniline (**3ac**) resulted in a high yield under the standard conditions, whereas the ortho-methyl analog (**3ad**) afforded a lower yield, which we attributed to steric hindrance that inhibited imine formation. Halogen-substituted anilines bearing F, Cl, or Br (**3af**–**3ai**) resulted in high yields, whereas the corresponding iodoaniline derivative (**3aj**) gave a diminished yield. Substrates bearing electron-withdrawing groups such as trifluoromethyl (**3ak**), methyl ester (**3al**), nitrile (**3am**), and sulfonate (**3an**) generally afforded moderate yields, which we attributed to reduced aryl amine nucleophilicity and, consequently, decreased accumulation of the imine intermediate. Consistent with this rationale, the yield of the nitrile-substituted substrate (**3am**) increased only marginally when the reaction time was increased, in contrast to the substantial improvement observed for the para-methyl-substituted substrate (**3ab**). Similar to the aldehyde series, the aryl amine bearing a pinacol boronic ester (**3ao**) also gave a high yield of the desired product, indicating that Suzuki coupling strategies can be applied to either aryl unit in the product. To further demonstrate the synthetic utility of this protocol, we examined its applicability to the late-stage functionalization of pharmaceutically relevant molecules bearing aryl amine motifs. The reaction was performed on sulfathiazole (**3ap**), a representative sulfonamide antibiotic, and aminoglutethimide (**3aq**), an aromatase and adrenal steroidogenesis inhibitor. Both substrates underwent difluoromethylation successfully.

Because LMCT-based difluoromethylation employs inexpensive and less toxic reagents under mild conditions, we further evaluated its scalability. The reaction was successfully performed on a decagram scale (100 mmol), affording the desired product (**3aa**) in a similarly high yield under operationally simple conditions, highlighting its practical potential.

To elucidate the reaction mechanism, we designed and conducted mechanistic experiments. First, to probe the formation of the CF_2_H radical, the reaction was conducted in the presence of TEMPO. Product formation was completely suppressed, and the TEMPO–CF_2_H adduct was detected by ^19^F NMR spectroscopy^23^ (Figure 2A). Additionally, when N-Benzylideneaniline (**4**) was used as the substrate, the desired product was obtained in high yield, supporting the involvement of an imine intermediate in the reaction (Figure 2B).

**Figure 2:**
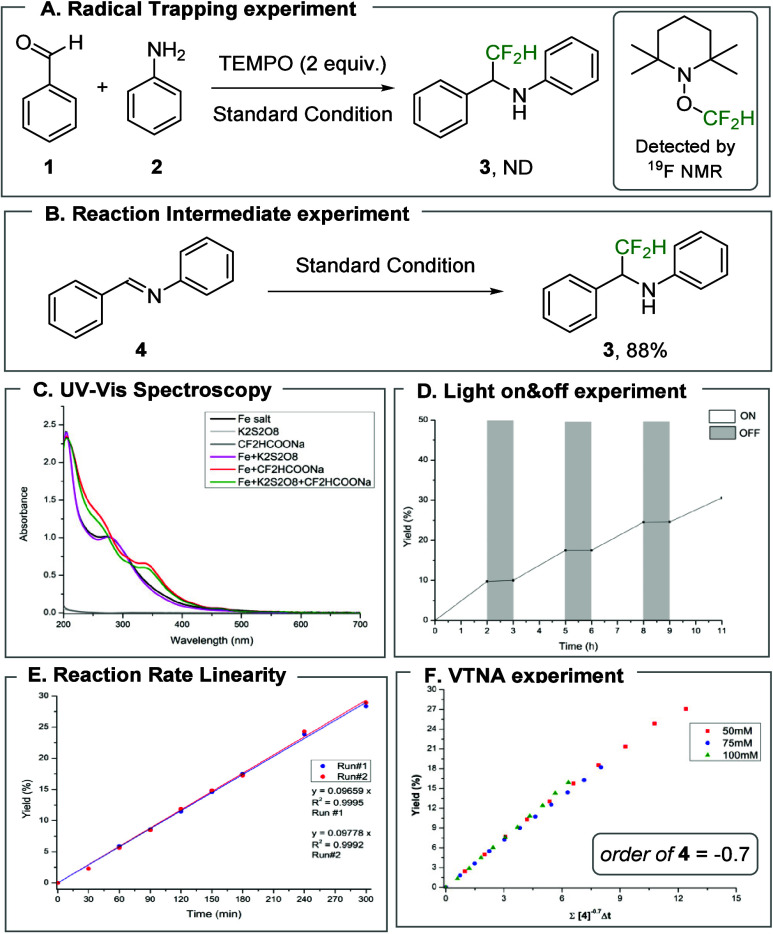
Mechanistic studies.

UV–vis spectra of Fe­(NO_3_)_3_·9H_2_O, CF_2_HCOONa, and K_2_S_2_O_8_, recorded both individually and in combination, revealed a new absorption band near 370 nm only when the iron salt and difluoroacetate were present together. This observation suggests the formation of an iron–difluoroacetate complex and accounts for the sharp decrease in yield upon irradiation at wavelengths above 400 nm (Figure 2C).

Light on–off experiments and quantum yield measurements were then performed to verify that the reaction proceeds via an LMCT-driven catalytic cycle rather than an N-centered radical-mediated chain process. Product formation occurred only under irradiation, and no further conversion was observed when the light was switched off, indicating that continuous photoexcitation is required (Figure 2D). Importantly, the quantum yield was exceptionally low (Φ = 0.006) compared with values reported for previous LMCT systems.^29,30^ This result effectively rules out a radical chain mechanism^31^ and instead supports turnover via an LMCT-driven catalytic cycle (see the Supporting Information (SI), Section 4.E).

To examine whether the proposed N-centered radical intermediate could oxidize Fe­(II) to enable catalyst turnover, cyclic voltammetry was performed. The Fe­(III/II) redox couple was observed at – 0.535 V (vs SCE), whereas the N-centered radical/product redox couple was located at 0.796 V (vs SCE), which is more positive than the Fe­(III/II) couple (see SI, Section 4.F). This redox relationship indicates that single-electron transfer from the Fe­(II) species to the N-centered radical is thermodynamically feasible and that regeneration of the iron catalyst via SET is therefore possible.

Next, we conducted a kinetic study to gain further insight into the mechanism of LMCT-driven radical addition. Monitoring the reaction progress revealed that the reaction rate remained essentially constant during the first 5 h despite increasing conversion (Figure 2E). To understand this kinetic behavior, we examined the dependence of the reaction rate on the reaction components. Variable time normalization analysis (VTNA) showed negative fractional reaction orders for the benzaldehyde (−0.6), aniline (−0.5), and imine (−0.7), suggesting substrate-induced inhibition within the catalytic cycle^32,33^ (Figure 2F, see SI, Section 4.H). Among the other reaction components, the reaction rate was first order only with respect to the iron catalyst (see SI, Section 4.I). Additional experiments indicated that aniline is selectively protonated under strongly acidic reaction conditions, whereas the imine remains neutral in the reaction mixture. A ^1^H NMR titration study further revealed that the imine intermediate can reversibly associate with the iron complex, potentially forming an off-cycle imine-iron complex that may attenuate the LMCT process^34–41^ (see SI, Section 4.J-K for details). Based on our mechanistic studies, we propose an iron-mediated LMCT catalytic cycle for the multicomponent difluoromethylation.

In conclusion, we report an LMCT-enabled iron-catalyzed platform for the direct multicomponent radical difluoromethylation of imines using inexpensive difluoroacetate salts as a CF_2_H source. The reaction affords α-CF_2_H amines with broad functional group tolerance, enabling late-stage functionalization and scalable synthesis of pharmaceutically relevant structures. This strategy establishes a practical platform for the efficient construction of α-CF_2_H amine motifs.

## Supplementary Material



## Data Availability

The data underlying this study are available in the published article and its Supporting Information.

## Notes

Optimization of reaction conditions, experimental procedures, characterization data, mechanistic investigation, computational studies, and NMR spectra (PDF)
