# Supplementary material for: Photoinduced Multicomponent Difluoromethylation of Imines via Iron-Mediated Ligand-to-Metal Charge Transfer
Source: Org Lett. 2026 Mar 26;28(14):4615–20. doi: 10.1021/acs.orglett.6c01025 (PMC13077681; doi:10.1021/acs.orglett.6c01025)

Supporting Information

Photoinduced Multicomponent Difluoromethylation of Imines via Iron-Mediated Ligand-to-Metal Charge Transfer

Hyungwoo Choi,[a] Jinwoo Lee,[a] Seok Beom Lee,[a] Sangmyung Han,[a] Joonseok Jang,[a] and Suckchang Hong[a,b]*

[a] Research Institute of Pharmaceutical Sciences, College of Pharmacy, Seoul National University, Seoul 08826, Republic of Korea

[b] Natural Products Research Institute, College of Pharmacy, Seoul National University, Seoul 08826, Republic of Korea

* Corresponding authors. E-mails: schong17@snu.ac.kr (S.Hong)

Table of Contents

1. General Information ---------- 3

2. Optimization of Reaction Conditions ---------- 4

2.A. General Procedure A for difluoromethylation of imines ---------- 4

2.B-L. Initial Screening and Optimization tables ---------- 5

3. Synthetic Examples ---------- 9

3.A. Scope of Aldehydes ---------- 9

3.B. Scope of Amines ---------- 20

3.C. Late-Stage Functionalization ---------- 29

3.D. Low yielding or Unsuccessful Substrates ---------- 30

3.E. Scale-Up Reaction ---------- 31

4. Mechanistic Investigation ---------- 33

4.A. Radical Trapping Experiment ---------- 33

4.B. Reaction Intermediate Experiment ---------- 35

4.C. Photochemical Measurements ---------- 36

4.D. Light ON/OFF experiment ---------- 37

4.E. Quantum Yield measurement ---------- 38

4.F. Cyclic Voltammetry measurement ---------- 41

4.G. Time-Course Reaction Profiles ---------- 43

4.H. Variable Time Normalization Analysis (VTNA) ---------- 45

4.I. Kinetic studies to Initial Rate Law Determination ---------- 55

4.J. Evidence for selective protonation of aniline under the reaction conditions ---------- 59

4.K. NMR Titration Experiment ---------- 61

5. Computational Studies ---------- 63

5.A. General Computational Information ---------- 63

5.B. Energy components from DFT calculations ---------- 63

5.C. DFT Energy Landscape ---------- 64

5.D. Cartesian Coordinates and Structures ---------- 64

6. References ---------- 69

7. NMR Spectra Copies ---------- 71

1. General Information

All commercially available reagents and solvents were purchased from Sigma-Aldrich, TCI, Alfa-Aesar, Acros, BLD pharm, Combi-blocks, Daejung, and Samchun and used without further purification unless otherwise noted. Dichloromethane (DCM), diethyl ether (Et_2O), tetrahydrofuran (THF), acetonitrile (MeCN) were purified and collected under argon using a Glass Contour Solvent Purification System. All reactions were carried out in oven-dried round-bottom flask, sealed tubes and vials. Reactions were monitored by thin layer chromatography (TLC) on silica gel 60 F254 plate (Merck, Darmstadt, Germany) using UV illumination at 254 nm (VL-4.LC, Vilber Lourmat, Eberhardzell, Germany). Flash column chromatography was performed on silica gel (230~400 mesh; Zeochem, Lake Zurich, Switzerland), using mixture of hexane and ethyl acetate as eluents. Melting points were measured on a Büchi B-540 melting point apparatus and were not corrected. Nuclear magnetic resonance (^{1}H-NMR, ^{13}C-NMR, and ^{19}F-NMR) spectra were measured on JEOL JNM-ECZ400s [400 MHz (^{1}H), 100 MHz (^{13}C), 376 MHz (^{19}F)] spectrometer. The chemical shifts are given in parts per million (ppm) on the delta (δ) scale. The solvent peak was used as a reference value, for ^{1}H NMR: $CDCl_3$ = 7.26 ppm, DMSO-d_6 = 2.50 ppm; for ^{13}C NMR: $CDCl_3$ = 77.16 ppm, DMSO-d_6 = 39.52 ppm. Coupling constants (J) are expressed in hertz (Hz). IR spectra were recorded on a JASCO, FT/IR-4200 Infrared spectrophotometer and are reported as cm^{-1}. All high-resolution mass spectra (HR-MS) were acquired using fast atom bombardment (FAB) ionization method on a JMS-700 MStation mass spectrometer (JEOL, Tokyo, Japan).

Setup for Photoreactor: Reactions were carried out using an LED lamp (Kessil PR160L-390nm, λ_{max} = 390 nm). The EvoluChem PhotoRedOx Box TC^{TM} (Temperature-Controlled) was connected to a recirculating chiller (ss), and ethanol was circulated through the photoreactor box to maintain the desired temperature (20–25 °C). The Kessil lamp was installed in the designated port of the photoreactor box, and the reaction vials were placed in a plastic holder located 5 cm from the lamp. The interior of the photoreactor box was lined with mirrors to reflect light toward the vials.

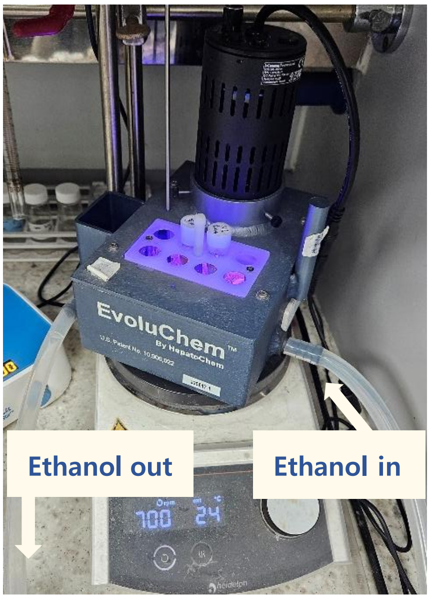

Figure 1. Photograph of the photochemical reaction setup with the coolant circulation.

2. Optimization of Reaction Conditions

A. General Procedure A for difluoromethylation of imines

The aldehyde (if liquid), amine (if liquid), and MeCN were degassed with an Ar balloon for at least 20 min prior to use. In an oven-dried 4 mL vial equipped with a Teflon®-coated magnetic stir bar, $CF_2HCOONa$ (94.4 mg, 4.0 equiv.), $K_2S_2O_8$ (16.2 mg, 0.30 equiv.) and $Fe(NO_3)_3{\cdot}9H_2O$ (12.1 mg, 15 mol%) were added. The vial was evacuated and backfilled with Ar gas (repeated 3 times), followed by addition of aldehyde (**1**, 0.2 mmol, 1.0 equiv.), amine (**2**, 0.24 mmol, 1.2 equiv.) and MeCN (2 mL, 0.10 M with respect to the aldehyde). If the aldehyde or amine was a solid, it was added prior to Ar charging. The mixture was stirred (700 rpm) for 3 min to dissolve the iron complex, followed by addition of TMSOTf (0.11 mL, 3.0 equiv.). The rubber septum was quickly replaced with a sealing cap, which was tightened securely to maintain an Ar atmosphere, and the vial was then sealed with Parafilm. The reaction mixture was placed in a photoreactor equipped with a 40 W, 390 nm Kessil lamp and stirred at 700 rpm for 24 h under ethanol coolant circulation to maintain room temperature. After completion, the reaction mixture was diluted with DCM, washed with saturated aqueous Na_2CO_3, and the layers were separated. The aqueous layer was extracted with DCM twice. The combined organic layers were dried with anhydrous MgSO4, filtered, and concentrated under reduced pressure. Dibromomethane (0.10 mmol, 7 μL) was added to the crude mixture as an internal standard for NMR analysis. The yields were determined by relative integration of dibromomethane (δ 4.93 ppm, s, 2H, normalized to 2.0) and product **3** (δ 4.73 ppm, td, 1H).

B. Initial Screening

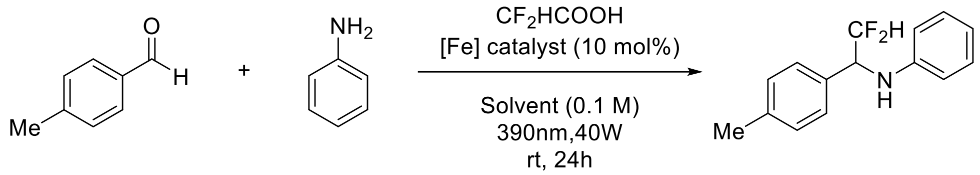

1 (1.0 equiv.) **2** (1.2 equiv.) **3**

Entry	DFA salt equiv.	Iron Catalyst	additives	Solvent	Yield
1	3	$Fe(acac)_3$	-	MeCN	5 %
2	3	$Fe(NO_3)_3{\cdot}9H_2O$	-	MeCN	15 %
3	3	$FeCl_3$	-	MeCN	2 %
4	3	$FeBr_3$	-	MeCN	ND
5	3	$Fe(NO_3)_3{\cdot}9H_2O$	Na_2CO_3 30mol%	MeCN	10%
6	3	$Fe(NO_3)_3{\cdot}9H_2O$	Na_2CO_3 60mol%	MeCN	10%
7	3	$Fe(NO_3)_3{\cdot}9H_2O$	Na_2CO_3 100mol%	MeCN	10%
8	3	$Fe(NO_3)_3{\cdot}9H_2O$	Molecular sieve 20mg	MeCN	11%
9	3	$Fe(NO_3)_3{\cdot}9H_2O$	Na_2SO_4 (anhydrous) 10mol%	MeCN	11%
10	3	$Fe(NO_3)_3{\cdot}9H_2O$	$MgSO_4$ (anhydrous) 10mol%	MeCN	14%

***Notes**: We initially based our reaction design on the conditions reported by the West group.[1] Accordingly, we employed CF_2HCOOH and Na_2CO_3. Then we considered removing water present in the reaction system, including that introduced by the hydrated iron salt and generated during imine formation; however, these modifications did not lead to a dramatic improvement.

C. Equivalent Iron catalyst loading

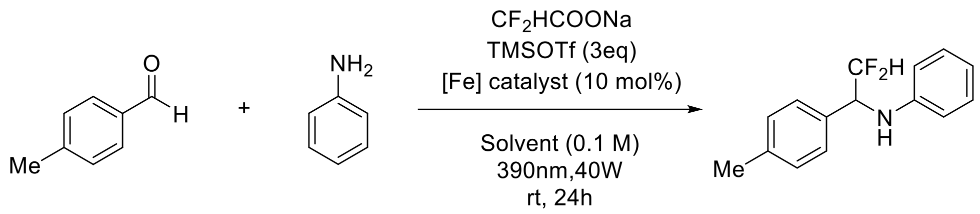

1 (1.0 equiv.) **2** (1.2 equiv.) **3**

Entry	DFA salt equiv.	Iron Catalyst	Iron catalyst equiv.	Solvent	Yield
1	3	$Fe(NO_3)_3{\cdot}9H_2O$	10 mol%	MeCN	ND
2	3	$Fe(NO_3)_3{\cdot}9H_2O$	10 mol%	DMSO	12%
3	4.5	$Fe(NO_3)_3{\cdot}9H_2O$	10 mol%	DMSO	13%
4	3	$Fe(NO_3)_3{\cdot}9H_2O$	30 mol%	DMSO	35%
5	3	$Fe(NO_3)_3{\cdot}9H_2O$	100 mol% (1 equiv.)	DMSO	41%
6	5	$Fe(NO_3)_3{\cdot}9H_2O$	100 mol% (1 equiv.)	DMSO	62%
7	5	$Fe(NO_3)_3{\cdot}9H_2O$	100 mol% (1 equiv.)	MeCN	99%
8	4	$Fe(NO_3)_3{\cdot}9H_2O$	50 mol%	MeCN	99%

***Notes:** As discussed in the main text, during our screening studies we hypothesized and subsequently confirmed that the iron loading was a determining factor governing the reaction yield. By switching the solvent system back from DMSO to MeCN, we were able to achieve a higher yield.

D. Catalytic Iron cycle with external oxidant

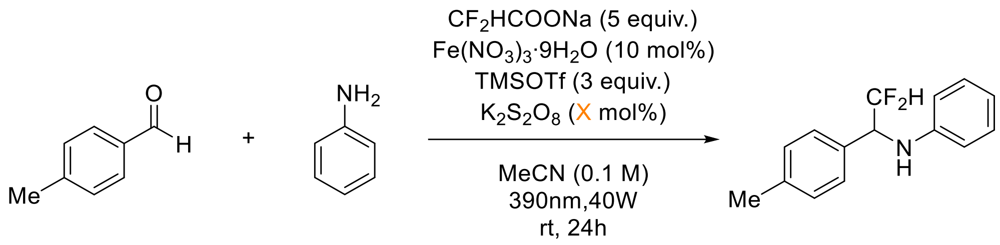

1 (1.0 equiv.) **2** (1.2 equiv.) **3**

Entry	$K_2S_2O_8$ equiv.	Yield
1	1.5	99 %
2	1.2	95 %
3	1.0	87 %
4	0.6	86 %
5	0.3	87 %
6	0.1	75 %

***Notes:** We anticipated that an external oxidant would enable catalytic iron turnover, and that efficient cycling would allow substoichiometric oxidant loadings. Given the reported compatibility of persulfate oxidants with LMCT systems, we initially employed $K_2S_2O_8$ and subsequently performed screening while gradually decreasing the oxidant loading.

E. Optimization of Iron (III) catalyst

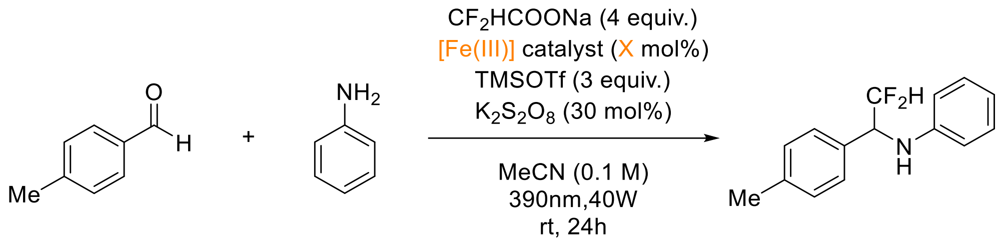

1 (1.0 equiv.) **2** (1.2 equiv.) **3**

Entry	Iron catalyst (equiv.)	Yield
1	$Fe(NO_3)_3 \cdot 9H_2O$ (15 mol%)	99 %
2	$FeCl_3 \cdot 6H_2O$ (15 mol%)	66 %
3	$Fe(OTf)_3$ (15 mol%)	60 %
4	$Fe(acac)_3$ (15 mol%)	12 %
5	$Fe(NO_3)_3 \cdot 9H_2O$ (10 mol%)	65 %
6	$Fe(NO_3)_3 \cdot 9H_2O$ (20 mol%)	94 %

F. Optimization of Solvent and Concentration

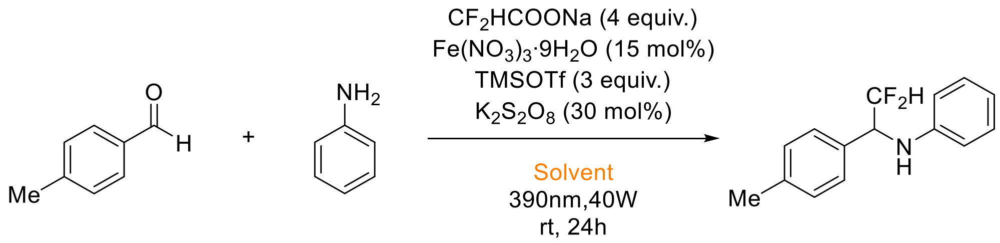

1 (1.0 equiv.) **2** (1.2 equiv.) **3**

Entry	Solvent	Yield
1	MeCN (0.1 M)	99 %
2	DMSO (0.1 M)	45 %
3	Ethyl Acetate (0.1 M)	62 %
4	DCM (0.1 M)	N.D
5	THF (0.1 M)	N.D
6	MeCN (0.05 M)	78 %
7	MeCN (0.2 M)	40 %

G. Optimization of Sodium difluoroacetate equivalent

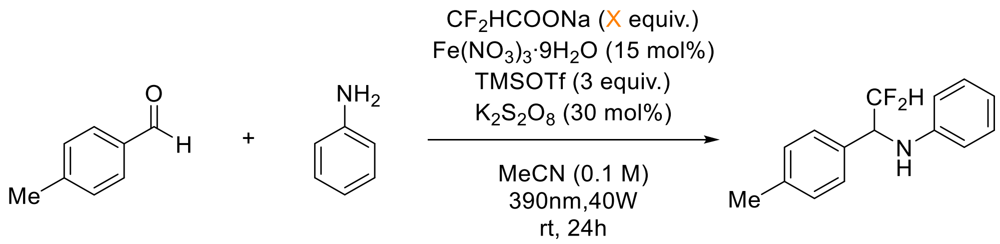

1 (1.0 equiv.) **2** (1.2 equiv.) **3**

Entry	$CF_2HCOONa$ salt equiv.	Yield
1	3	18 %
2	4	99 %
3	5	89 %

H. Optimization of TMSOTf equivalent

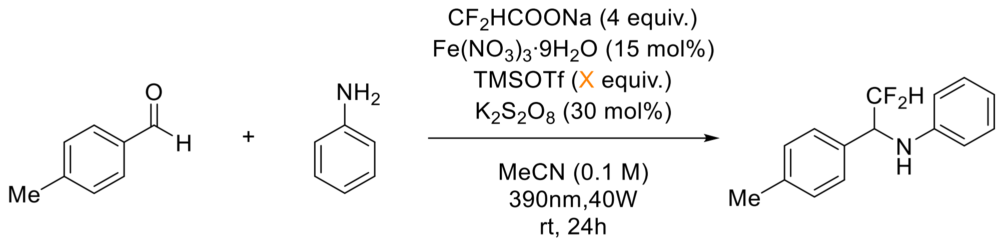

1 (1.0 equiv.) **2** (1.2 equiv.) **3**

Entry	TMSOTf equiv.	Yield
1	3	99 %
2	2	64 %
3	1	37 %
4	0	18 %

I. Optimization of $K_2S_2O_8$ equivalent

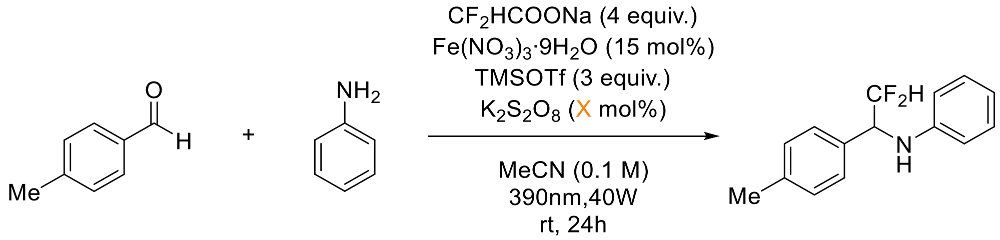

1 (1.0 equiv.) **2** (1.2 equiv.) **3**

Entry	$K_2S_2O_8$ equiv.	Yield
1	10 mol%	75 %
2	20 mol%	94 %
3	30 mol%	99 %
4	50 mol %	99 %

J. Optimization of Oxidant additive

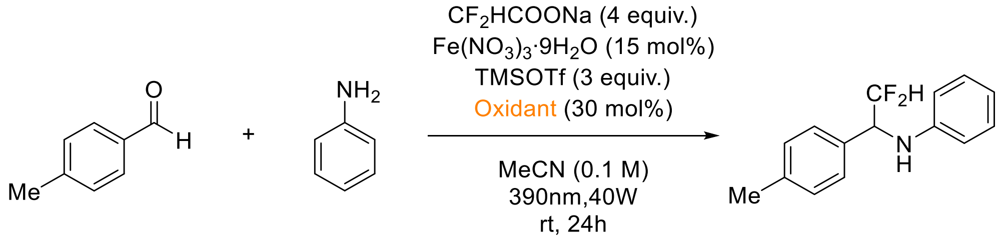

1 (1.0 equiv.) **2** (1.2 equiv.) **3**

Entry	Oxidant	Yield
1	$K_2S_2O_8$	99 %
2	$Na_2S_2O_8$	54 %
3	Bz_2O_2	44 %
4	H_2O_2	ND
5	NFSI	37 %
6	DTBP	21 %
7	DDQ	8 %
8	Oxone® ($2KHSO_5{\cdot}KHSO_4{\cdot}K_2SO_4$)	35 %

K. Optimization of light source

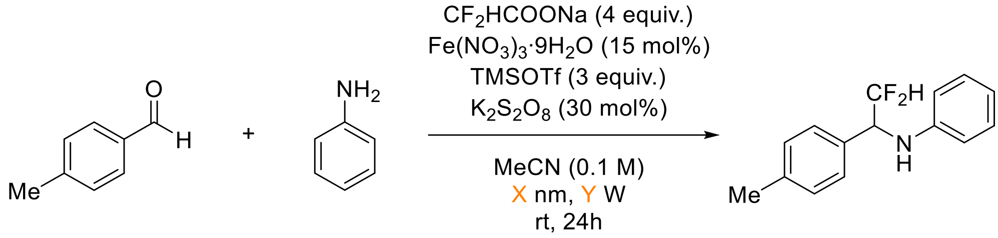

1 (1.0 equiv.) **2** (1.2 equiv.) **3**

Entry	Wavelength, Intensity	Yield
1	370 nm, 40 W	98 %
2	390 nm, 40 W	99 %
3	427 nm, 40 W	46 %
4	456 nm, 40 W	19 %
5	390 nm, 30 W	40 %
6	390 nm, 20 W	20 %

L. Control Experiments

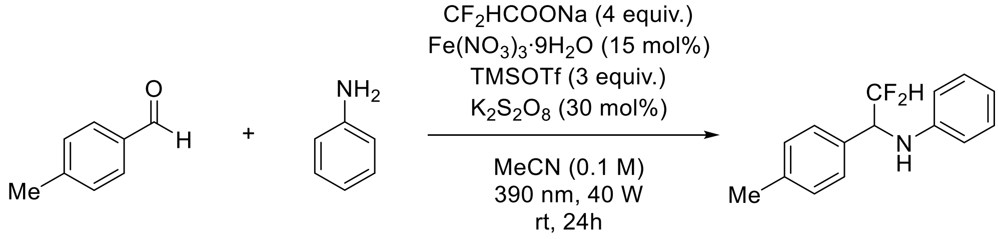

1 (1.0 equiv.) **2** (1.2 equiv.) **3**

Entry	Deviation from above	Yield
1	No iron salt	ND
2	No light source	ND
3	Under open-air condition	ND

***Notes:** ^{1}H NMR analysis of the crude reaction mixture showed that the unconverted substrate was present predominantly as the imine, occasionally with trace amounts of the aldehyde (< 3%).

3. Synthetic Examples

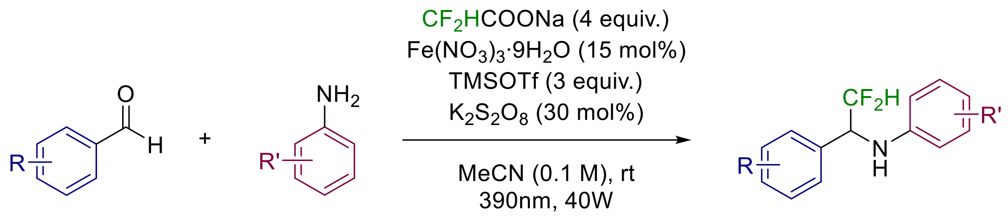

1 (0.2 mmol, 1.0 equiv.) **2** (1.2 equiv.) **3**

General Procedure A for difluoromethylation of imines

The aldehyde (if liquid), amine (if liquid), and MeCN were degassed with an Ar balloon for at least 20 min prior to use. In an oven-dried 4 mL vial equipped with a Teflon®-coated magnetic stir bar, $CF_2HCOONa$ (94.4 mg, 4.0 equiv.), $K_2S_2O_8$ (16.2 mg, 0.30 equiv.) and $Fe(NO_3)_3{\cdot}9H_2O$ (12.1 mg, 15 mol%) were added. The vial was evacuated and backfilled with Ar gas (repeated 3 times), followed by addition of aldehyde (**1**, 0.2 mmol, 1.0 equiv.), amine (**2**, 0.24 mmol, 1.2 equiv.) and MeCN (2 mL, 0.10 M with respect to the aldehyde). If the aldehyde or amine was a solid, it was added prior to Ar charging. The mixture was stirred (700 rpm) for 3 min to dissolve the iron complex, followed by addition of TMSOTf (0.11 mL, 3.0 equiv.). The rubber septum was quickly replaced with a sealing cap, which was tightened securely to maintain an Ar atmosphere, and the vial was then sealed with Parafilm. The reaction mixture was placed in a photoreactor equipped with a 40 W, 390 nm Kessil lamp and stirred at 700 rpm for 24 h under ethanol coolant circulation to maintain room temperature. After completion, the reaction mixture was diluted with DCM, washed with saturated aqueous Na_2CO_3, and the layers were separated. The aqueous layer was extracted with DCM twice. The combined organic layers were dried with anhydrous MgSO4, filtered, and concentrated under reduced pressure. Purification by flash column chromatography afforded the corresponding α-difluoromethylated amines (**3**).

A. Scope of Aldehydes

N-(2,2-difluoro-1-phenylethyl)aniline (3aa)

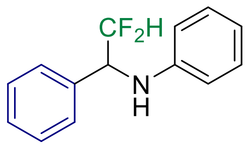

3aa

Prepared according to General Procedure A using benzaldehyde (21.2 mg, 0.2 mmol, 1.0 equiv.) and aniline (22.4 mg, 0.24 mmol, 1.2 equiv.) as starting materials. After purification by flash column chromatography (hexane: EtOAc = 100 : 2), the title compound was isolated as a yellow oil (43.0mg, 92.2 % yield).

^{1}H-NMR (400MHz, $CDCl_3$) δ 7.45-7.33 (m, 5H), 7.17-7.13 (m, 2H), 6.75 (t, J = 7.3 Hz, 1H), 6.62 (d, J = 7.8 Hz, 2H), 6.01 (td, J = 55.8, 2.7 Hz, 1H), 4.73 (td, J = 13.3, 2.7 Hz, 1H)

^{13}C-NMR (100MHz, $CDCl_3$) δ 146.2, 135.5 (d, J = 2 Hz), 129.4, 129.0, 128.7, 127.9, 118.9, 115.9 (t, J = 245.6 Hz), 114.1, 60.4 (t, J = 22.1 Hz)

^{19}F-NMR (376MHz, $CDCl_3$) δ -125.9 (dddd, J = 828.3, 278.5, 56.0, 13.4 Hz)

HRMS (FAB) m/z: $[M]^+$ Calcd for $C_{14}H_{13}F_2N^+$ 233.1016; Found 233.1012.

IR (neat) ν 3415, 3057, 3030, 1604, 1509, 1317, 1296, 1122, 1074, 1053, 751, 714, 693 cm^{-1}

N-(2,2-difluoro-1-(p-tolyl)ethyl)aniline (3ba)

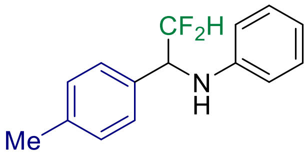

3ba

Prepared according to General Procedure A using p-tolualdehyde (24.0 mg, 0.2 mmol, 1.0 equiv.) and aniline (22.4 mg, 0.24 mmol, 1.2 equiv.) as starting materials. After purification by flash column chromatography (hexane: EtOAc = 100 : 2), the title compound was isolated as a yellow oil (41.7mg, 84.4 % yield).

^{1}H-NMR (400MHz, $CDCl_3$) δ 7.32 (d, J = 7.8 Hz, 2H), 7.20 (d, J = 7.8 Hz, 2H), 7.15 (t, J = 8.0 Hz, 2H), 6.75 (t, J = 7.4 Hz, 1H), 6.63 (d, J = 7.8 Hz, 2H), 5.99 (td, J = 55.8, 2.9 Hz, 1H), 4.69 (td, J = 13.2, 3.1 Hz, 1H), 2.36 (s, 3H)

^{13}C-NMR (100MHz, $CDCl_3$) δ 146.3, 138.5, 132.5 (d, J = 2 Hz), 129.7, 129.4, 127.7, 118.8, 116.0 (t, J = 246.1 Hz), 114.1, 60.1 (t, J = 21.6 Hz), 21.3

^{19}F-NMR (376MHz, $CDCl_3$) δ -125.9 (dddd, J = 788.9, 278.9, 56.4, 13.0 Hz)

HRMS (FAB) m/z: $[M]^+$ Calcd for $C_{15}H_{15}F_2N^+$ 247.1173; Found 247.1171.

IR (neat) ν 3413, 3054, 3026, 2925, 2858, 1605, 1508, 1317, 1303, 1123, 1075, 1053, 813, 750, 692 cm^{-1}

N-(2,2-difluoro-1-(m-tolyl)ethyl)aniline (3ca)

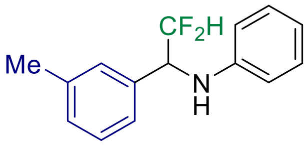

3ca

Prepared according to General Procedure A using m-tolualdehyde (24.0 mg, 0.2 mmol, 1.0 equiv.) and aniline (22.4 mg, 0.24 mmol, 1.2 equiv.) as starting materials. After purification by flash column chromatography (hexane: EtOAc = 100 : 2), the title compound was isolated as a yellow oil (38.8mg, 78.5 % yield).

^{1}H-NMR (400MHz, $CDCl_3$) δ 7.29 (d, J = 7.3 Hz, 1H), 7.23-7.13 (m, 5H), 6.75 (t, J = 7.3 Hz, 1H), 6.64-6.62 (m, 2H), 5.99 (td, J = 55.8, 3.2 Hz, 1H), 4.67 (td, J = 13.3, 2.7 Hz, 1H), 2.37 (s, 3H)

^{13}C-NMR (100MHz, $CDCl_3$) δ 146.3, 138.8, 135.5 (d, J = 2 Hz), 129.5, 129.4, 128.9, 128.5, 124.9, 118.8, 116.0 (t, J = 246.1 Hz), 114.0, 60.4 (t, J = 21.6 Hz), 21.6

^{19}F-NMR (376MHz, $CDCl_3$) δ -125.8 (dddd, J = 1010.0, 278.2, 55.6, 13.0 Hz)

HRMS (FAB) m/z: $[M]^+$ Calcd for $C_{15}H_{15}F_2N^+$ 247.1173; Found 247.1171.

IR (neat) ν 3414, 3054, 3026, 1604, 1508, 1379, 1314, 1252, 1120, 1076, 1054, 787, 750, 692 cm^{-1}

N-(2,2-difluoro-1-(o-tolyl)ethyl)aniline (3da)

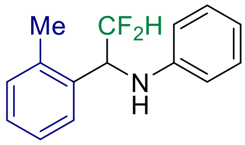

3da

Prepared according to General Procedure A using o-tolualdehyde (24.0 mg, 0.2 mmol, 1.0 equiv.) and aniline (22.4 mg, 0.24 mmol, 1.2 equiv.) as starting materials. After purification by flash column chromatography (hexane: EtOAc = 100 : 2), the title compound was isolated as a yellow oil (39.4mg, 79.7 % yield).

^{1}H-NMR (400MHz, $CDCl_3$) δ 7.44 (d, J = 5.9 Hz, 1H), 7.25-7.20 (m, 3H), 7.17-7.13 (m, 2H), 6.75 (t, J = 7.3 Hz, 1H), 6.58 (d, J = 8.7 Hz, 2H), 6.01 (td, J = 55.8, 3.2 Hz, 1H), 5.00 (td, J = 12.6, 3.4 Hz, 1H), 2.48 (s, 3H)

^{13}C-NMR (100MHz, $CDCl_3$) δ 146.3, 136.5, 133.9, 131.0, 129.4, 128.5, 127.0, 126.8, 118.8, 116.0 (t, J = 246.1 Hz), 113.8, 56.4 (t, J = 21.6 Hz), 19.7

^{19}F-NMR (376MHz, $CDCl_3$) δ -125.4 (dddd, J = 1087.0, 278.5, 56.0, 12.6 Hz)

HRMS (FAB) m/z: $[M]^+$ Calcd for $C_{15}H_{15}F_2N^+$ 247.1173; Found 247.1171.

IR (neat) ν 3413, 3054, 3025, 2976, 1603, 1508, 1315, 1296, 1128, 1076, 1054, 750, 729, 691 cm^{-1}

N-(1-(4-(tert-butyl)phenyl)-2,2-difluoroethyl)aniline (3ea)

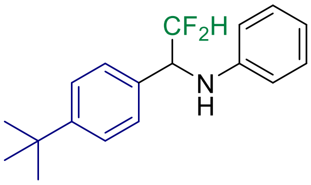

3ea

Prepared according to General Procedure A using 4-tert-butylbenzaldehyde (35.6 mg, 0.2 mmol, 1.0 equiv.) and aniline (22.4 mg, 0.24 mmol, 1.2 equiv.) as starting materials. After purification by flash column chromatography (hexane: EtOAc = 100 : 3), the title compound was isolated as a brown oil (43.7mg, 75.5 % yield).

^{1}H-NMR (400MHz, $CDCl_3$) δ 7.42-7.29 (m, 4H), 7.18-7.12 (m, 2H), 6.74 (td, J = 7.3, 0.9 Hz, 1H), 6.63 (dt, J = 8.7, 1.6 Hz, 2H), 5.99 (td, J = 55.9, 2.9 Hz, 1H), 4.70 (td, J = 13.4, 2.9 Hz, 1H), 1.31 (s, 9H)

^{13}C-NMR (100MHz, $CDCl_3$) δ 151.6, 146.3, 132.4 (d, J = 2 Hz), 129.4, 127.5, 126.0, 118.8, 116.0 (t, J = 246.1 Hz), 114.0, 60.0 (t, J = 21.6 Hz), 34.7, 31.4

^{19}F-NMR (376MHz, $CDCl_3$) δ -125.8 (dddd, J = 930.2, 278.5, 56.0, 12.6 Hz)

HRMS (FAB) m/z: $[M]^+$ Calcd for $C_{18}H_{21}F_2N^+$ 289.1642; Found 289.1633.

IR (neat) ν 3416, 3056, 3027, 2964, 2905, 2869, 1605, 1508, 1380, 1364, 1269, 1126, 1075, 1054, 836, 749, 691 cm^{-1}

N-(2,2-difluoro-1-mesitylethyl)aniline (3fa)

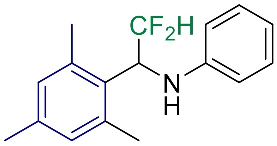

3fa

Prepared according to General Procedure A using mesitylaldehyde (29.6 mg, 0.2 mmol, 1.0 equiv.) and aniline (22.4 mg, 0.24 mmol, 1.2 equiv.) as starting materials. After purification by flash column chromatography (hexane: EtOAc = 100 : 2.5), the title compound was isolated as a yellow oil (25.2mg, 45.8 % yield).

^{1}H-NMR (400MHz, $CDCl_3$) δ 7.17-7.10 (m, 2H), 6.86 (s, 2H), 6.73 (t, J = 7.3 Hz, 1H), 6.57 (dd, J = 8.7, 0.9 Hz, 2H), 6.25-5.96 (m, 1H), 5.17-5.09 (m, 1H), 2.45 (s, 6H), 2.25 (s, 3H)

^{13}C-NMR (100MHz, $CDCl_3$) δ 146.8, 138.0, 137.1, 130.9, 129.4, 129.0 (t, J = 2 Hz), 118.6, 116.5 (t, J = 246.1 Hz), 113.4, 57.7 (t, J = 23.5 Hz), 21.5, 20.9

^{19}F-NMR (376MHz, $CDCl_3$) δ -118.9 (d, J = 54.9 Hz), -121.7 (dddd, J = 1040.4, 280.3, 56.4, 13.0 Hz), -127.1 (d, J = 52.0 Hz)

HRMS (FAB) m/z: $[M]^+$ Calcd for $C_{17}H_{19}F_2N^+$ 275.1486; Found 275.1485.

IR (neat) ν 3426, 2734, 2922, 1605, 1507, 1316, 1254, 1071, 1054, 852, 749, 691 cm^{-1}

N-(2,2-difluoro-1-(3-methoxyphenyl)ethyl)aniline (3ga)

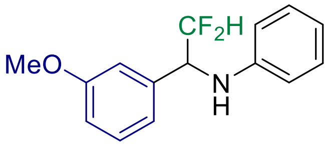

3ga

Prepared according to General Procedure A using 3-methoxybenzaldehyde (27.2 mg, 0.2 mmol, 1.0 equiv.) and aniline (22.4 mg, 0.24 mmol, 1.2 equiv.) as starting materials. After purification by flash column chromatography (hexane: EtOAc = 100 : 4), the title compound was isolated as a yellow oil (30.8mg, 58.5 % yield).

^{1}H-NMR (400MHz, $CDCl_3$) δ 7.30 (t, J = 7.8 Hz, 1H), 7.17-7.11 (m, 2H), 7.02-6.97 (m, 2H), 6.88 (dt, J = 8.2, 1.4 Hz, 1H), 6.76-6.72 (m, 1H), 6.63-6.60 (m, 2H), 5.99 (td, J = 55.8, 3.2 Hz, 1H), 4.68 (td, J = 13.2, 2.9 Hz, 1H), 3.80 (s, 3H)

^{13}C-NMR (100MHz, $CDCl_3$) δ 160.2, 146.2, 137.3 (d, J = 2.9 Hz), 130.1, 129.4, 120.1, 118.9, 115.9 (t, J = 246.6 Hz), 114.1, 113.9, 113.7, 60.4 (t, J = 21.6 Hz), 55.4

^{19}F-NMR (376MHz, $CDCl_3$) δ -125.8 (dddd, J = 1032.8, 279.2, 56.0, 13.4 Hz)

HRMS (FAB) m/z: $[M]^+$ Calcd for $C_{15}H_{15}F_2NO^+$ 263.1122; Found 263.1116.

IR (neat) ν 3406, 3055, 2963, 3024, 1603, 1508, 1496, 1314, 1256, 1140, 1076, 1050, 751, 693 cm^{-1}

4-(2,2-difluoro-1-(phenylamino)ethyl)phenyl acetate (3ha)

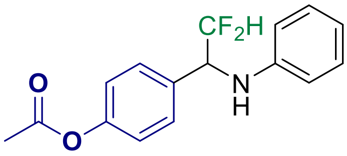

3ha

Prepared according to General Procedure A using 4-acetoxybenzaldehyde (32.8 mg, 0.2 mmol, 1.0 equiv.) and aniline (22.4 mg, 0.24 mmol, 1.2 equiv.) as starting materials. After purification by flash column chromatography (hexane: EtOAc = 100 : 12), the title compound was isolated as a yellow oil (36.6mg, 62.8 % yield).

^{1}H-NMR (400MHz, $CDCl_3$) δ 7.44 (d, J = 8.2 Hz, 2H), 7.14 (dd, J = 13.7, 8.2 Hz, 4H), 6.75 (t, J = 7.1 Hz, 1H), 6.60 (d, J = 7.8 Hz, 2H), 5.98 (td, J = 55.7, 2.4 Hz, 1H), 4.72 (t, J = 12.3 Hz, 1H), 4.39 (s, 1H), 2.30 (s, 3H)

^{13}C-NMR (100MHz, $CDCl_3$) δ 169.4, 150.9, 145.9, 133.0, 129.5, 128.9, 122.1, 119.0, 115.7 (t, J = 246.2 Hz), 114.0, 59.8 (t, J = 21.1 Hz), 21.3

^{19}F-NMR (376MHz, $CDCl_3$) δ -125.9 (dddd, J = 706.6, 279.6, 55.6, 13.0 Hz)

HRMS (FAB) m/z: $[M]^+$ Calcd for $C_{16}H_{15}F_2NO_2^+$ 291.1071; Found 291.1068.

IR (neat) ν 3396, 3056, 2925, 1757, 1605, 1507, 1371, 1205, 1075, 1054, 914, 752, 693 cm^{-1}

N-(2,2-difluoro-1-(4-fluorophenyl)ethyl)aniline (3ia)

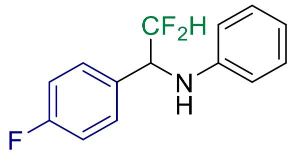

3ia

Prepared according to General Procedure A using 4-fluorobenzaldehyde (24.8 mg, 0.2 mmol, 1.0 equiv.) and aniline (22.4 mg, 0.24 mmol, 1.2 equiv.) as starting materials. After purification by flash column chromatography (hexane: EtOAc = 100 : 2.5), the title compound was isolated as a yellow oil (43.0mg, 85.6 % yield).

^{1}H-NMR (400MHz, $CDCl_3$) δ 7.41 (dd, *J* = 8.7, 5.5 Hz, 2H), 7.15 (td, *J* = 7.0, 1.7 Hz, 2H), 7.08 (tt, *J* = 9.0, 2.4 Hz, 2H), 6.76 (t, *J* = 7.3 Hz, 1H), 6.59 (d, *J* = 7.8 Hz, 2H), 5.98 (td, *J* = 55.8, 3.2 Hz, 1H), 4.70 (td, *J* = 13.3, 2.7 Hz, 1H)

^{13}C-NMR (100MHz, $CDCl_3$) δ 163.0 (d, *J* = 246.5 Hz), 145.8, 131.1, 129.6 (d, *J* = 8.6 Hz), 129.5, 119.1, 116.0 (d, *J* = 22.1 Hz), 115.6 (t, *J* = 246.5 Hz), 114.1, 59.7 (t, *J* = 21.6 Hz)

^{19}F-NMR (376MHz, $CDCl_3$) δ -113.3 (t, *J* = 7.2 Hz, 1F), -126.1 (m, 2F)

HRMS (FAB) m/z: $[M]^+$ Calcd for $C_{14}H_{12}F_3N^+$ 251.0922; Found 251.0923.

IR (neat) ν 3418, 3056, 3027, 2981, 1605, 1509, 1315, 1296, 1227, 1121, 1077, 1055, 834, 794, 752, 692 cm^{-1}

N-(1-(4-chlorophenyl)-2,2-difluoroethyl)aniline (3ja)

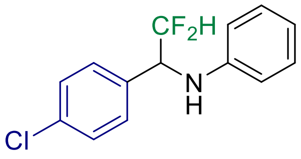

3ja

Prepared according to General Procedure A using 4-chlorobenzaldehyde (28.1 mg, 0.2 mmol, 1.0 equiv.) and aniline (22.4 mg, 0.24 mmol, 1.2 equiv.) as starting materials. After purification by flash column chromatography (hexane: EtOAc = 100 : 2.5), the title compound was isolated as a yellow oil (48.7mg, 91.0 % yield).

^{1}H-NMR (400MHz, $CDCl_3$) δ 7.40-7.35 (m, 4H), 7.15 (t, *J* = 7.8 Hz, 2H), 6.77 (t, *J* = 7.4 Hz, 1H), 6.59 (d, *J* = 7.8 Hz, 2H), 5.99 (td, *J* = 55.7, 2.9 Hz, 1H), 4.70 (td, *J* = 13.2, 2.9 Hz, 1H)

^{13}C-NMR (100MHz, $CDCl_3$) δ 145.7, 134.7, 133.9, 129.5, 129.3, 129.2, 119.2, 115.5 (t, *J* = 244.9 Hz), 114.1, 59.8 (t, *J* = 22.0 Hz)

^{19}F-NMR (376MHz, $CDCl_3$) δ -126.0 (dq, *J* = 55.6, 15.4 Hz)

HRMS (FAB) m/z: $[M]^+$ Calcd for $C_{14}H_{12}ClF_2N^+$ 267.0626; Found 267.0636.

IR (neat) ν 3416, 3055, 3027, 1604, 1508, 1493, 1315, 1296, 1124, 1091, 1078, 1056, 1014, 752, 692 cm^{-1}

N-(1-(4-bromophenyl)-2,2-difluoroethyl)aniline (3ka)

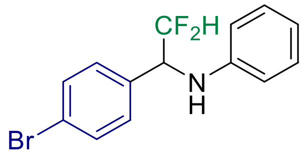

3ka

Prepared according to General Procedure A using 4-bromobenzaldehyde (37.0 mg, 0.2 mmol, 1.0 equiv.) and aniline (22.4 mg, 0.24 mmol, 1.2 equiv.) as starting materials. After purification by flash column chromatography (hexane: EtOAc = 100 : 2.5), the title compound was isolated as a yellow oil (49.6mg, 79.4 % yield).

^{1}H-NMR (400MHz, $CDCl_3$) ν 1H-NMR (400 MHz, CHLOROFORM-D) δ 7.53-7.50 (m, 2H), 7.31 (d, *J* = 8.3 Hz, 2H), 7.15 (dd, *J* = 8.3, 7.4 Hz, 2H), 6.77 (t, *J* = 7.4 Hz, 1H), 6.58 (d, *J* = 7.8 Hz, 2H), 5.98 (td, *J* = 55.7, 2.9 Hz, 1H), 4.68 (td, *J* = 13.2, 2.9 Hz, 1H), 4.43 (s, 1H)

^{13}C-NMR (100MHz, $CDCl_3$) δ 145.6, 134.4, 132.2, 129.6, 129.5, 122.8, 119.2, 115.4 (t, *J* = 244.9 Hz), 114.2, 59.9 (t, *J* = 22.0 Hz),

^{19}F-NMR (376MHz, $CDCl_3$) δ -126.0 (m)

HRMS (FAB) m/z: $[M]^+$ Calcd for $C_{14}H_{12}BrF_2N^+$ 311.0121; Found 311.0113.

IR (neat) ν 3413, 3055, 3027, 2978, 1604, 1508, 1489, 1315, 1295, 1123, 1073, 1056, 1012, 749, 692 cm^{-1}

N-(1-(3-bromophenyl)-2,2-difluoroethyl)aniline (3la)

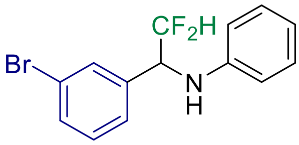

3la

Prepared according to General Procedure A using 3-bromobenzaldehyde (37.0 mg, 0.2 mmol, 1.0 equiv.) and aniline (22.4 mg, 0.24 mmol, 1.2 equiv.) as starting materials. After purification by flash column chromatography (hexane: EtOAc = 100 : 2.5), the title compound was isolated as a brown oil (39.0mg, 62.5 % yield).

^{1}H-NMR (400MHz, $CDCl_3$) δ 7.61 (s, 1H), 7.50 (dq, *J* = 8.0, 1.1 Hz, 1H), 7.39 (d, *J* = 7.8 Hz, 1H), 7.30-7.28 (m, 1H), 7.20-7.15 (m, 2H), 6.81-6.77 (m, 1H), 6.63-6.60 (m, 2H), 6.01 (td, *J* = 55.7, 3.0 Hz, 1H), 4.70 (td, *J* = 13.3, 2.7 Hz, 1H), 4.41 (s, 1H)

^{13}C-NMR (100MHz, $CDCl_3$) δ 145.7, 137.9, 132.0, 131.0, 130.6, 129.5, 126.6, 123.2, 119.2, 115.5, 114.0 (t, *J* = 246.5 Hz), 60.0 (t, *J* = 22.1 Hz)

^{19}F-NMR (376MHz, $CDCl_3$) δ -125.9 (dddd, *J* = 423.0, 280.7, 55.3, 12.6 Hz)

HRMS (FAB) m/z: $[M]^+$ Calcd for $C_{14}H_{12}BrF_2N^+$ 311.0121; Found 311.0112.

IR (neat) ν 3414, 3056, 3026, 2979, 1604, 1508, 1476, 1428, 1379, 1313, 1292, 1253, 1199, 1122, 1073, 1057, 751, 692 cm^{-1}

N-(1-(2-bromophenyl)-2,2-difluoroethyl)aniline (3ma)

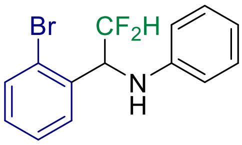

3ma

Prepared according to General Procedure A using 2-bromobenzaldehyde (37.0 mg, 0.2 mmol, 1.0 equiv.) and aniline (22.4 mg, 0.24 mmol, 1.2 equiv.) as starting materials. After purification by flash column chromatography (hexane: EtOAc = 100 : 2.5), the title compound was isolated as a brown oil (46.2mg, 74.0 % yield).

^{1}H-NMR (400MHz, $CDCl_3$) δ 7.62 (dd, *J* = 8.0, 1.1 Hz, 1H), 7.48 (dd, *J* = 7.8, 1.4 Hz, 1H), 7.31 (td, *J* = 7.5, 1.1 Hz, 1H), 7.20 (td, *J* = 7.7, 1.7 Hz, 1H), 7.17-7.12 (m, 2H), 6.76-6.72 (m, 1H), 6.56 (dd, *J* = 8.7, 0.9 Hz, 2H), 6.23-5.95 (m, 1H), 5.30 (ddd, *J* = 17.4, 10.1, 1.8 Hz, 1H)

^{13}C-NMR (100MHz, $CDCl_3$) δ 145.7, 134.6, 134.5, 133.3, 130.3, 129.5, 128.3, 124.1, 118.9, 114.8 (t, *J* = 247.0 Hz), 113.8, 59.0 (t, *J* = 20.7 Hz)

^{19}F-NMR (376MHz, $CDCl_3$) δ -123.1 (ddd, *J* = 276.7, 55.6, 9.4 Hz, 1F), -131.9 (ddd, *J* = 277.4, 54.9, 17.3 Hz, 1F)

HRMS (FAB) m/z: $[M]^+$ Calcd for $C_{14}H_{12}BrF_2N^+$ 311.0121; Found 311.0114.

IR (neat) ν 3422, 3057, 3025, 1604, 1508, 1440, 1380, 1313, 1295, 1125, 1079, 1059, 876, 750, 691 cm^{-1}

N-(2,2-difluoro-1-(4-iodophenyl)ethyl)aniline (3na)

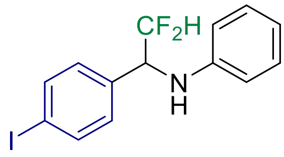

3na

Prepared according to General Procedure A using 4-iodobenzaldehyde (46.4 mg, 0.2 mmol, 1.0 equiv.) and aniline (22.4 mg, 0.24 mmol, 1.2 equiv.) as starting materials. After purification by flash column chromatography (hexane: EtOAc = 100 : 2.5), the title compound was isolated as a pale-brown solid (57.4mg, 79.9 % yield).

^{1}H-NMR (400MHz, $CDCl_3$) δ 7.71 (dt, *J* = 8.7, 2.0 Hz, 2H), 7.19-7.12 (m, 4H), 6.76 (t, *J* = 7.4 Hz, 1H), 6.58 (dd, *J* = 8.5, 1.1 Hz, 2H), 5.98 (td, *J* = 55.7, 2.9 Hz, 1H), 4.66 (td, *J* = 13.2, 2.9 Hz, 1H)

^{13}C-NMR (100MHz, $CDCl_3$) δ 145.7, 138.1, 135.1, 129.8, 129.5, 119.2, 115.4 (t, *J* = 245.4 Hz), 114.1, 94.5, 60.0 (t, *J* = 22.0 Hz)

^{19}F-NMR (376MHz, $CDCl_3$) δ -126.0 (m)

HRMS (FAB) m/z: $[M]^+$ Calcd for $C_{14}H_{12}F_2IN^+$ 358.9983; Found 358.9993.

IR (neat) ν 3411, 3054, 3025, 1604, 1508, 1485, 1315, 1295, 1123, 1076, 1060, 1007, 751, 692 cm^{-1}

m.p. (purified from DCM): 85–87 °C

N-(2,2-difluoro-1-(4-(trifluoromethyl)phenyl)ethyl)aniline (3oa)

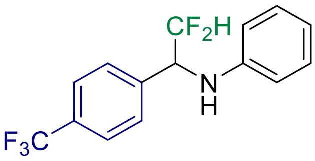

3oa

Prepared according to General Procedure A using 4-trifluoromethylbenzaldehyde (34.8 mg, 0.2 mmol, 1.0 equiv.) and aniline (22.4 mg, 0.24 mmol, 1.2 equiv.) as starting materials. After purification by flash column

chromatography (hexane: EtOAc = 100 : 4.5), the title compound was isolated as a brown oil (28.2mg, 46.8 % yield).

^{1}H-NMR (400MHz, $CDCl_3$) δ 7.65 (d, *J* = 8.2 Hz, 2H), 7.57 (d, *J* = 8.2 Hz, 2H), 7.18-7.12 (m, 2H), 6.78 (d, *J* = 7.8 Hz, 1H), 6.58 (d, *J* = 7.8 Hz, 2H), 6.03 (td, *J* = 55.6, 2.9 Hz, 1H), 4.79 (td, *J* = 13.3, 2.7 Hz, 1H)

^{13}C-NMR (100MHz, $CDCl_3$) δ 145.4, 139.4, 131.0 (q, *J* = 32.6 Hz), 129.6, 128.4, 126.0 (q, *J* = 3.5 Hz), 124.0 (q, *J* = 270.5 Hz), 119.4, 115.3 (t, *J* = 246.6 Hz), 114.1, 60.0 (t, *J* = 22.1 Hz)

^{19}F-NMR (376MHz, $CDCl_3$) δ -62.6 (s, 3F), -126.0 (m, 2F)

HRMS (FAB) m/z: $[M]^+$ Calcd for $C_{15}H_{12}F_5N^+$ 301.0890; Found 301.0895.

IR (neat) ν 3420, 3057, 1605, 1509, 1327, 1168, 1125, 1067, 1018, 752, 692 cm^{-1}

Methyl 4-(2,2-difluoro-1-(phenylamino)ethyl)benzoate (3pa)

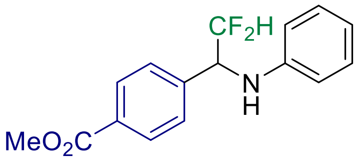

3pa

Prepared according to General Procedure A using methyl 4-formylbenzoate (32.8 mg, 0.2 mmol, 1.0 equiv.) and aniline (22.4 mg, 0.24 mmol, 1.2 equiv.) as starting materials. After purification by flash column chromatography (hexane: EtOAc = 100 : 8), the title compound was isolated as a brown solid (46.1mg, 79.1 % yield).

^{1}H-NMR (400MHz, $CDCl_3$) δ 8.06 (d, *J* = 6.9 Hz, 2H), 7.52 (d, *J* = 8.2 Hz, 2H), 7.16-7.11 (m, 2H), 6.77-6.73 (m, 1H), 6.58 (dd, *J* = 8.7, 0.9 Hz, 2H), 6.02 (td, *J* = 55.6, 2.9 Hz, 1H), 4.78 (td, *J* = 13.3, 2.6 Hz, 1H), 3.91 (s, 3H)

^{13}C-NMR (100MHz, $CDCl_3$) δ 166.8, 145.7, 140.5, 130.6, 130.2, 129.5, 128.0, 119.2, 115.5 (t, *J* = 246.6 Hz), 114.1, 60.2 (t, *J* = 21.6 Hz), 52.3

^{19}F-NMR (376MHz, $CDCl_3$) δ -125.8 (dddd, *J* = 362.3, 280.7, 55.3, 13.4 Hz)

HRMS (FAB) m/z: $[M+H]^+$ Calcd for $C_{16}H_{16}F_2NO_2^+$ 292.1149; Found 292.1140.

IR (neat) ν 3387, 3055, 2954, 2917, 1719, 1604, 1509, 1437, 1315, 1285, 1115, 1077, 1057, 1019, 752, 733, 693 cm^{-1}

m.p. (purified from DCM): 75–77 ℃

4-(2,2-difluoro-1-(phenylamino)ethyl)benzonitrile (3qa)

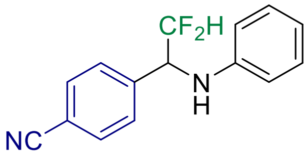

3qa

Prepared according to General Procedure A using 4-trifluoromethylbenzaldehyde (26.2 mg, 0.2 mmol, 1.0 equiv.) and aniline (22.4 mg, 0.24 mmol, 1.2 equiv.) as starting materials. After purification by flash column chromatography (hexane: EtOAc = 100 : 10), the title compound was isolated as a white solid (34.4mg, 66.6 % yield).

^{1}H-NMR (400MHz, $CDCl_3$) δ 7.68 (dt, *J* = 8.4, 1.8 Hz, 2H), 7.56 (d, *J* = 8.2 Hz, 2H), 7.17-7.12 (m, 2H), 6.80-6.75 (m, 1H), 6.55 (dd, *J* = 8.7, 0.9 Hz, 2H), 6.02 (td, *J* = 55.6, 2.7 Hz, 1H), 4.82-4.75 (m, 1H), 4.43 (s, 1H)

^{13}C-NMR (100MHz, $CDCl_3$) δ 145.3, 140.8 (d, *J* = 2 Hz), 132.8, 129.6, 128.9, 119.5, 118.5, 115.1 (t, *J* = 246.5 Hz), 114.0, 112.8, 60.1 (t, *J* = 22.1 Hz)

^{19}F-NMR (376MHz, $CDCl_3$) δ -125.8 (dddd, *J* = 527.0, 282.9, 55.3, 13.4 Hz)

HRMS (FAB) m/z: $[M]^+$ Calcd for $C_{15}H_{12}F_2N_2^+$ 258.0969; Found 258.0966.

IR (neat) ν 3384, 3056, 2230, 1927, 1604, 1508, 1318, 1294, 1125, 1078, 1059, 844, 752, 693 cm^{-1}

m.p. (purified from DCM): 83–85 ℃

N-(2,2-difluoro-1-(4-(methylsulfonyl)phenyl)ethyl)aniline (3ra)

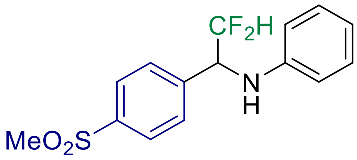

3ra

Prepared according to General Procedure A using 4-(methylsulfonyl)benzaldehyde (36.8 mg, 0.2 mmol, 1.0 equiv.) and aniline (22.4 mg, 0.24 mmol, 1.2 equiv.) as starting materials. After purification by flash column chromatography (hexane: EtOAc = 100 : 30), the title compound was isolated as a brown solid (40.2mg, 64.6 % yield).

^{1}H-NMR (400MHz, $CDCl_3$) δ 7.96 (dt, *J* = 8.7, 1.8 Hz, 2H), 7.66 (d, *J* = 8.2 Hz, 2H), 7.17-7.12 (m, 2H), 6.79-6.75 (m, 1H), 6.58-6.54 (m, 2H), 6.04 (td, *J* = 55.6, 2.7 Hz, 1H), 4.86-4.79 (m, 1H), 3.06 (s, 3H)

^{13}C-NMR (100MHz, $CDCl_3$) δ 145.3, 141.8 (d, *J* = 2 Hz), 141.0, 129.6, 129.1, 128.1, 119.5, 115.1 (t, *J* = 246.5 Hz), 114.1, 60.0 (t, *J* = 22.1 Hz), 44.6

^{19}**F-NMR** (376MHz, $CDCl_3$) δ -125.8 (dddd, *J* = 527.8, 282.9, 55.3, 13.4 Hz)

HRMS (FAB) m/z: $[M]^+$ Calcd for $C_{15}H_{16}F_2NO_2S^+$ 312.0870; Found 312.0865.

IR (neat) ν 3380, 3056, 3027, 2927, 1930, 1604, 1510, 1499, 1410, 1306, 1149, 1059, 959, 770, 754, 739, 693 cm^{-1}

m.p. (purified from DCM): 110–115 ℃

N-(2,2-difluoro-1-(4-(4,4,5,5-tetramethyl-1,3,2-dioxaborolan-2-yl)phenyl)ethyl)aniline (3sa)

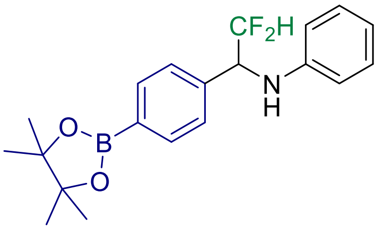

3sa

Prepared according to General Procedure A using 4-formylbenzeneboronic pinacol ester (46.4 mg, 0.2 mmol, 1.0 equiv.) and aniline (22.4 mg, 0.24 mmol, 1.2 equiv.) as starting materials. After purification by preparative thin layer chromatography (hexane: EtOAc = 100 : 15), the title compound was isolated as a pale brown solid (45.0 mg, 62.6 % yield).

***Notes:** The substrate containing a pinacol boronate, separation by flash column chromatography was inefficient due to dragging; therefore, the reaction was repeated and the product was purified by preparative TLC.

^{1}**H-NMR** (400MHz, $CDCl_3$) δ 7.83 (dd, *J* = 6.4, 1.8 Hz, 2H), 7.43 (d, *J* = 8.2 Hz, 2H), 7.15-7.10 (m, 2H), 6.75-6.71 (m, 1H), 6.59 (dt, *J* = 7.3, 1.3 Hz, 2H), 5.99 (td, *J* = 55.8, 2.7 Hz, 1H), 4.72 (td, *J* = 13.3, 2.3 Hz, 1H), 4.40 (s, 1H), 1.34 (s, 12H)

^{13}**C-NMR** (100MHz, $CDCl_3$) δ 146.1, 138.6 (d, *J* = 2 Hz), 135.4, 129.4, 127.2, 118.9, 115.8 (t, *J* = 246.0 Hz), 114.1, 84.0, 60.6 (t, *J* = 22.1 Hz), 25.0 (d, *J* = 4.8 Hz)

^{19}**F-NMR** (376MHz, $CDCl_3$) δ -124.7 (ddd, *J* = 278.9, 56.4, 13.0 Hz, 1F), -127.0 (ddd, *J* = 278.9, 54.9, 13.0 Hz, 1F)

HRMS (FAB) m/z: $[M]^+$ Calcd for $C_{20}H_{24}BF_2NO_2^+$ 359.1868; Found 359.1876.

IR (neat) ν 3412, 3053, 2979, 2931, 1604, 1508, 1399, 1361, 1320, 1144, 1088, 858 cm^{-1}

m.p. (purified from DCM): 135–140 ℃

B. Scope of Amines

N-(2,2-difluoro-1-phenylethyl)-4-methylaniline (3ab)

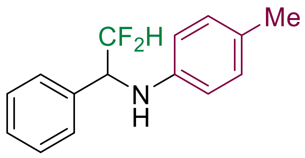

3ab

Prepared according to General Procedure A using benzaldehyde (21.2 mg, 0.2 mmol, 1.0 equiv.) and p-toluidine (25.7 mg, 0.24 mmol, 1.2 equiv.) as starting materials. After purification by flash column chromatography (hexane: EtOAc = 100 : 2), the title compound was isolated as a brown oil (26.6mg, 53.8 % yield for 24 h reaction, 43.5 mg, 88.0 % yield for 48 h reaction).

^{1}H-NMR (400MHz, $CDCl_3$) δ 7.44-7.32 (m, 5H), 6.95 (d, J = 8.3 Hz, 2H), 6.54 (d, J = 8.7 Hz, 2H), 6.00 (td, J = 56.0, 3.1 Hz, 1H), 4.69 (td, J = 13.2, 2.9 Hz, 1H), 2.21 (s, 3H)

^{13}C-NMR (100MHz, $CDCl_3$) δ 143.8, 135.6, 129.9, 129.0, 128.7, 128.2, 127.9, 116.0 (t, J = 244.9 Hz), 114.3, 60.7 (t, J = 21.5 Hz), 20.5

^{19}F-NMR (376MHz, $CDCl_3$) δ -125.9 (dddd, J = 600.7, 279.2, 56.0, 13.4 Hz)

HRMS (FAB) m/z: $[M]^+$ Calcd for $C_{15}H_{15}F_2N^+$ 247.1173; Found 247.1168.

IR (neat) ν 3412, 3032, 2921, 2865, 1618, 1521, 1294, 1127, 1074, 1056, 808, 711, 701 cm^{-1}

N-(2,2-difluoro-1-phenylethyl)-3-methylaniline (3ac)

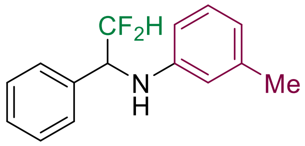

3ac

Prepared according to General Procedure A using benzaldehyde (21.2 mg, 0.2 mmol, 1.0 equiv.) and m-toluidine (25.7 mg, 0.24 mmol, 1.2 equiv.) as starting materials. After purification by flash column chromatography (hexane: EtOAc = 100 : 2), the title compound was isolated as a brown solid (42.1mg, 85.1 % yield).

^{1}H-NMR (400MHz, $CDCl_3$) δ 7.45-7.33 (m, 5H), 7.03 (t, J = 7.8 Hz, 1H), 6.58 (d, J = 7.8 Hz, 1H), 6.48 (s, 1H), 6.42 (dd, J = 7.8, 2.3 Hz, 1H), 6.01 (td, J = 55.8, 3.2 Hz, 1H), 4.72 (td, J = 13.4, 3.0 Hz, 1H), 2.25 (s, 3H)

^{13}C-NMR (100MHz, $CDCl_3$) δ 146.2, 139.3, 135.6 (d, J = 2.8 Hz), 129.3, 129.0, 128.7, 127.9, 119.8, 115.9 (t, J = 246.5 Hz), 115.0, 111.0, 60.3 (t, J = 21.6 Hz), 21.7

^{19}F-NMR (376MHz, $CDCl_3$) δ -125.9 (dddd, J = 721.4, 278.5, 55.3, 13.4 Hz)

HRMS (FAB) m/z: $[M]^+$ Calcd for $C_{15}H_{15}F_2N^+$ 247.1173; Found 247.1177.

IR (neat) ν 3414, 3034, 2977, 2920, 2862, 1608, 1592, 1516, 1492, 1454, 1323, 1305, 1121, 1075, 1056, 771, 711, 701 cm^{-1}

m.p. (purified from DCM): 58–60 ℃

N-(2,2-difluoro-1-phenylethyl)-2-methylaniline (3ad)

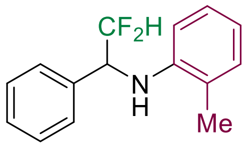

3ad

Prepared according to General Procedure A using benzaldehyde (21.2 mg, 0.2 mmol, 1.0 equiv.) and o-toluidine (25.7 mg, 0.24 mmol, 1.2 equiv.) as starting materials. After purification by flash column chromatography (hexane: EtOAc = 100 : 2), the title compound was isolated as a brown oil (24.2mg, 48.9 % yield).

^{1}H-NMR (400MHz, $CDCl_3$) δ 7.45-7.32 (m, 5H), 7.09 (d, *J* = 7.4 Hz, 1H), 7.00 (t, *J* = 7.8 Hz, 1H), 6.70 (td, *J* = 7.4, 0.9 Hz, 1H), 6.44 (d, *J* = 8.3 Hz, 1H), 6.04 (td, *J* = 55.8, 2.9 Hz, 1H), 4.77 (td, *J* = 13.2, 2.9 Hz, 1H), 2.28 (s, 3H)

^{13}C-NMR (100MHz, $CDCl_3$) δ 144.0, 135.5, 130.5, 129.0, 128.7, 127.8, 127.2, 123.1, 118.6, 116.0 (t, *J* = 244.9 Hz), 111.6, 60.4 (t, *J* = 21.5 Hz), 17.6

^{19}F-NMR (376MHz, $CDCl_3$) δ -126.0 (dddd, *J* = 790.0, 279.2, 55.3, 11.2 Hz)

HRMS (FAB) m/z: $[M]^+$ Calcd for $C_{15}H_{15}F_2N^+$ 247.1173; Found 247.1168.

IR (neat) ν 3439, 3062, 3034, 2976, 2923, 2857, 1606, 1589, 1514, 1454, 1380, 1309, 1255, 1128, 1075, 1051, 796, 710 cm^{-1}

4-(tert-butyl)-N-(2,2-difluoro-1-phenylethyl)aniline (3ae)

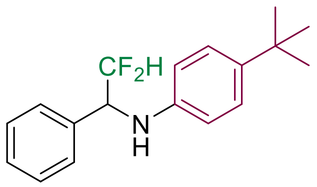

3ae

Prepared according to General Procedure A using benzaldehyde (21.2 mg, 0.2 mmol, 1.0 equiv.) and 4-tert-butylaniline (35.8 mg, 0.24 mmol, 1.2 equiv.) as starting materials. After purification by flash column

chromatography (hexane: EtOAc = 100 : 2.5), the title compound was isolated as a brown oil (33.3mg, 57.5 % yield).

^{1}H-NMR (400MHz, $CDCl_3$) δ 7.45-7.32 (m, 5H), 7.17 (dt, *J* = 9.3, 2.5 Hz, 2H), 6.57 (td, *J* = 5.7, 3.7 Hz, 2H), 5.98 (td, *J* = 55.8, 3.2 Hz, 1H), 4.68 (td, *J* = 13.2, 3.1 Hz, 1H), 1.25 (s, 9H)

^{13}C-NMR (100MHz, $CDCl_3$) δ 143.8, 141.6, 135.9 (d, *J* = 1.9 Hz), 129.0, 128.7, 127.9, 126.2, 116.0 (t, *J* = 244.9 Hz), 113.7, 60.7 (t, *J* = 22 Hz), 34.0, 31.6

^{19}F-NMR (376MHz, $CDCl_3$) δ -125.8 (dddd, *J* = 660.0, 278.5, 55.3, 13.4 Hz)

HRMS (FAB) m/z: $[M]^+$ Calcd for $C_{18}H_{21}F_2N^+$ 289.1642; Found 289.1640.

IR (neat) ν 3415, 3063, 3031, 2962, 2903, 2867, 1615, 1520, 1455, 1302, 1267, 1128, 1074, 1056, 822, 700 cm^{-1}

N-(2,2-difluoro-1-phenylethyl)-4-fluoroaniline (3af)

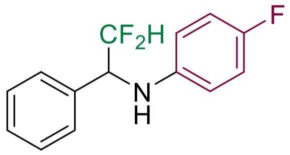

3af

Prepared according to General Procedure A using benzaldehyde (21.2 mg, 0.2 mmol, 1.0 equiv.) and 4-fluoroaniline (28.6 mg, 0.24 mmol, 1.2 equiv.) as starting materials. After purification by flash column chromatography (hexane: EtOAc = 100 : 3), the title compound was isolated as a brown oil (41.8mg, 83.2 % yield).

^{1}H-NMR (400MHz, $CDCl_3$) δ 7.42-7.32 (m, 5H), 6.87-6.81 (m, 2H), 6.57-6.52 (m, 2H), 5.99 (td, *J* = 55.7, 2.9 Hz, 1H), 4.63 (td, *J* = 13.0, 3.2 Hz, 1H)

^{13}C-NMR (100MHz, $CDCl_3$) δ 156.6 (d, *J* = 246 Hz), 142.3, 135.3 (d, *J* = 2.9 Hz), 129.1, 128.8, 127.9, 115.9 (t, *J* =246.6 Hz), 115.9 (d, *J* = 23.1 Hz), 115.2 (d, *J* = 7.7 Hz), 61.1 (t, *J* = 21.6 Hz)

^{19}F-NMR (376MHz, $CDCl_3$) δ -125.9 (dddd, *J* = 902.0, 280.5, 54.9, 14.3 Hz, 2F), -126.9 (s, 1F)

HRMS (FAB) m/z: $[M]^+$ Calcd for $C_{14}H_{12}F_3N^+$ 251.0922; Found 251.0917.

IR (neat) ν 3421, 3063, 3034, 2980, 1614, 1510, 1454, 1378, 1306, 1292, 1122, 1075, 1055, 822, 802, 775, 757, 712, 702 cm^{-1}

4-chloro-N-(2,2-difluoro-1-phenylethyl)aniline (3ag)

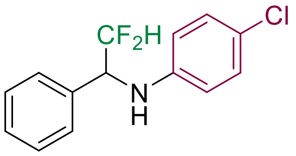

3ag

Prepared according to General Procedure A using benzaldehyde (21.2 mg, 0.2 mmol, 1.0 equiv.) and 4-chloroaniline (30.6 mg, 0.24 mmol, 1.2 equiv.) as starting materials. After purification by flash column chromatography (hexane: EtOAc = 100 : 3), the title compound was isolated as a brown solid (44.8mg, 83.7 % yield).

^{1}H-NMR (400MHz, $CDCl_3$) δ 7.40-7.33 (m, 5H), 7.08 (dt, J = 10.0, 2.6 Hz, 2H), 6.55-6.51 (m, 2H), 5.99 (td, J = 55.7, 3.1 Hz, 1H), 4.66 (td, J = 13.1, 2.9 Hz, 1H)

^{13}C-NMR (100MHz, $CDCl_3$) δ 144.7, 135.1 (d, J = 1.9 Hz), 129.3, 129.1, 128.9, 127.8, 123.6, 115.8 (t, J = 245.4 Hz), 115.2, 60.5 (t, J = 21.5 Hz)

^{19}F-NMR (376MHz, $CDCl_3$) δ -126.0 (dddd, J = 1156.3, 279.2, 56.0, 12.6 Hz)

HRMS (FAB) m/z: $[M]^+$ Calcd for $C_{14}H_{12}ClF_2N^+$ 267.0626; Found 267.0618.

IR (neat) ν 3419, 3064, 3033, 2979, 2927, 1600, 1503, 1454, 1378, 1315, 1293, 1126, 1075, 1056, 816, 732, 716, 700, 672 cm^{-1}

m.p. (purified from DCM): 58–60 ℃

4-bromo-N-(2,2-difluoro-1-phenylethyl)aniline (3ah)

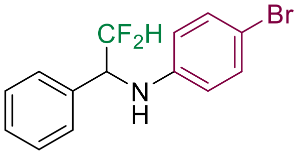

3ah

Prepared according to General Procedure A using benzaldehyde (21.2 mg, 0.2 mmol, 1.0 equiv.) and 4-bromoaniline (41.3 mg, 0.24 mmol, 1.2 equiv.) as starting materials. After purification by flash column chromatography (hexane: EtOAc = 100 : 3), the title compound was isolated as a pale-brown solid (48.9mg, 78.3 % yield).

^{1}H-NMR (400MHz, $CDCl_3$) δ 7.40-7.33 (m, 5H), 7.21 (td, J = 6.2, 3.7 Hz, 2H), 6.48 (dt, J = 9.5, 2.6 Hz, 2H), 5.99 (td, J = 55.6, 2.8 Hz, 1H), 4.66 (td, J = 13.1, 2.9 Hz, 1H), 4.43 (s, 1H)

^{13}**C-NMR** (100MHz, $CDCl_3$) δ 145.1, 135.0 (d, J = 1.9 Hz), 132.2, 129.1, 128.9, 127.8, 115.8 (t, J = 245.4 Hz), 115.7, 110.7, 60.4 (t, J = 21.0 Hz)

^{19}**F-NMR** (376MHz, $CDCl_3$) δ -126.0 (dddd, J = 1199.0, 279.2, 55.3, 13.4 Hz)

HRMS (FAB) m/z: $[M]^+$ Calcd for $C_{14}H_{12}BrF_2N^+$ 311.0121; Found 311.0127.

IR (neat) ν 3417, 3064, 3032, 2979, 1594, 1498, 1454, 1378, 1315, 1293, 1246, 1143, 1125, 1074, 1055, 1002, 814, 724, 716, 700 cm^{-1}

m.p. (purified from DCM): 75–77 ℃

3-bromo-N-(2,2-difluoro-1-phenylethyl)aniline (3ai)

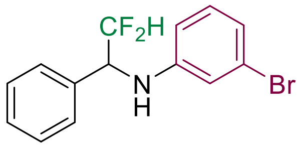

3ai

Prepared according to General Procedure A using benzaldehyde (21.2 mg, 0.2 mmol, 1.0 equiv.) and 3-bromoaniline (41.3 mg, 0.24 mmol, 1.2 equiv.) as starting materials. After purification by flash column chromatography (hexane: EtOAc = 100 : 3), the title compound was isolated as a brown oil (35.2mg, 56.4%).

^{1}**H-NMR** (400MHz, $CDCl_3$) 7.41-7.34 (m, 5H), 6.98 (t, J = 8.0 Hz, 1H), 6.85 (dq, J = 7.8, 0.9 Hz, 1H), 6.77 (d, J = 4.1 Hz, 1H), 6.51 (dq, J = 8.2, 1.1 Hz, 1H), 5.99 (td, J = 55.6, 2.7 Hz, 1H), 4.68 (td, J = 13.3, 2.7 Hz, 1H)

^{13}**C-NMR** (100MHz, $CDCl_3$) δ 147.5, 134.9 (d, J = 2.9 Hz), 130.7, 129.2, 128.9, 127.7, 123.3, 121.7, 116.8, 115.7 (t, J = 246.6 Hz), 112.6, 60.2 (t, J = 21.6 Hz)

^{19}**F-NMR** (376MHz, $CDCl_3$) δ -126.0 (dddd, J = 1298.3, 279.6, 55.6, 13.0 Hz)

HRMS (FAB) m/z: $[M]^+$ Calcd for $C_{14}H_{12}BrF_2N^+$ 311.0121; Found 311.0126.

IR (neat) ν 3417, 3064, 3032, 2977, 1596, 1504, 1481, 1378, 1320, 1283, 1121, 1071, 1056, 988, 842, 765, 700, 681 cm^{-1}

N-(2,2-difluoro-1-phenylethyl)-4-iodoaniline (3aj)

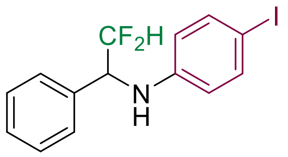

3aj

Prepared according to General Procedure A using benzaldehyde (21.2 mg, 0.2 mmol, 1.0 equiv.) and 4-iodoaniline (52.6 mg, 0.24 mmol, 1.2 equiv.) as starting materials. After purification by flash column chromatography (hexane: EtOAc = 100 : 3), the title compound was isolated as a pale-brown solid (25.4mg, 35.3 % yield).

^{1}H-NMR (400MHz, $CDCl_3$) δ 7.39-7.32 (m, 7H), 6.39 (dt, *J* = 9.3, 2.5 Hz, 2H), 6.13-5.84 (m, 1H), 4.66 (td, *J* = 13.1, 2.9 Hz, 1H)

^{13}C-NMR (100MHz, $CDCl_3$) δ 145.7, 138.0, 134.9 (d, *J* = 2.9 Hz), 129.2, 128.9, 127.8, 116.3, 115.7 (t, *J* = 245.4 Hz), 80.0, 60.3 (t, *J* = 21.5 Hz)

^{19}F-NMR (376MHz, $CDCl_3$) δ -126.0 (dddd, *J* = 1232.2, 279.2, 55.3, 13.4 Hz)

HRMS (FAB) m/z: $[M]^+$ Calcd for $C_{14}H_{12}F_2IN^+$ 358.9983; Found 358.9996.

IR (neat) ν 3414, 3062, 3031, 2977, 1591, 1497, 1454, 1316, 1293, 1125, 1075, 1059, 811, 716, 700 cm^{-1}

m.p. (purified from DCM): 80–82 ℃

N-(2,2-difluoro-1-phenylethyl)-4-(trifluoromethyl)aniline (3ak)

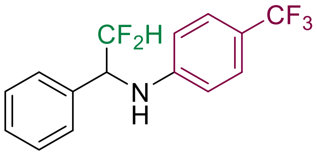

3ak

Prepared according to General Procedure A using benzaldehyde (21.2 mg, 0.2 mmol, 1.0 equiv.) and 4-(trifluoromethyl)aniline (38.7 mg, 0.24 mmol, 1.2 equiv.) as starting materials. After purification by flash column chromatography (hexane: EtOAc = 100 : 5), the title compound was isolated as a brown oil (34.3mg, 56.9 % yield).

^{1}H-NMR (400MHz, $CDCl_3$) 1H-NMR (400 MHz, CHLOROFORM-D) δ 7.41-7.34 (m, 7H), 6.62 (d, *J* = 8.7 Hz, 2H), 6.01 (td, *J* = 55.5, 2.5 Hz, 1H), 4.79-4.72 (m, 2H)

^{13}C-NMR (100MHz, $CDCl_3$) δ 148.7, 134.7 (d, *J* = 2.9 Hz), 129.2, 129.1, 127.7, 126.8 (q, *J* = 3.8 Hz), 124. 8 (q, *J* = 249.6 Hz), 120.5 (q, *J* = 32.5 Hz), 115.7 (t, *J* = 245.4 Hz), 113.2, 60.0 (t, *J* = 21.0 Hz)

^{19}F-NMR (376MHz, $CDCl_3$) δ -61.2 (s, 3F), -126.1 (dddd, *J* = 1655.2, 278.2, 55.6, 13.0 Hz, 2F)

HRMS (FAB) m/z: $[M]^+$ Calcd for $C_{15}H_{12}F_5N^+$ 301.0890; Found 301.0887.

IR (neat) ν 3437, 3066, 3035, 2982, 1619, 1532, 1496, 1455, 1330, 1273, 1110, 1066, 826, 714, 700 cm^{-1}

Methyl 4-((2,2-difluoro-1-phenylethyl)amino)benzoate (3al)

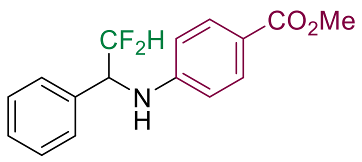

3al

Prepared according to General Procedure A using benzaldehyde (21.2 mg, 0.2 mmol, 1.0 equiv.) and methyl 4-aminobenzoate (36.3 mg, 0.24 mmol, 1.2 equiv.) as starting materials. After purification by flash column chromatography (hexane: EtOAc = 100 : 30), the title compound was isolated as a yellow solid (34.0mg, 58.4 % yield).

^{1}H-NMR (400MHz, $CDCl_3$) δ 7.82 (dt, *J* = 9.2, 2.2 Hz, 2H), 7.40-7.33 (m, 5H), 6.58 (dt, *J* = 9.3, 2.3 Hz, 2H), 6.02 (td, *J* = 55.4, 2.8 Hz, 1H), 4.83-4.76 (m, 1H), 3.83 (s, 3H)

^{13}C-NMR (100MHz, $CDCl_3$) δ 167.2, 150.0, 134.7 (d, *J* = 2.9 Hz), 131.6, 129.2, 129.0, 127.7, 120.2, 115.6 (t, *J* = 245.4 Hz), 112.9, 59.8 (t, J = 21.5 Hz), 51.8

^{19}F-NMR (376MHz, $CDCl_3$) δ -126.1 (dddd, *J* = 1631.7, 280.0, 55.3, 13.4 Hz)

HRMS (FAB) m/z: $[M]^+$ Calcd for $C_{16}H_{15}F_2NO_2^+$ 291.1071; Found 291.1076.

IR (neat) ν 3362, 3064, 3033, 2952, 1701, 1607, 1526, 1436, 1313, 1283, 1178, 1114, 1076, 840, 771, 700 cm^{-1}

m.p. (purified from DCM): 79–81 ℃

4-((2,2-difluoro-1-phenylethyl)amino)benzonitrile (3am)

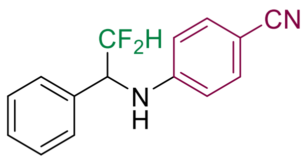

3am

Prepared according to General Procedure A using benzaldehyde (21.2 mg, 0.2 mmol, 1.0 equiv.) and 4-aminobenzonitrile (28.4 mg, 0.24 mmol, 1.2 equiv.) as starting materials. After purification by flash column chromatography (hexane: EtOAc = 100 : 35), the title compound was isolated as a white solid (15.8mg, 30.6 % yield for 24 h reaction, 22.1 mg, 42.8 % yield for 48 h reaction).

^{1}H-NMR (400MHz, $CDCl_3$) δ 7.44-7.33 (m, 7H), 6.59 (dt, *J* = 9.3, 2.3 Hz, 2H), 6.01 (td, *J* = 55.3, 2.7 Hz, 1H), 4.92 (s, 1H), 4.79-4.72 (m, 1H)

^{13}C-NMR (100MHz, $CDCl_3$) δ 149.5, 134.3 (d, *J* = 3.9 Hz), 133.9, 129.4, 129.2, 127.6, 120.0, 115.5 (t, *J* = 247.0 Hz), 113.6, 100.9, 59.8 (t, *J* = 21.1 Hz)

^{19}F-NMR (376MHz, $CDCl_3$) δ -126.1 (dddd, J = 2103.5, 279.2, 55.3, 13.4 Hz)

HRMS (FAB) m/z: $[M]^+$ Calcd for $C_{15}H_{12}F_2N_2^+$ 258.0969; Found 258.0962.

IR (neat) ν 3353, 3034, 2216, 1608, 1524, 1336, 1176, 1076, 1057, 825, 701 cm^{-1}

m.p. (purified from DCM): 88–90 ℃

N-(2,2-difluoro-1-phenylethyl)-4-(methylsulfonyl)aniline (3an)

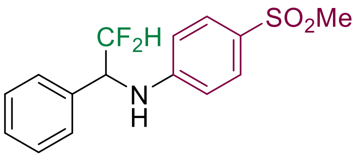

3an

Prepared according to General Procedure A using benzaldehyde (21.2 mg, 0.2 mmol, 1.0 equiv.) and 4-(methylsulfonyl)aniline (41.1 mg, 0.24 mmol, 1.2 equiv.) as starting materials. After purification by flash column chromatography (hexane: EtOAc = 100 : 35), the title compound was isolated as a yellow oil (17.6mg, 28.3 % yield).

^{1}H-NMR (400MHz, $CDCl_3$) δ 7.69-7.64 (m, 2H), 7.44-7.35 (m, 5H), 6.62-6.70 (2H), 6.03 (td, J = 55.3, 2.7 Hz, 1H), 4.99 (s, 1H), 4.82-4.75 (m, 1H), 2.97 (s, 3H)

^{13}C-NMR (100MHz, $CDCl_3$) δ 150.5, 134.2 (d, J = 4.8 Hz), 129.5, 129.4, 129.4, 129.2, 127.6, 115.5 (t, J = 246.5 Hz), 113.3, 59.9 (t, J = 21.6 Hz), 45.1 (t, J = 3.9 Hz)

^{19}F-NMR (376MHz, $CDCl_3$) δ -126.1 (dddd, J = 2101.7, 279.6, 55.6, 13.0 Hz)

HRMS (FAB) m/z: $[M+H]^+$ Calcd for $C_{15}H_{16}F_2NO_2S^+$ 312.0870; Found 312.0878.

IR (neat) ν 3364, 2927, 1597, 1522, 1337, 1294, 1141, 958, 827, 770, 701 cm^{-1}

N-(2,2-difluoro-1-phenylethyl)-4-(4,4,5,5-tetramethyl-1,3,2-dioxaborolan-2-yl)aniline (3ao)

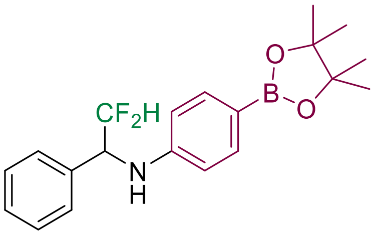

3ao

Prepared according to General Procedure A using benzaldehyde (21.2 mg, 0.2 mmol, 1.0 equiv.) and 4-(4,4,5,5,-tetramethyl-1,3,2-dioxaborolane) (52.6 mg, 0.24 mmol, 1.2 equiv.) as starting materials. After purification by

preparative thin layer chromatography (hexane: EtOAc = 100 : 20), the title compound was isolated as a pale-brown solid (53.5mg, 74.5 % yield).

***Notes:** The substrate containing a pinacol boronate, separation by flash column chromatography was inefficient due to dragging; therefore, the reaction was repeated and the product was purified by preparative TLC.

^{1}H-NMR (400MHz, $CDCl_3$) δ 7.61 (dd, *J* = 8.5, 2.1 Hz, 2H), 7.42-7.31 (m, 5H), 6.60 (d, *J* = 8.7 Hz, 2H), 6.02 (td, *J* = 55.8, 2.9 Hz, 1H), 4.79 (td, *J* = 13.3, 2.8 Hz, 1H), 1.31 (s, 12H)

^{13}C-NMR (100MHz, $CDCl_3$) δ 148.6, 136.4, 135.1 (d, *J* = 2.9 Hz), 129.0, 128.8, 127.8, 115.7 (t, *J* = 244.9 Hz), 113.1, 83.5, 59.8 (t, *J* = 21.5 Hz), 29.8, 24.9 (d, *J* = 4.8 Hz)

^{19}F-NMR (376MHz, $CDCl_3$) δ -126.0 (dddd, *J* = 989.8, 279.6, 55.6, 13.7 Hz)

HRMS (FAB) m/z: $[M]^+$ Calcd for $C_{20}H_{24}BF_2NO_2^+$ 359.1868; Found 359.1880.

IR (neat) ν 3408, 2979, 2929, 1607, 1493, 1484, 1455, 1397, 1361, 1321, 1284, 1273, 1144, 1089, 962, 859, 824, 716, 700 cm^{-1}

m.p. (purified from DCM): 125–127 ℃

C. Late-Stage Functionalization

4-((2,2-difluoro-1-phenylethyl)amino)-N-(thiazol-2-yl)benzenesulfonamide (3ap)

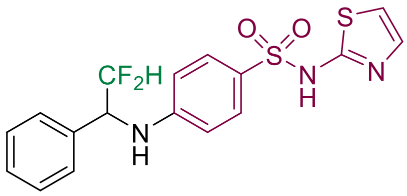

3ap

Prepared according to General Procedure A using benzaldehyde (21.2 mg, 0.2 mmol, 1.0 equiv.) and 4-amino-N-(thiazol-2-yl)benzenesulfonamide (Sulfathiazole, 61.3 mg, 0.24 mmol, 1.2 equiv.) as starting materials. After purification by flash column chromatography (DCM: MeOH = 100 : 2), the title compound was isolated as a pale-yellow solid (37.0 mg, 46.7 % yield).

^{1}H-NMR (400MHz, DMSO-d6) δ 7.47 (dd, J = 13.1, 7.6 Hz, 4H), 7.39-7.35 (m, 2H), 7.32-7.29 (m, 1H), 7.22 (d, J = 9.1 Hz, 1H), 7.17 (d, J = 4.9 Hz, 1H), 6.77-6.72 (m, 3H), 6.27 (td, J = 55.3, 3.5 Hz, 1H), 5.10-5.00 (m, 1H)

^{13}C-NMR (100MHz, DMSO-d6) δ 168.2, 150.2, 135.7, 129.5, 128.5, 128.2, 127.4, 124.4, 115.8 (t, J = 243.4 Hz), 112.1, 107.6, 57.6 (t, J = 22.0 Hz), 48.6

^{19}F-NMR (376MHz, DMSO-d6) δ -124.1 (dddd, J = 406.0, 276.7, 55.6, 13.7 Hz)

HRMS (FAB) m/z: $[M+H]^+$ Calcd for $C_{17}H_{16}F_2N_3O_2S_2^+$ 396.0652; Found 396.0647.

IR (neat) ν 3353, 3102, 2970, 1740, 1598, 1535, 1417, 1328, 1288, 1141, 1090, 929, 702 cm^{-1}

m.p. (purified from DCM): 150–152 ℃

3-(4-((2,2-difluoro-1-phenylethyl)amino)phenyl)-3-ethylpiperidine-2,6-dione (3aq)

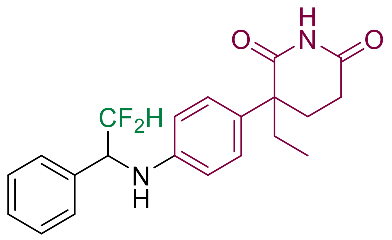

3aq

Prepared according to General Procedure A using benzaldehyde (21.2 mg, 0.2 mmol, 1.0 equiv.) and 3-(4-aminophenyl)-3-ethylpiperidine-2,6-dione (Aminoglutethimide, 55.7 mg, 0.24 mmol, 1.2 equiv.) as starting materials. After purification by preparative flash column chromatography (DCM: MeOH = 100 : 1.5), the title compound was isolated as a yellow oil (64.4mg, 86.3 % yield).

^{1}H-NMR (400 MHz, $CDCl_3$) δ 8.03-7.95 (m, 1H), 7.43-7.32 (m, 5H), 7.02 (dd, J = 11.2, 3.0 Hz, 2H), 6.58 (dt, J = 9.5, 2.6 Hz, 2H), 5.98 (td, J = 55.7, 2.9 Hz, 1H), 4.68 (td, J = 13.0, 2.6 Hz, 1H), 4.47 (s, 1H), 2.58-2.50 (m, 1H), 2.45-2.34 (m, 1H), 2.30-2.23 (m, 1H), 2.18-2.09 (m, 1H), 1.97 (td, J = 14.5, 7.2 Hz, 1H), 1.83 (td, J = 14.4, 7.3 Hz, 1H), 0.82 (td, J = 7.3, 1.4 Hz, 3H)

^{13}C-NMR (100 MHz, $CDCl_3$) δ 175.5 (d, J = 1.9 Hz), 172.6 (d, J = 1.9 Hz), 145.5, 135.3 (d, J = 1.9 Hz), 129.1, 128.9, 128.4, 127.8, 127.3, 115.8 (t, J = 246.1 Hz), 114.3, 60.4 (t, J = 21.6 Hz), 50.4 (d, J = 2.0 Hz), 33.0 (d, J = 2.9 Hz), 29.4, 27.0, 9.1

^{19}F-NMR (376 MHz, $CDCl_3$) δ -125.9 (dddd, J = 1118.4, 278.9, 56.4, 13.0 Hz)

HRMS (FAB) m/z: $[M+H]^+$ Calcd for $C_{21}H_{23}F_2N_2O_2^+$ 373.1728; Found 373.1726.

IR (neat) ν 3376, 3221, 3092, 3063, 2972, 2881, 1696, 1613, 1520, 1455, 1351, 1267, 1191, 1129, 1116, 1073, 1056, 824, 736, 702 cm^{-1}

D. Low Yielding or unsuccessful Substrates

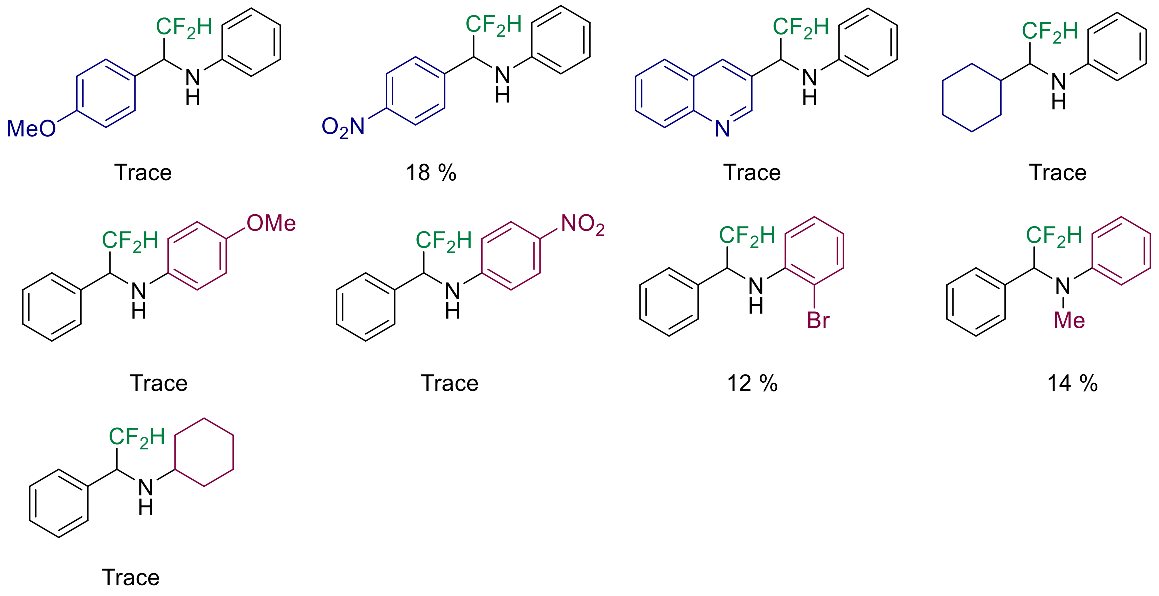

Figure 2. List of unsuccessful substrates

***Notes:** Our reaction conditions were incompatible with a strongly electron-donating p-methoxy group. For nitro-substituted substrates, the yield was low for the corresponding aldehydes, and no conversion was observed for nitro-substituted anilines. In the latter case, imine formation itself was not readily detected, presumably because the nitro substituent markedly reduces the nucleophilicity of the amine. The reaction did not proceed with aliphatic substrates, likely due to the instability of the corresponding imines under the reaction conditions.

E. Scale-up Reaction

General Procedure B for large scale synthesis

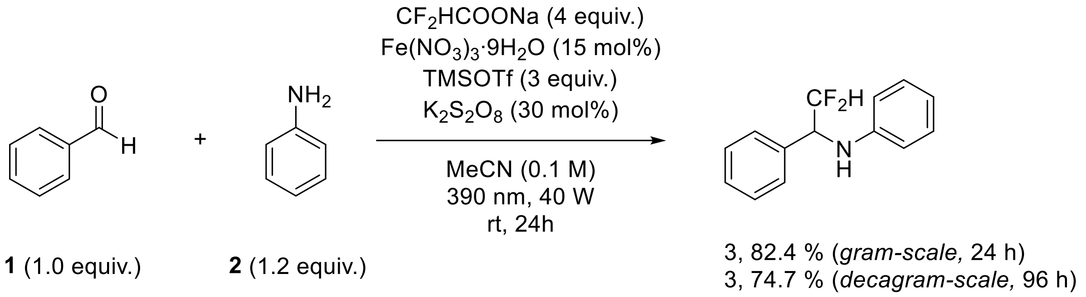

For gram-scale reaction: The benzaldehyde, aniline, and MeCN were degassed with an Ar balloon for at least 20 min prior to use. In an oven-dried 100 mL pear-shaped flask equipped with a Teflon®-coated magnetic stir bar, $CF_2HCOONa$ (4.72 g, 4.0 equiv.), $K_2S_2O_8$ (811.0 mg, 0.30 equiv.) and $Fe(NO_3)_3{\cdot}9H_2O$ (606.0 mg, 15 mol%) were added. The flask was evacuated and backfilled with Ar gas (repeated 3 times), followed by addition of benzaldehyde (**1**, 10 mmol, 1.0 equiv.), aniline (**2**, 12 mmol, 1.2 equiv.) and MeCN (100 mL, 0.10 M with respect to the aldehyde). The mixture was stirred (700 rpm) for 10 min to dissolve the iron complex, followed by dropwise

addition of TMSOTf (5.43 mL, 3.0 equiv.). The reaction mixture was irradiated with two 40 W, 390 nm Kessil LED lamps and stirred at 700rpm for 24 h under fan cooling. After completion, the reaction mixture was concentrated under reduced pressure to remove acetonitrile, then diluted with DCM, washed with saturated aqueous Na_2CO_3, and the layers were separated. The aqueous layer was extracted with DCM twice. The combined organic layers were dried with anhydrous MgSO4, filtered, and concentrated under reduced pressure. Purification by flash column chromatography afforded the corresponding α-difluoromethylated amines (1.92 g, 82.4 % yield).

For decagram-scale reaction: The benzaldehyde, aniline, and MeCN were degassed with an Ar balloon for at least 20 min prior to use. In an oven-dried 1 L pear-shaped flask equipped with a Teflon®-coated magnetic stir bar, $CF_2HCOONa$ (47.2 g, 4.0 equiv.), $K_2S_2O_8$ (8.11 g, 0.30 equiv.) and $Fe(NO_3)_3{\cdot}9H_2O$ (6.06 g, 15 mol%) were added. The flask was evacuated and backfilled with Ar gas (repeated 3 times), followed by addition of benzaldehyde (**1**, 0.1 mol, 1.0 equiv.), aniline (**2**, 0.12 mol, 1.2 equiv.) and MeCN (1.0 L, 0.10 M with respect to the aldehyde). The mixture was stirred (900 rpm) for 10 min to dissolve the iron complex, followed by dropwise addition of TMSOTf (54.3 mL, 3.0 equiv.). The reaction mixture was irradiated with two 40 W, 390 nm Kessil LED lamps and stirred at 900rpm for 96 h under fan cooling. After completion, the reaction mixture was concentrated under reduced pressure to remove acetonitrile, then diluted with DCM, washed with saturated aqueous Na_2CO_3, and the layers were separated. The aqueous layer was extracted with DCM twice. The combined organic layers were dried with anhydrous MgSO4, filtered, and concentrated under reduced pressure. Purification by flash column chromatography afforded the corresponding α-difluoromethylated amines (17.4 g, 74.7 % yield).

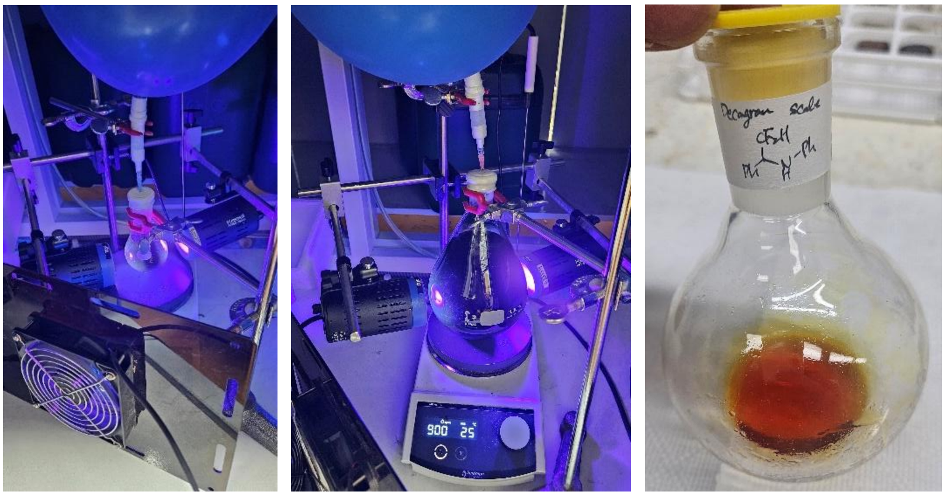

Figure 3. Photograph of *gram*-scale(left), *decagram*-scale(middle) reaction, and isolated product (right) of *decagram-scale* reaction.

***Notes:** Although solvent volume increases upon scale-up, increasing the reaction concentration should be avoided. As demonstrated in the optimization studies (Section 2.F.), higher concentrations result in a sharp decrease in reaction yield. In addition, as the reaction scale increased, the photon flux per unit volume (and per molecule) decreased under otherwise identical irradiation conditions; therefore, the reaction progress was monitored by taking aliquots and analyzing the crude mixture by ^1H NMR.

4. Mechanistic Investigation

A. Radical Trapping Experiment

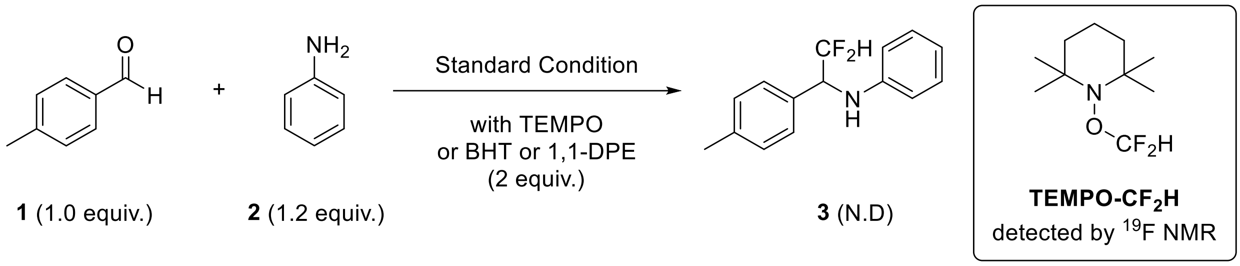

1 (1.0 equiv.) **2** (1.2 equiv.) **3** (N.D)

The p-tolualdehyde, aniline, and MeCN were degassed with an Ar balloon for at least 20 min prior to use. In an oven-dried 4 mL vial equipped with a Teflon®-coated magnetic stir bar, $CF_2HCOONa$ (94.4 mg, 4.0 equiv.), $K_2S_2O_8$ (16.2 mg, 0.30 equiv.), $Fe(NO_3)_3{\cdot}9H_2O$ (12.1 mg, 15 mol%) and a radical scavenger (0.4 mmol, 2.0 equiv.; TEMPO, BHT or 1,1-diphenylethylene) were added. The vial was evacuated and backfilled with Ar gas (repeated 3 times), followed by addition of p-tolualdehyde (**1**, 0.2 mmol, 1.0 equiv.), aniline (**2**, 0.24 mmol, 1.2 equiv.) and MeCN (2 mL, 0.10 M with respect to the aldehyde). The mixture was stirred (700 rpm) for 3 min to dissolve the iron complex, followed by addition of TMSOTf (0.11 mL, 3.0 equiv.). The rubber septum was quickly replaced with a sealing cap, which was tightened securely to maintain an Ar atmosphere, and the vial was then sealed with Parafilm. The reaction mixture was placed in a photoreactor equipped with a 40 W, 390 nm Kessil lamp and stirred at 700 rpm for 24 h under ethanol coolant circulation to maintain room temperature. After completion, the reaction mixture was diluted with DCM, washed with saturated aqueous Na_2CO_3, and the layers were separated. The aqueous layer was extracted with DCM twice. The combined organic layers were dried with anhydrous MgSO4, filtered, and concentrated under reduced pressure. Dibromomethane (0.10 mmol, 7 μL) was added to the crude mixture as an internal standard for NMR analysis. The yields were determined by relative integration of dibromomethane (δ 4.93 ppm, s, 2H, normalized to 2.0) and product **3** (δ 4.73 ppm, td, 1H).

For the experiment in which TEMPO was added as a radical scavenger, ^{19}F NMR analysis was performed using $PhCF_3$ (14 μL, 0.10 mmol) as an internal standard to probe for the TEMPO–radical adduct. A signal at δ −79.85 ppm (d, 72.2 Hz) was observed, consistent with the previously reported TEMPO–CF_2H adduct.[2]

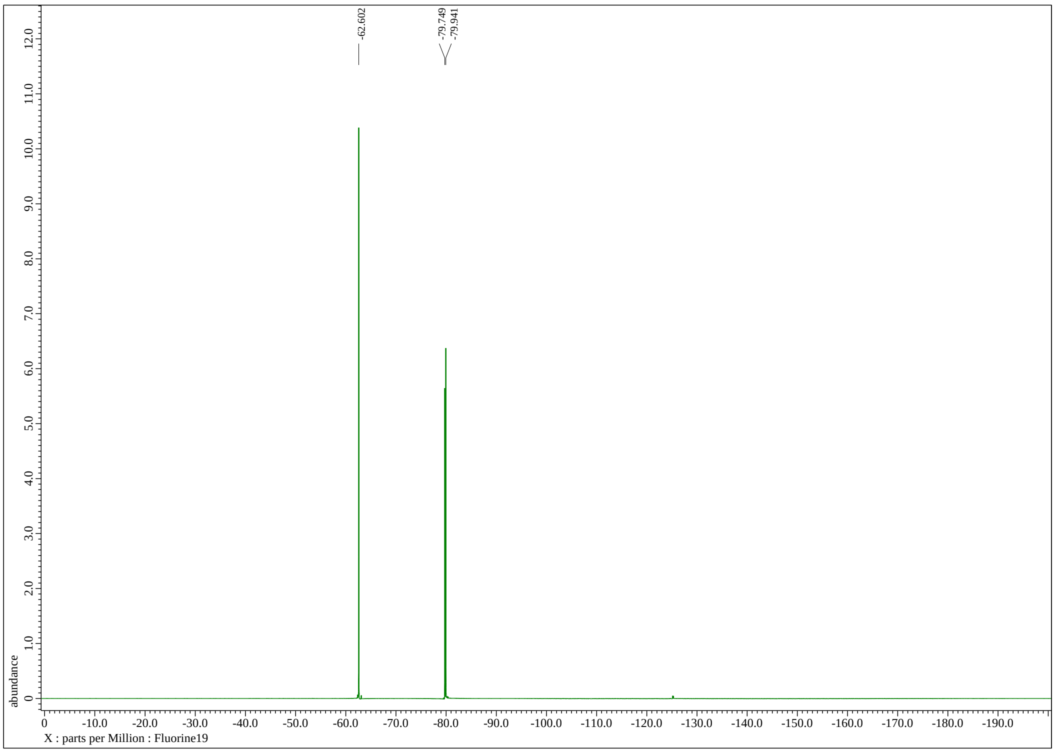

Figure 4. ^{19}F NMR spectroscopy of TEMPO-CF_2H adduct with $PhCF_3$ as NMR reference

B. Reaction Intermediate Experiment

Synthesis of Imine intermediate

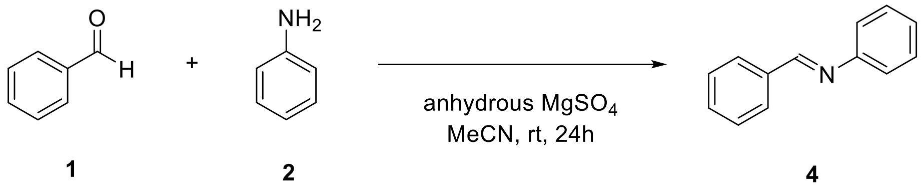

In an oven-dried 25 mL vial equipped with a Teflon®-coated magnetic stir bar, anhydrous $MgSO_4$ (240.7 mg, 2.0 equiv.) was added. The vial was evacuated and backfilled with Ar gas (repeated 3 times), followed by addition of benzaldehyde (**1**, 1.1 mmol), aniline (**2**, 1.0 mmol) and MeCN (5 mL, 0.20 M with respect to the aniline). The reaction mixture was stirred at 700 rpm for 24 h. After completion, the reaction mixture was concentrated under reduced pressure. Purification by flash column chromatography (Et_3N/hexane = 5:95) afforded the corresponding Imine intermediate **4** (N-benzylideneaniline) as a pale-yellow solid (170.5 mg, 94.1 % yield).

N-benzylideneaniline (4)

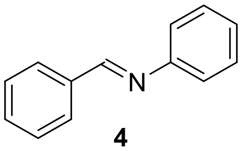

4

^{1}H-NMR (400MHz, $CDCl_3$) δ 8.47 (s, 1H), 7.94-7.90 (m, 2H), 7.51-7.46 (m, 3H), 7.43-7.39 (m, 2H), 7.27-7.21 (m, 3H)

^{13}C-NMR (100MHz, $CDCl_3$) δ 160.5, 152.2, 136.4, 131.5, 129.3, 128.9, 128.9, 126.1, 121.0

HRMS (FAB) m/z: $[M+H]^+$ Calcd for $C_{13}H_{12}N^+$ 182.0970; Found 182.0977.

IR (neat) ν 3061, 3029, 1627, 1591, 1579, 1485, 1451, 1191, 1169, 767, 693 cm^{-1}

Reaction starting from Imine intermediate (4)

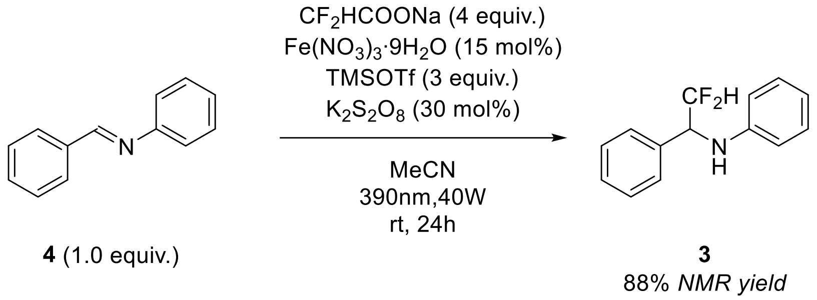

The MeCN was degassed with an Ar balloon for at least 20 min prior to use. In an oven-dried 4 mL vial equipped with a Teflon®-coated magnetic stir bar, $CF_2HCOONa$ (94.4 mg, 4.0 equiv.), $K_2S_2O_8$ (16.2 mg, 0.30 equiv.), $Fe(NO_3)_3·9H_2O$ (12.1 mg, 15 mol%) and N-benzylideneaniline (**4**, 0.2 mmol, 1.0 equiv.) were added. The vial was evacuated and backfilled with Ar gas (repeated 3 times), followed by addition of MeCN (2 mL, 0.10 M with respect to the imine). The mixture was stirred (700 rpm) for 3 min to dissolve the iron complex, followed by addition of TMSOTf (0.11 mL, 3.0 equiv.). The rubber septum was quickly replaced with a sealing cap, which was tightened securely to maintain an Ar atmosphere, and the vial was then sealed with Parafilm. The reaction mixture was placed in a photoreactor equipped with a 40 W, 390 nm Kessil lamp and stirred at 700 rpm for 24 h under ethanol coolant circulation to maintain room temperature. After completion, the reaction mixture was diluted with DCM, washed with saturated aqueous Na_2CO_3, and the layers were separated. The aqueous layer was extracted with DCM twice. The combined organic layers were dried with anhydrous MgSO4, filtered, and concentrated under reduced pressure. Dibromomethane (0.10 mmol, 7 μL) was added to the crude mixture as an internal standard for NMR analysis. The yields were determined by relative integration of dibromomethane (δ 4.93 ppm, s, 2H, normalized to 2.0) and product **3** (δ 4.73 ppm, td, 1H).

C. Photochemical Measurements

UV-Vis absorption spectra were recorded using a 1 cm path-length quartz cuvette. Samples were prepared by dissolving iron nitrate nonahydrate ($Fe(NO_3)_3{\cdot}9H_2O$), potassium persulfate ($K_2S_2O_8$), and sodium difluoroacetate ($CF_2HCOONa$) both individually and in combination. To maintain an appropriate absorbance (Abs $\leq$ 1), the samples were diluted as needed. For the iron nitrate sample, the absorbance at the standard condition concentration (0.015 M) was too high; therefore, it was diluted 50-fold and measured at 0.30 mM. For $CF_2HCOONa$ (8.0 mM) and $K_2S_2O_8$ (0.60 mM), the solutions were prepared to match the molar equivalents employed under the standard conditions ($CF_2HCOONa$, 4.0 equiv.; $K_2S_2O_8$, 0.30 equiv.; $Fe(NO_3)_3{\cdot}9H_2O$, 0.15 equiv. relative to benzaldehyde). UV-Vis spectra were obtained at room temperature using a Thermo Scientific™ Orion AquaMate 8100 UV-Vis spectrophotometer.

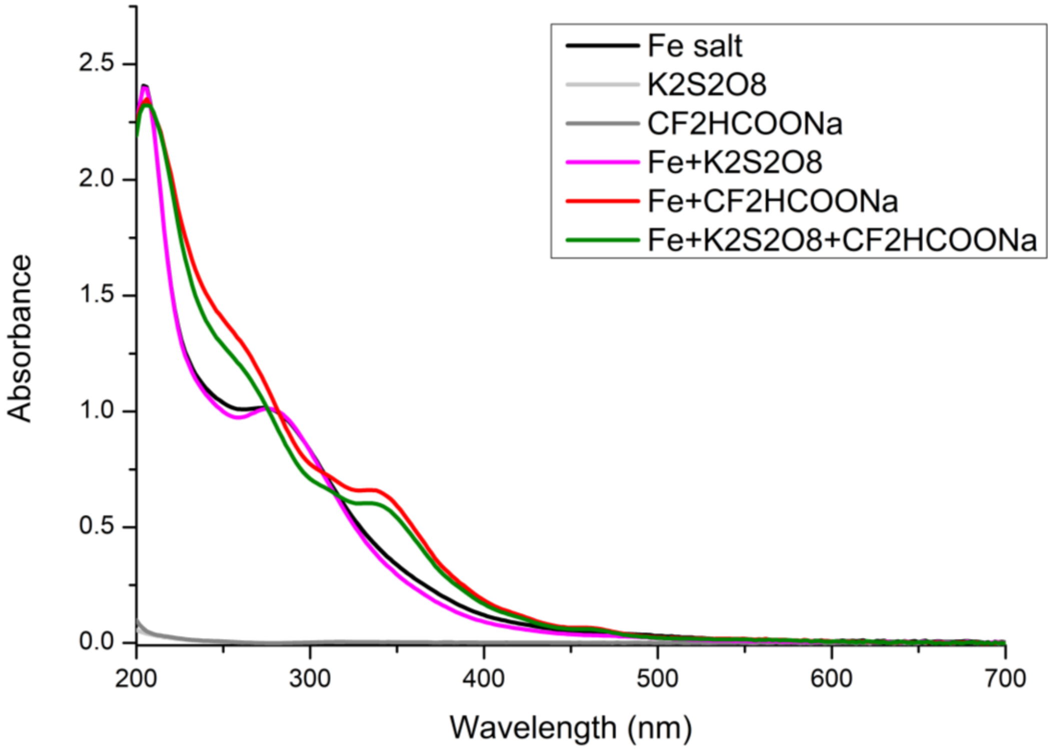

Figure 5. UV-Vis absorption spectra

***Notes:** A new absorption band appearing near 370 nm is observed in the red trace ($Fe(NO_3)_3{\cdot}9H_2O$ + $CF_2HCOONa$) and the green trace ($Fe(NO_3)_3{\cdot}9H_2O$ + $CF_2HCOONa$ + $K_2S_2O_8$). This feature suggests that, in the presence of both $Fe(NO_3)_3{\cdot}9H_2O$ and $CF_2HCOONa$, a productive LMCT-active iron complex is formed.

D. Light ON/OFF experiment

The benzaldehyde, aniline, and MeCN were degassed with an Ar balloon for at least 20 min prior to use. In an oven-dried 4 mL vial equipped with a Teflon®-coated magnetic stir bar, $CF_2HCOONa$ (94.4 mg, 4.0 equiv.), $K_2S_2O_8$ (16.2 mg, 0.30 equiv.) and $Fe(NO_3)_3{\cdot}9H_2O$ (12.1 mg, 15 mol%) were added. The vial was evacuated and backfilled with Ar gas (repeated 3 times), followed by addition of benzaldehyde (**1**, 0.2 mmol, 1.0 equiv.), aniline (**2**, 0.24 mmol, 1.2 equiv.) and MeCN (2 mL, 0.10 M with respect to the aldehyde). The mixture was stirred (700 rpm) for 3 min to dissolve the iron complex, followed by addition of TMSOTf (0.11 mL, 3.0 equiv.). The reaction mixture was placed in a photoreactor equipped with a 40 W, 390 nm Kessil lamp and stirred at 700 rpm under ethanol coolant circulation to maintain room temperature. The light was cycled on for 2 h and off for 1 h, and this irradiation program was repeated up to 11 h. At each time interval, an aliquot was taken and the product yield was determined by HPLC analysis using trimethoxybenzene as an internal standard.

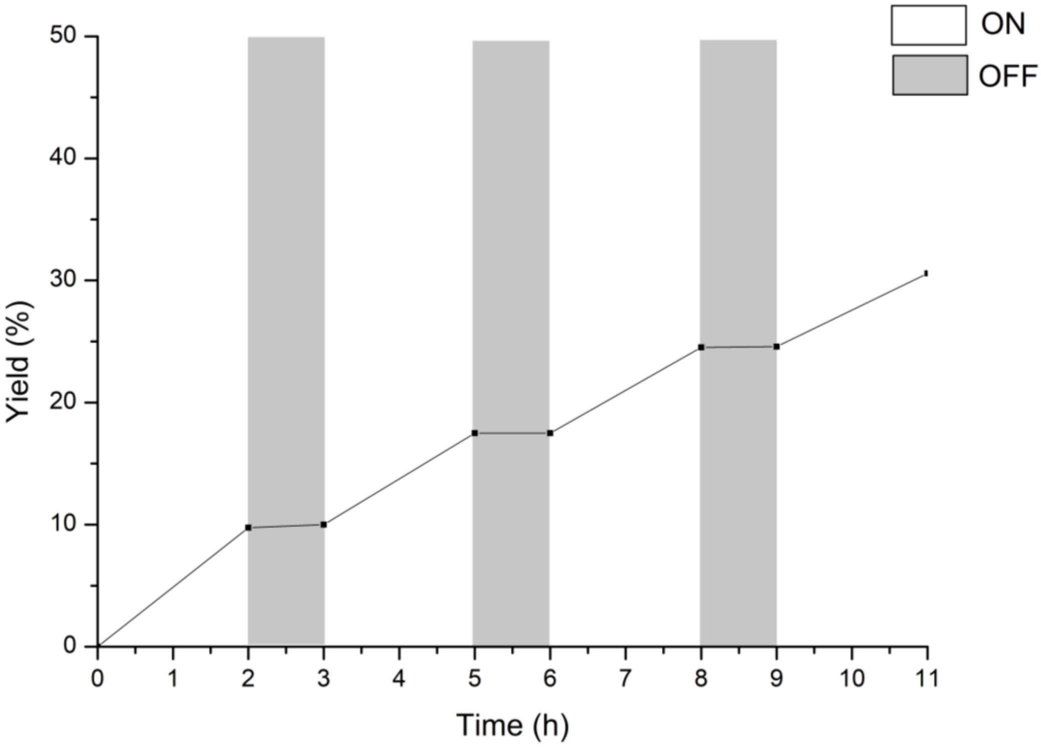

Figure 6. Light on/off experiment

***Notes:** We confirmed that the reaction proceeded only in the presence of light, which serves as evidence to rule out radical chain propagation mechanism.

E. Quantum Yield measurement

Part 1: Determination of Photon Flux of the Reactor

Photon flux was determined by chemical actinometry using potassium ferrioxalate in a setup using purple LEDs (Kessil PR160L-390nm Gen 2) as a source of irradiation. Purple LEDs were used because their emission profile lines up with known photochemical data for the actinometer. Great care is taken to ensure that the compound is kept in the dark when not in use.

Procedure for Photon Flux

The following solutions must be prepared ahead of time:

1. 0.05 M sulfuric acid stock solution

In a 100 mL volumetric flask, 0.281 mL of concentrated sulfuric acid (17.8 M) was added to 90 mL deionized water. Then, water was added until the 100 mL graduation mark was reached.

2. Ferrioxalate solution

A 0.15 M solution of potassium ferrioxolate was prepared by dissolving potassium ferrioxolate ($K_3FeC_2O_4{\cdot}3H_2O$, MW 491.243) (1.842 g, 3.75 mmol) with the 0.05 M sulfuric acid solution prepared in a 25 mL volumetric flask. Make every precaution to prepare and store the solution in the dark.

3. Developer solution

225 g of sodium acetate trihydrate was dissolved in 1 liter of 0.5 M sulfuric acid. 10 g of 1,10-phenantroline was added to this solution. Store in the dark.

To reproduce the irradiation conditions used under the standard reaction conditions, an oven-dried 4 mL vial equipped with a magnetic stir bar was purged with Ar and charged with 2 mL of 0.15 M aqueous potassium ferrioxalate solution. While stirring, the solution was irradiated with Kessil 390 nm LEDs at room temperature maintained by EtOH coolant circulation. 10 μL aliquots of solution were taken at time points between 1 and 5 minutes of irradiation. This aliquot is immediately added to 5 mL of the developer solution and the flask is wrapped in aluminum foil. A blank sample is prepared by adding 10 μL of the ferrioxalate solution to 5 mL of developer solution. The solutions were left in the dark for 30 minutes to an hour, eventually becoming bright red color. Solutions were transferred to a separate cuvette and the absorbance spectrum of the $Fe(phen)_3^{2+}$ complex was obtained. The absorbance at 510 nm (ε = 11,100 $M^{-1}cm^{-1}$) was measured for each sample.

Data Processing for Photon Flux

For each time point taken as indicated above, a plot of moles Fe^{2+} formed as a function of the time (in seconds) is generated. The moles of Fe^{2+} is given by the following equation V_1

$$moles\ Fe^{2+} = \frac{\Delta A_{510nm}\ V_1\ V_3}{\varepsilon_{510nm}\ l\ V_2}$$

$\Delta A_{510\ nm}$ = the absorbance difference between the sample and the blank at 510 nm.
l = the cuvette path length (1.0 cm).

ε = the molar extinction coefficient of the $Fe(phen)_3^{2+}$ complex at 510 nm (11,100 $M^{-1}\cdot cm^{-1}$).

V_1 = the total volume of the irradiated solution, is 2.0 mL (2 × 10^{-3} L) for Run#1, 0.2 mL (2 × 10^{-4} L) for Run#2.

V_2 = the volume of the aliquot taken from the solution, is 10.0 μL (10^{-5} L).

V_3 = the volume used to dilute the aliquot, is 5.0 mL (5 × 10^{-5} L).

Photon flux can be determined by

$$Photon\ flux = \frac{moles\ Fe^{2+}}{\Phi_{390nm} \times t \times F}$$

A plot of moles Fe^{2+} as a function of time yields a linear equation with an intercept at zero. Division of the slope by the documented quantum yield of the actinometer (Φ = 1.13 at 390 nm) and the mean fraction of light absorbed by the ferrioxalate solution (F ~ 1 at 390 nm at 0.15 M ferrioxalate) provides the photon flux in einsteins s^{-1}.[4-6]

Run	Time (s)	$\Delta A_{510\ nm}$	Moles Fe^{2+}
#1	0	0	0
	60	0.50972	4.59× 10^{-6}
	120	0.82378	7.42 × 10^{-6}
	180	1.24341	1.12 × 10^{-5}
	240	1.74076	1.57 × 10^{-5}
	300	2.20745	1.99 × 10^{-5}
Determined photon flux for Run #1 = 5.75× 10^{-7} einsteins s^{-1}			
#2	0	0	0
	60	0.04677	4.21 × 10^{-6}
	120	0.08398	7.56 × 10^{-6}
	180	0.12552	1.13 × 10^{-5}
	240	0.15079	1.36 × 10^{-5}
	300	0.20096	1.81 × 10^{-5}
Determined photon flux for Run #2 = 5.16× 10^{-7} einsteins s^{-1}			

***Notes**: Because the absorbance in Run #1 was too high, raising concerns about the validity of the Beer–Lambert law, Run #2 was performed using a 10-fold diluted solution.

Part 2: Determination of the Reaction Quantum Yield

The quantum yield is defined as:

$$\Phi = \frac{Moles\ of\ Product\ Formed}{Photons\ Absorbed\ by\ Sample}$$

If the transmittance of photons at 390nm LED is sufficiently small, it can be assumed that all the photons which pass through the cell are absorbed. The above equation may be then written as:

$$\Phi = \frac{Moles\ of\ Product\ Formed}{Photons\ Flux\ \times Time} = \frac{Rate\ of\ Product\ Formed}{Photon\ Flux}$$

Based on the experimental procedure and results of the UV–VIS absorption spectra, it is reasonable to assume that, at the concentration of the crude reaction mixture (0.1 M), all the photons which pass through the cell are absorbed. Accordingly, the reaction quantum yield can be determined using the equation above.

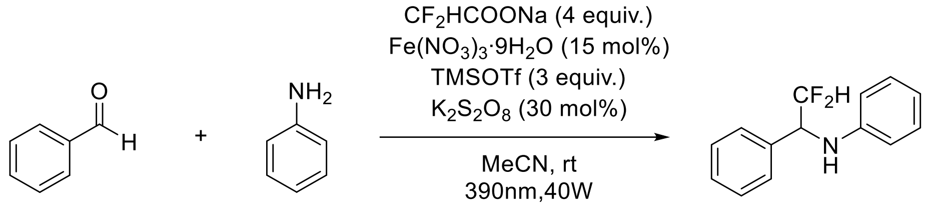

1 (1.0 equiv.) **2** (1.2 equiv.) **3**

The benzaldehyde, aniline, and MeCN were degassed with an Ar balloon for at least 20 min prior to use. In an oven-dried 4 mL vial equipped with a Teflon®-coated magnetic stir bar, $CF_2HCOONa$ (94.4 mg, 4.0 equiv.), $K_2S_2O_8$ (16.2 mg, 0.30 equiv.) and $Fe(NO_3)_3{\cdot}9H_2O$ (12.1 mg, 15 mol%) were added. The vial was evacuated and backfilled with Ar gas (repeated 3 times), followed by addition of benzaldehyde (**1**, 0.2 mmol, 1.0 equiv.), aniline (**2**, 0.24 mmol, 1.2 equiv.) and MeCN (2 mL, 0.10 M with respect to the aldehyde). The mixture was stirred (700 rpm) for 3 min to dissolve the iron complex, followed by addition of TMSOTf (0.11 mL, 3.0 equiv.). The reaction mixture was placed in a photoreactor equipped with a 40 W, 390 nm Kessil lamp and stirred at 700 rpm for 5 h under ethanol coolant circulation to maintain room temperature. Aliquots were taken every 30 min, and the product yield was determined by HPLC analysis using trimethoxybenzene as an internal standard.

Run	Time	Moles Product	Run	Time	Moles Product
**#1**	**0**	**0**	**#2**	**0**	**0**
	**3600**	**1.182×10^{-5}**		**1800**	**4.586×10^{-6}**
	**5400**	**1.720×10^{-5}**		**3600**	**1.129×10^{-5}**
	**7200**	**2.292×10^{-5}**		**5400**	**1.694×10^{-5}**
	**9000**	**2.919×10^{-5}**		**7200**	**2.375×10^{-5}**
	**10800**	**3.498×10^{-5}**		**9000**	**2.971×10^{-5}**
	**14400**	**4.775×10^{-5}**		**10800**	**3.449×10^{-5}**
	**18000**	**5.673×10^{-5}**		**14400**	**4.862×10^{-5}**
				**18000**	**5.787×10^{-5}**
Rate of Product Formation = 3.21×10^{-7} moles s^{-1}			Rate of Product Formation = 3.30×10^{-7} moles s^{-1}		

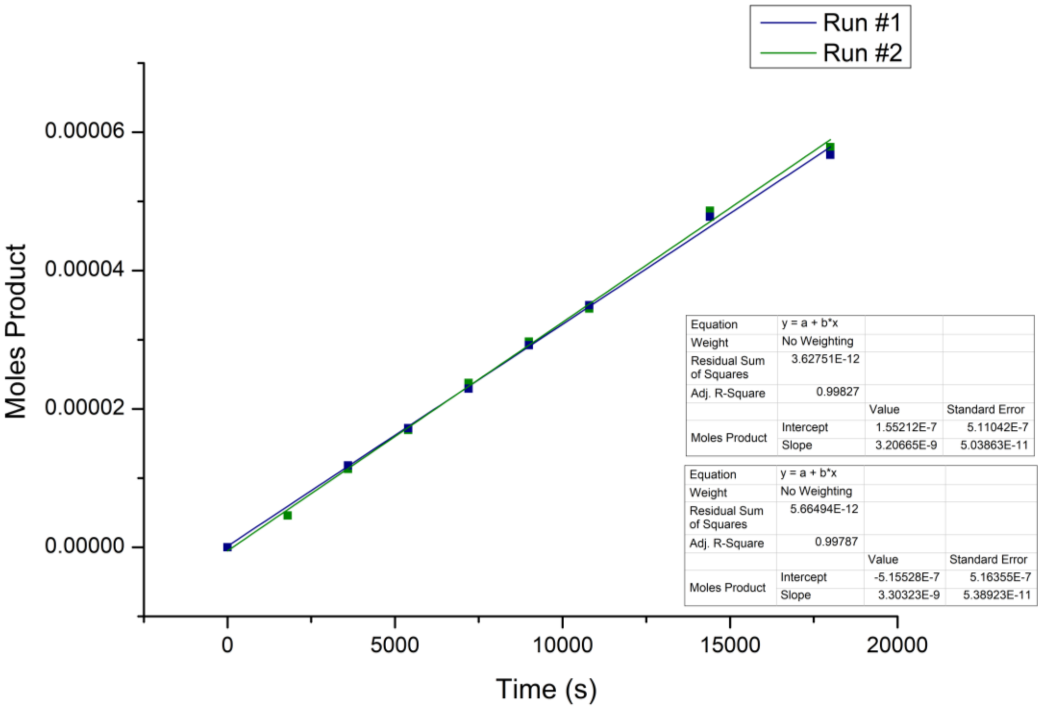

Figure 7. Rate of Product Formation

From this data, the quantum yield can be determined as Φ = 0. 006

$$\Phi = \frac{Rate\ of\ Product\ Formed}{Photons\ Flux} = \frac{3.25\ \times 10^{-9}\ (moles * s^{-1})}{5.45\ \times 10^{-7}\ (einsteins * s^{-1})} = 0.00597$$

***Notes:** The measured quantum yield was very low, which is consistent with the results in Section 5.D. and further supports ruling out a radical chain propagation mechanism.

F. Cyclic Voltammetry measurement

Cyclic voltammetry experiments were performed using a VSP potentiostat (Bio-Logic SAS, France) equipped with an SVC-3 voltammetry cell kit (Bio-Logic, A-012669). A three-electrode setup was employed, consisting of an Ag/AgCl electrode (LF-2-45, 3M NaCl) as the reference electrode, a 3 mm glassy carbon disk electrode (Bio-Logic, A-002012) as the working electrode, and a Pt wire as the counter electrode. Samples were prepared at a substrate concentration of 5.0 mM in MeCN with 0.1 M tetrabutylammonium hexafluorophosphate as the electrolyte. Before measurements, the sample solution was sparged with argon for 5 min. All experiments were conducted at a scan rate of 100 mV/s within the selected potential window. Between each measurement, the Ag/AgCl (3M NaCl) reference electrode and Pt wire counter electrode were rinsed with acetone, water, ethanol and MeCN, and the glassy carbon electrode was polished on CH Instruments MicroPolish alumina powder (0.05 µm). Voltammograms were plotted according to the polarographic convention. Oxidation potentials ($E_{p/2}$) were identified as half of the absolute maximum current during the reduction event. Fc/Fc+ couple redox potential was experimentally measured to be 0.283 V (vs Ag/AgCl) in the current setup; thus oxidation potential values obtained were referenced to Saturated Calomel Electrode (SCE) by using the conversion $E^0_{1/2}(Fc/Fc^+)$ = 0.400 (V vs SCE).[6,7]

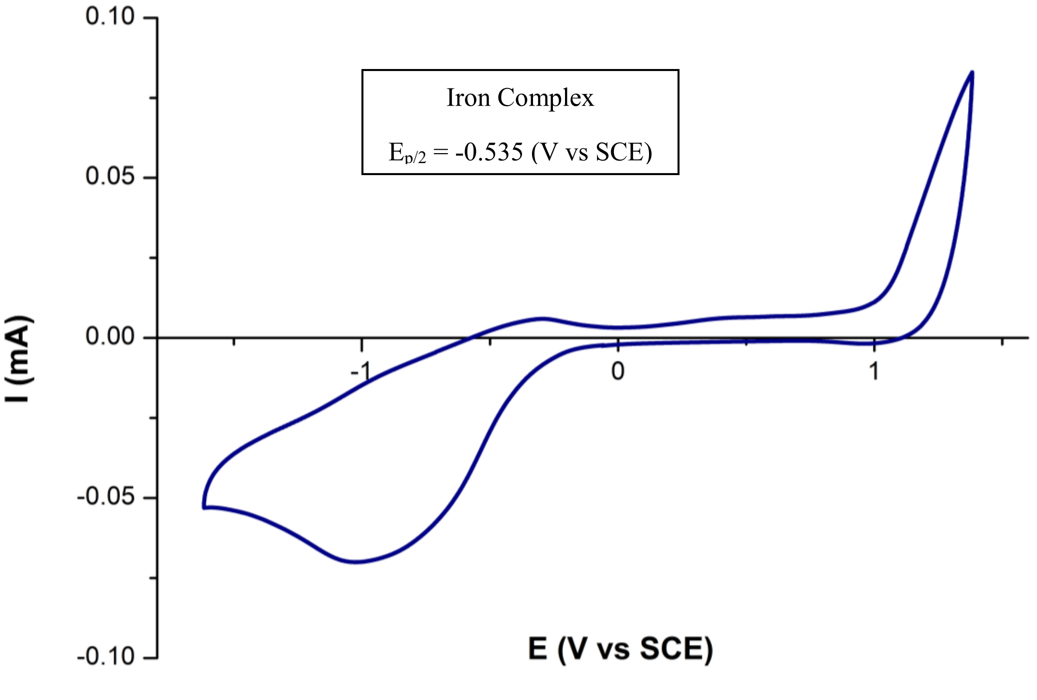

Figure 8. Cyclic Voltammogram of Iron complex

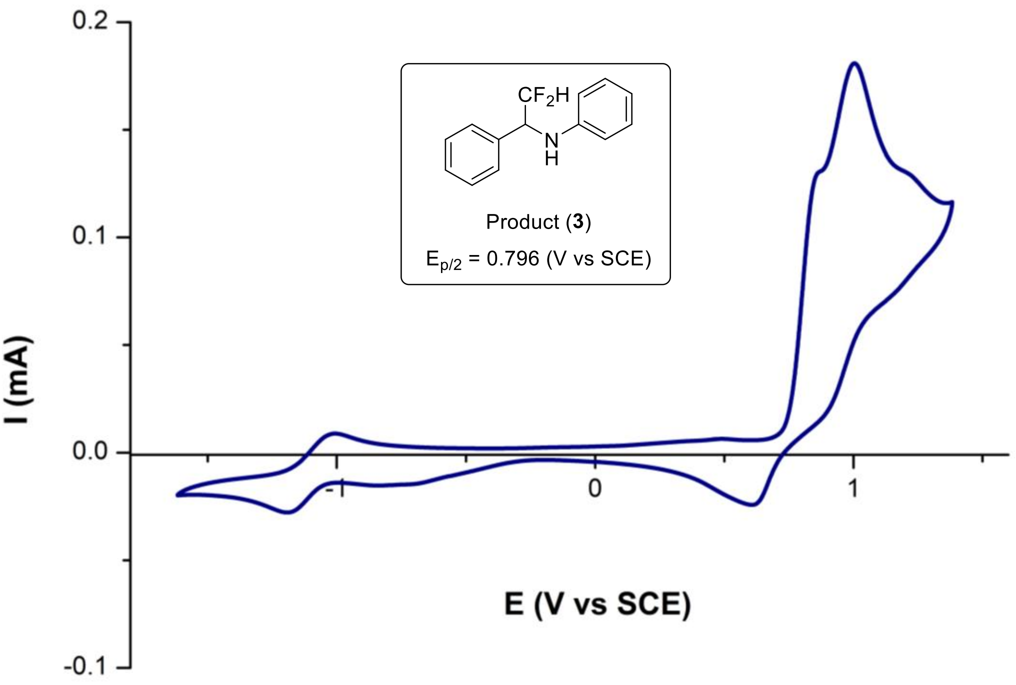

Figure 9. Cyclic Voltammogram of Product **3**.

***Notes:** The sum of the iron-complex reduction potential and the product oxidation potential is approximately −1.3 V, corresponding to a driving force greater than 1.3 V for the reverse electron-transfer process. Even accounting for the irreversible nature of the voltammograms, these data suggest that the single electron-transfer process, namely reduction of the N-radical intermediate by the Fe(II) complex, is thermodynamically favored.

G. Time-Course Reaction Profiles

General Procedure for Reaction Yield Tracking

The benzaldehyde, aniline, and MeCN were degassed with an Ar balloon for at least 20 min prior to use. In an oven-dried 4 mL vial equipped with a Teflon®-coated magnetic stir bar, $CF_2HCOONa$ (94.4 mg, 4.0 equiv.), $K_2S_2O_8$ (16.2 mg, 0.30 equiv.) and $Fe(NO_3)_3{\cdot}9H_2O$ (12.1 mg, 15 mol%) were added. The vial was evacuated and backfilled with Ar gas (repeated 3 times), followed by addition of benzaldehyde (**1**, 0.2 mmol, 1.0 equiv.), aniline (**2**, 0.24 mmol, 1.2 equiv.) and MeCN (2 mL, 0.10 M with respect to the aldehyde). The mixture was stirred (700 rpm) for 3 min to dissolve the iron complex, followed by addition of TMSOTf (0.11 mL, 3.0 equiv.). The reaction mixture was placed in a photoreactor equipped with a 40 W, 390 nm Kessil lamp and stirred at 700 rpm under ethanol coolant circulation to maintain room temperature. Aliquots were taken every 15 min, and the product yield was determined by HPLC analysis using trimethoxybenzene as an internal standard. At each time point, the concentration of the starting material was estimated from the product concentration.

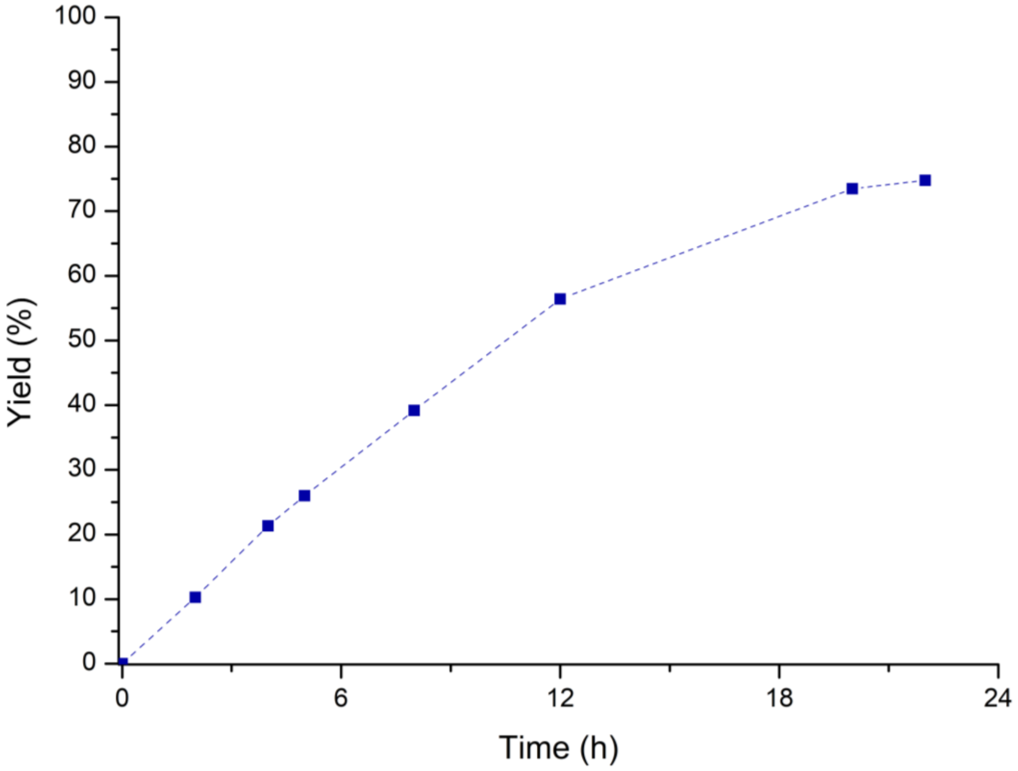

Figure 10. Time-course reaction profiles

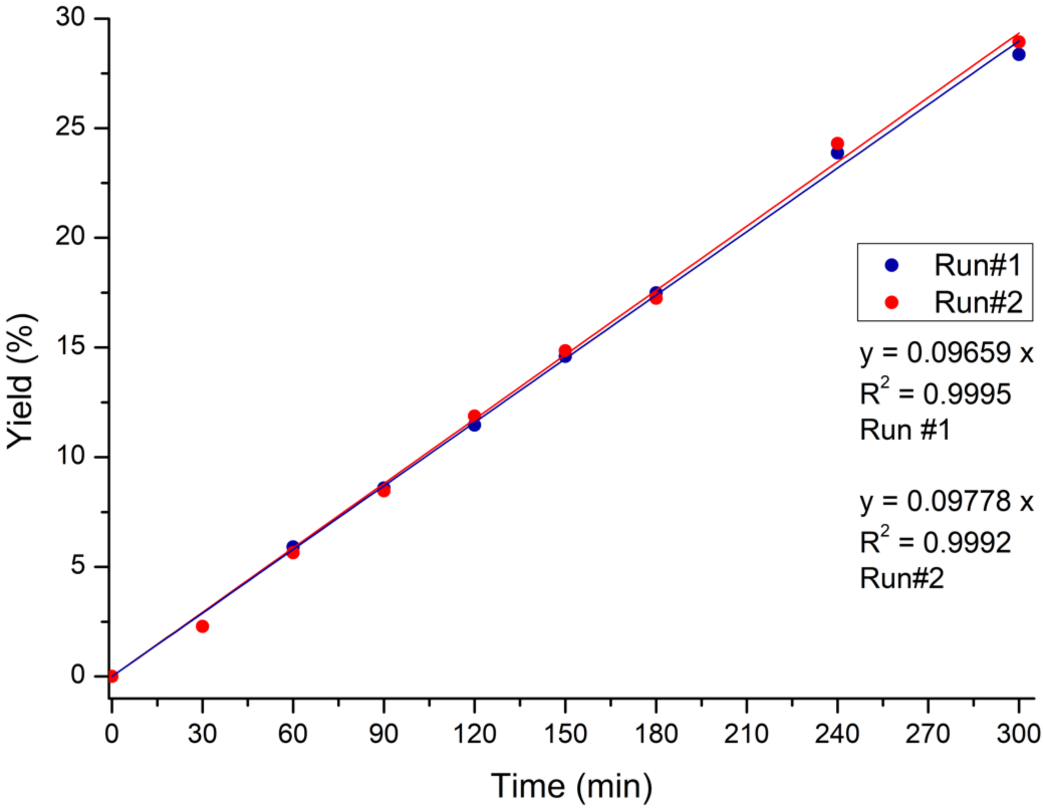

Figure 11. Time-course reaction profile recorded every 30 min for the first 5 h after reaction initiation

***Notes:** While monitoring the time-course reaction profile, we observed that the reaction did not exhibit a rapid initial acceleration during the first 5 hours but instead proceeded slowly with an approximately constant rate. Accordingly, we measured the initial rate at regular time intervals. This kinetic feature motivated subsequent, more detailed kinetic studies.

H. Variable Time Normalization Analysis (VTNA)

General Procedure for VTNA kinetics

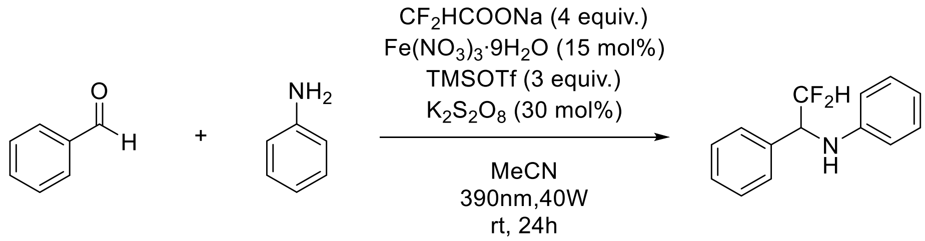

1 (1.0 equiv.) **2** (1.2 equiv.) **3**

Standard Conditions: The benzaldehyde, aniline, and MeCN were degassed with an Ar balloon for at least 20 min prior to use. In an oven-dried 4 mL vial equipped with a Teflon®-coated magnetic stir bar, $CF_2HCOONa$ (94.4 mg, 4.0 equiv.), $K_2S_2O_8$ (16.2 mg, 0.30 equiv.) and $Fe(NO_3)_3{\cdot}9H_2O$ (12.1 mg, 15 mol%) were added. The vial was evacuated and backfilled with Ar gas (repeated 3 times), followed by addition of benzaldehyde (**1**, 0.2 mmol, 1.0 equiv.), aniline (**2**, 0.24 mmol, 1.2 equiv.) and MeCN (2 mL, 0.10 M with respect to the aldehyde). The mixture was stirred (700 rpm) for 3 min to dissolve the iron complex, followed by addition of TMSOTf (0.11 mL, 3.0 equiv.). The reaction mixture was placed in a photoreactor equipped with a 40 W, 390 nm Kessil lamp and stirred at 700 rpm under ethanol coolant circulation to maintain room temperature. Aliquots were taken every 15 min, and the product yield was determined by HPLC analysis using trimethoxybenzene as an internal standard. At each time point, the concentration of the starting material was estimated from the product concentration.

The variable time normalization analysis was conducted by varying the initial concentration of reaction component **1** or **2** to 100% (standard conditions), 75%, and 50% of the standard loading. Concentration data was then normalized based on variable-time normalization analysis to construct the time integral for a reaction component of interest. The kinetic order corresponds to the value of the exponent “a”, which allows for the overlay of the reaction profiles using the trapezoidal approximation, as described by Burés and coworkers.[8]

$$\int_{t=0}^{t=t_n}[A]^a dt = \sum_{i=1}^{n}\left(\frac{[A]_i + [A]_{i-1}}{2}\right)^a (t_i - t_{i-1})$$

Determining Kinetic Order of **1**

(i) The 0th order fitting of **1**

$[\mathbf{1}]_0$ = 50 Mm		$[\mathbf{1}]_0$ = 75 Mm		$[\mathbf{1}]_0$ = 100 Mm	
$\Sigma\,[\mathbf{1}]^0\Delta t$	[**3**] (mM)	$\Sigma\,[\mathbf{1}]^0\Delta t$	[**3**] mM	$\Sigma\,[\mathbf{1}]^0\Delta t$	[**3**] mM
0	0	0	0	0	0
15	1.72938	15	1.482268	15	1.043651
30	3.687142	30	3.09376	30	2.401744
45	5.660076	45	4.600388	45	3.62443
60	7.948684	60	6.274914	60	4.770216

75	10.05166	75	8.311275	75	5.968039
90	12.12261	90	9.751282	90	7.109716
105	14.16904	105	11.67893	105	8.405641
120	16.79093	120	13.7077	120	9.743712
135	17.533	135	14.211	135	10.83141
150	18.20358	150	16.0793	150	12.05532

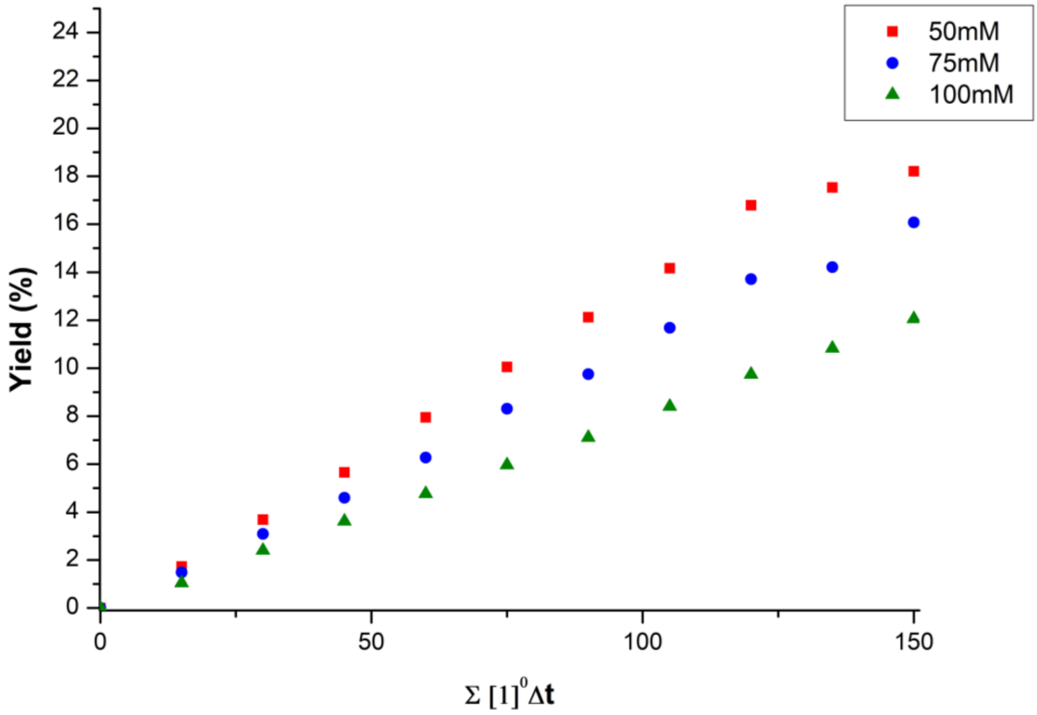

Figure 12. The variable time normalization analysis at an order of 1 for substrate **1**

(ii) The -0.6 order fitting of **1**

[**1**]$_0$ = 50 Mm		[**1**]$_0$ = 75 Mm		[**1**]$_0$ = 100 Mm	
Σ [**1**]$^{-0.6}$Δt	[**3**] (mM)	Σ [**1**]$^{-0.6}$Δt	[**3**] mM	Σ [**1**]$^{-0.6}$Δt	[**3**] mM
0	0	0	0	0	0
1.449623	1.72938	1.131468	1.482268	0.949412	1.043651
2.932892	3.687142	2.277318	3.09376	1.905767	2.401744
4.454423	5.660076	3.438166	4.600388	2.869737	3.62443
6.020551	7.948684	4.614869	6.274914	3.840838	4.770216
7.636479	10.05166	5.810815	8.311275	4.819137	5.968039
9.303862	12.12261	7.025569	9.751282	5.804764	7.109716
11.02652	14.16904	8.259315	11.67893	6.798185	8.405641
12.81815	16.79093	9.516417	13.7077	7.800214	9.743712
14.66429	17.533	10.7891	14.211	8.81035	10.83141

16.53468	18.20358	12.07685	16.0793	9.828375	12.05532

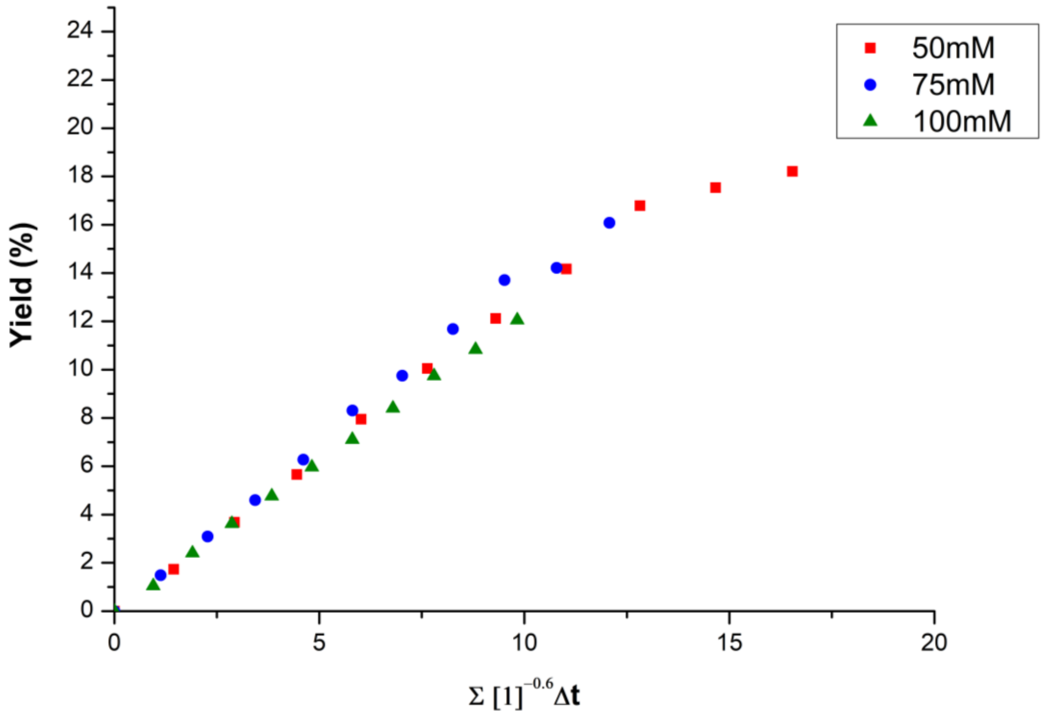

Figure 13. The variable time normalization analysis at an order of -0.6 for substrate **1**

(i) The 1st order fitting of **1**

[**1**]$_0$ = 50 Mm		[**1**]$_0$ = 75 Mm		[**1**]$_0$ = 100 Mm	
Σ [**1**]^{1}Δt	[**3**] (mM)	Σ [**1**]^{1}Δt	[**3**] mM	Σ [**1**]^{1}Δt	[**3**] mM
0	0	0	0	0	0
737.0296	1.72938	1113.883	1.482268	1492.173	1.043651
1446.406	3.687142	2204.563	3.09376	2966.332	2.401744
2126.302	5.660076	3271.857	4.600388	4421.136	3.62443
2774.236	7.948684	4315.292	6.274914	5858.176	4.770216
3389.233	10.05166	5330.895	8.311275	7277.639	5.968039
3972.926	12.12261	6320.426	9.751282	8679.556	7.109716
4525.739	14.16904	7284.7	11.67893	10063.19	8.405641
5043.539	16.79093	8219.3	13.7077	11427.07	9.743712
5536.11	17.533	9134.91	14.211	12772.76	10.83141
6018.085	18.20358	10032.73	16.0793	14101.11	12.05532

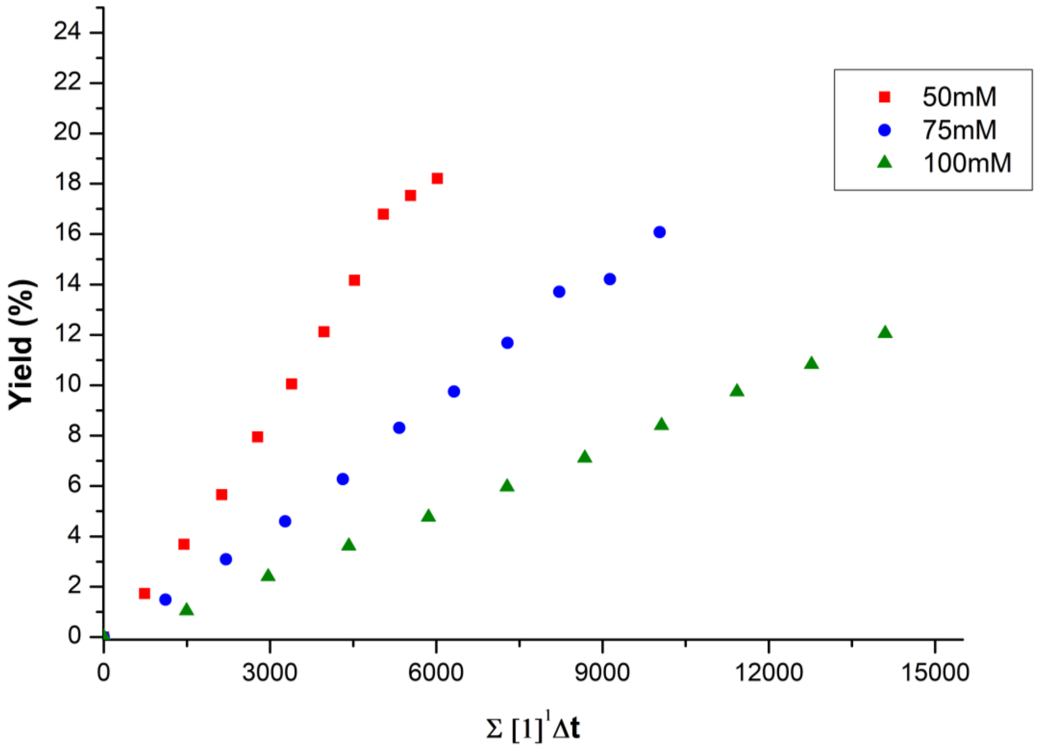

Figure 14. The variable time normalization analysis at an order of 1 for substrate **1**

Determining Kinetic Order of **2**

(i) The 0th order fitting of **2**

[**2**]$_0$ = 60 Mm		[**2**]$_0$ = 90 Mm		[**2**]$_0$ = 120 Mm	
Σ [**2**]^{0}Δt	[**3**] (mM)	Σ [**2**]^{0}Δt	[**3**] mM	Σ [**2**]^{0}Δt	[**3**] mM
0	0	0	0	0	0
15	1.382601	15	1.247109	15	1.043651
30	2.981212	30	2.564319	30	2.401744
45	4.704753	45	3.813424	45	3.62443
60	6.583198	60	5.182077	60	4.770216
75	8.256837	75	6.663402	75	5.968039
90	9.912698	90	8.054588	90	7.109716
105	11.91239	105	9.709626	105	8.405641
120	13.30273	120	11.18506	120	9.743712
135	14.79897	135	12.83432	135	10.83141
150	16.25966	150	13.99432	150	12.05532

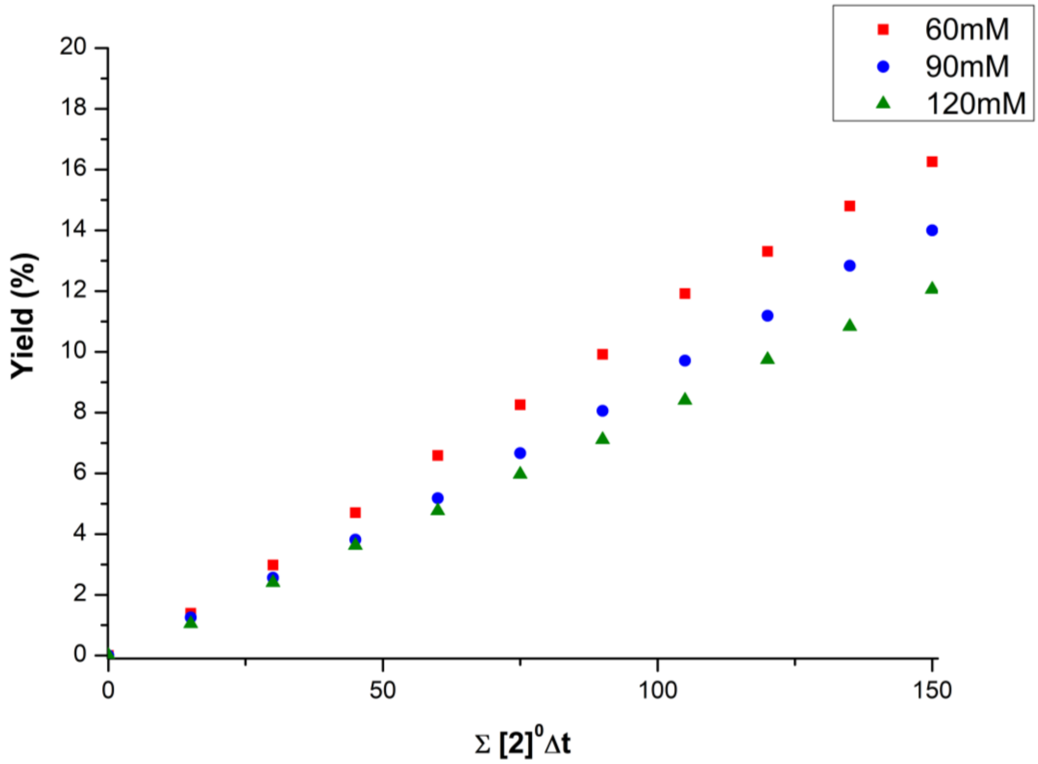

Figure 15. The variable time normalization analysis at an order of 0 for substrate **2**

(ii) The -0.5 order fitting of **2**

[**2**]$_0$ = 60 Mm		[**2**]$_0$ = 90 Mm		[**2**]$_0$ = 120 Mm	
Σ [**2**]$^{-0.5}$Δt	[**3**] (mM)	Σ [**2**]$^{-0.5}$Δt	[**3**] mM	Σ [**2**]$^{-0.5}$Δt	[**3**] mM
0	0	0	0	0	0
1.587246	1.382601	1.293986	1.247109	1.119658	1.043651
3.194245	2.981212	2.597247	2.564319	2.244729	2.401744
4.824425	4.704753	3.910166	3.813424	3.376084	3.62443
6.480861	6.583198	5.232917	5.182077	4.513262	4.770216
8.165421	8.256837	6.566653	6.663402	5.656185	5.968039
9.877201	9.912698	7.912015	8.054588	6.805022	7.109716
11.61937	11.91239	9.269548	9.709626	7.959936	8.405641
13.3941	13.30273	10.64058	11.18506	9.121637	9.743712
15.19601	14.79897	12.02498	12.83432	10.28995	10.83141
17.02757	16.25966	13.42271	13.99432	11.46433	12.05532

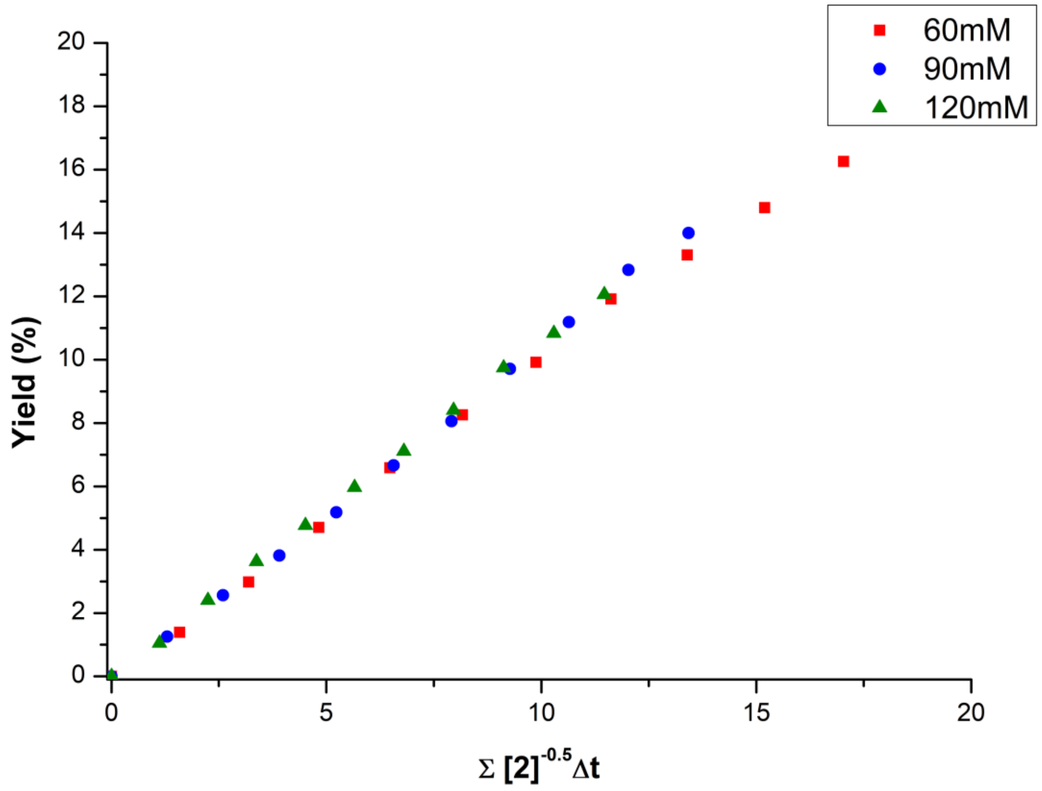

Figure 16. The variable time normalization analysis at an order of -0.5 for substrate **2**

(i) The 1st order fitting of **2**

$[\mathbf{2}]_0$ = 60 Mm		$[\mathbf{2}]_0$ = 90 Mm		$[\mathbf{2}]_0$ = 120 Mm	
Σ $[\mathbf{2}]^1$Δt	[**3**] (mM)	Σ $[\mathbf{2}]^1$Δt	[**3**] mM	Σ $[\mathbf{2}]^1$Δt	[**3**] mM
0	0	0	0	0	0
1339.63	1.382601	2015.647	1.247109	2692.173	1.043651
2646.532	2.981212	4002.708	2.564319	5358.505	2.401744
3916.529	4.704753	5960.642	3.813424	7995.295	3.62443
5146.583	6.583198	7889.575	5.182077	10605.15	4.770216
6335.909	8.256837	9786.869	6.663402	13188.84	5.968039
7487.711	9.912698	11651.51	8.054588	15746	7.109716
8599.678	11.91239	13482.87	9.709626	18276.31	8.405641
9671.222	13.30273	15278.33	11.18506	20777.14	9.743712
10710.69	14.79897	17039.3	12.83432	23249.75	10.83141
11716.76	16.25966	18766.83	13.99432	25696.87	12.05532

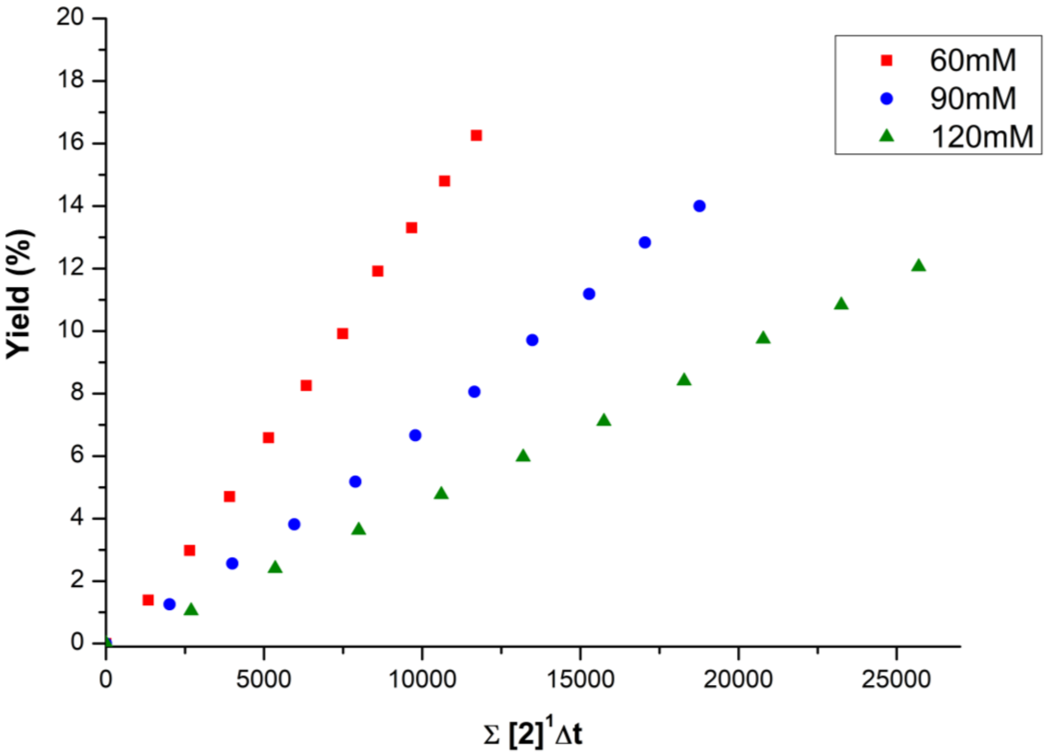

Figure 17. The variable time normalization analysis at an order of 1 for substrate **2**

General Procedure for VTNA kinetics for intermediate 4

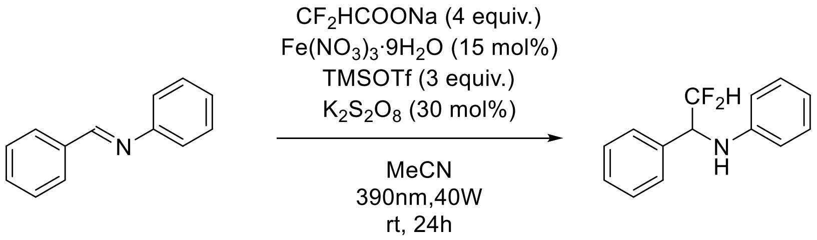

4 (1.0 equiv.) **3**

Standard Conditions: The MeCN was degassed with an Ar balloon for at least 20 min prior to use. In an oven-dried 4 mL vial equipped with a Teflon®-coated magnetic stir bar, $CF_2HCOONa$ (94.4 mg, 4.0 equiv.), $K_2S_2O_8$ (16.2 mg, 0.30 equiv.), $Fe(NO_3)_3·9H_2O$ (12.1 mg, 15 mol%) and N-benzylideneaniline (**4**, 0.2 mmol, 1.0 equiv.) were added. The vial was evacuated and backfilled with Ar gas (repeated 3 times), followed by addition of MeCN (2 mL, 0.10 M with respect to the imine). The mixture was stirred (700 rpm) for 3 min to dissolve the iron complex, followed by addition of TMSOTf (0.11 mL, 3.0 equiv.). The reaction mixture was placed in a photoreactor equipped with a 40 W, 390 nm Kessil lamp and stirred at 700 rpm under ethanol coolant circulation to maintain room temperature. Aliquots were taken every 15 min, and the product yield was determined by HPLC analysis using trimethoxybenzene as an internal standard. At each time point, the concentration of the starting material was estimated from the product concentration.

The variable time normalization analysis was conducted by varying the initial concentration of reaction component **4** to 100% (standard conditions), 75%, and 50% of the standard loading. Concentration data was then normalized based on variable-time normalization analysis to construct the time integral for a reaction component of interest. The kinetic order corresponds to the value of the exponent "a", which allows for the overlay of the reaction profiles using the trapezoidal approximation, as described by Burés and coworkers.[8]

$$\int_{t=0}^{t=t_n}[A]^a dt = \sum_{i=1}^{n}(\frac{[A]_i + [A]_{i-1}}{2})^a(t_i - t_{i-1})$$

Determining Kinetic Order of **4**

(i) The 0th order fitting of **4**

$[\mathbf{4}]_0$ = 50 Mm		$[\mathbf{4}]_0$ = 75 Mm		$[\mathbf{4}]_0$ = 100 Mm	
Σ $[\mathbf{4}]^0\Delta$t	[**3**] (mM)	Σ $[\mathbf{4}]^0\Delta$t	[**3**] mM	Σ $[\mathbf{4}]^0\Delta$t	[**3**] mM
0	0	0	0	0	0
15	2.434562	15	1.817385	15	1.334228
30	5.000793	30	3.621702	30	2.871803
45	7.673797	45	5.477217	45	4.52756
60	10.30759	60	7.238567	60	6.03061
75	13.05102	75	9.021739	75	7.5859
90	15.77916	90	10.73875	90	9.111048
105	18.52655	105	12.55001	105	10.82283
120	21.33559	120	14.39327	120	12.41164
135	24.87878	135	16.25866	135	14.29331
150	27.07404	150	18.21636	150	15.91316

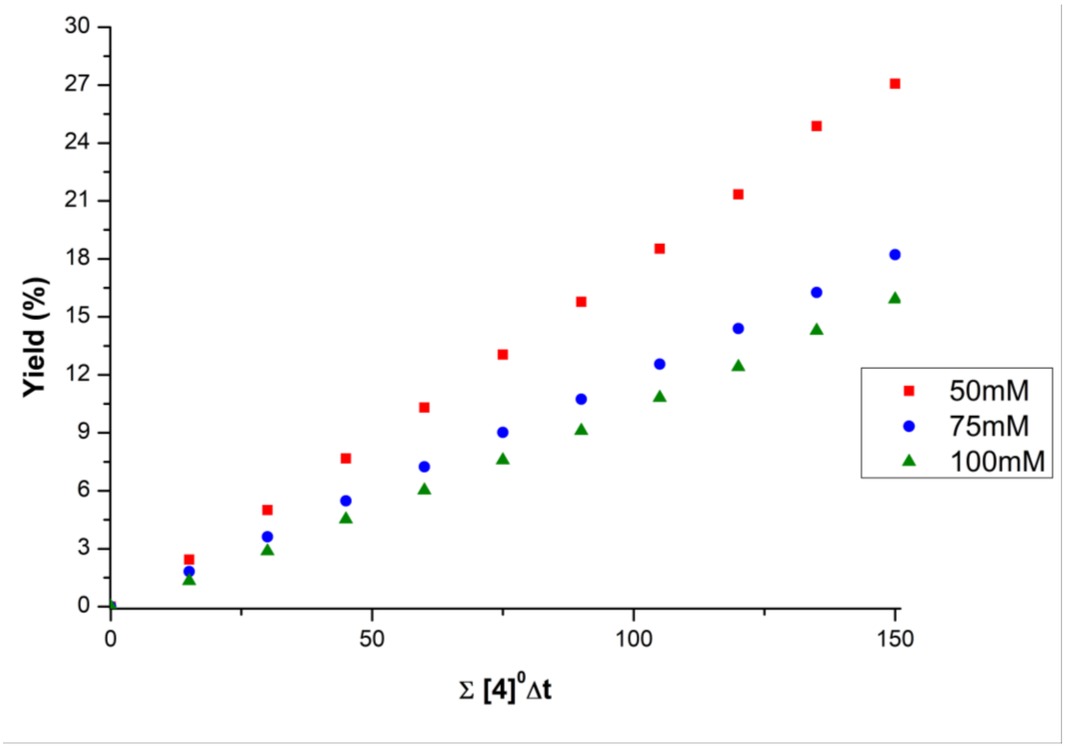

Figure 18. The variable time normalization analysis at an order of 0 for intermediate **4**

(ii) The -0.7 order fitting of **4**

[**4**]$_0$ = 50 Mm		[**4**]$_0$ = 75 Mm		[**4**]$_0$ = 100 Mm	
Σ [**4**]$^{-0.7}$Δt	[**3**] (mM)	Σ [**4**]$^{-0.7}$Δt	[**3**] mM	Σ [**4**]$^{-0.7}$Δt	[**3**] mM
0	0	0	0	0	0
0.986973	2.434562	0.736638	1.817385	0.599965	1.334228
2.010974	5.000793	1.486147	3.621702	1.206077	2.871803
3.077604	7.673797	2.249231	5.477217	1.819206	4.52756
4.192087	10.30759	3.026332	7.238567	2.439474	6.03061
5.360747	13.05102	3.817794	9.021739	3.066849	7.5859
6.591597	15.77916	4.624086	10.73875	3.701585	9.111048
7.893393	18.52655	5.446029	12.55001	4.344287	10.82283
9.278262	21.33559	6.284984	14.39327	4.995367	12.41164
10.77569	24.87878	7.142103	16.25866	5.655546	14.29331
12.39617	27.07404	8.018981	18.21636	6.325226	15.91316

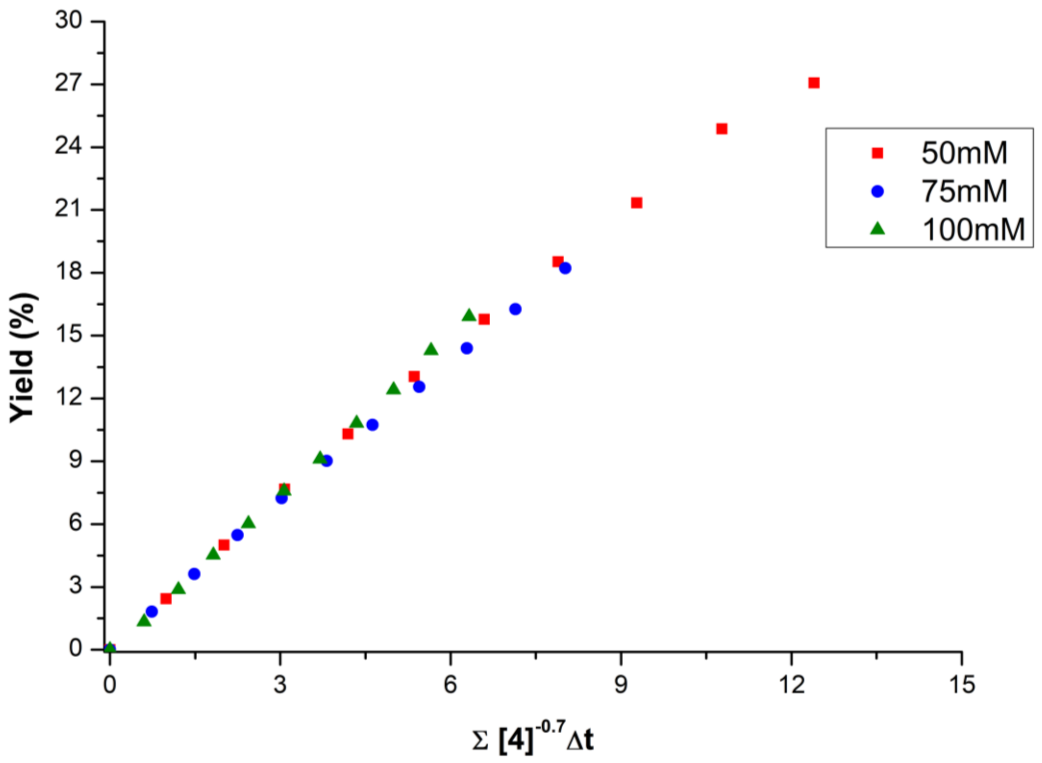

Figure 19. The variable time normalization analysis at an order of -0.7 for intermediate **4**

(iii) The 1st order fitting of **4**

$[\mathbf{4}]_0$ = 50 Mm		$[\mathbf{4}]_0$ = 75 Mm		$[\mathbf{4}]_0$ = 100 Mm	
$\Sigma\ [\mathbf{4}]^1\Delta t$	[**3**] (mM)	$\Sigma\ [\mathbf{4}]^1\Delta t$	[**3**] mM	$\Sigma\ [\mathbf{4}]^1\Delta t$	[**3**] mM
0	0	0	0	0	0
1339.63	2.434562	2015.647	1.817385	2692.173	1.334228
2646.532	5.000793	4002.708	3.621702	5358.505	2.871803
3916.529	7.673797	5960.642	5.477217	7995.295	4.52756
5146.583	10.30759	7889.575	7.238567	10605.15	6.03061
6335.909	13.05102	9786.869	9.021739	13188.84	7.5859
7487.711	15.77916	11651.51	10.73875	15746	9.111048
8599.678	18.52655	13482.87	12.55001	18276.31	10.82283
9671.222	21.33559	15278.33	14.39327	20777.14	12.41164
10710.69	24.87878	17039.3	16.25866	23249.75	14.29331
11716.76	27.07404	18766.83	18.21636	25696.87	15.91316

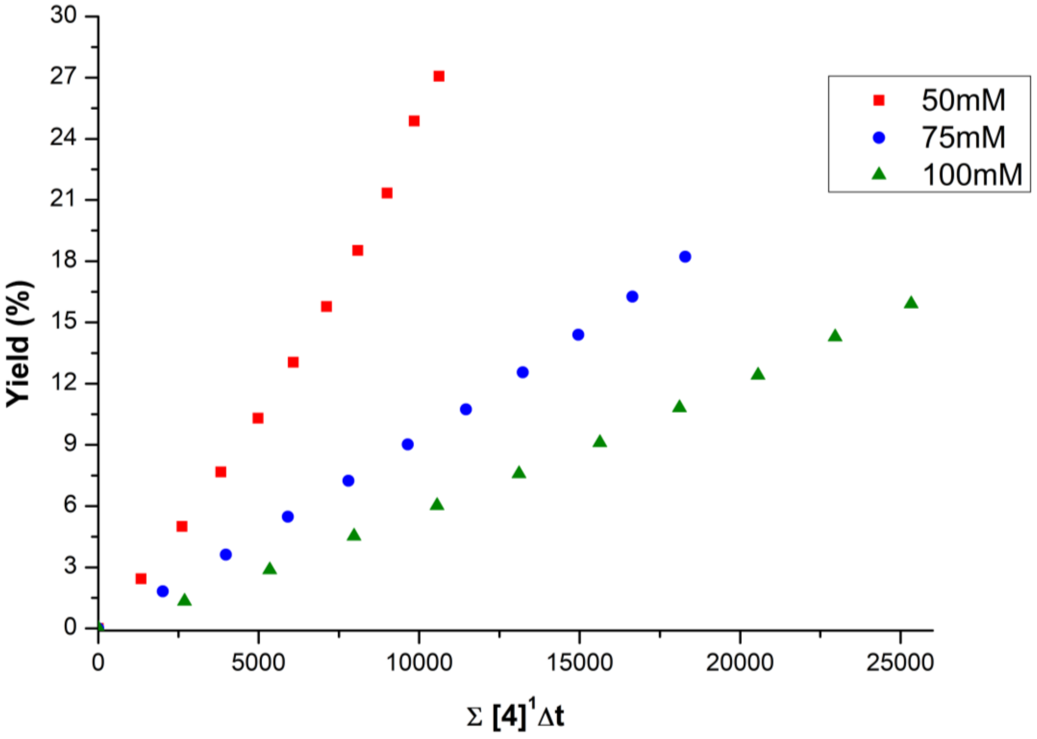

Figure 20. The variable time normalization analysis at an order of 1 for intermediate **4**

I. Kinetic studies to Initial Rate Law Determination

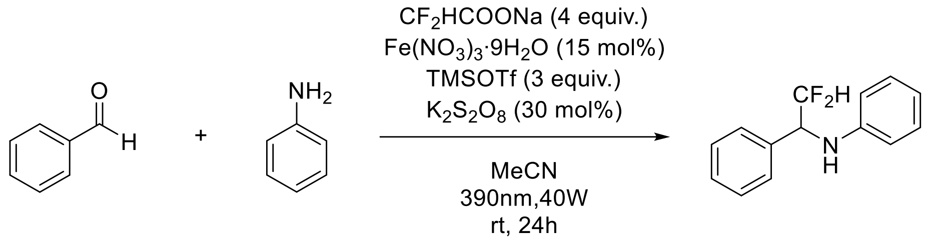

1 (1.0 equiv.) **2** (1.2 equiv.) **3**

Standard Conditions: The benzaldehyde, aniline, and MeCN were degassed with an Ar balloon for at least 20 min prior to use. In an oven-dried 4 mL vial equipped with a Teflon®-coated magnetic stir bar, $CF_2HCOONa$ (94.4 mg, 4.0 equiv.), $K_2S_2O_8$ (16.2 mg, 0.30 equiv.) and $Fe(NO_3)_3{\cdot}9H_2O$ (12.1 mg, 15 mol%) were added. The vial was evacuated and backfilled with Ar gas (repeated 3 times), followed by addition of benzaldehyde (**1**, 0.2 mmol, 1.0 equiv.), aniline (**2**, 0.24 mmol, 1.2 equiv.) and MeCN (2 mL, 0.10 M with respect to the aldehyde). The mixture was stirred (700 rpm) for 3 min to dissolve the iron complex, followed by addition of TMSOTf (0.11 mL, 3.0 equiv.). The reaction mixture was placed in a photoreactor equipped with a 40 W, 390 nm Kessil lamp and stirred at 700 rpm under ethanol coolant circulation to maintain room temperature. Aliquots were taken every 15 min, and the product yield was determined by HPLC analysis using trimethoxybenzene as an internal standard.

Based on the standard conditions described above, we independently varied the concentrations of $CF_2HCOONa$, $K_2S_2O_8$, $Fe(NO_3)_3{\cdot}9H_2O$, and TMSOTf, as well as the light intensity. The initial reaction rate was plotted as a function of each factor.

(i) Initial reaction rate versus $Fe(NO_3)_3{\cdot}9H_2O$ concentration

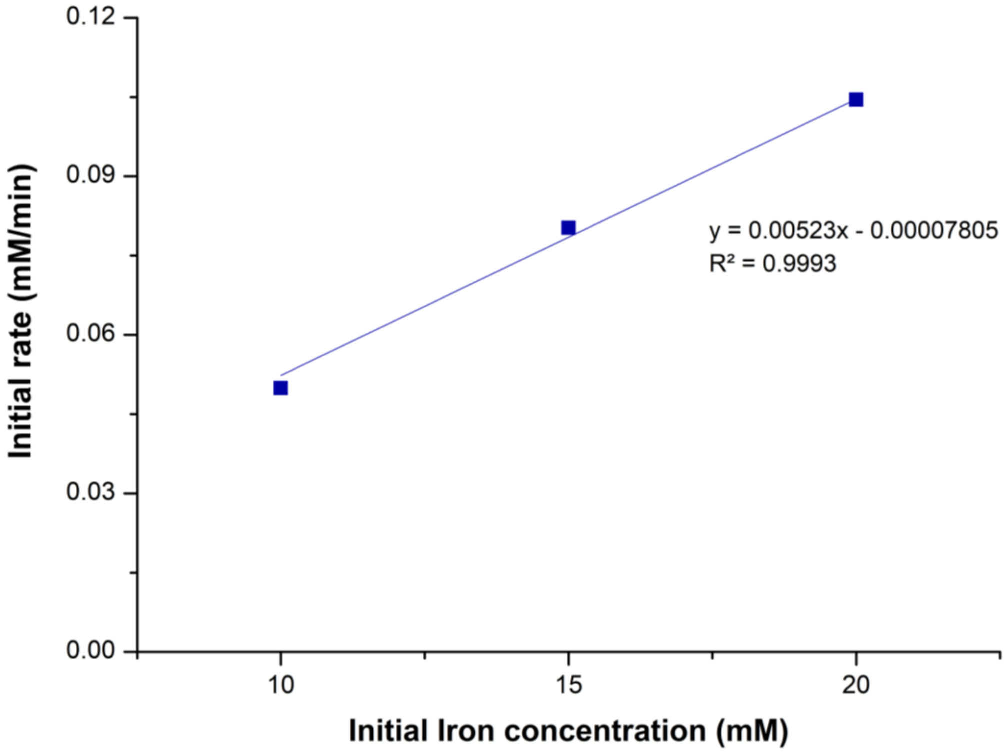

(ii) Initial reaction rate versus $CF_2HCOONa$ concentration

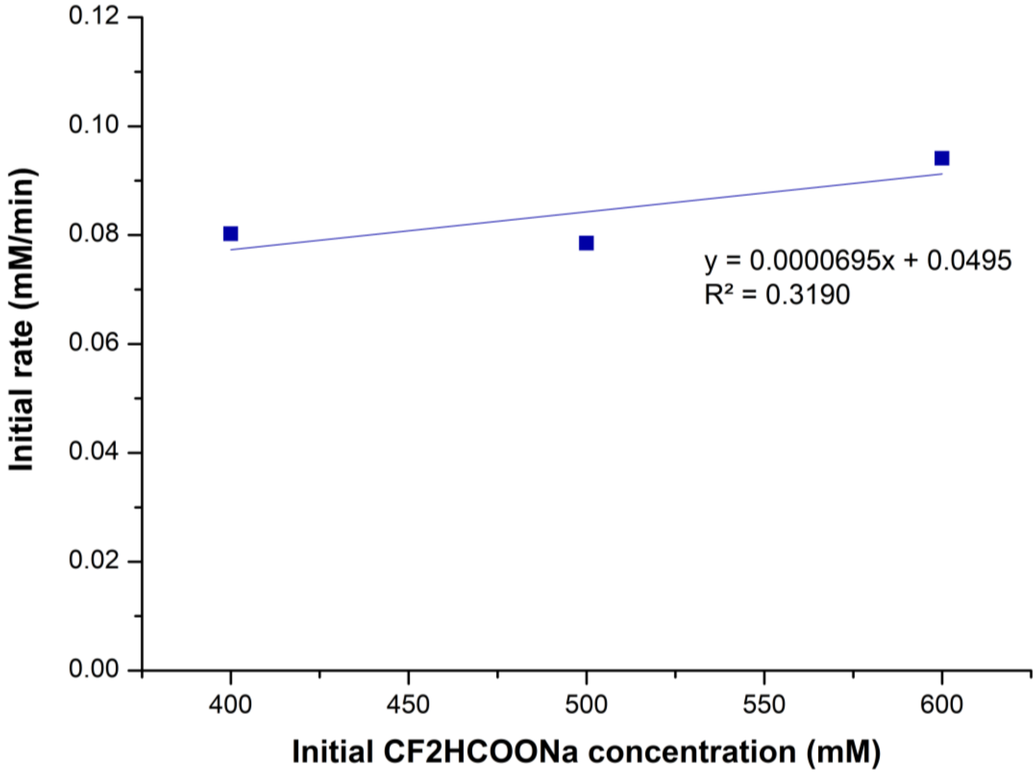

(iii) Initial reaction rate versus $K_2S_2O_8$ concentration

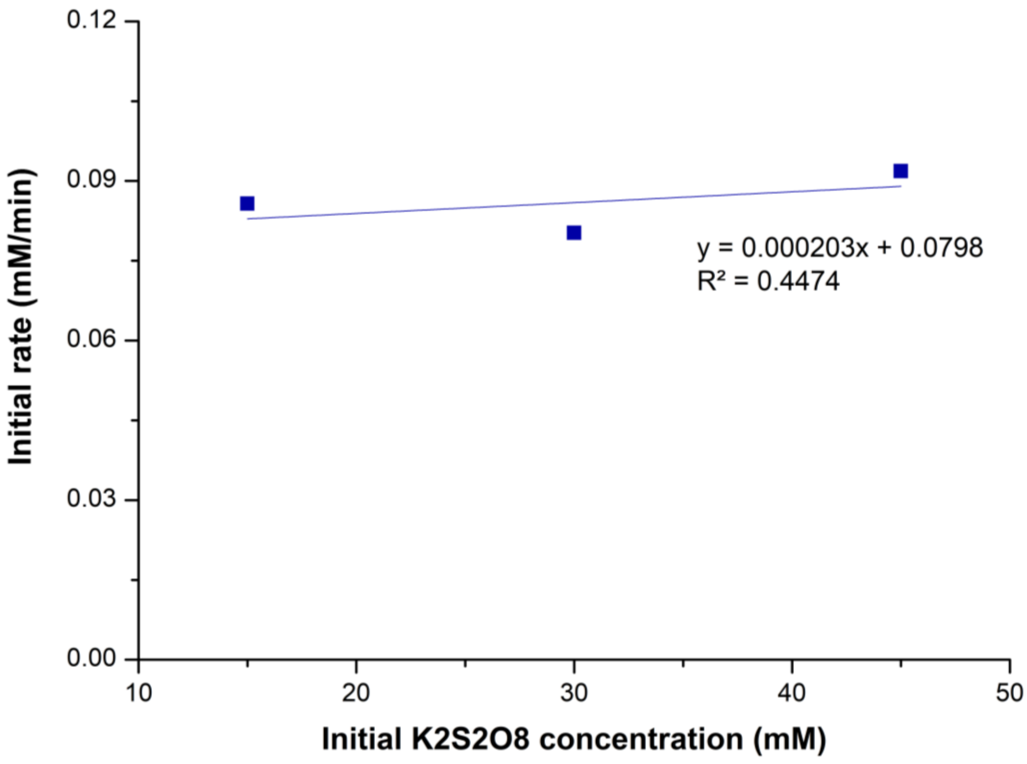

(iv) Initial reaction rate versus TMSOTf concentration

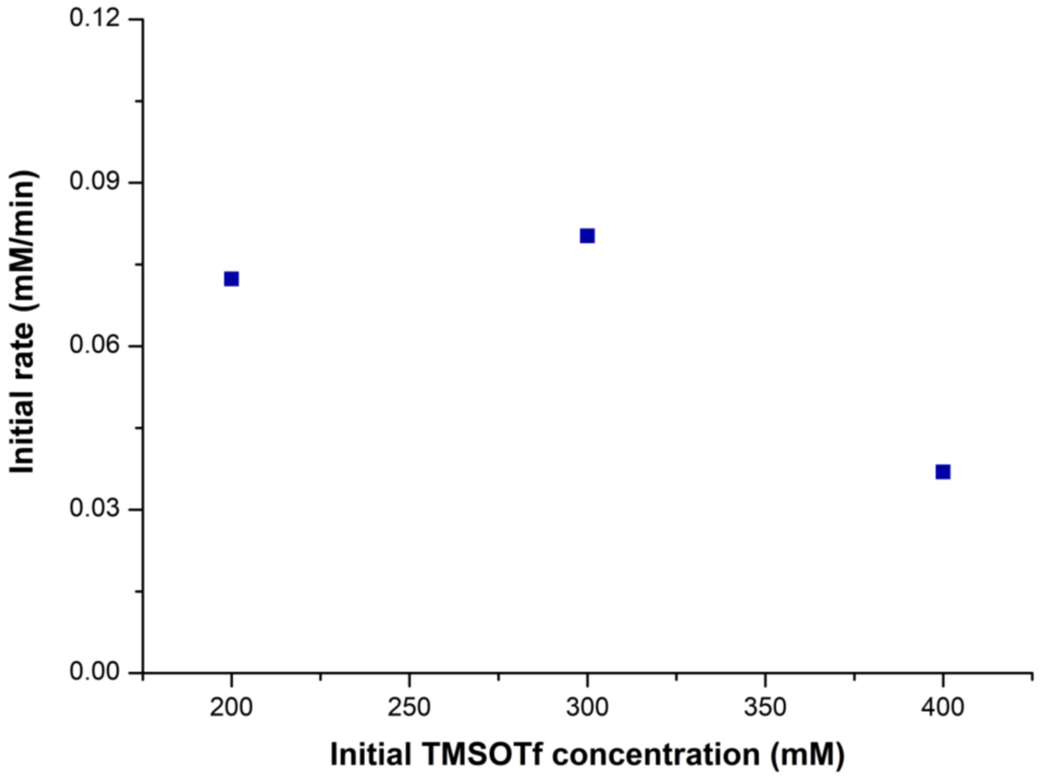

(v) Initial reaction rate versus light intensity

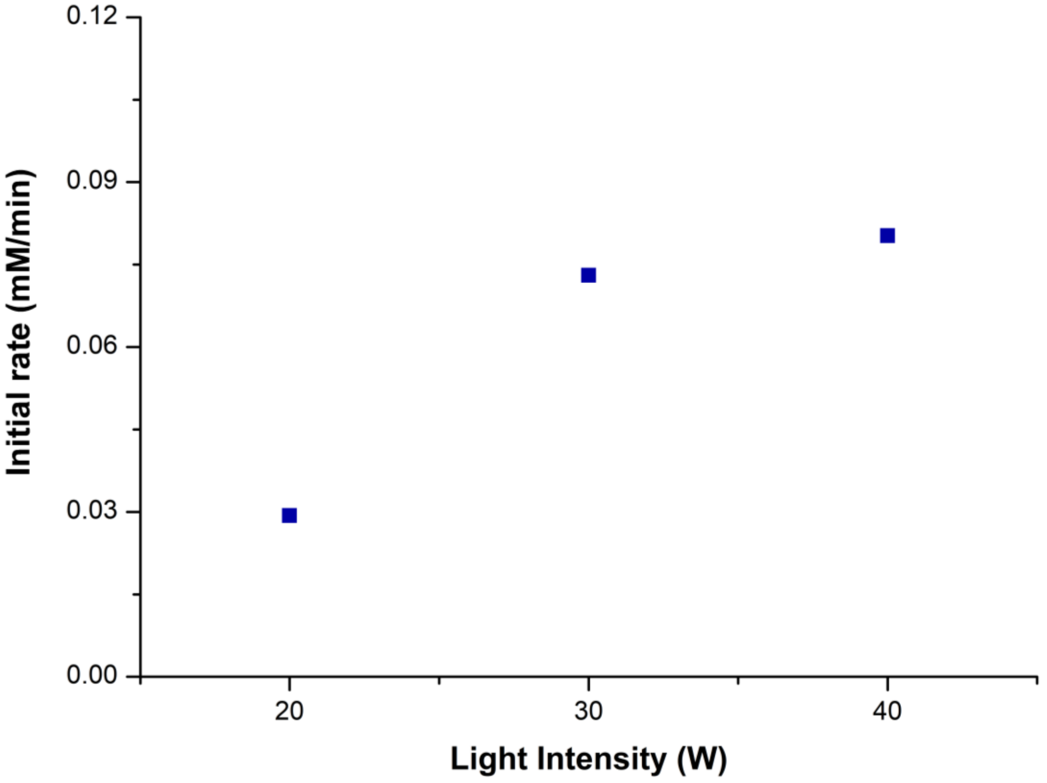

***Notes:** Consistent with the VTNA results, we confirmed that the initial rate was proportional to the iron catalyst concentration and inversely proportional to the concentration of the substrate or intermediate. Other reaction parameters had little effect on the initial rate; however, the reaction was markedly retarded when the light intensity was reduced to half of the standard condition (20 W) or when strongly acidic conditions were generated by using ≥4.0 equiv of TMSOTf. In particular, the strongly acidic conditions are expected to be associated with imine protonation, which is discussed in the next section (**Section 4.J. Evidence for selective protonation of aniline under the reaction conditions**).

J. Evidence for selective protonation of aniline under the reaction conditions

General Procedure for ^{1}H NMR protonation study

Imine intermediate (**4**), aniline (**2**), TMSOTf, and $CF_2HCOONa$ were dissolved in CD_3CN to prepare NMR samples at concentrations suitable for analysis (20 mM with respect to both the imine and aniline), while matching the standard reaction stoichiometry (TMSOTf, 3.0 equiv; $CF_2HCOONa$, 4.0 equiv relative to aniline). The samples were prepared as follows:

Sample 1: Imine (20 mM), aniline (20 mM), $CF_2HCOONa$ (66.6 mM)

Sample 2: Imine (20 mM), aniline (20 mM), $CF_2HCOONa$ (66.6 mM), TMSOTf (50 mM)

Sample 3: Imine (20 mM), aniline (20 mM)

Sample 4: Imine (20 mM), aniline (20 mM), TMSOTf (50 mM)

^{1}H NMR spectra were recorded for the samples listed above. Protonation of the imine and aniline was assessed by comparing Sample 1 with Sample 2, and Sample 3 with Sample 4. The effect of the acid (TMSOTf) was evaluated by comparing Sample 1 with Sample 2 in the absence of $CF_2HCOONa$, and Sample 3 with Sample 4 in the presence of $CF_2HCOONa$.

***Notes:** As shown in Figure 22, in the presence of $CF_2HCOONa$, selective protonation of aniline was evident from the disappearance of the N–H signal. Because these samples were prepared to mimic the standard reaction stoichiometry, this observation supports preferential protonation of aniline over the imine under the standard conditions. By contrast, when the samples were prepared in the absence of $CF_2HCOONa$ (Figure 23), addition of TMSOTf led to protonation of the imine as well. This result suggests formation of a CF_2HCOO^-/CF_2HCOOH buffer system and may account for the pronounced reaction retardation observed when the TMSOTf loading reaches or exceeds that of $CF_2HCOONa$ (≥ 4.0 equiv.) in the preceding experiments.

Below are TLC (hexane/ethyl acetate = 5:1) images of the standard reaction mixture before (left) and after (right) addition of TMSOTf (Figure 21). Upon addition of TMSOTf, the aniline spot (R_f = 0.25) disappeared and a spot that remained at the baseline was observed.

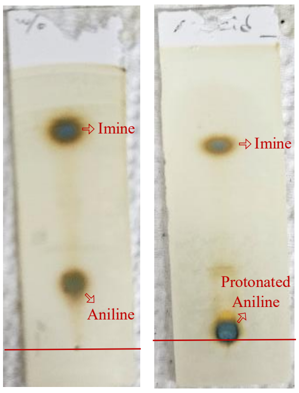

Figure 21. Thin Layer Chromatography of reaction mixture before(left)/after(right) TMSOTf added

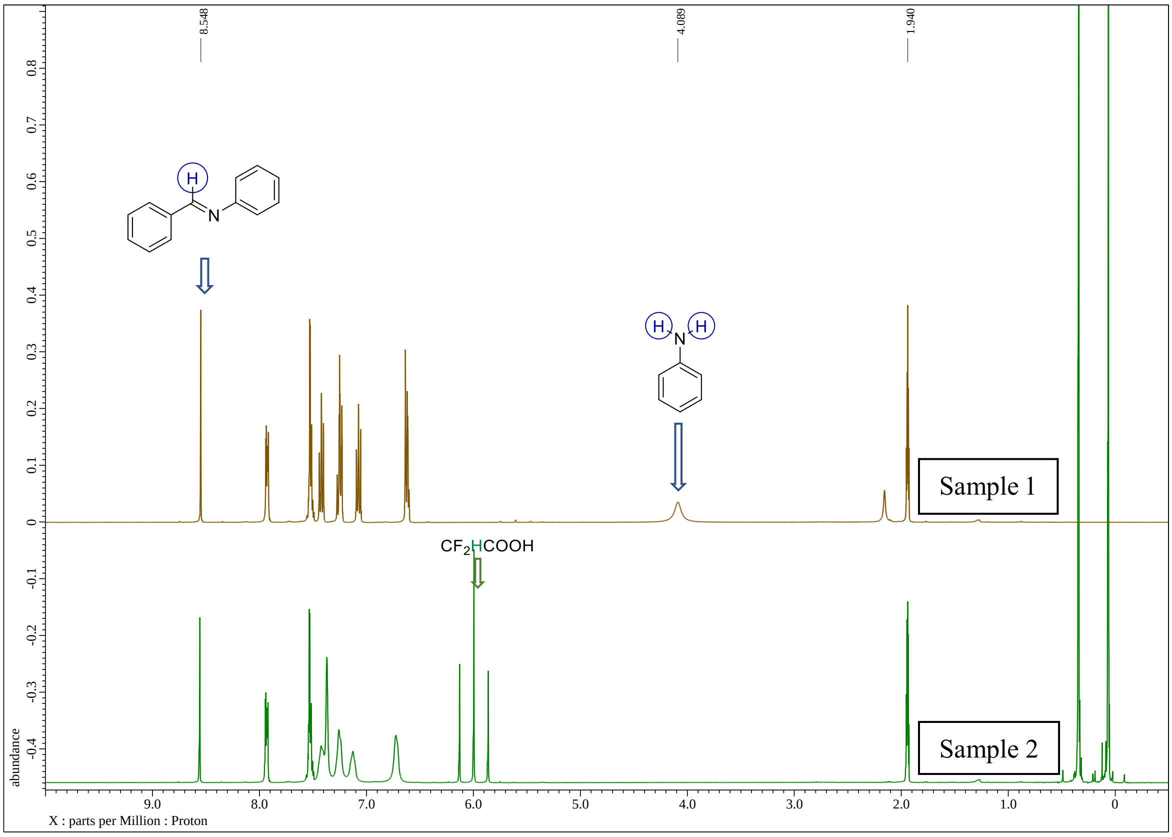

Figure 22. ^{1}H NMR spectra of Sample 1 compared Sample 2

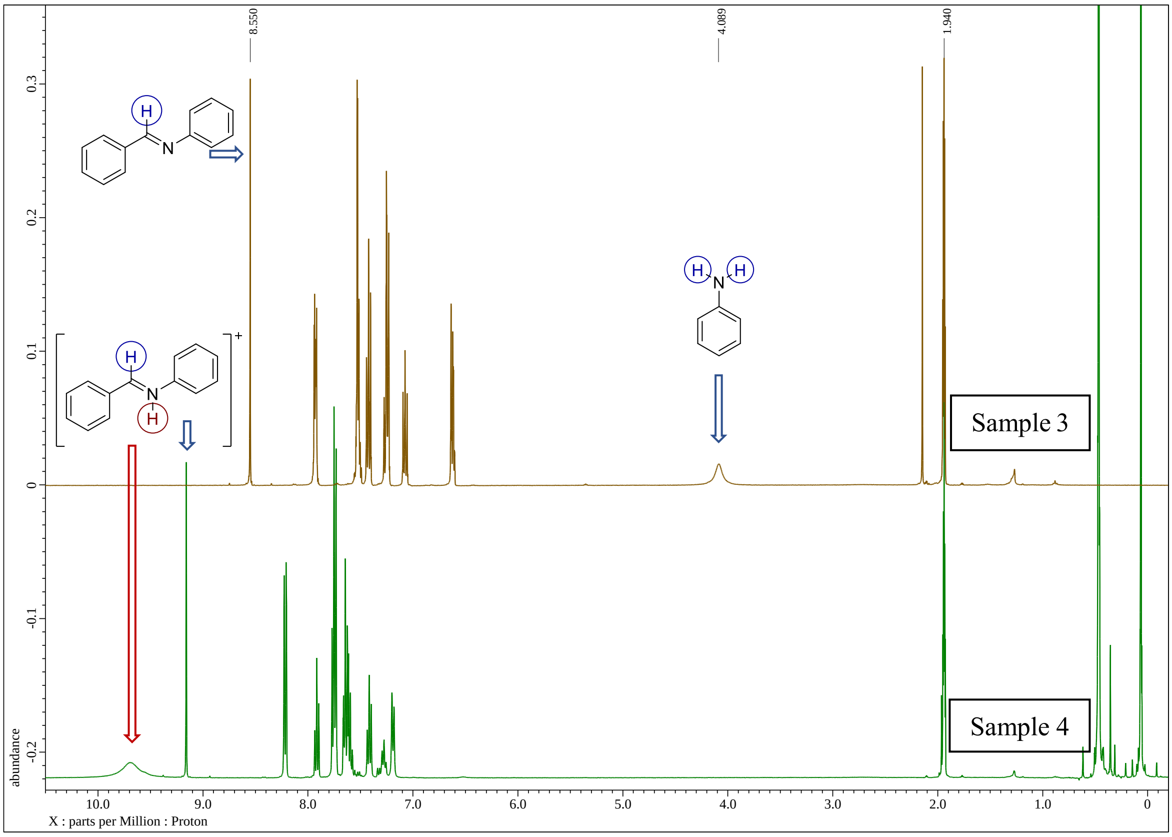

Figure 23. ^{1}H NMR spectra of Sample 3 compared Sample 4

K. NMR Titration Experiment

General Procedure for NMR titration

Imine intermediate (**4**), $Fe(NO_3)_3 \cdot 9H_2O$, and $CF_2HCOONa$ were dissolved in CD_3CN to prepare NMR samples at concentrations suitable for analysis (20 mM with respect to the imine). The stock solutions were prepared as follows:

Solution A: Imine (20 mM) in CD_3CN

Solution B: Imine (20 mM), $Fe(NO_3)_3 \cdot 9H_2O$ (20 mM), and $CF_2HCOONa$ (80 mM) in CD_3CN

Samples 1–6, each containing a different equivalent of the iron complex relative to the imine, were then prepared by mixing solutions A and B according to table below, and ^1H NMR spectra were recorded.

	Solution A	Solution B
Sample 1 (iron complex 0 mol%)	0.60 mL	0 mL
Sample 2 (iron complex 10 mol%)	0.54 mL	0.06 mL
Sample 3 (iron complex 20 mol%)	0.48 mL	0.12 mL
Sample 4 (iron complex 30 mol%)	0.42 mL	0.18 mL
Sample 5 (iron complex 50 mol%)	0.30 mL	0.30 mL
Sample 6 (iron complex 100 mol%)	0 mL	0.60 mL

***Notes:** We observed a shift in the ^1H NMR chemical shift of the imine $C(sp^2)$–H signal, consistent with reversible binding between the imine and the iron complex. Also, the increase in FWHM(Full Width at Half Maximum) further supports binding to the paramagnetic iron (III) complex.

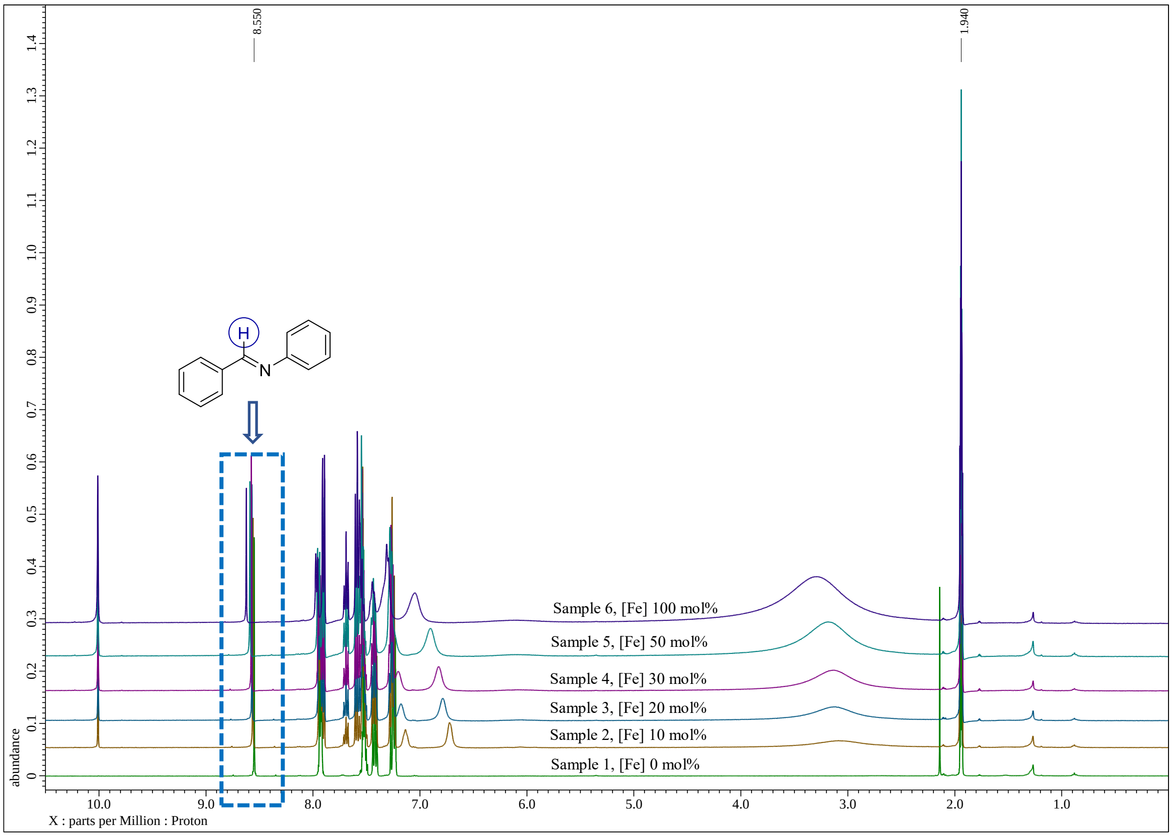

Figure 24. ^{1}H NMR titration of imine (**4**) with iron catalyst

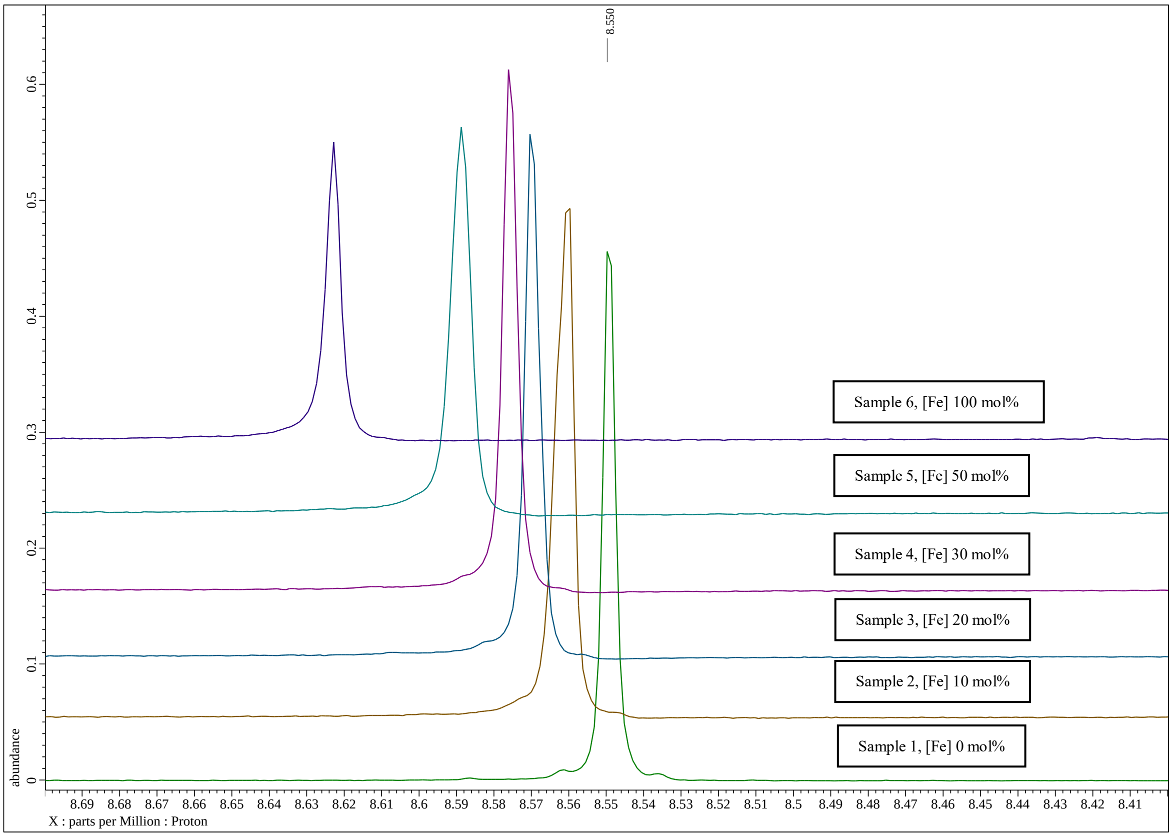

Figure 25. Expanded region of the ^{1}H NMR spectra shown in Figure 24

5. Computational Studies

A. General Computational Information

All DFT computations were carried out using Gaussian 16[9] and GaussView 6.0[10]. Geometry optimizations were performed with the (U)ωB97X-D[11] functional, and the def2-TZVP[12] basis set was used for all atoms. Vibrational frequency calculations were performed at the same level of theory as the geometry optimizations to confirm the stationary points (no imaginary frequencies for local minima and only one imaginary frequency for transition states) and to provide the thermal corrections for Gibbs free energy determinations. For transition structures, an intrinsic reaction coordinate (IRC) calculations were performed in order to confirm intermediates along the reaction pathway. The single-point energy calculations were computed on the optimized geometries of intermediates and transition structures, with (U)ωB97X-D[11] functional and def2-TZVP[12] basis set for all atoms including solvation energy corrections using the SMD[13] model (solvent = acetonitrile). Three-dimensional molecular structures were visualized using GaussView 6.0[9] and CYLview20[14].

B. Energy components from DFT calculations

	E(sol) (SCF, Hartree)	Thermal correction (Hartree)	G(sol) (kcal/mol)
**1a (Benzaldehyde)**	-345.59164989	0.080155	-345.51149489
**2a (Aniline)**	-287.62537977	0.088919	-287.53646077
**CF_2H radical**	-238.35449889	-0.005760	-238.36025889
**4 (Imine)**	-556.76513971	0.166512	-556.59862771
**TS1**	-795.1175978	0.179435	-794.93816281
**Int1**	-795.1722406	0.184386	-794.98785460
**3 (Product)**	-795.82746383	0.199316	-795.62814783

C. DFT Energy Landscape

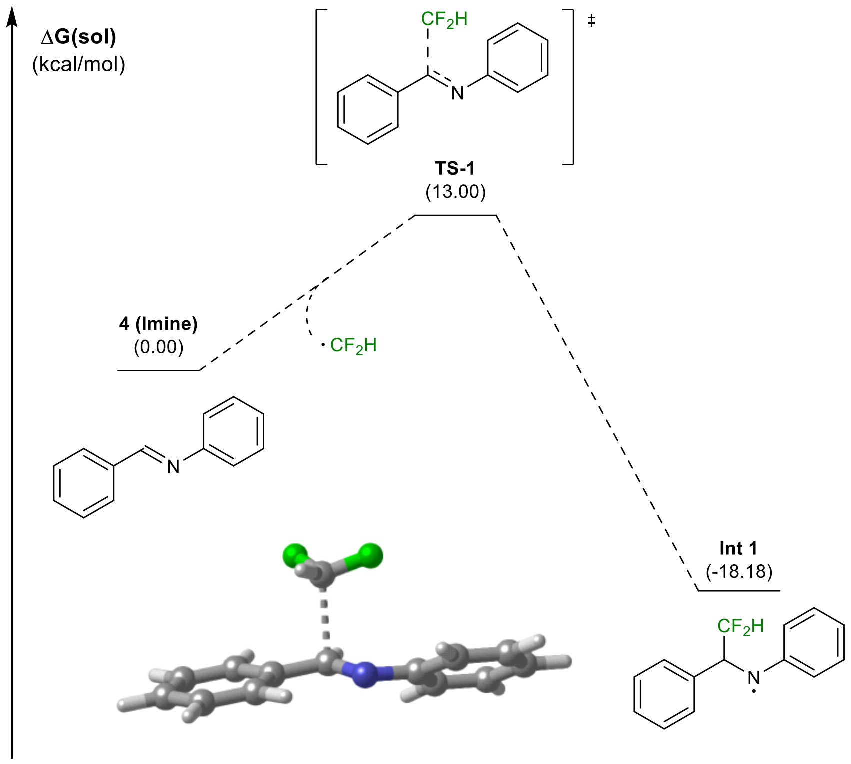

D. Cartesian Coordinates of all optimized geometries

**1a**, Charge 0, multiplicity 1			
C	-1.906413	0.621061	0.216166
C	-0.679295	0.404230	-0.401002
C	0.179970	1.463750	-0.621942
C	-0.186822	2.747387	-0.224816
C	-1.414338	2.959741	0.391629
C	-2.275822	1.897091	0.613085
H	-2.577161	-0.211535	0.387653
H	-0.398192	-0.594968	-0.707964
H	1.140420	1.320002	-1.100351
H	-1.692974	3.962154	0.697898
H	-3.231700	2.062012	1.093062
C	0.720061	3.894597	-0.452165
H	0.327608	4.870150	-0.096278
O	1.800545	3.823335	-0.974806

**2a**, Charge 0, multiplicity 1			
C	-1.255516	1.052302	-0.018410
C	0.126428	1.124026	-0.112885
C	0.776892	2.345082	-0.073332
C	0.051478	3.529501	0.061451
C	-1.338614	3.453758	0.157384
C	-1.979420	2.227667	0.116823
H	-1.760149	0.095976	-0.050080
H	0.709569	0.217490	-0.219208
H	1.858020	2.386021	-0.143333
H	-1.916648	4.364233	0.268343
H	-3.059382	2.192735	0.191876
N	0.703417	4.754192	0.154112
H	1.622988	4.785073	-0.251777
H	0.149808	5.557194	-0.090860

CF_2H radical, Charge 0, multiplicity 2			
C	-0.316167	0.285770	0.055001
H	-1.402328	0.328900	-0.023299
F	0.179316	0.949965	1.084957
F	0.183402	-0.936207	-0.014389

**4**, Charge 0, multiplicity 1			
C	-1.717906	-0.343727	0.032682
C	-0.398095	-0.300065	0.452203
C	0.278808	0.909422	0.471498
C	-0.356809	2.081379	0.072949
C	-1.685437	2.029387	-0.347387
C	-2.359358	0.823219	-0.366952
H	-2.250020	-1.286569	0.016157
H	0.104383	-1.206775	0.763750
H	1.311955	0.946841	0.798464
H	-2.170169	2.946668	-0.655134
H	-3.390748	0.787355	-0.694172
C	0.386972	3.346795	0.105206
H	1.423811	3.281530	0.460289
N	-0.116646	4.448526	-0.262464
C	0.642766	5.624389	-0.157416

C	0.622845	6.520571	-1.224117
C	1.370688	5.944566	0.987454
C	1.354435	7.693039	-1.162861
H	0.033482	6.275250	-2.098166
C	2.088016	7.129266	1.048513
H	1.347622	5.276705	1.839797
C	2.090471	8.003246	-0.026965
H	1.343059	8.375203	-2.003699
H	2.642452	7.372326	1.946406
H	2.651032	8.927714	0.023568

TS1, Charge 0, multiplicity 2			
C	-2.985702	-3.936466	0.090801
C	-1.796211	-3.889829	0.800640
C	-1.025273	-2.738263	0.783919
C	-1.437439	-1.622844	0.061988
C	-2.635110	-1.675174	-0.647537
C	-3.402241	-2.826285	-0.632883
H	-3.589654	-4.834944	0.102110
H	-1.467380	-4.750920	1.368324
H	-0.092050	-2.703960	1.334229
H	-2.952870	-0.799012	-1.197614
H	-4.333208	-2.859059	-1.184800
C	-0.598519	-0.404547	0.057479
H	0.265064	-0.427300	0.732402
N	-1.044451	0.704994	-0.438346
C	-0.338644	1.890524	-0.238171
C	-0.381475	2.845113	-1.256609
C	0.363163	2.188771	0.933858
C	0.297002	4.042658	-1.128067
H	-0.947374	2.616494	-2.150427
C	1.026311	3.397116	1.063139
H	0.359755	1.488898	1.759967
C	1.006117	4.324484	0.031891
H	0.266285	4.765651	-1.933539
H	1.556521	3.619629	1.980794
H	1.527134	5.267291	0.137384
C	0.816850	-1.087254	-1.545201

H	0.141177	-1.438601	-2.323386
F	1.587463	-2.037481	-1.035939
F	1.540031	-0.039533	-1.890254

Int1, Charge 0, multiplicity 2			
C	-0.998884	1.862563	1.077613
C	-0.465372	0.971827	0.155189
C	-0.176689	1.417691	-1.129785
C	-0.418916	2.735502	-1.483526
C	-0.950647	3.620295	-0.557774
C	-1.240756	3.180642	0.724624
H	-1.233635	1.519653	2.078533
H	0.249654	0.730716	-1.849987
H	-0.188877	3.072891	-2.486255
H	-1.137148	4.650140	-0.834525
H	-1.656643	3.865094	1.453100
C	-0.221972	-0.476355	0.542848
H	-0.201834	-0.553695	1.637352
N	0.967089	-1.012804	-0.068559
C	2.160031	-0.710916	0.451828
C	3.289282	-1.297928	-0.182223
C	2.398055	0.142448	1.565943
C	4.563857	-1.058157	0.271696
H	3.100855	-1.944114	-1.029429
C	3.681137	0.375359	2.003284
H	1.570377	0.629752	2.062023
C	4.769394	-0.220807	1.367284
H	5.409662	-1.519174	-0.222059
H	3.847693	1.031860	2.847957
H	5.773510	-0.030008	1.723462
C	-1.381633	-1.356451	0.083900
H	-1.470164	-1.395679	-1.004815
F	-1.211693	-2.618761	0.547392
F	-2.550581	-0.899316	0.602529

**3**, Charge 0, multiplicity 1			
C	-0.713948	3.991074	1.917771
C	-1.428479	5.176185	2.002087

C	-1.721994	5.890862	0.852210
C	-1.311305	5.430075	-0.392434
C	-0.592411	4.245388	-0.468182
C	-0.295460	3.528593	0.679855
H	-0.478567	3.432769	2.814890
H	-1.754355	5.547665	2.965328
H	-2.279534	6.817758	0.922526
H	-0.248875	3.882762	-1.428551
H	0.268708	2.607548	0.606876
C	-1.659057	6.223183	-1.641631
H	-1.748365	7.278266	-1.364867
C	-3.030723	5.855673	-2.201568
H	-3.294734	6.480511	-3.058366
N	-0.731894	6.120271	-2.739051
H	-0.836588	5.285553	-3.292048
C	0.579621	6.575317	-2.642184
C	1.494189	6.211189	-3.634575
C	1.011231	7.408167	-1.610790
C	2.794817	6.674402	-3.599080
H	1.171267	5.565129	-4.443464
C	2.318232	7.871850	-1.589815
H	0.340963	7.685678	-0.808991
C	3.219519	7.513298	-2.577134
H	3.484130	6.377912	-4.379958
H	2.632520	8.516587	-0.778414
H	4.238427	7.875621	-2.550639
F	-3.041892	4.558323	-2.616609
F	-3.988609	5.988092	-1.257323

6. References

(1) Bian, K.-J.; Lu, Y.-C.; Nemoto, D., Jr.; Kao, S.-C.; Chen, X.; West, J. G. Photocatalytic Hydrofluoroalkylation of Alkenes with Carboxylic Acids. *Nat. Chem.* **2023**, *15*, 1683–1692.

(2) Qi, X.-K.; Yao, L.-J.; Zheng, M.-J.; Zhao, L.; Yang, C.; Guo, L.; Xia, W. Photoinduced Hydrodifluoromethylation and Hydromethylation of Alkenes Enabled by Ligand-to-Iron Charge Transfer Mediated Decarboxylation. *ACS Catal.* **2024**, *14*, 1300–1310.

(3) Hatchard, C. G.; Parker, C. A. A New Sensitive Chemical Actinometer. II. Potassium Ferrioxalate as a Standard Chemical Actinometer. *Proc. R. Soc. London, Ser. A.* **1956**, *235*, 518–536.

(4) Choi, G. J.; Zhu, Q.; Miller, D. C.; Gu, C. J.; Knowles, R. R. Catalytic alkylation of remote C–H bonds enabled by proton-coupled electron transfer. *Nature.* **2016**, *539*, 268–271.

(5) Kim, S.; Oh, H.; Dong, W.; Majhi, J.; Sharique, M.; Matsuo, B.; Keess, S.; Molander, G. A. Metal-Free Photoinduced Acylboration of [1.1.1]Propellane via Energy Transfer Catalysis. *ACS Catal.* **2023**, *13*, 9542–9549.

(6) Kim, D.; You, J.; Lee, D. H.; Hong, H.; Kim, D.; Park, Y. Photocatalytic furan-to-pyrrole conversion. *Science.* **2024**, *386*, 99–105.

(7) Hong, Y.; Park, C.; Jang, J.; Oh, M.; Kim, D.; Lee, S.; Hong, S. Y. Cooperative Organosulfur/Photoredox Catalysis Enables Radical–Polar Crossover C(sp3)–N Coupling via Inner-Sphere Electron Shuttling. *J. Am. Chem. Soc.* **2025**, *147*, 19583–19594.

(8) Burés, J. Variable Time Normalization Analysis: General Graphical Elucidation of Reaction Orders from Concentration Profiles. *Angew. Chem. Int. Ed.* **2016**, *55*, 16084–16087.

(9) *Gaussian 16, Revision C.02*, M. J. Frisch, G. W. Trucks, H. B. Schlegel, G. E. Scuseria, M. A. Robb, J. R. Cheeseman, G. Scalmani, V. Barone, G. A. Petersson, H. Nakatsuji, X. Li, M. Caricato, A. V. Marenich, J. Bloino, B. G. Janesko, R. Gomperts, B. Mennucci, H. P. Hratchian, J. V. Ortiz, A. F. Izmaylov, J. L. Sonnenberg, D. Williams-Young, F. Ding, F. Lipparini, F. Egidi, J. Goings, B. Peng, A. Petrone, T. Henderson, D. Ranasinghe, V. G. Zakrzewski, J. Gao, N. Rega, G. Zheng, W. Liang, M. Hada, M. Ehara, K. Toyota, R. Fukuda, J. Hasegawa, M. Ishida, T. Nakajima, Y. Honda, O. Kitao, H. Nakai, T. Vreven, K. Throssell, J. A. Montgomery, Jr., J. E. Peralta, F. Ogliaro, M. J. Bearpark, J. J. Heyd, E. N. Brothers, K. N. Kudin, V. N. Staroverov, T. A. Keith, R. Kobayashi, J. Normand, K. Raghavachari, A. P. Rendell, J. C. Burant, S. S. Iyengar, J. Tomasi, M. Cossi, J. M. Millam, M. Klene, C. Adamo, R. Cammi, J. W. Ochterski, R. L. Martin, K. Morokuma, O. Farkas, J. B. Foresman and D. J. Fox, *Gaussian, Inc.*, Wallingford CT, **2016**.

(10) *GaussView, Version 6*, R. Dennington, T. A. Keith and J. M. Millam, Semichem Inc., Shawnee Mission, KS, **2016**.

(11) Chai, J.-D.; Head-Gordon, M. Long-range corrected hybrid density functionals with damped atom–atom dispersion corrections. *Phys. Chem. Chem. Phys.* **2008**, *10*, 6615–6620.

(12) Weigend, F.; Ahlrichs, R. Balanced basis sets of split valence, triple zeta valence and quadruple zeta valence quality for H to Rn: Design and assessment of accuracy. *Phys. Chem. Chem. Phys*. **2005**, *7*, 3297–3305.

(13) A. V. Marenich, C. J. Cramer and D. G. Truhlar, Universal Solvation Model Based on Solute Electron Density and on a Continuum Model of the Solvent Defined by the Bulk Dielectric Constant and Atomic Surface Tensions, *J. Phys. Chem. B*, **2009**, *113*, 6378–6396.

(14) *CYLview20*, Legault, C. Y. Université de Sherbrooke, **2020** (http://www.cylview.org)

7. NMR Spectra Copies

^{1}H NMR spectrum of **3aa** in $CDCl_3$. (400 MHz)

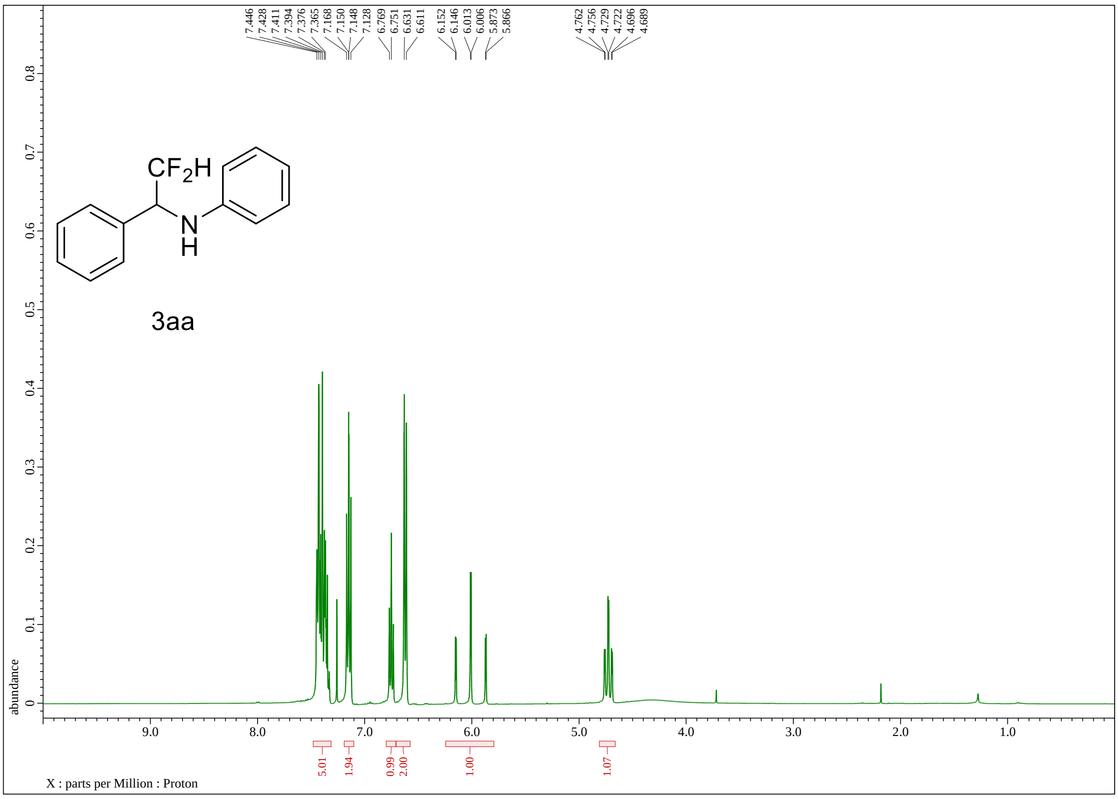

^{13}C NMR spectrum of **3aa** in $CDCl_3$. (100 MHz)

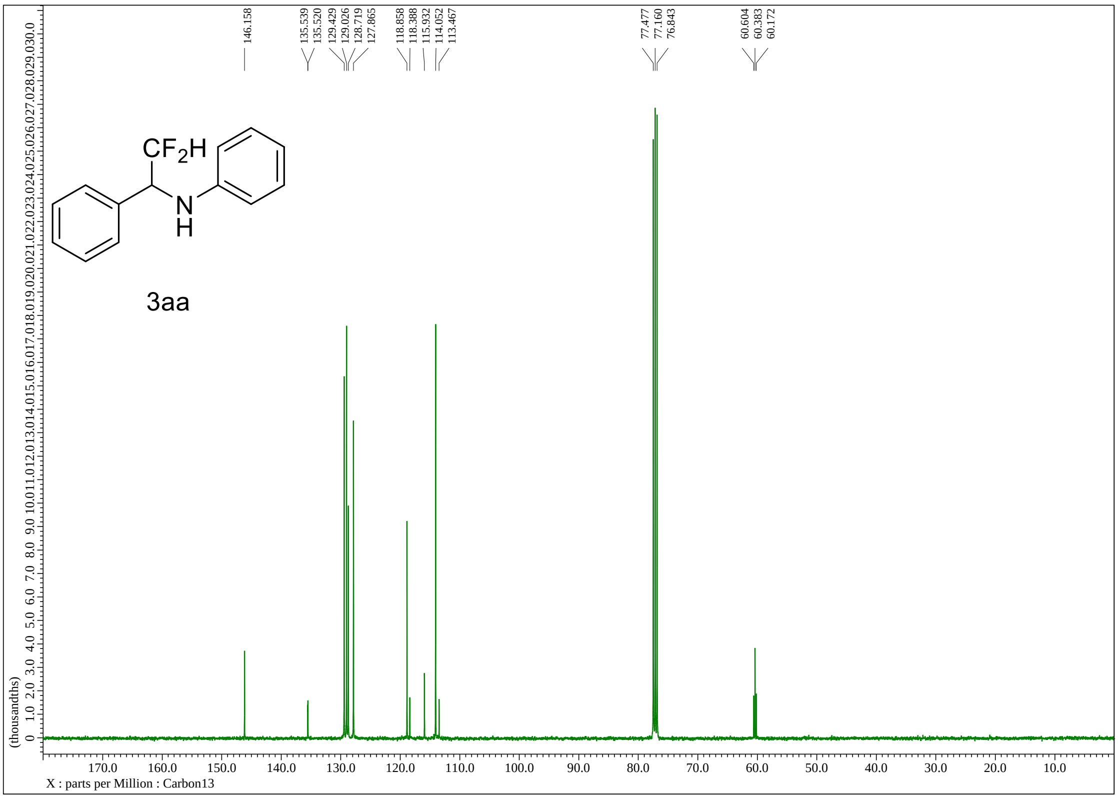

^{19}F NMR spectrum of **3aa** in $CDCl_3$. (376 MHz)

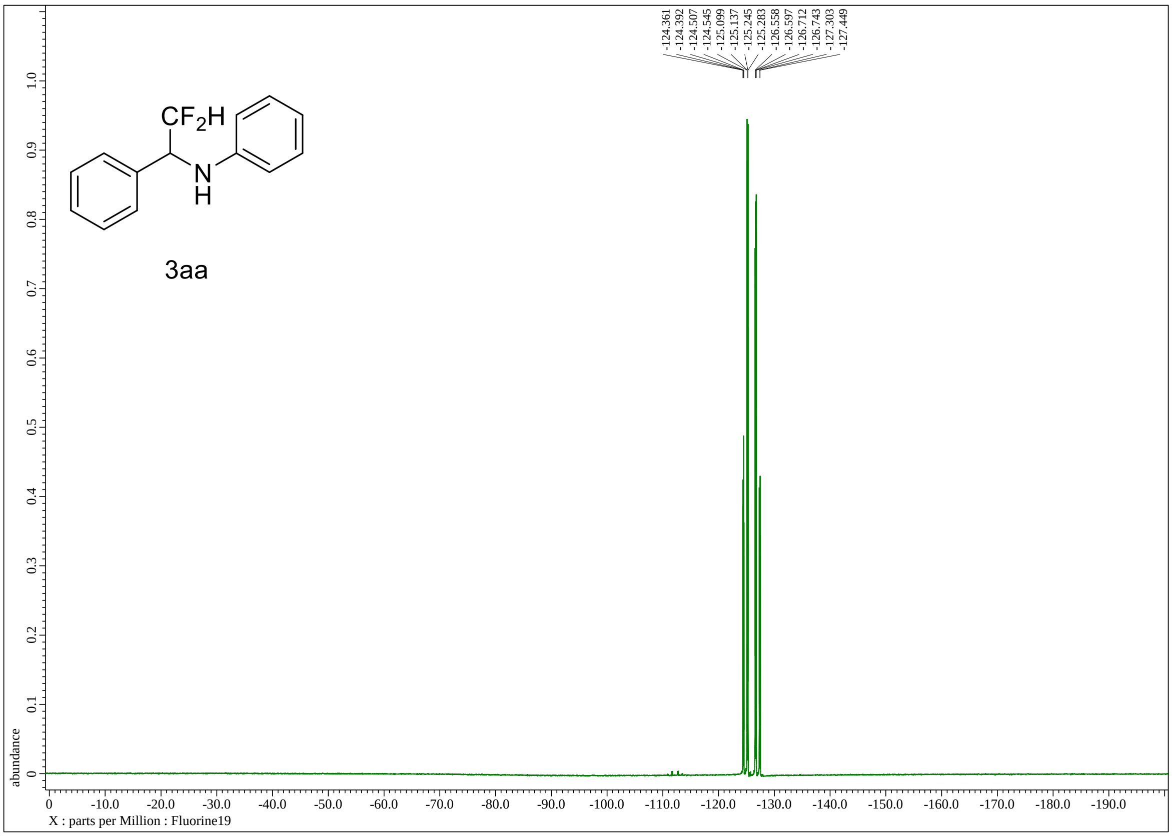

^{1}H NMR spectrum of **3ba** in $CDCl_3$. (400 MHz)

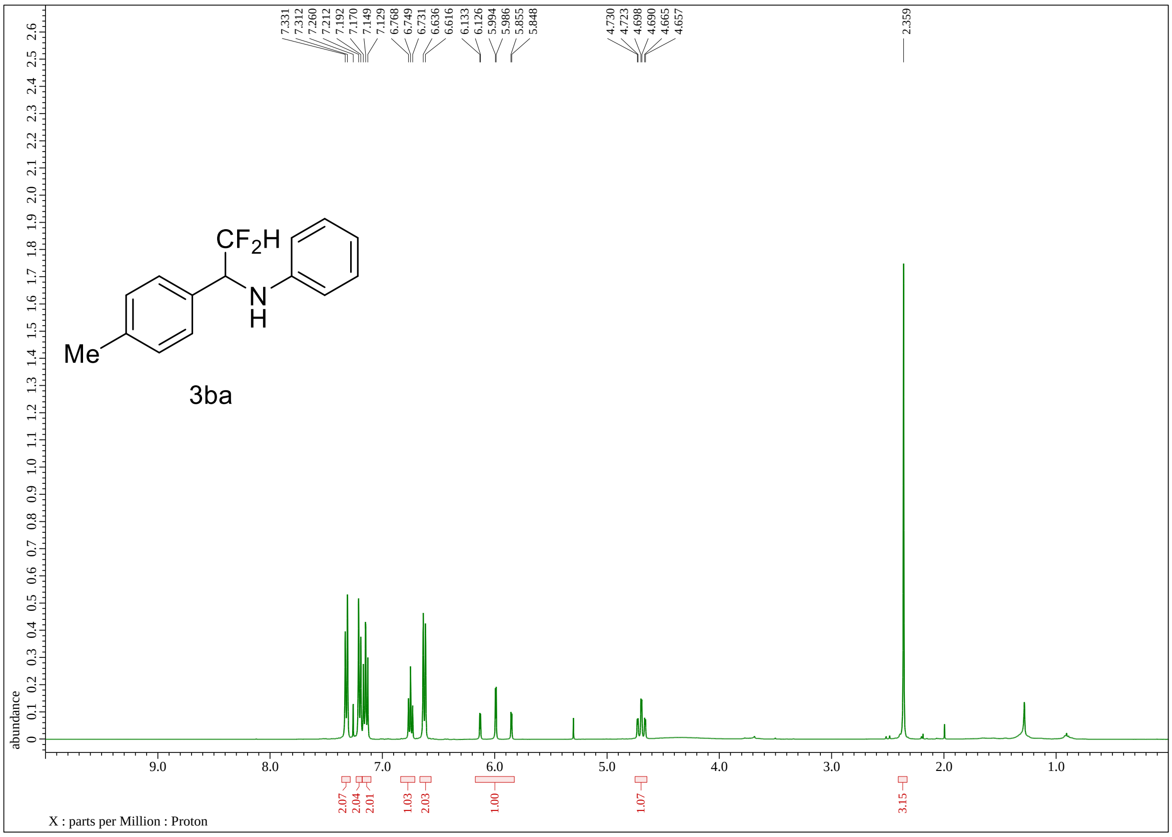

^{13}C NMR spectrum of **3ba** in $CDCl_3$. (100 MHz)

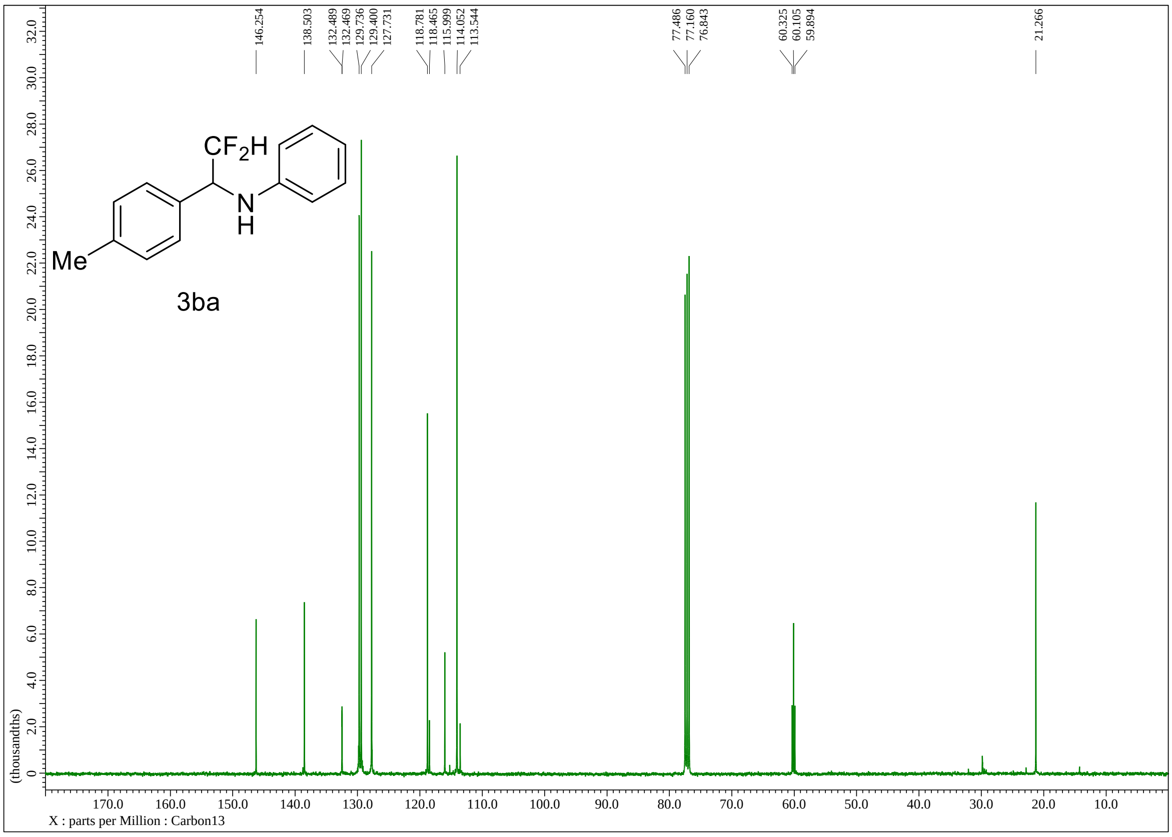

^{19}F NMR spectrum of **3ba** in $CDCl_3$. (376 MHz)

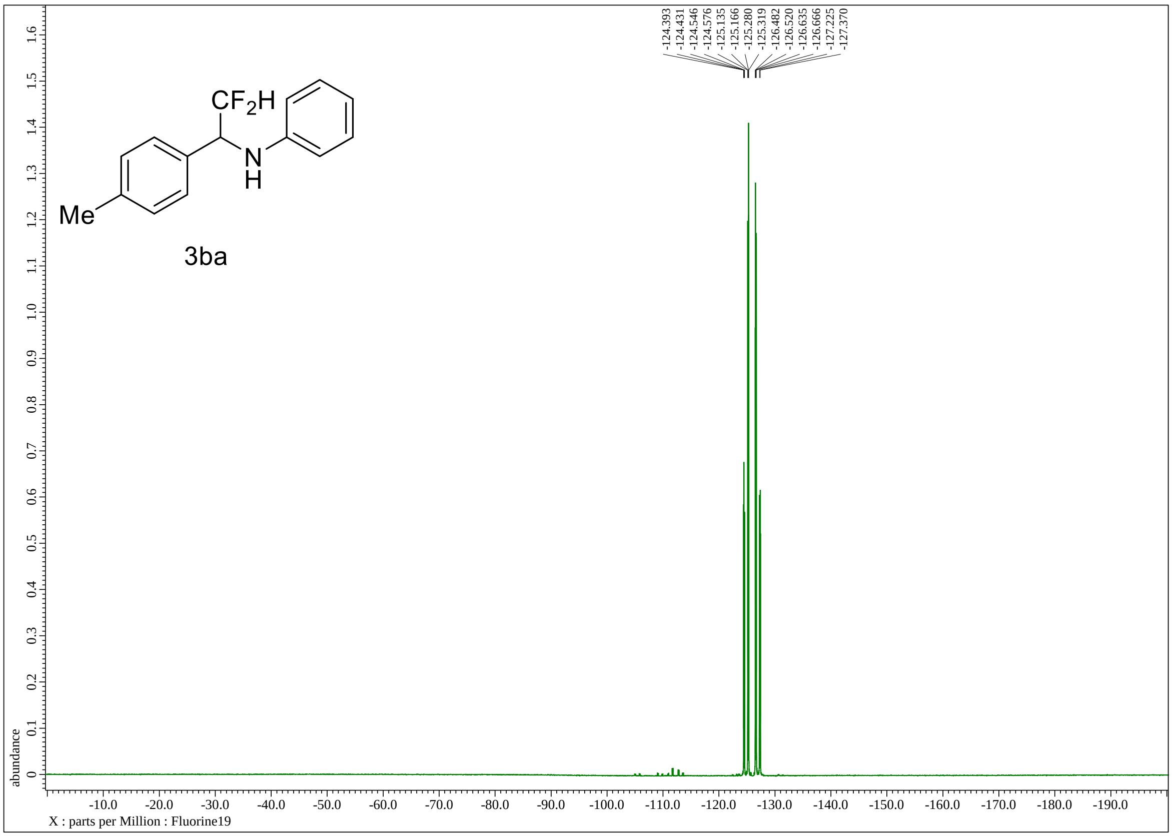

^{1}H NMR spectrum of **3ca** in $CDCl_3$. (400 MHz)

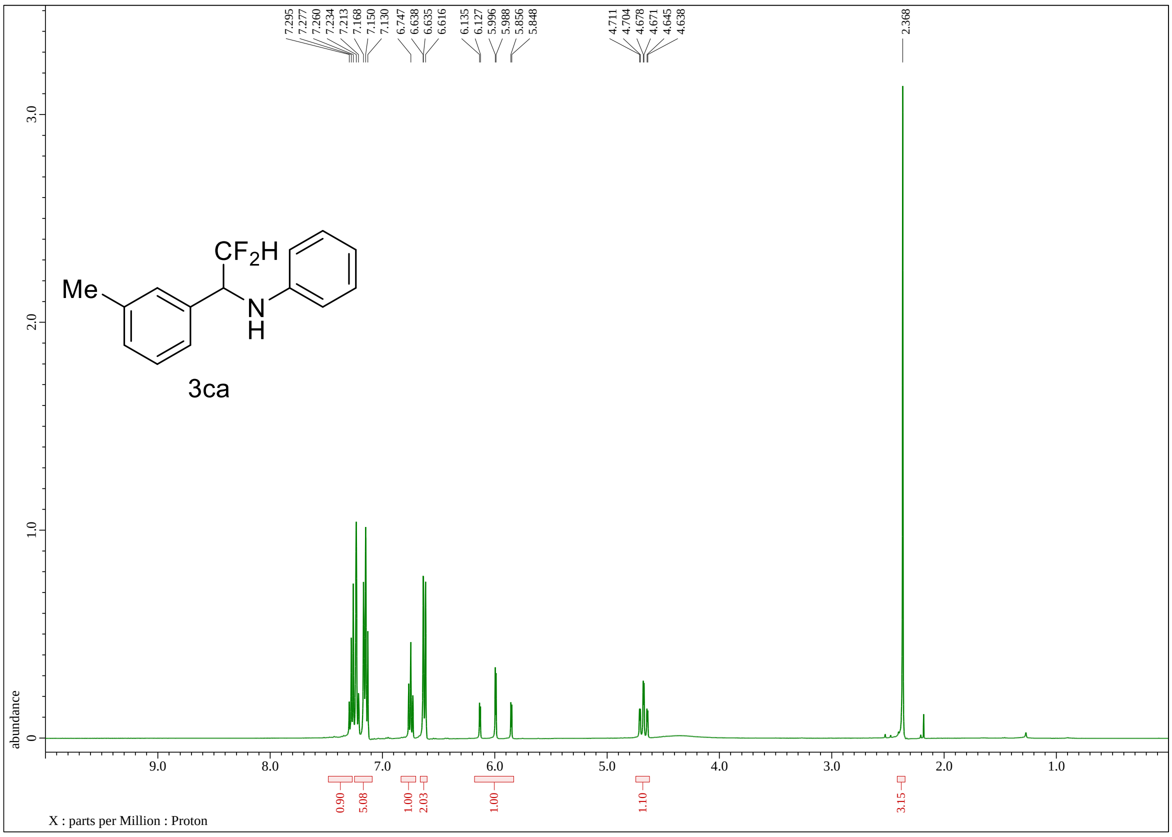

^{13}C NMR spectrum of **3ca** in $CDCl_3$. (100 MHz)

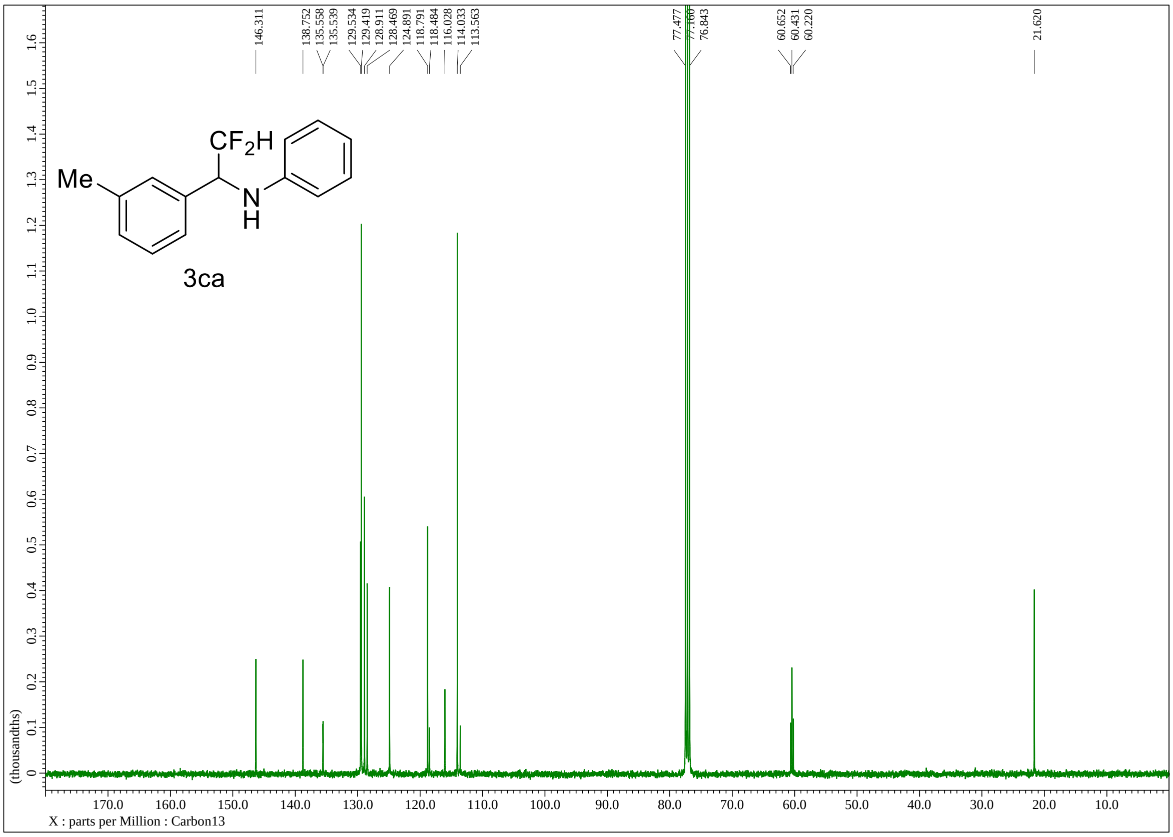

^{19}F NMR spectrum of **3ca** in $CDCl_3$. (376 MHz)

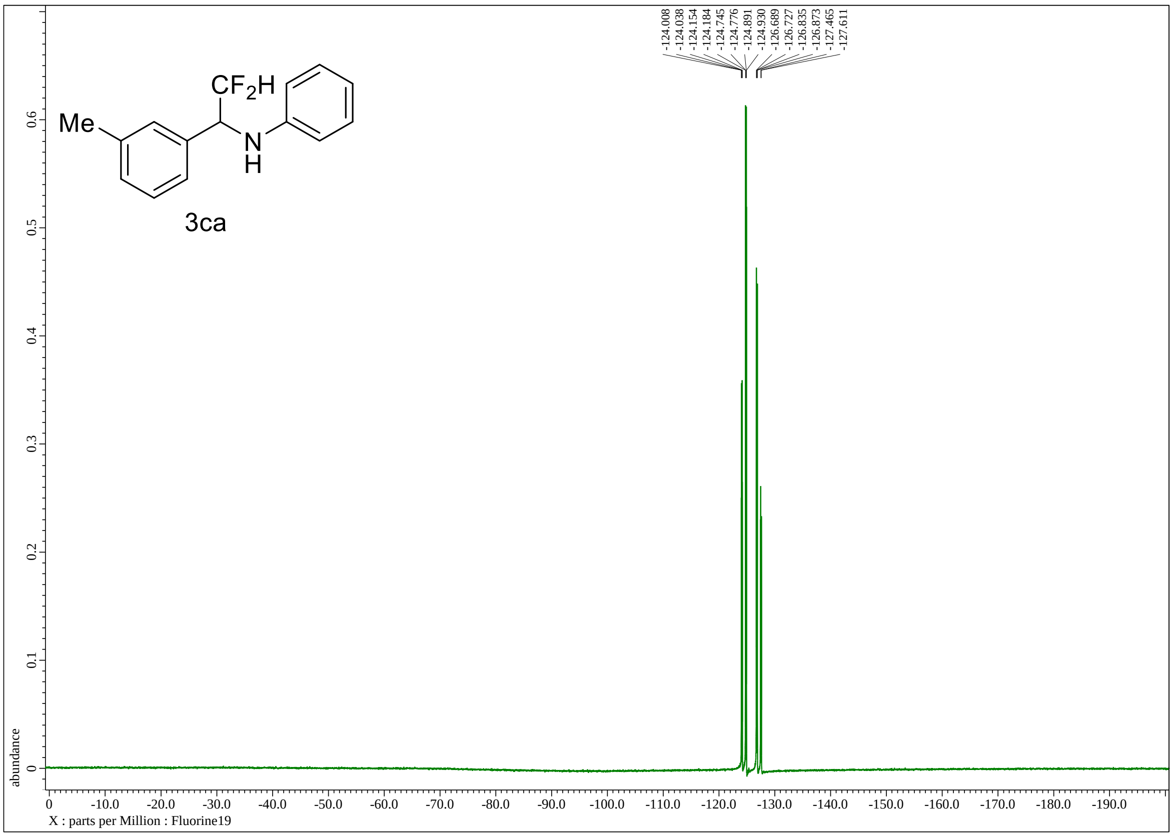

^{1}H NMR spectrum of **3da** in $CDCl_3$. (400 MHz)

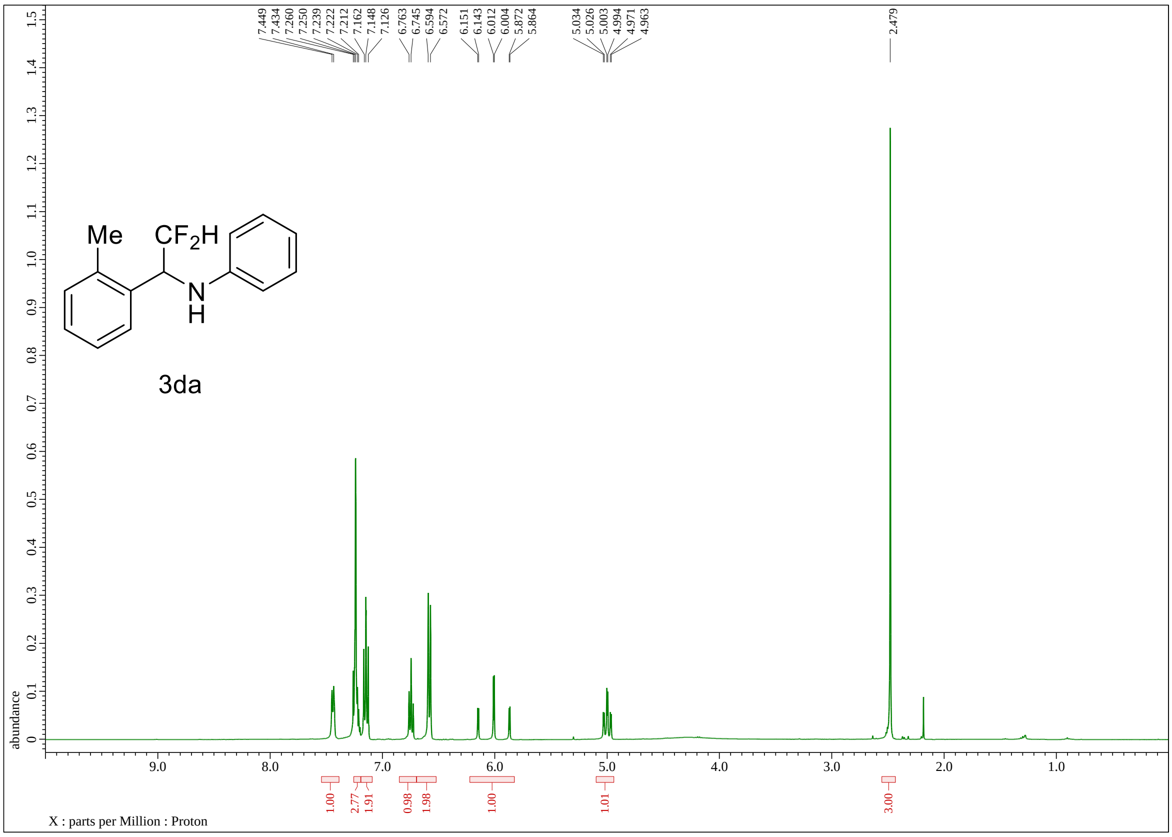

^{13}C NMR spectrum of **3da** in $CDCl_3$. (100 MHz)

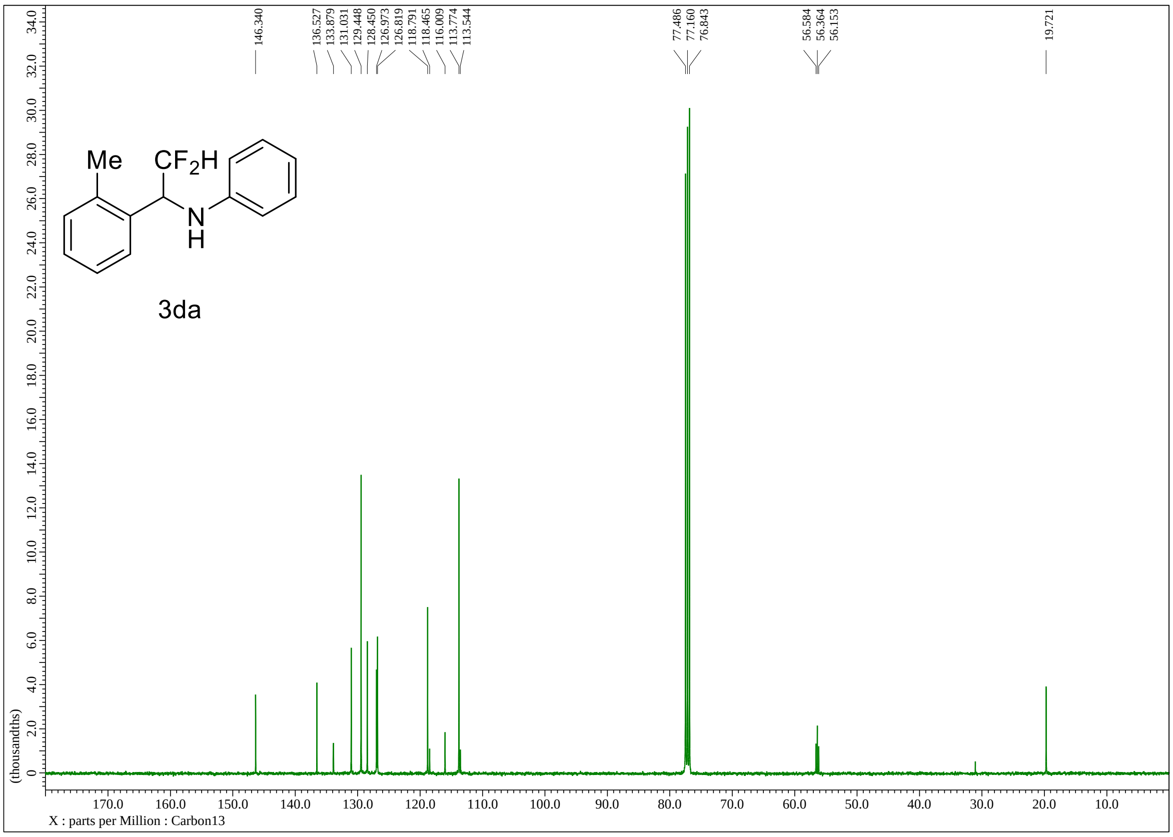

^{19}F NMR spectrum of **3da** in $CDCl_3$. (376 MHz)

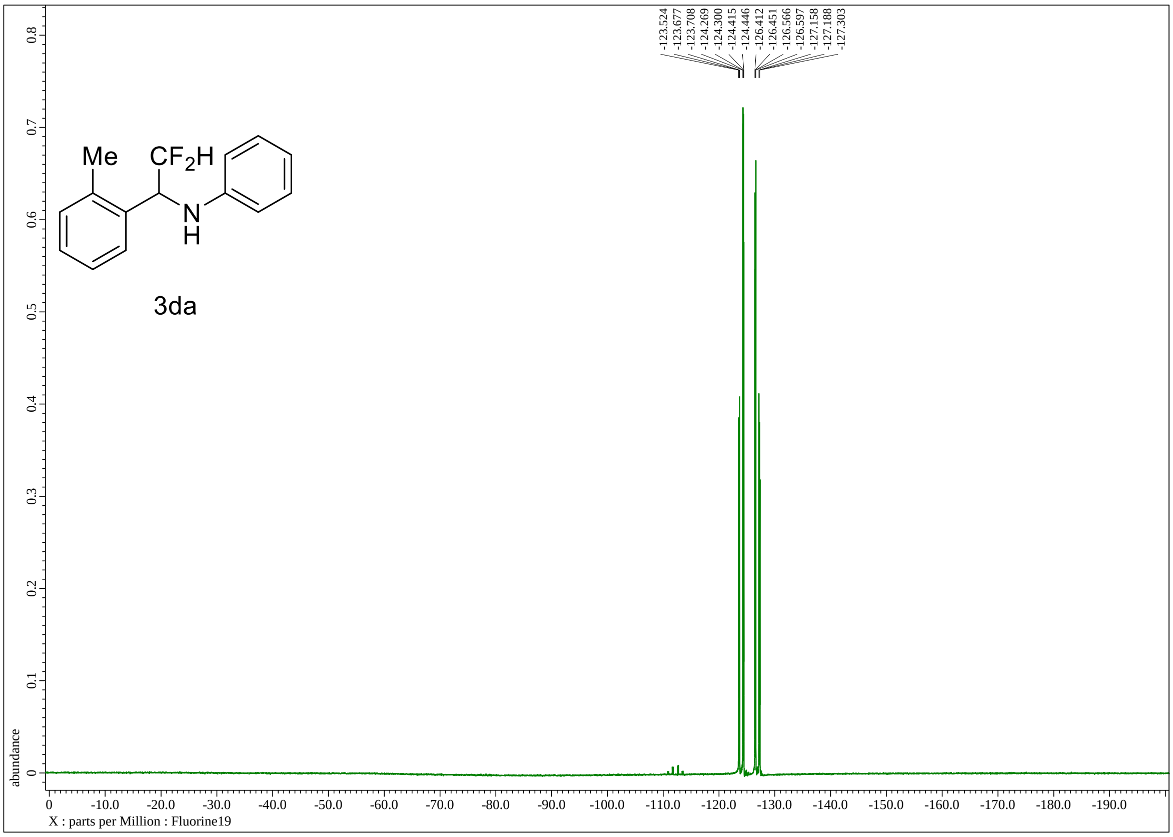

^{1}H NMR spectrum of **3ea** in $CDCl_3$. (400 MHz)

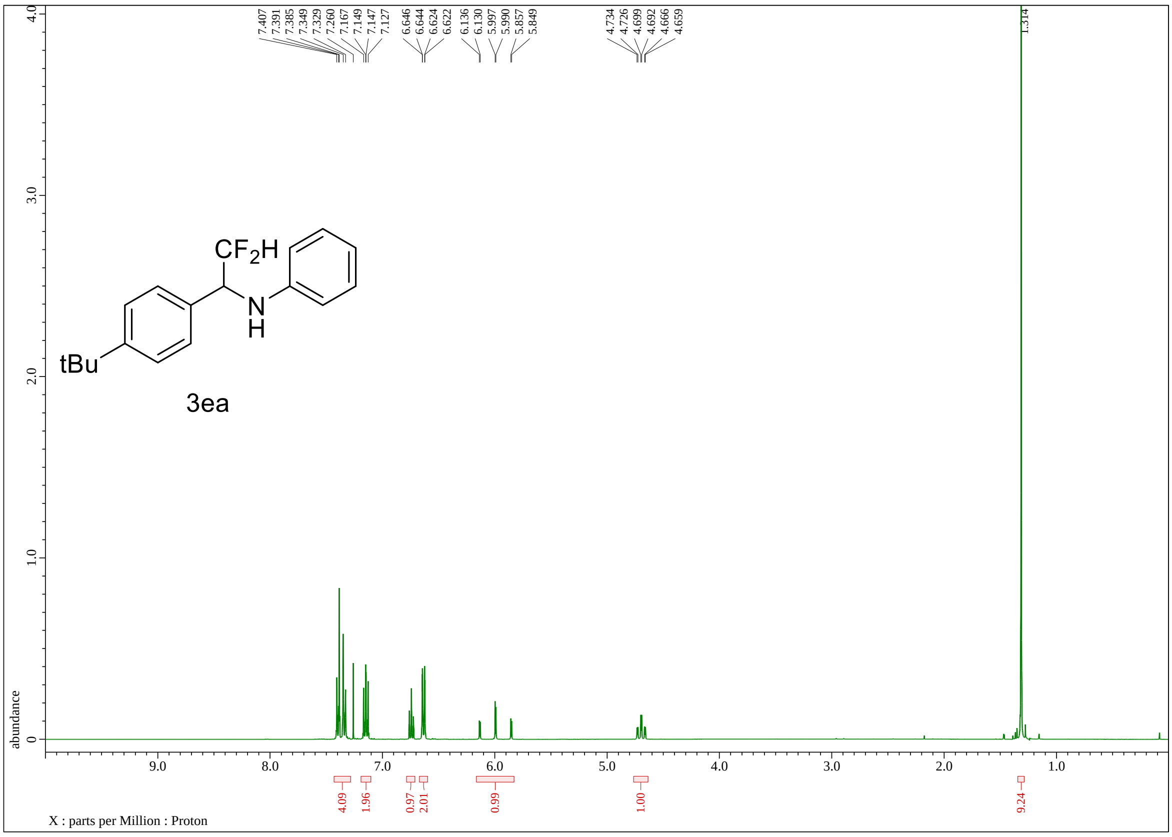

^{13}C NMR spectrum of **3ea** in $CDCl_3$. (100 MHz)

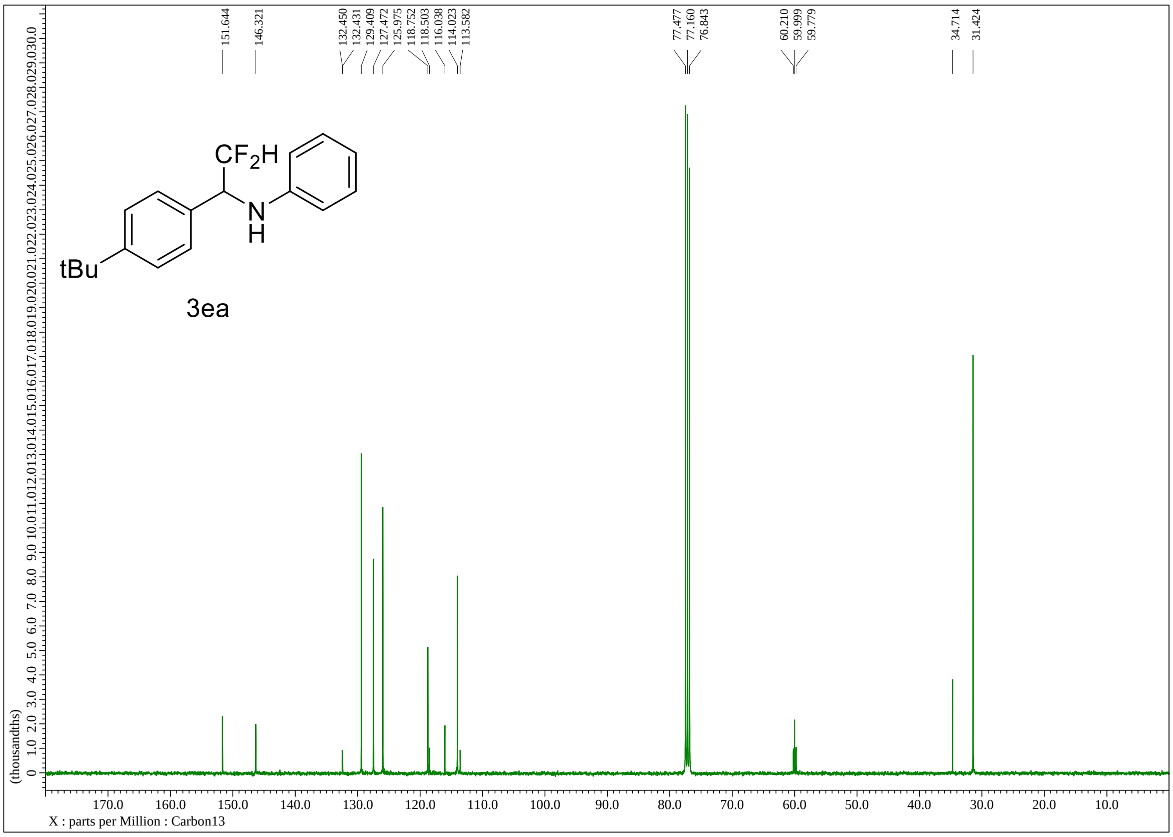

^{19}F NMR spectrum of **3ea** in $CDCl_3$. (376 MHz)

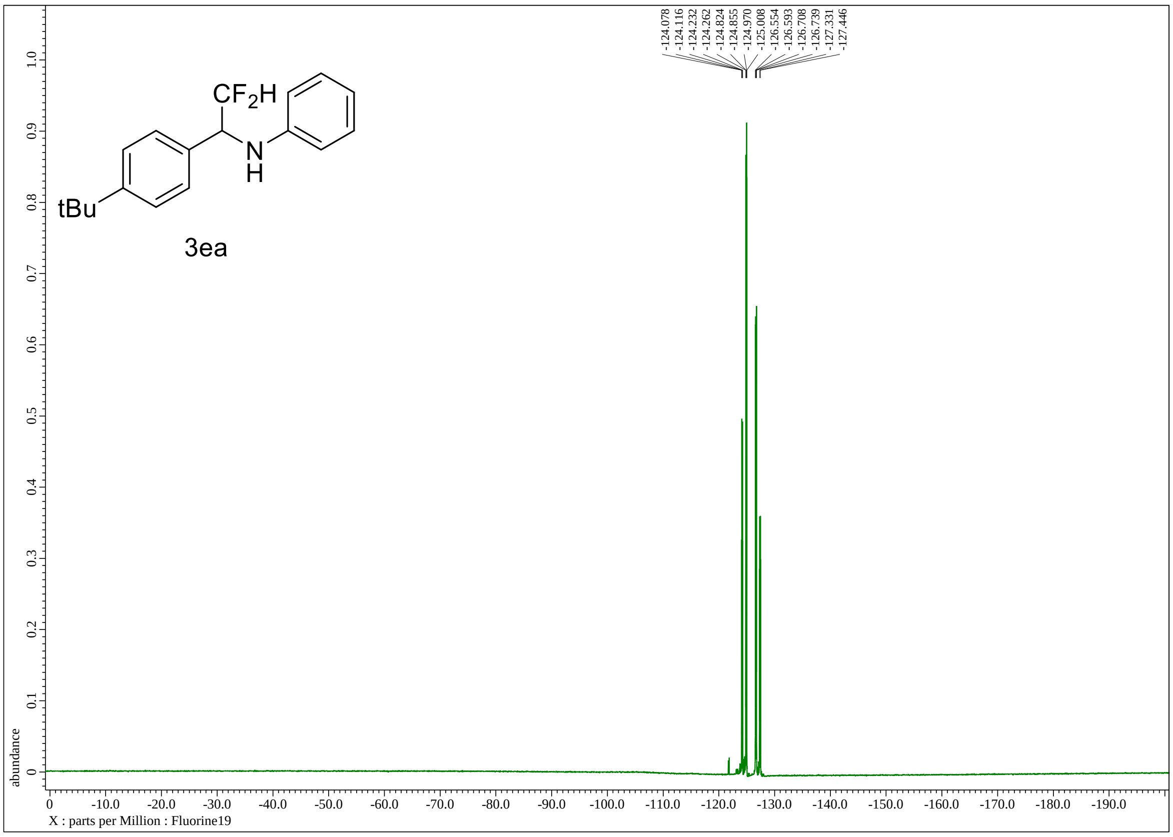

^{1}H NMR spectrum of **3fa** in $CDCl_3$. (400 MHz)

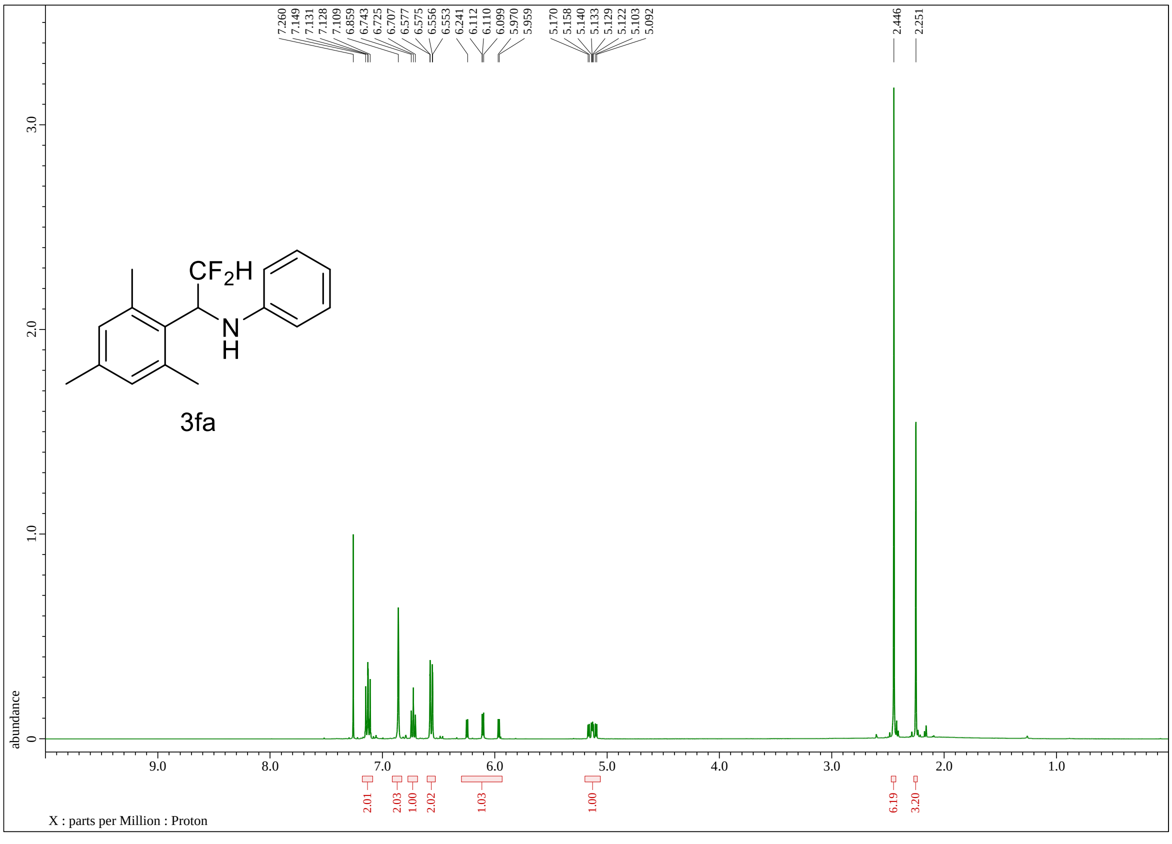

^{13}C NMR spectrum of **3fa** in $CDCl_3$. (100 MHz)

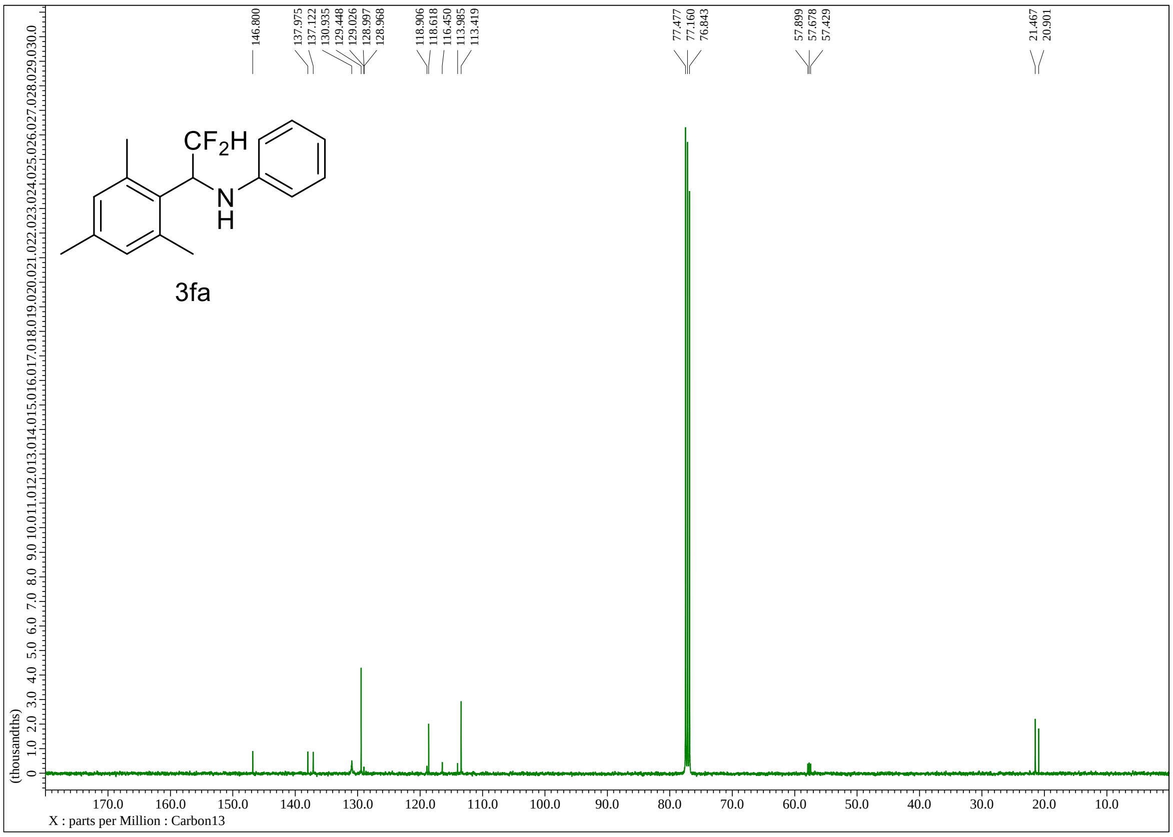

^{19}F NMR spectrum of **3fa** in $CDCl_3$. (376 MHz)

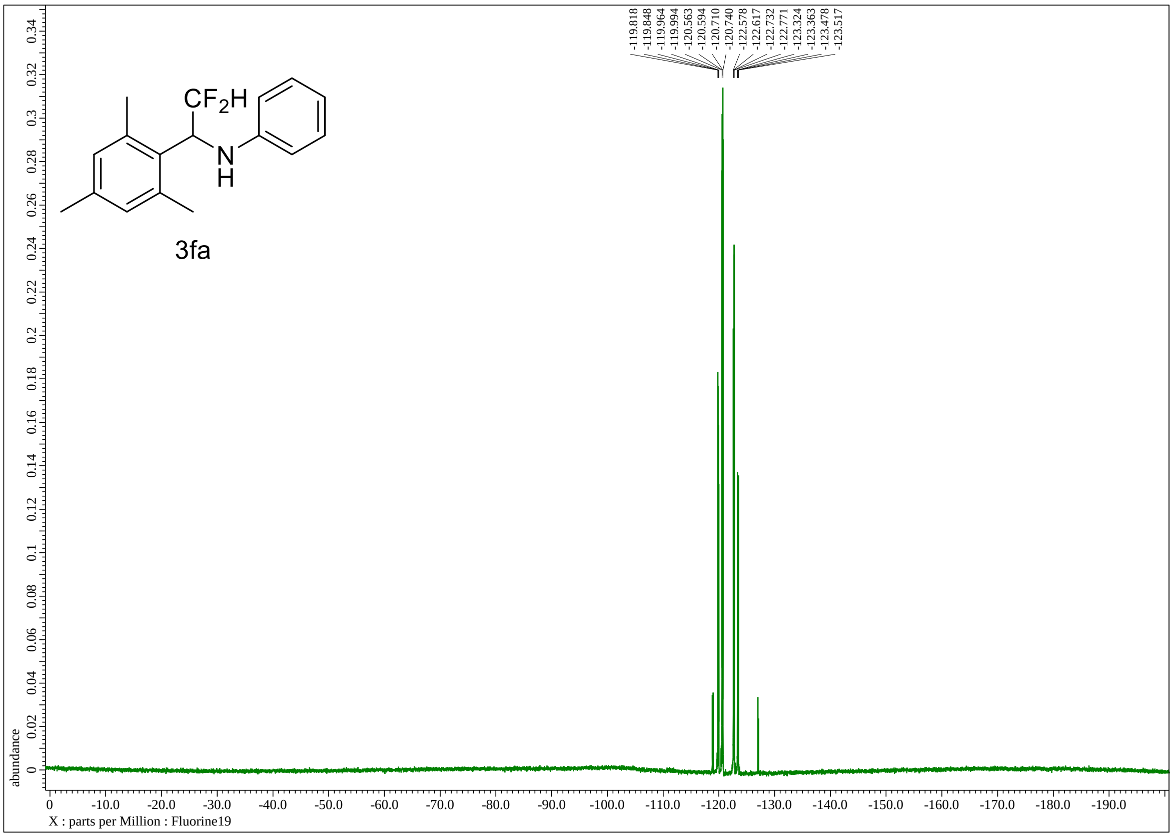

^{1}H NMR spectrum of **3ga** in $CDCl_3$. (400 MHz)

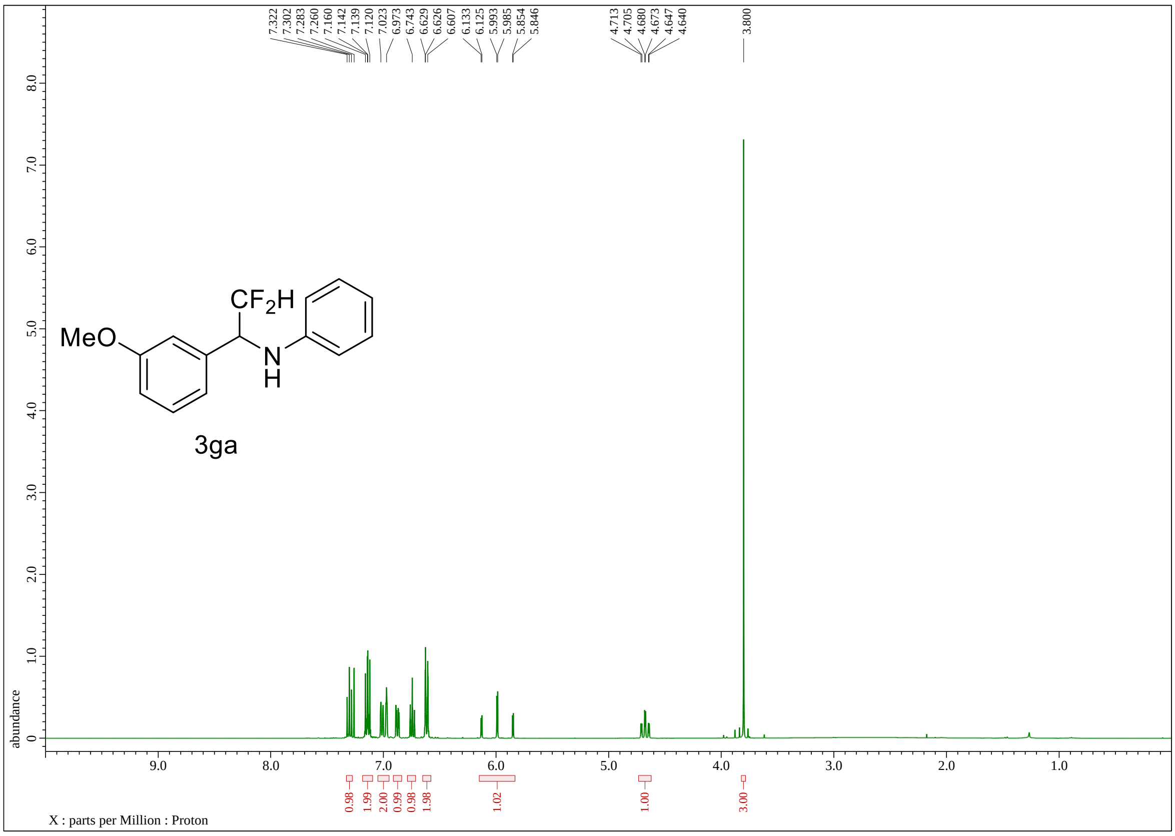

^{13}C NMR spectrum of **3ga** in $CDCl_3$. (100 MHz)

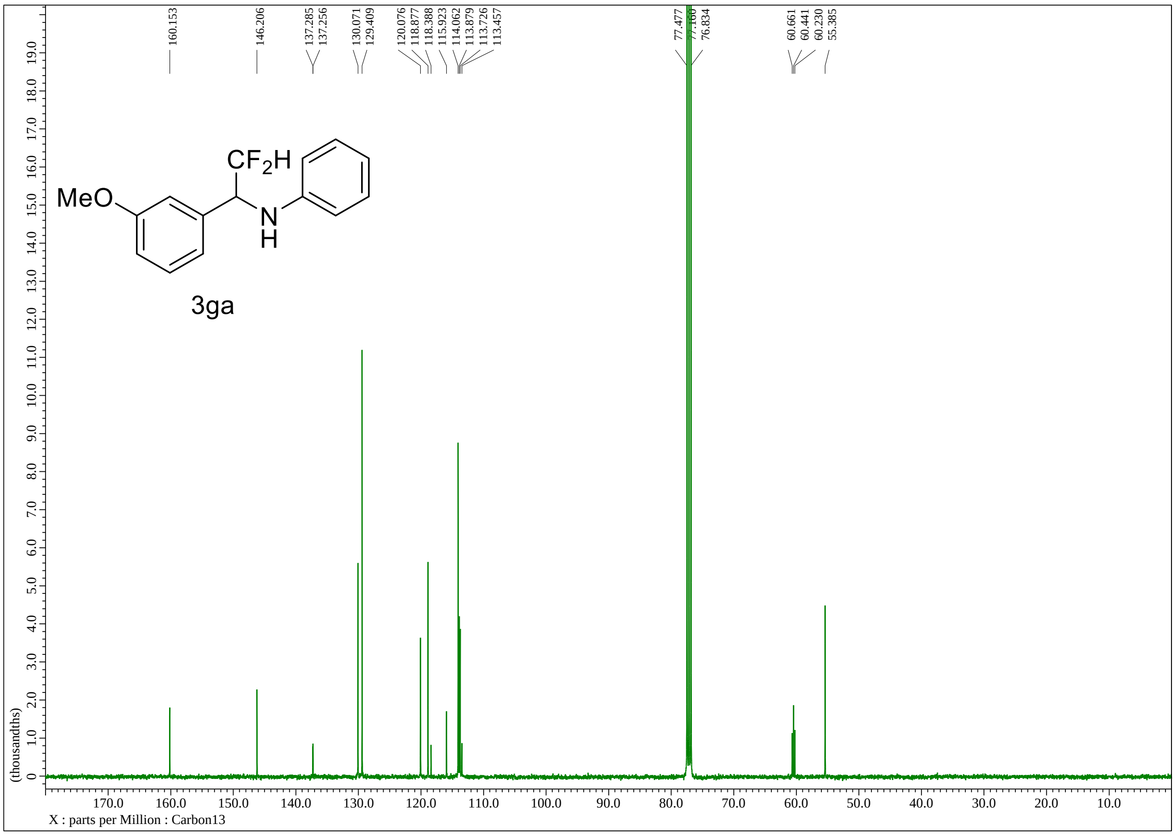

^{19}F NMR spectrum of **3ga** in $CDCl_3$. (376 MHz)

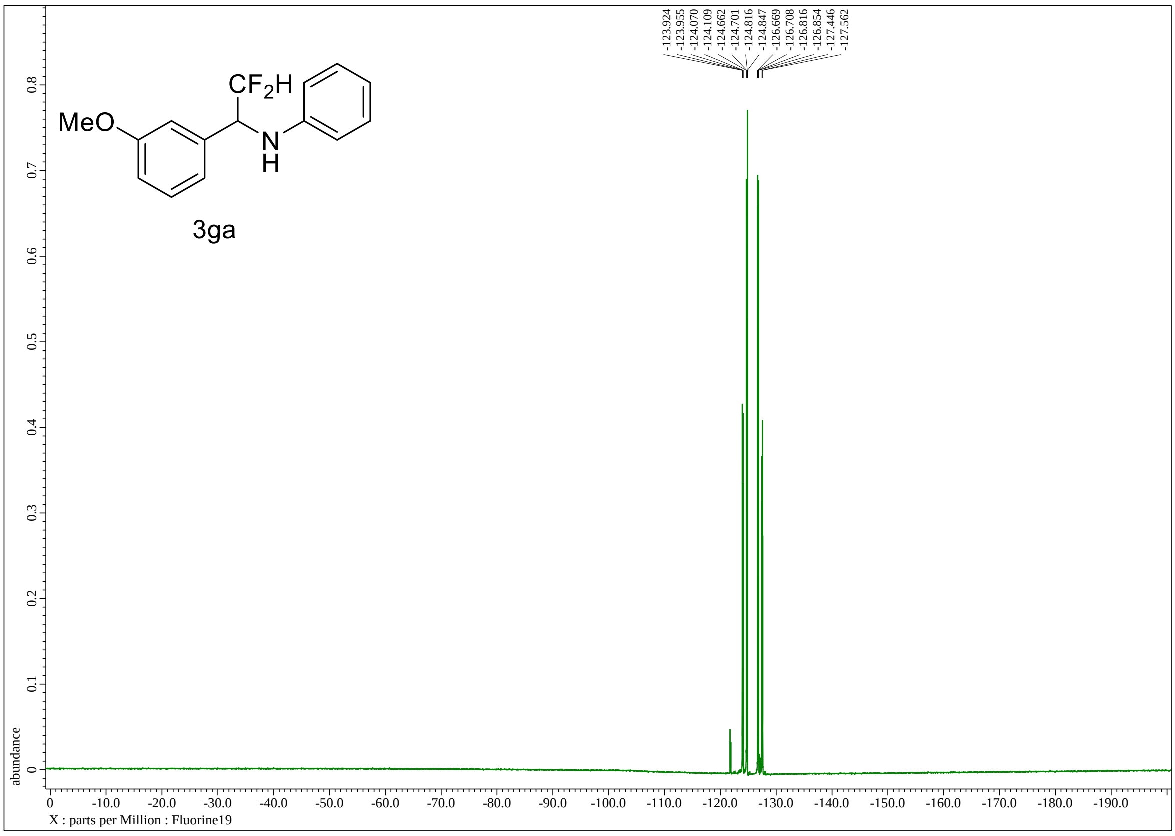

^{1}H NMR spectrum of **3ha** in $CDCl_3$. (400 MHz)

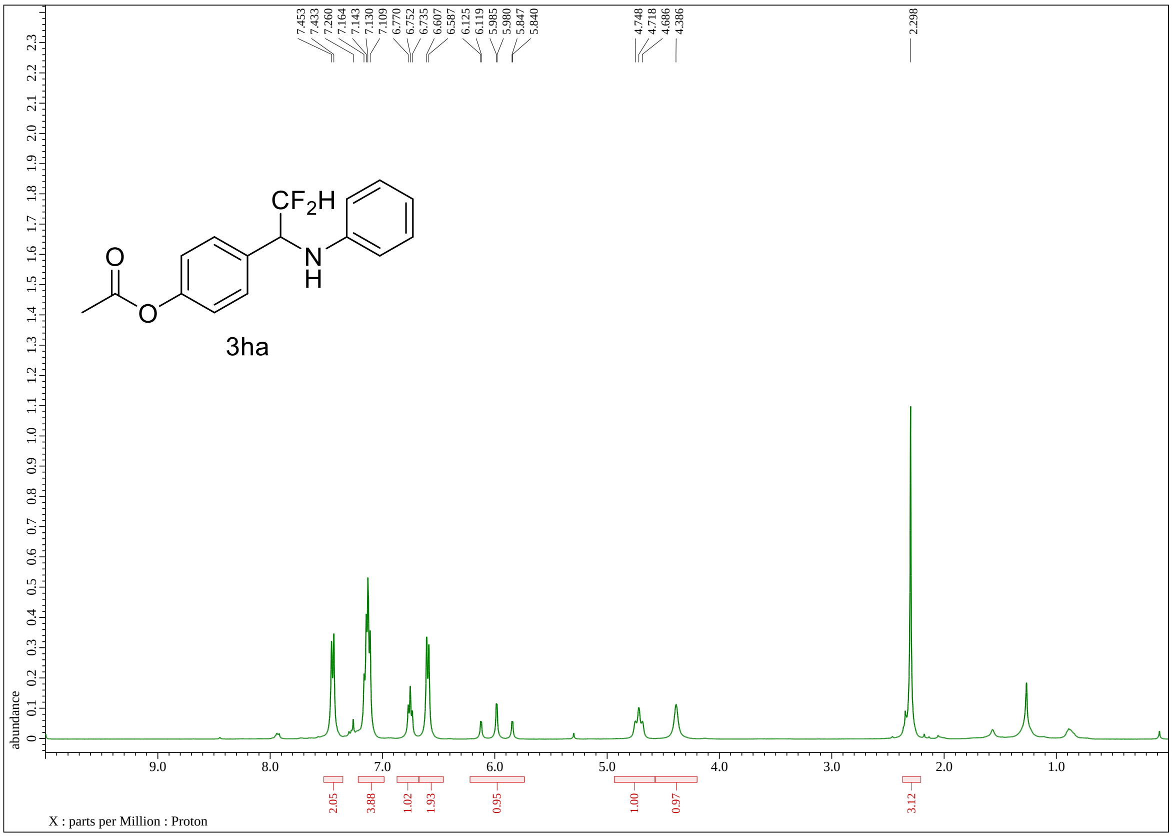

^{13}C NMR spectrum of **3ha** in $CDCl_3$. (100 MHz)

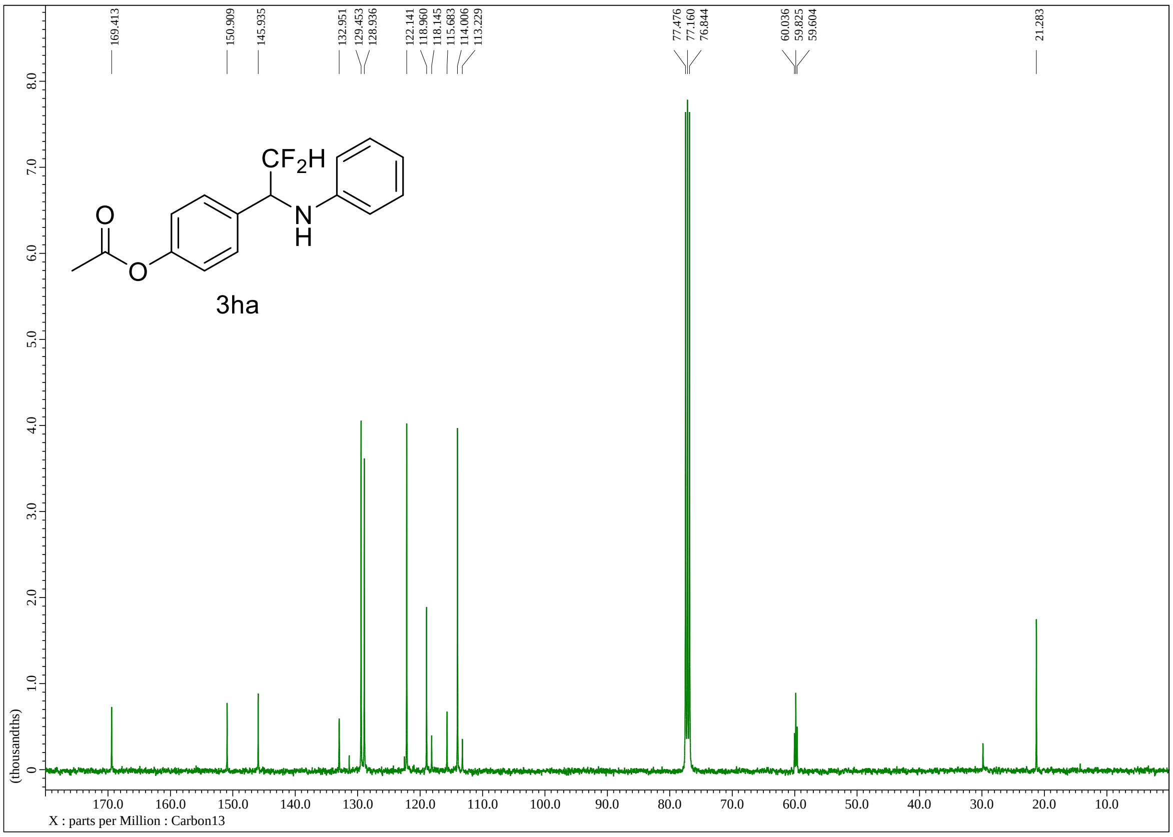

^{19}F NMR spectrum of **3ha** in $CDCl_3$. (376 MHz)

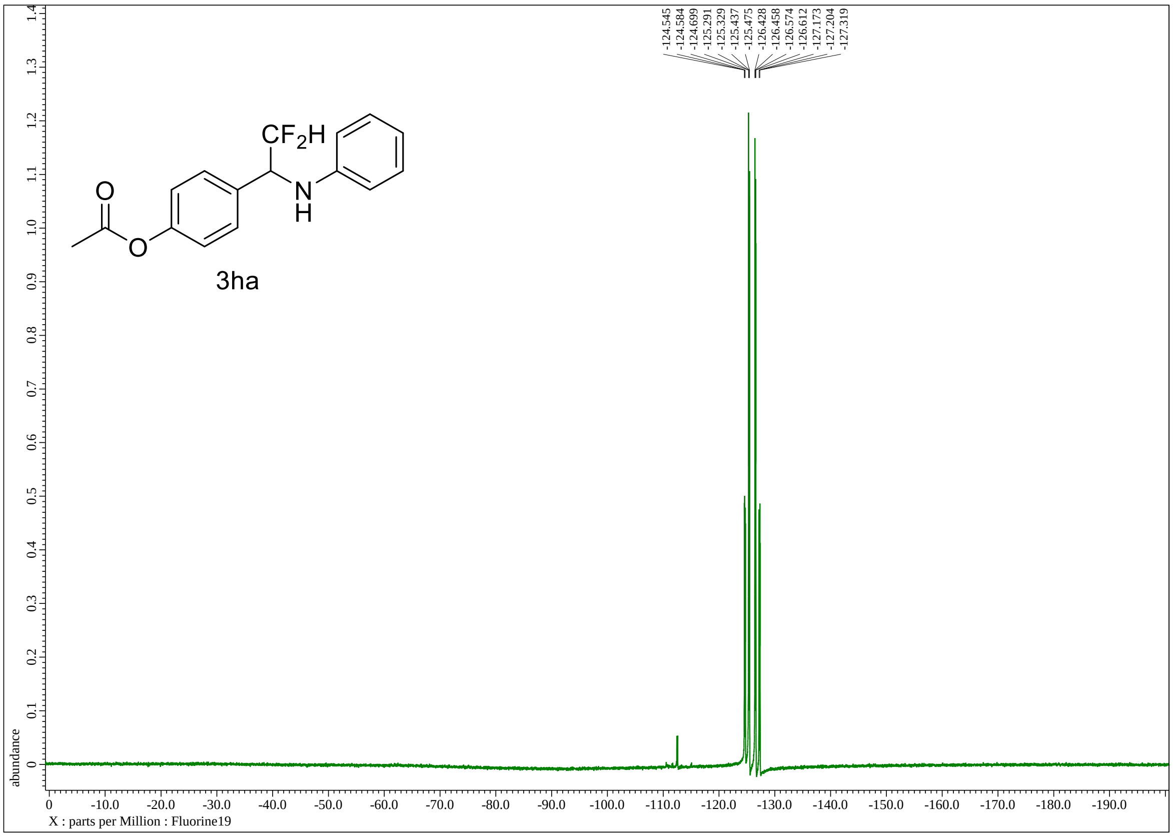

^{1}H NMR spectrum of **3ia** in $CDCl_3$. (400 MHz)

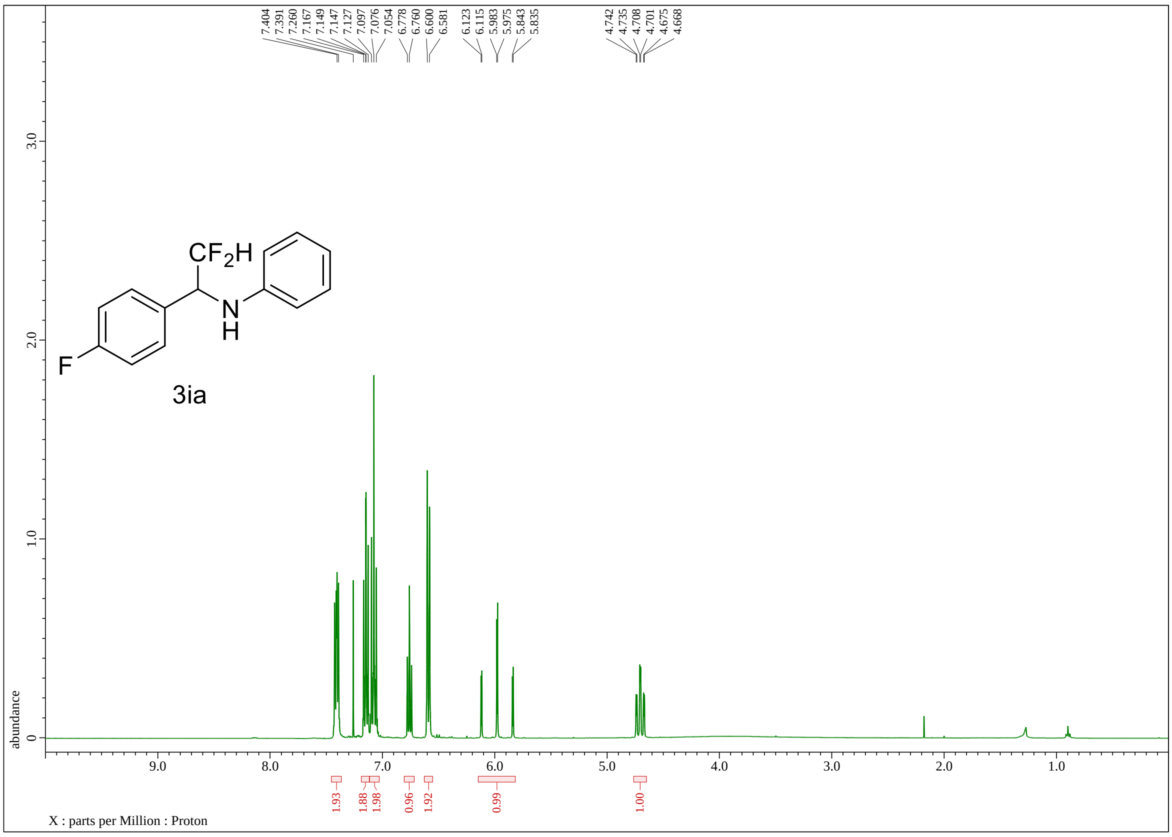

^{13}C NMR spectrum of **3ia** in $CDCl_3$. (100 MHz)

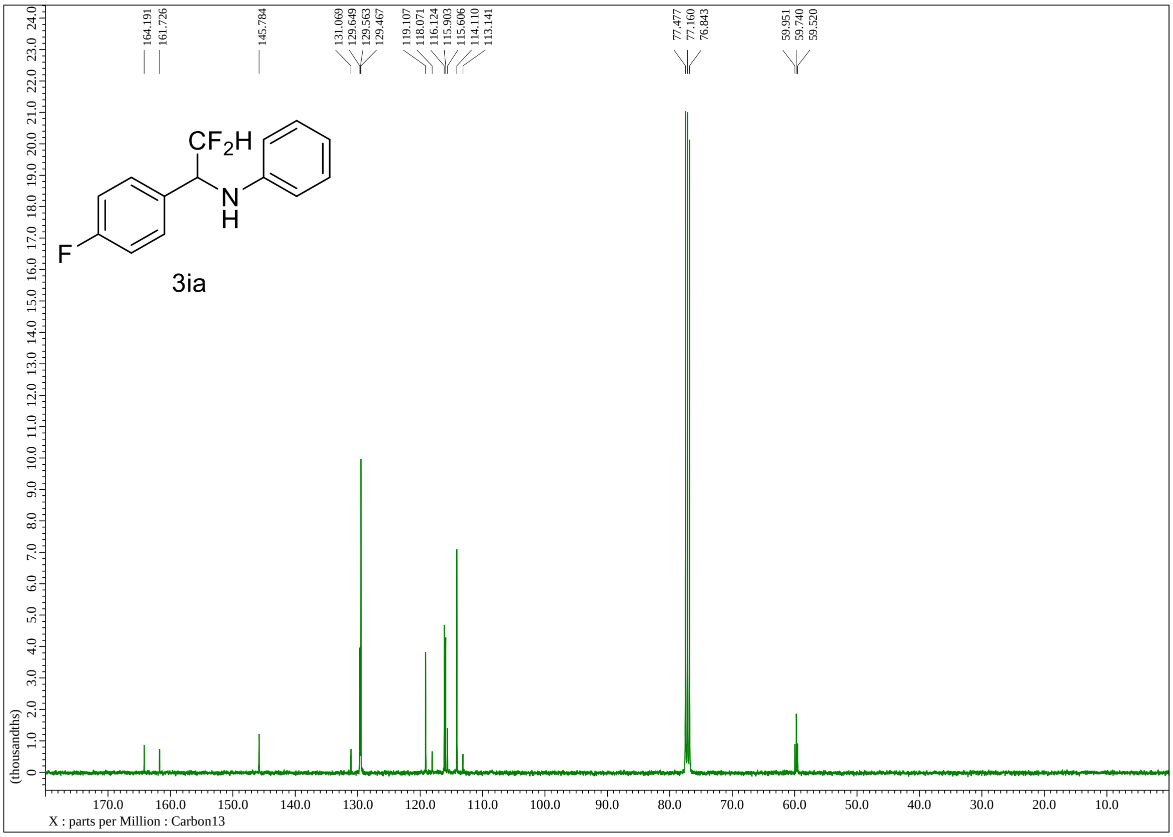

^{19}F NMR spectrum of **3ia** in $CDCl_3$. (376 MHz)

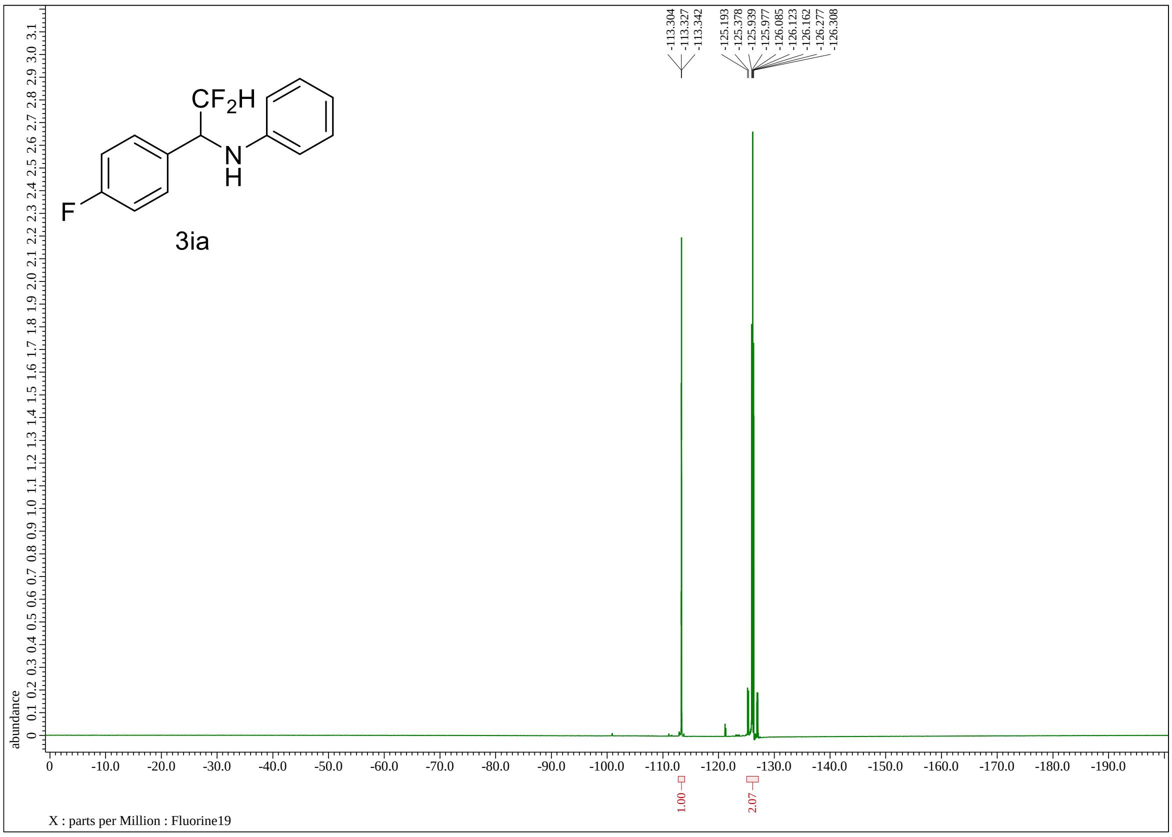

^{1}H NMR spectrum of **3ja** in $CDCl_3$. (400 MHz)

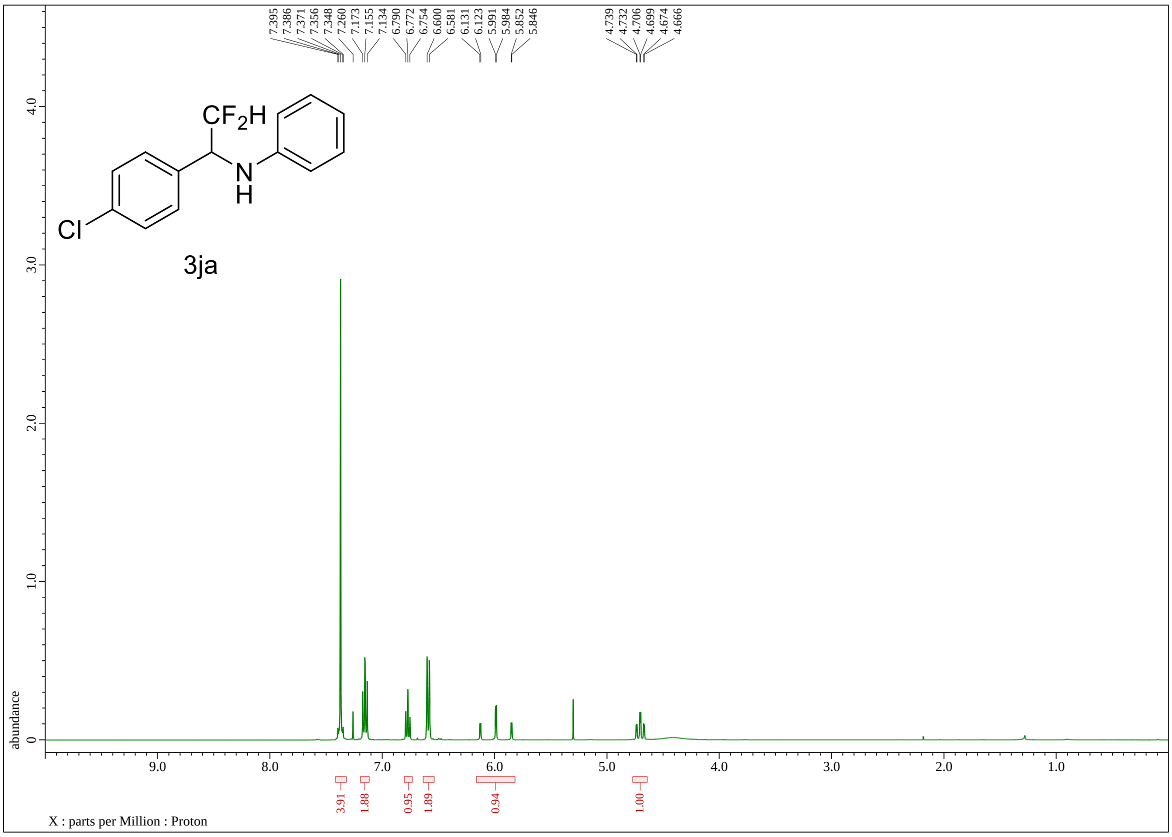

^{13}C NMR spectrum of **3ja** in $CDCl_3$. (100 MHz)

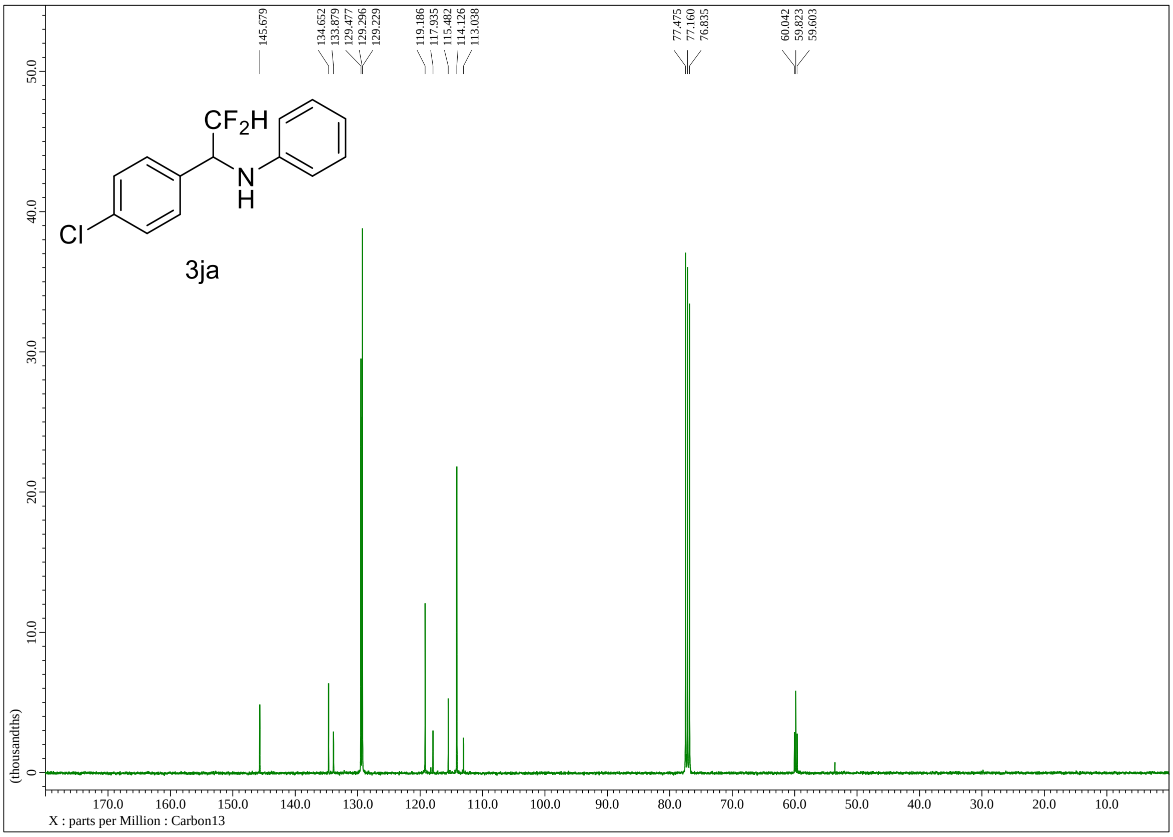

^{19}F NMR spectrum of **3ja** in $CDCl_3$. (376 MHz)

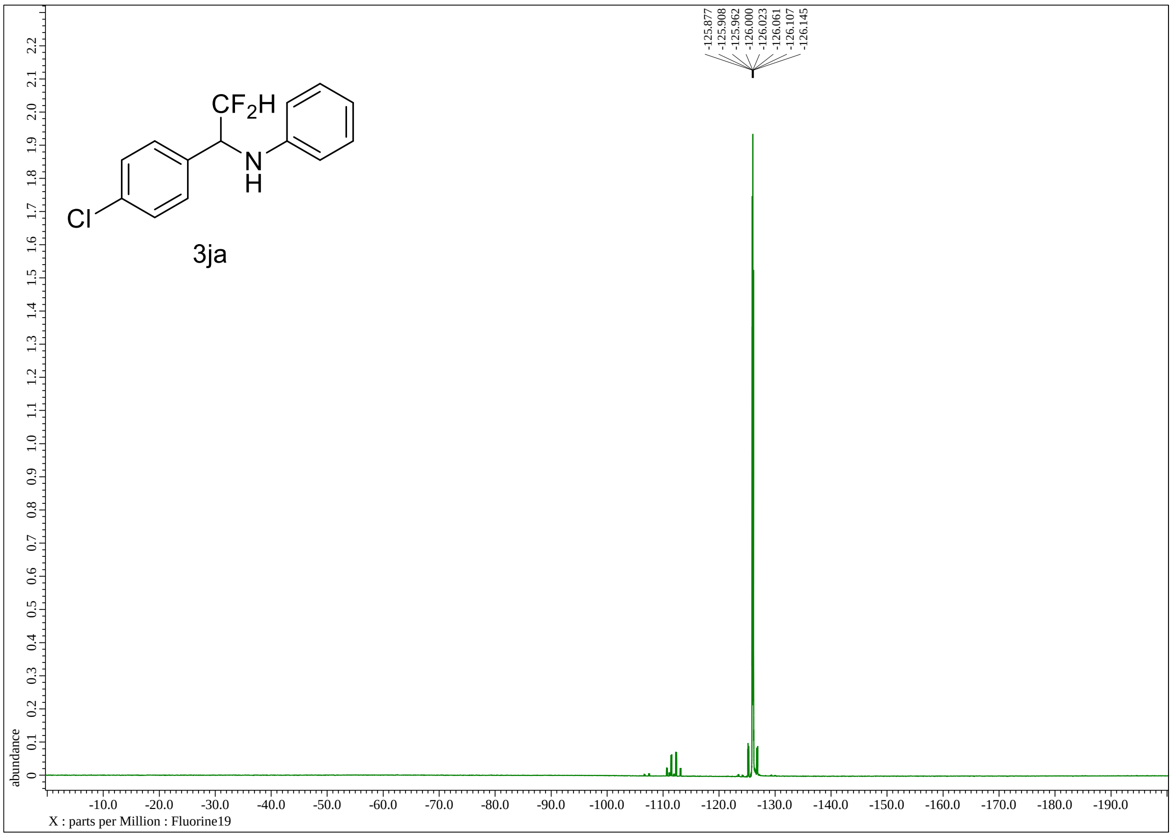

^{1}H NMR spectrum of **3ka** in $CDCl_3$. (400 MHz)

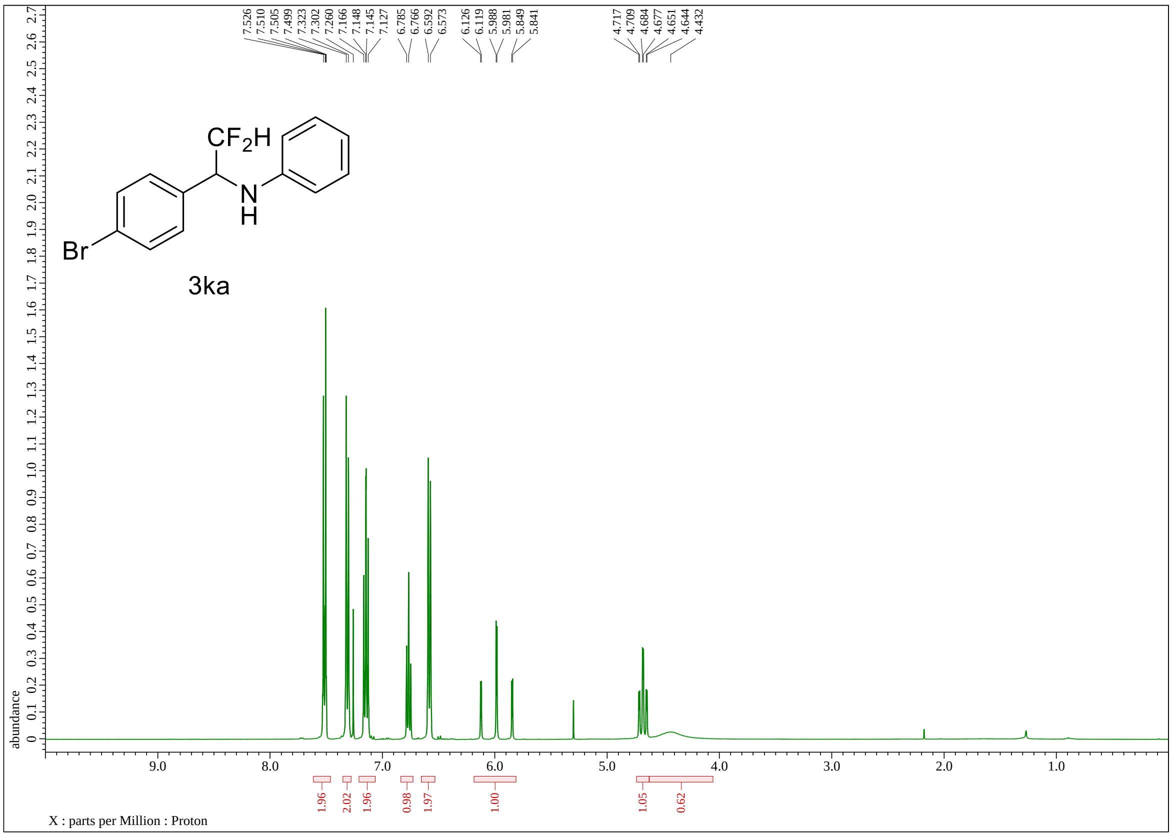

^{13}C NMR spectrum of **3ka** in $CDCl_3$. (100 MHz)

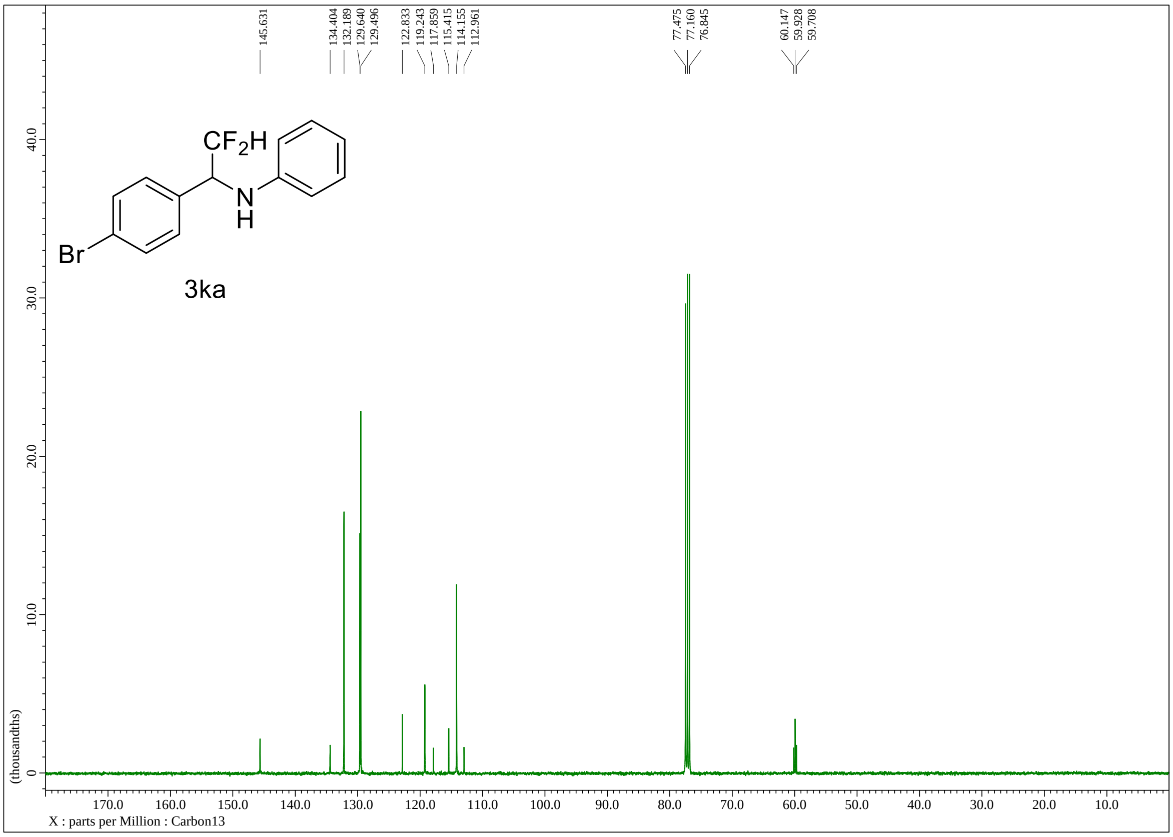

^{19}F NMR spectrum of **3ka** in $CDCl_3$. (376 MHz)

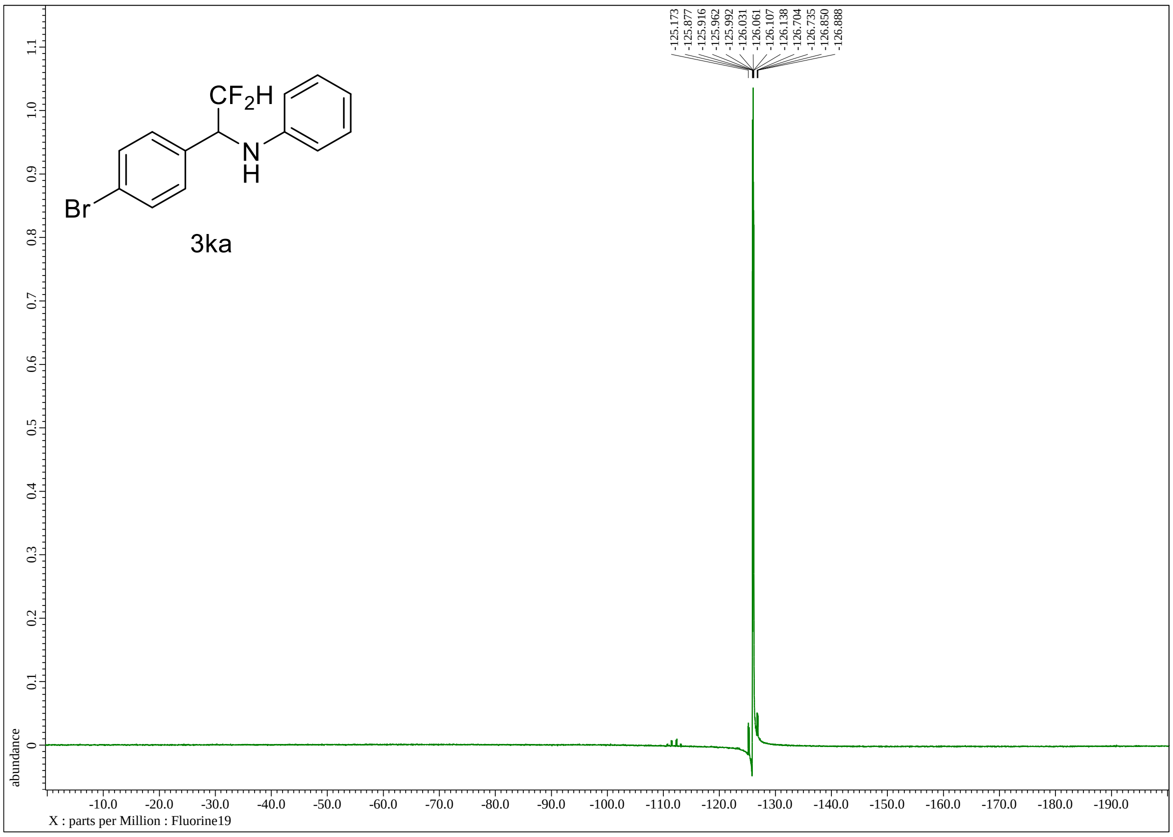

^{1}H NMR spectrum of **3la** in $CDCl_3$. (400 MHz)

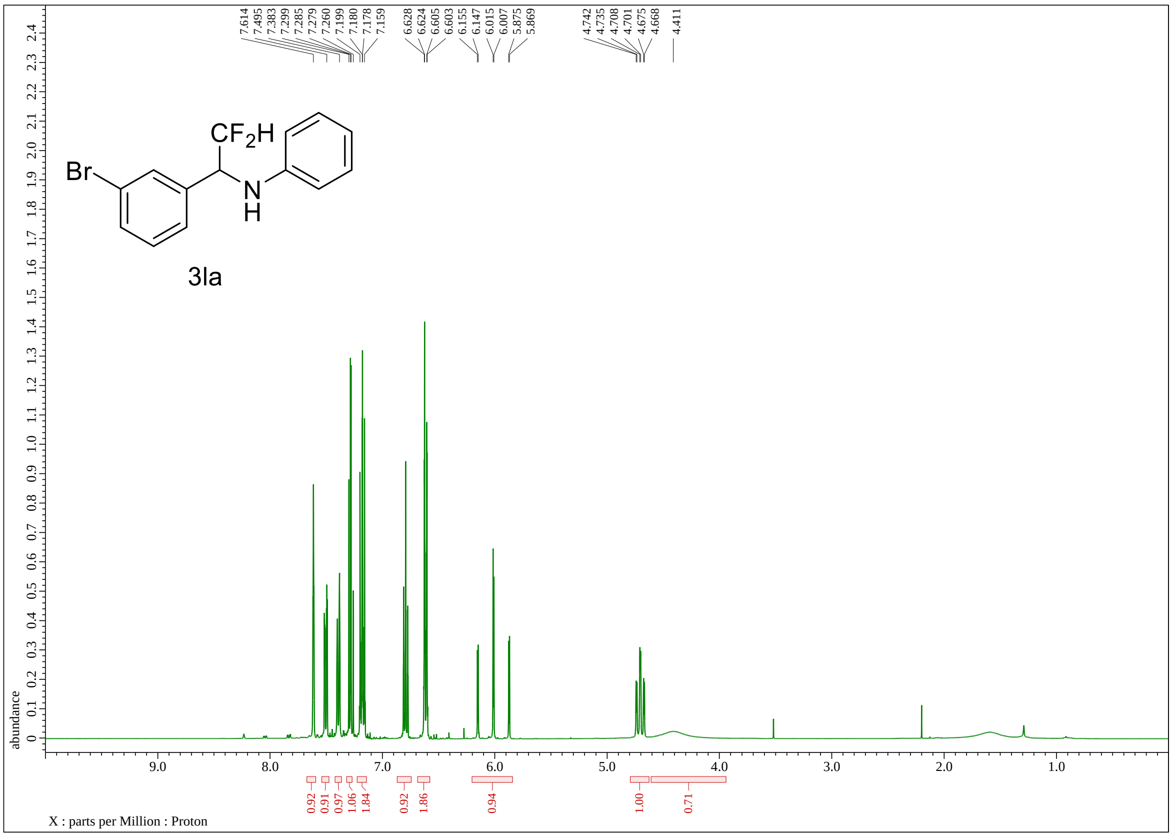

^{13}C NMR spectrum of **3la** in $CDCl_3$. (100 MHz)

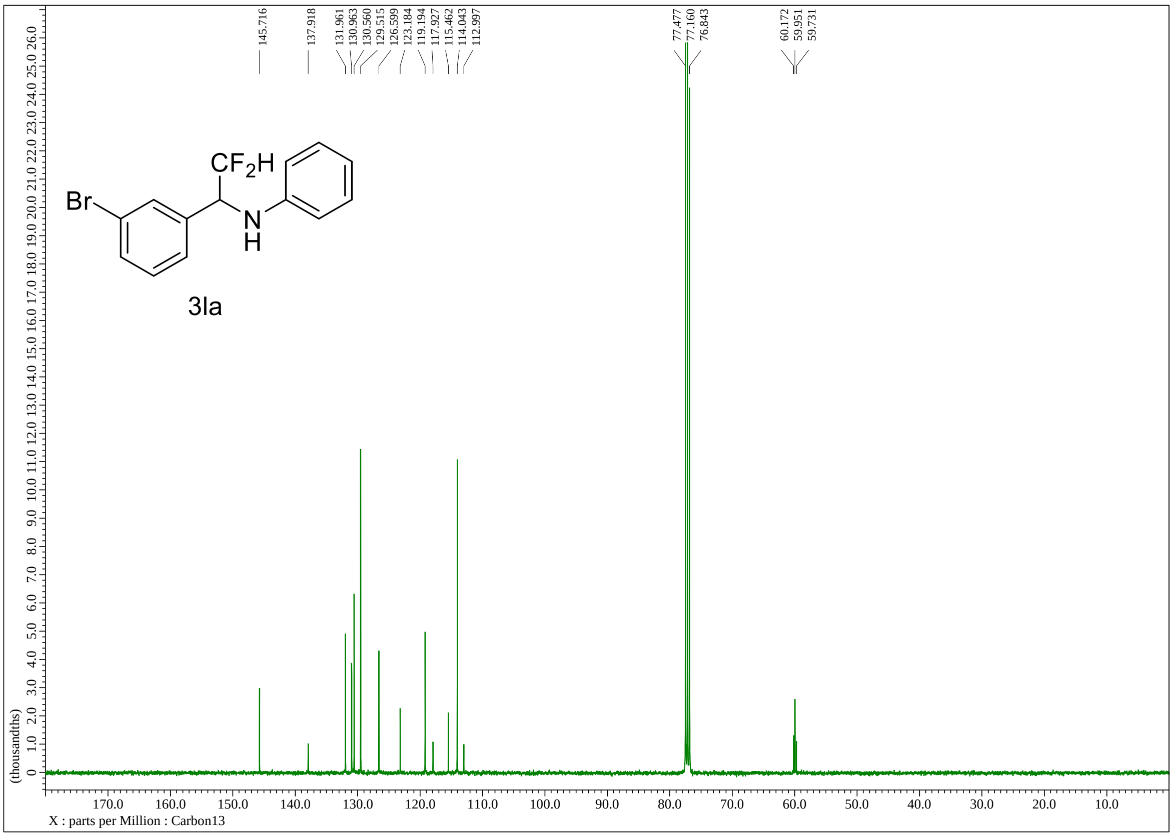

^{19}F NMR spectrum of **3la** in $CDCl_3$. (376 MHz)

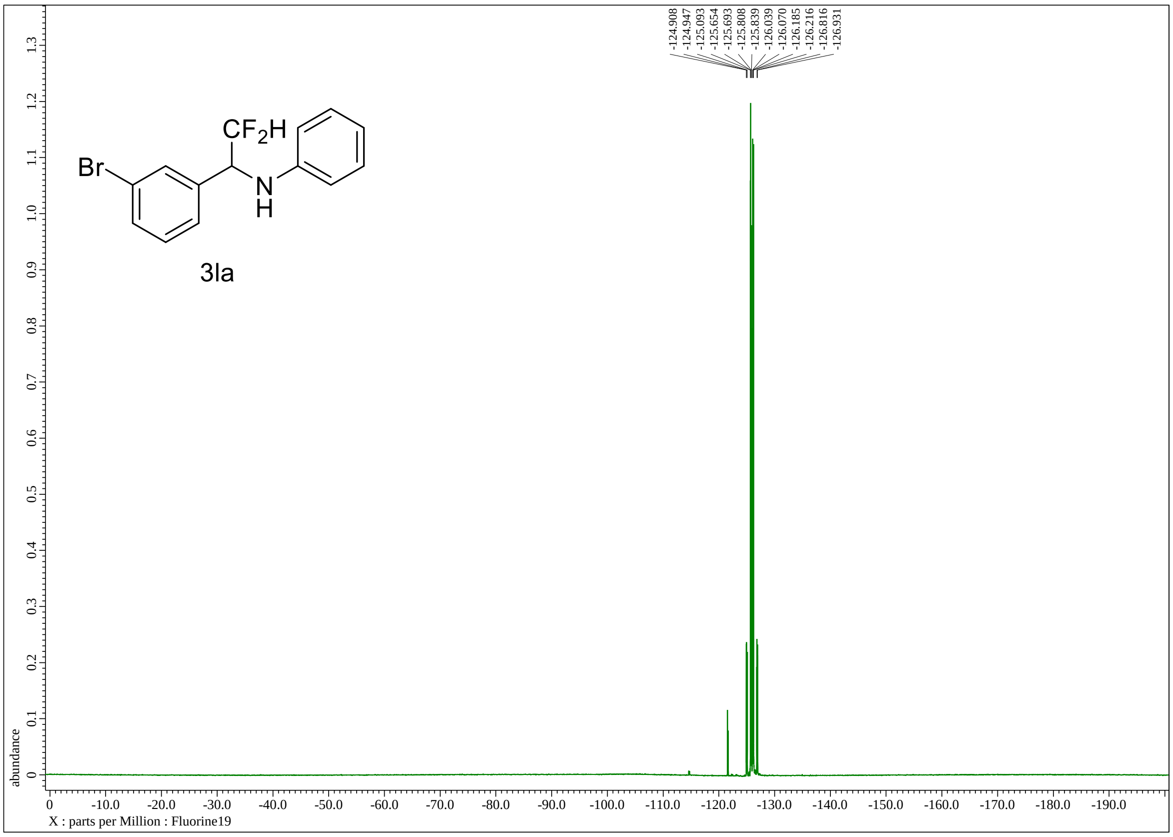

^{1}H NMR spectrum of **3ma** in $CDCl_3$. (400 MHz)

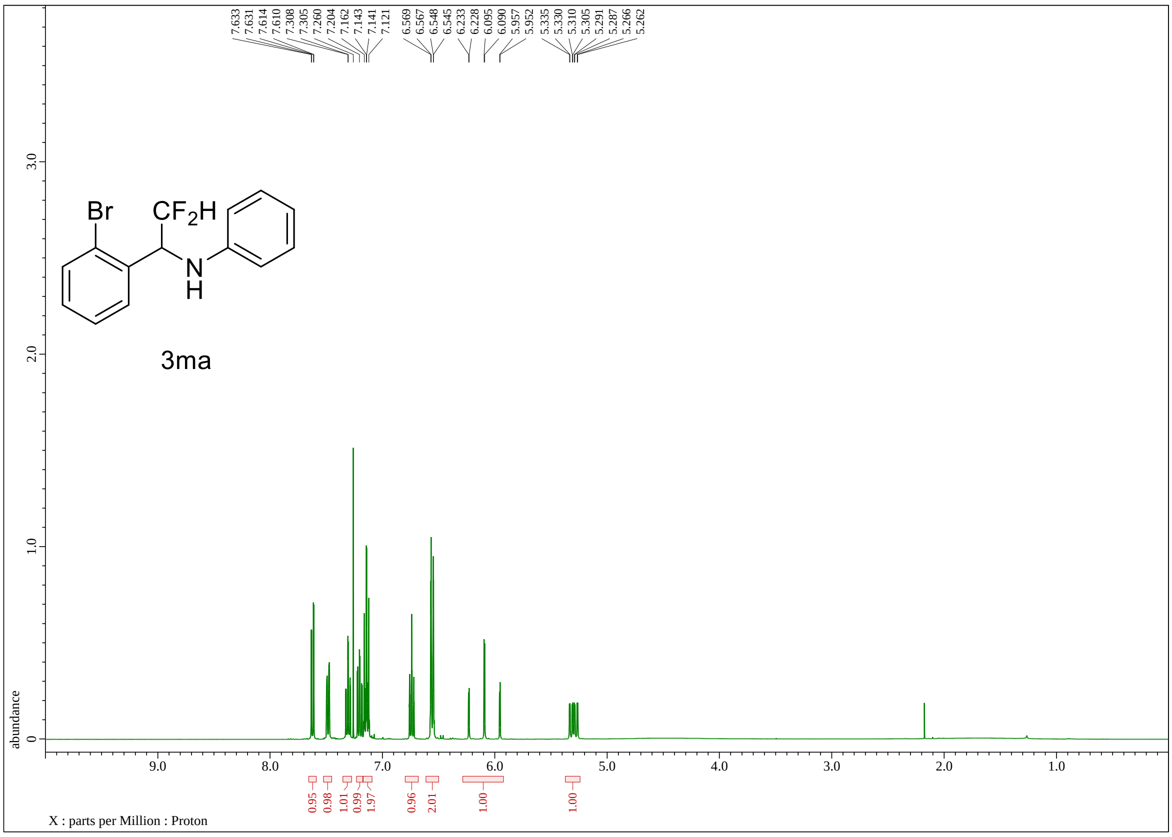

^{13}C NMR spectrum of **3ma** in $CDCl_3$. (100 MHz)

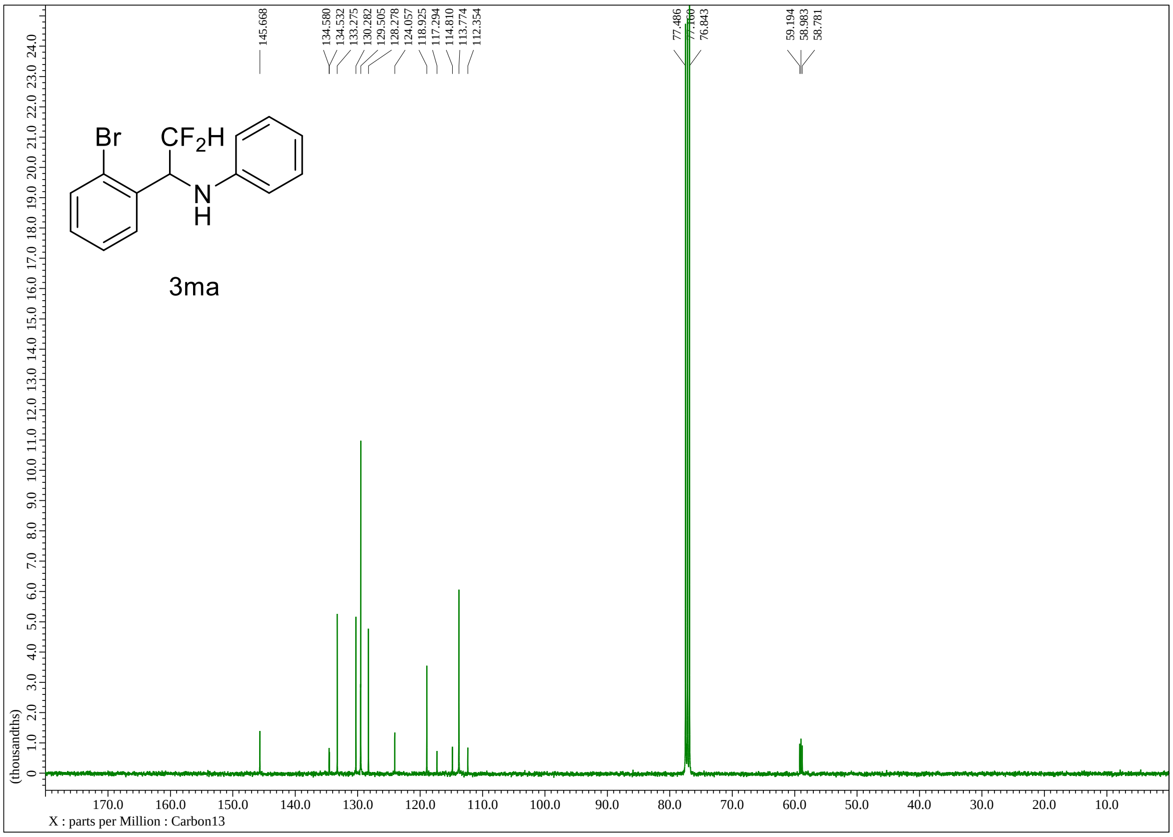

^{19}F NMR spectrum of **3ma** in $CDCl_3$. (376 MHz)

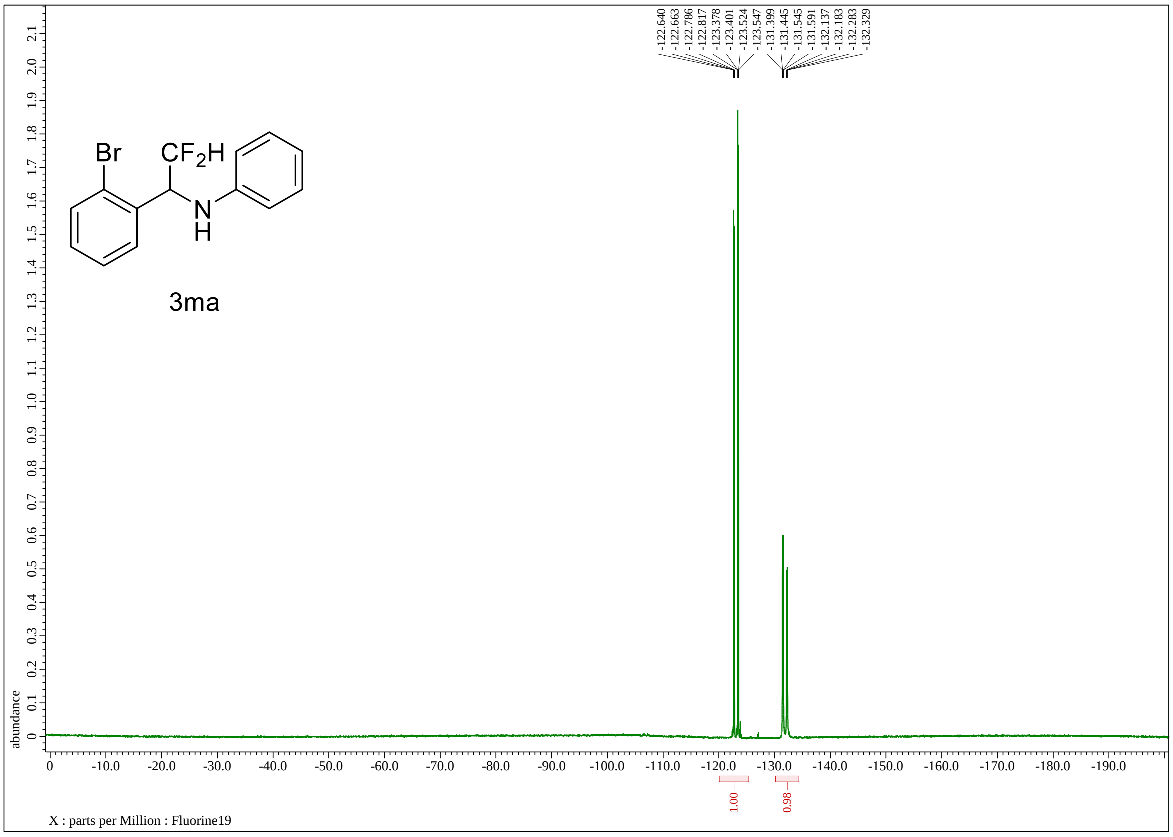

^{1}H NMR spectrum of **3na** in $CDCl_3$. (400 MHz)

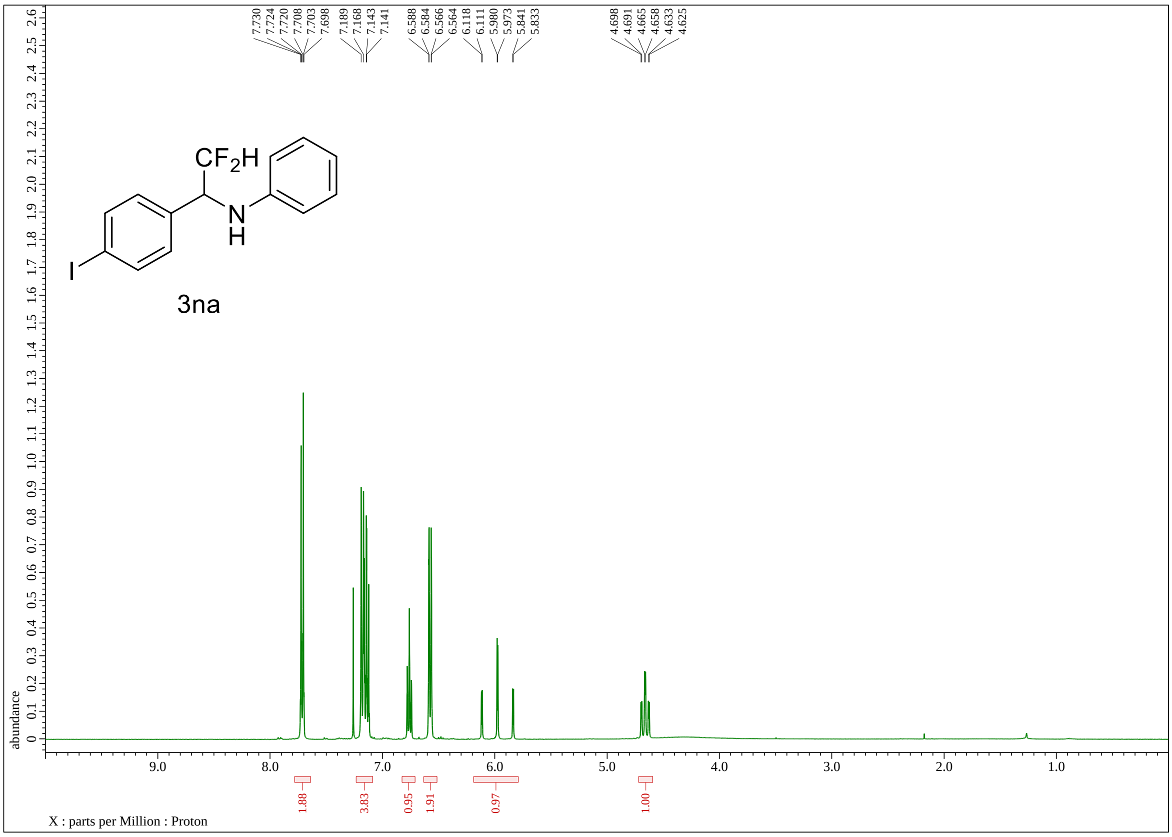

^{13}C NMR spectrum of **3na** in $CDCl_3$. (100 MHz)

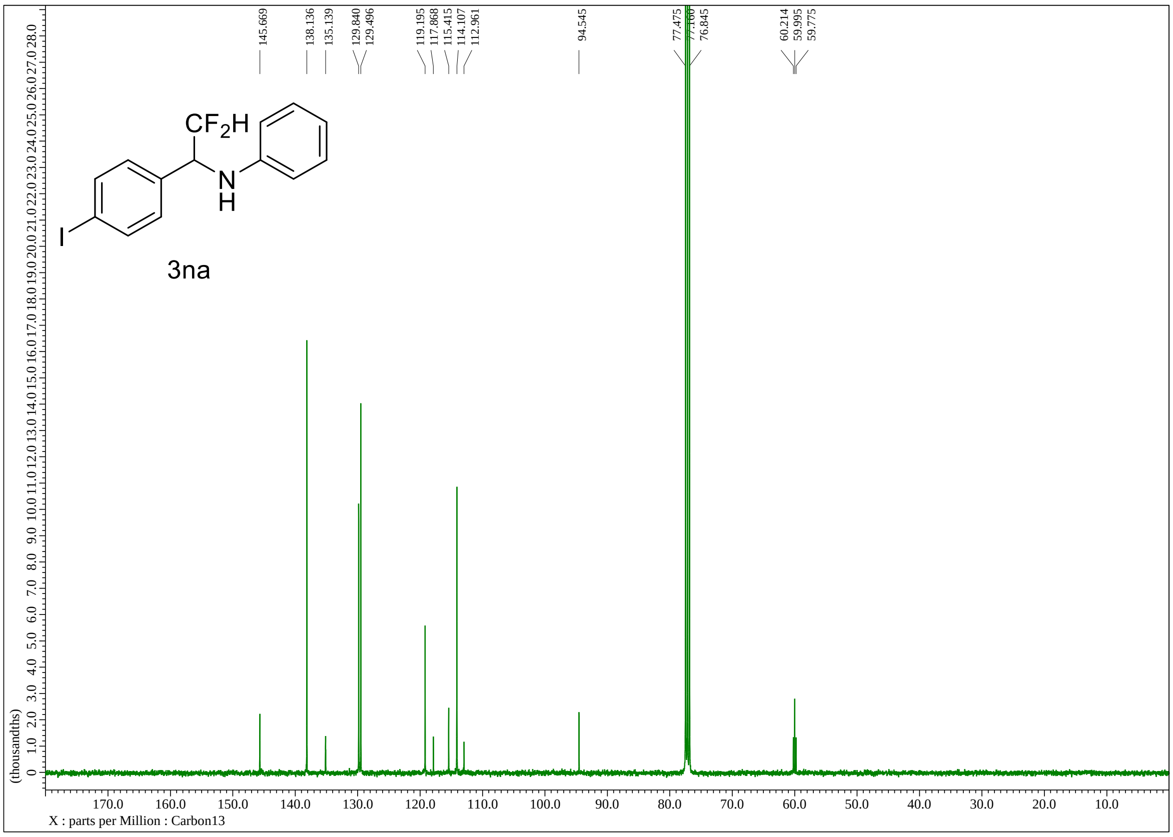

^{19}F NMR spectrum of **3na** in $CDCl_3$. (376 MHz)

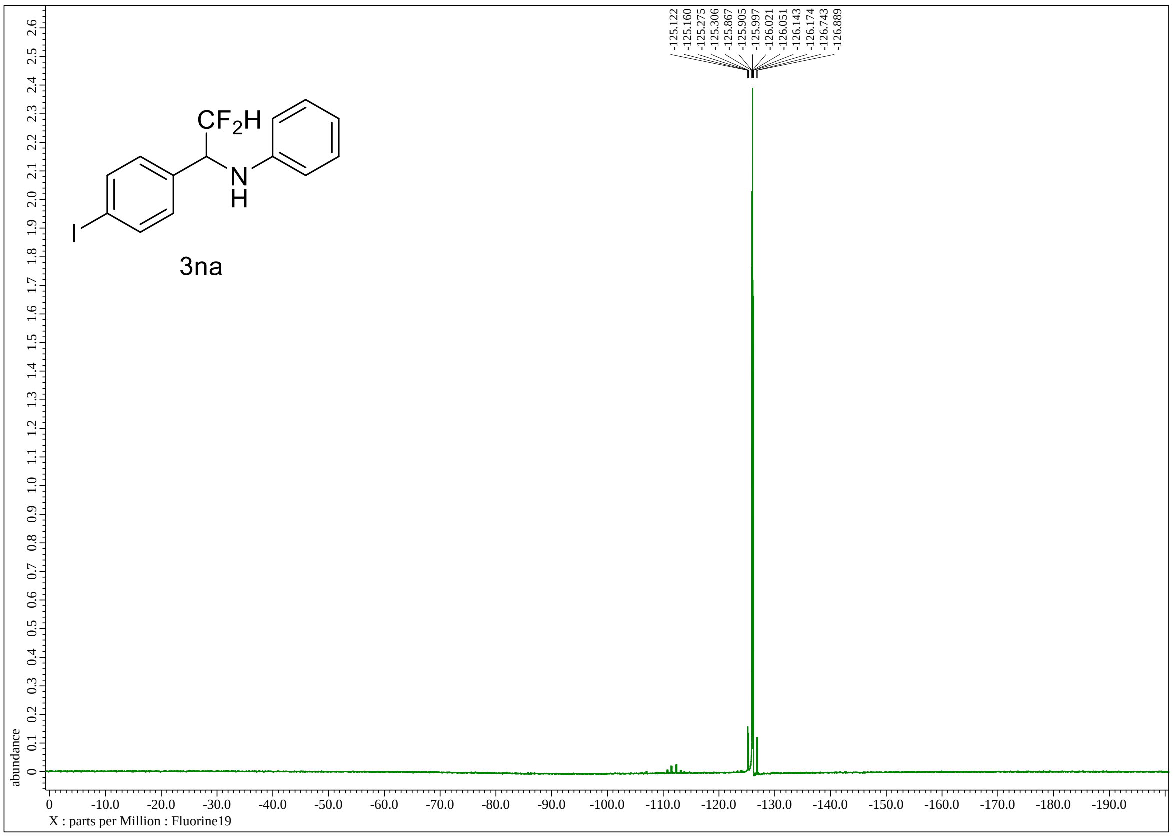

^{1}H NMR spectrum of **3oa** in $CDCl_3$. (400 MHz)

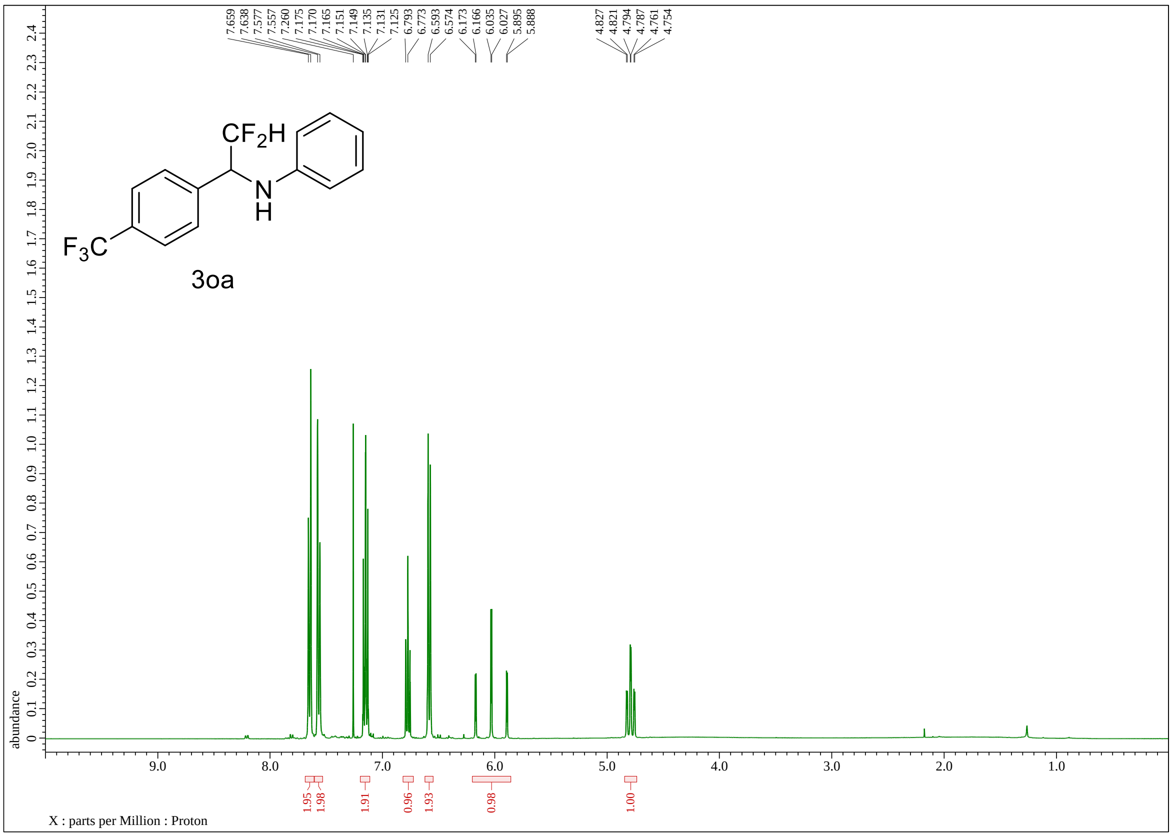

^{13}C NMR spectrum of **3oa** in $CDCl_3$. (100 MHz)

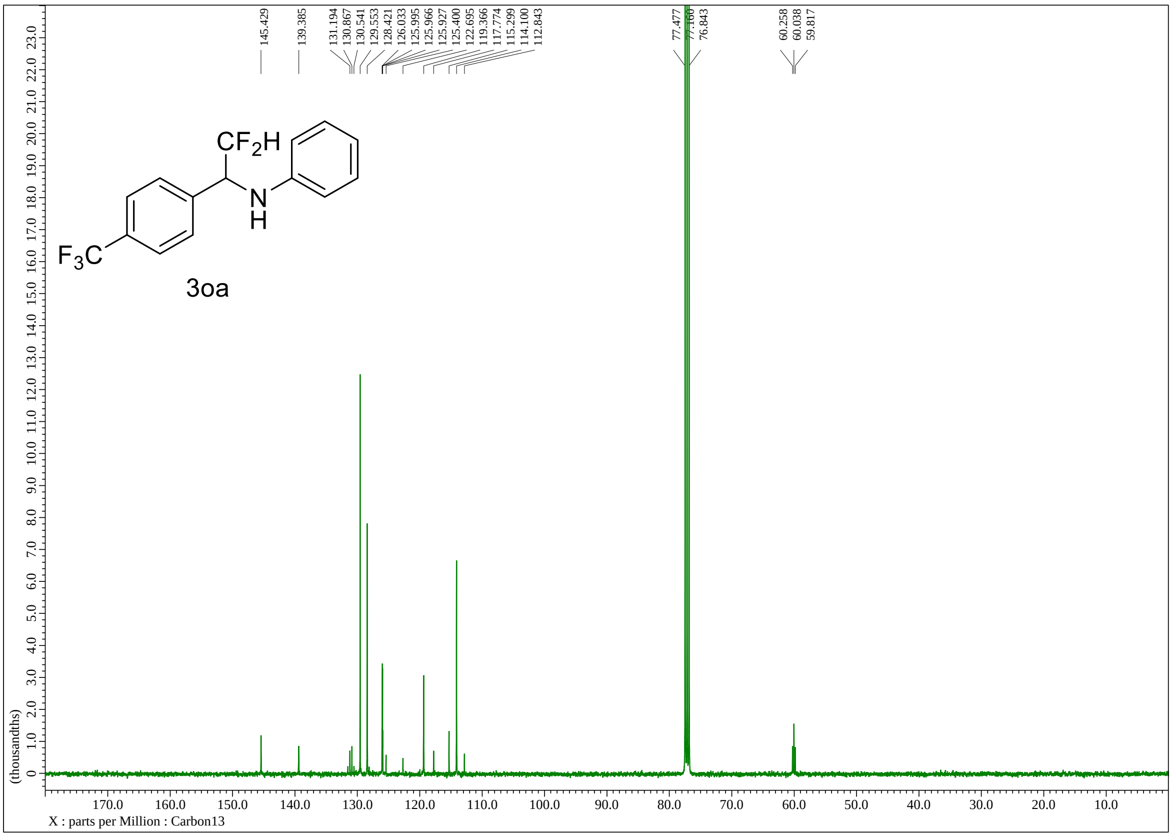

^{19}F NMR spectrum of **3oa** in $CDCl_3$. (376 MHz)

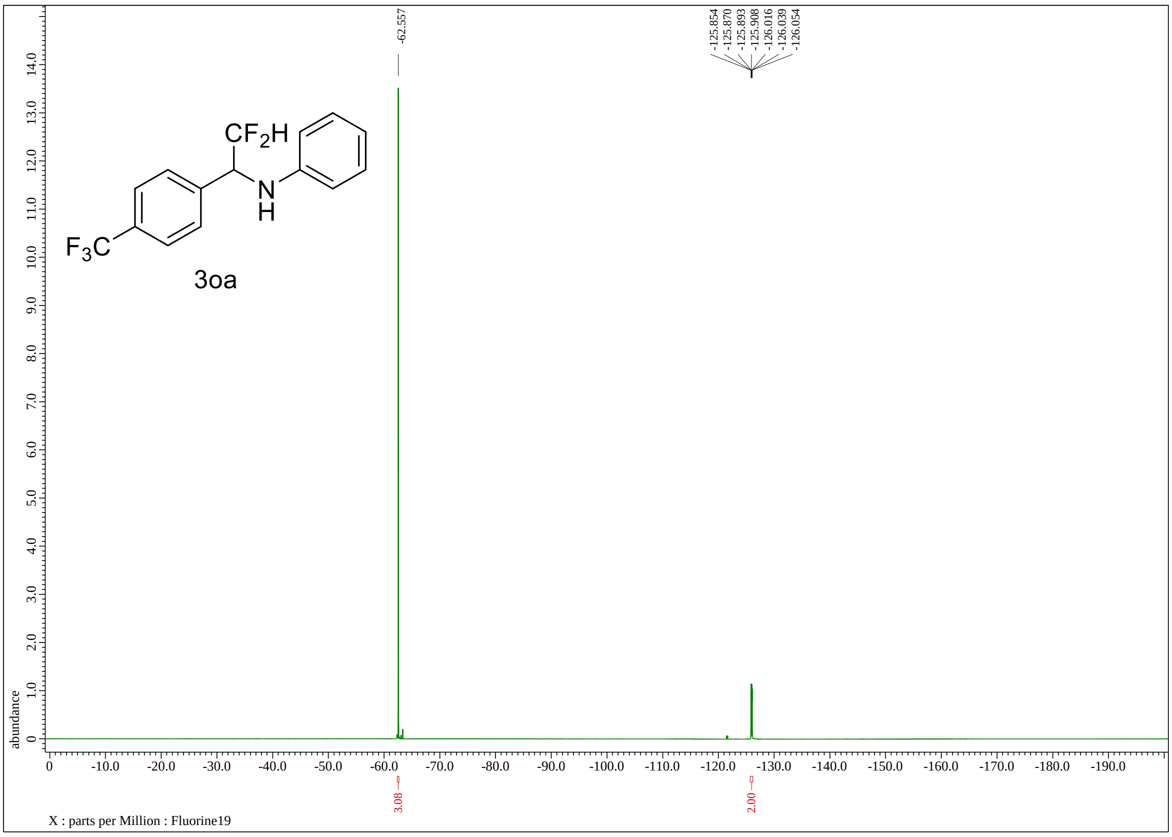

^{1}H NMR spectrum of **3pa** in $CDCl_3$. (400 MHz)

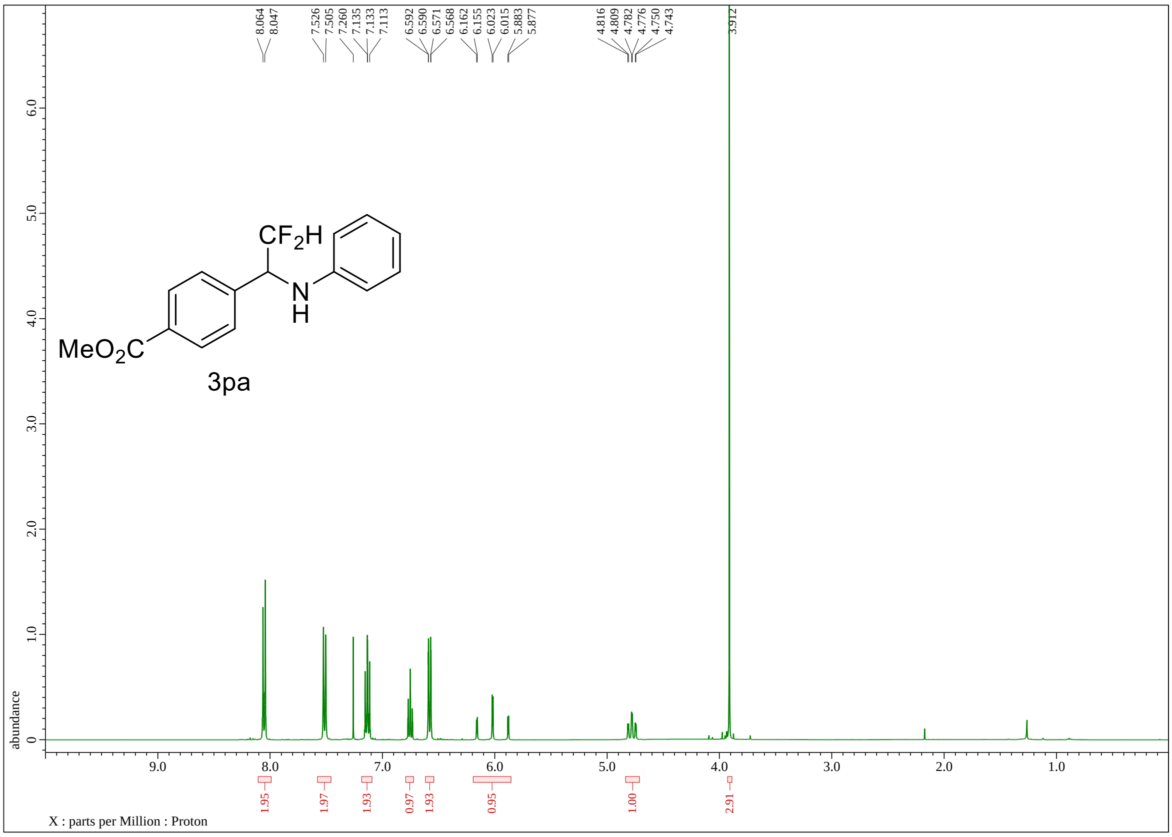

^{13}C NMR spectrum of **3pa** in $CDCl_3$. (100 MHz)

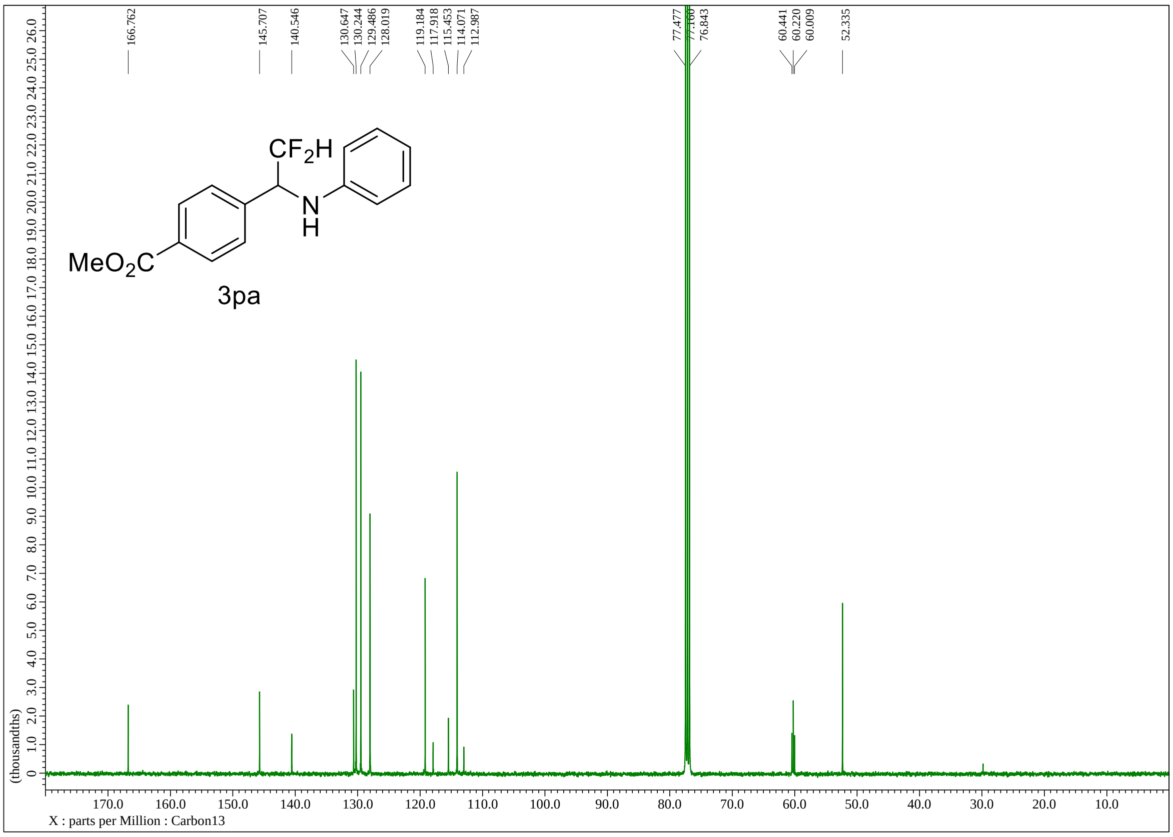

^{19}F NMR spectrum of **3pa** in $CDCl_3$. (376 MHz)

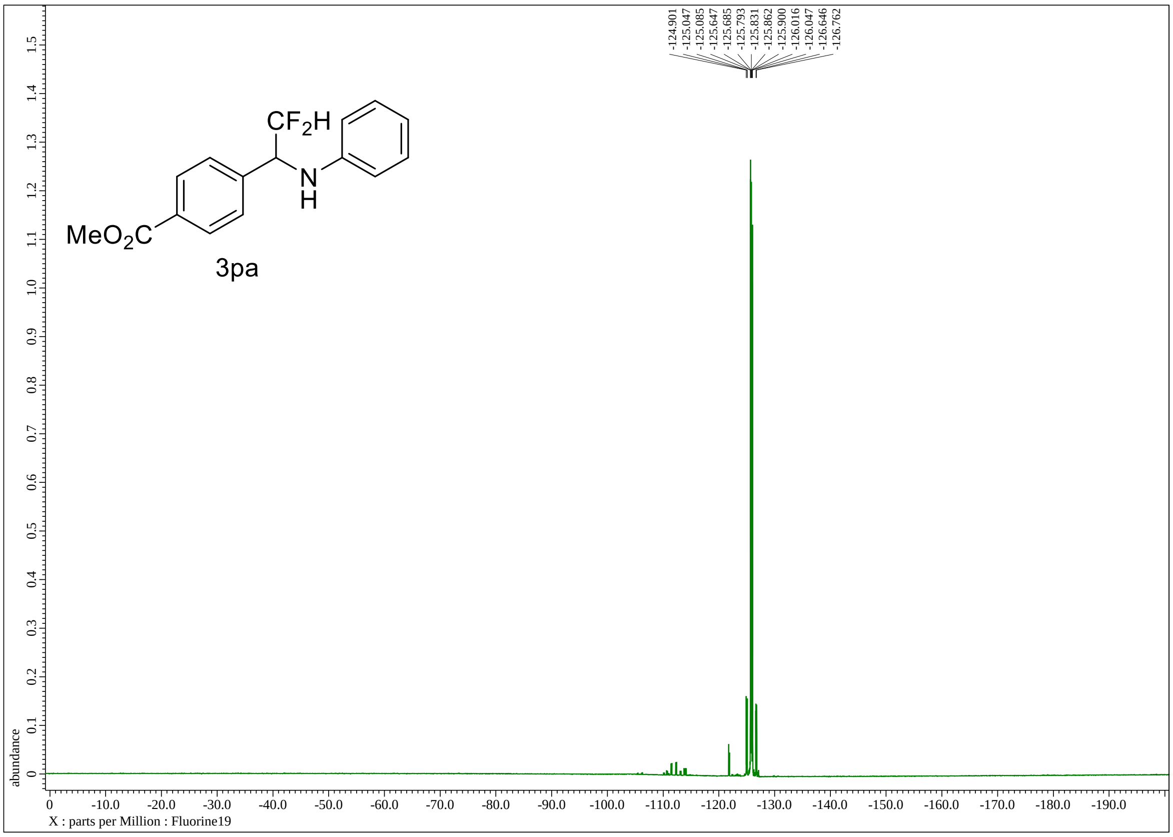

^{1}H NMR spectrum of **3qa** in $CDCl_3$. (400 MHz)

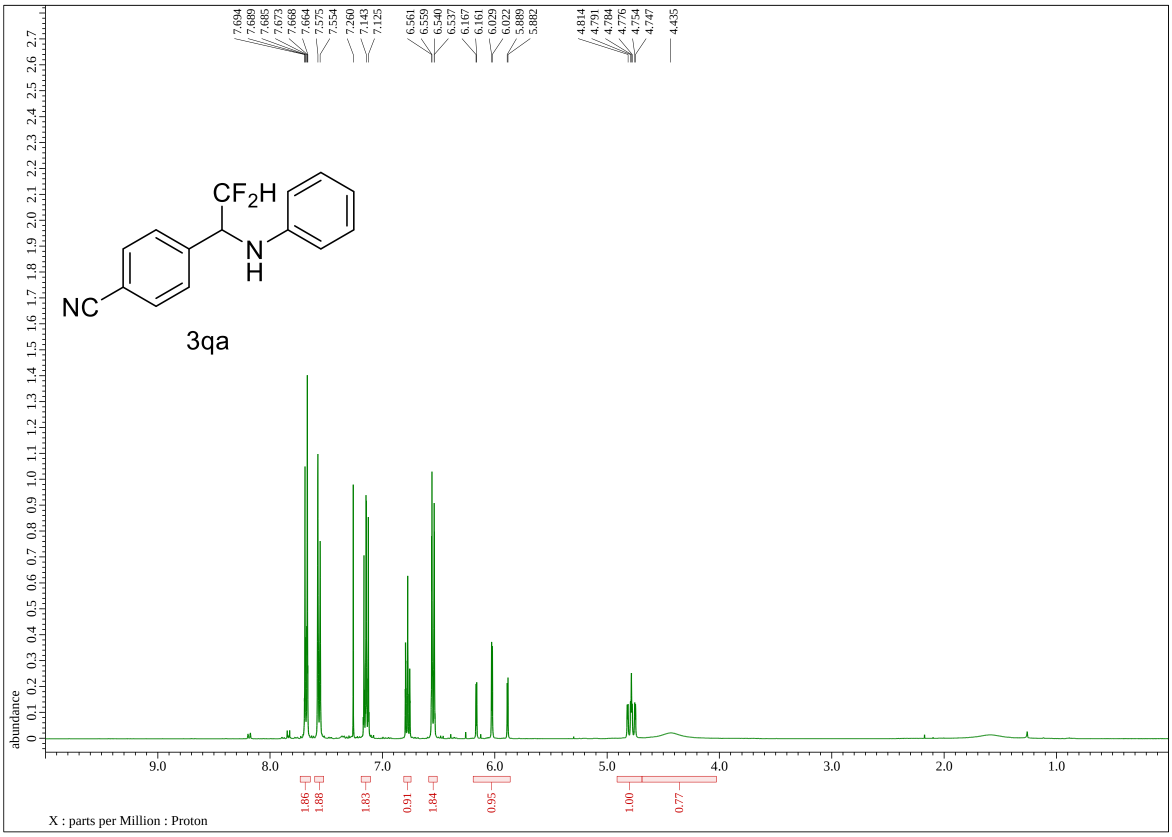

^{13}C NMR spectrum of **3qa** in $CDCl_3$. (100 MHz)

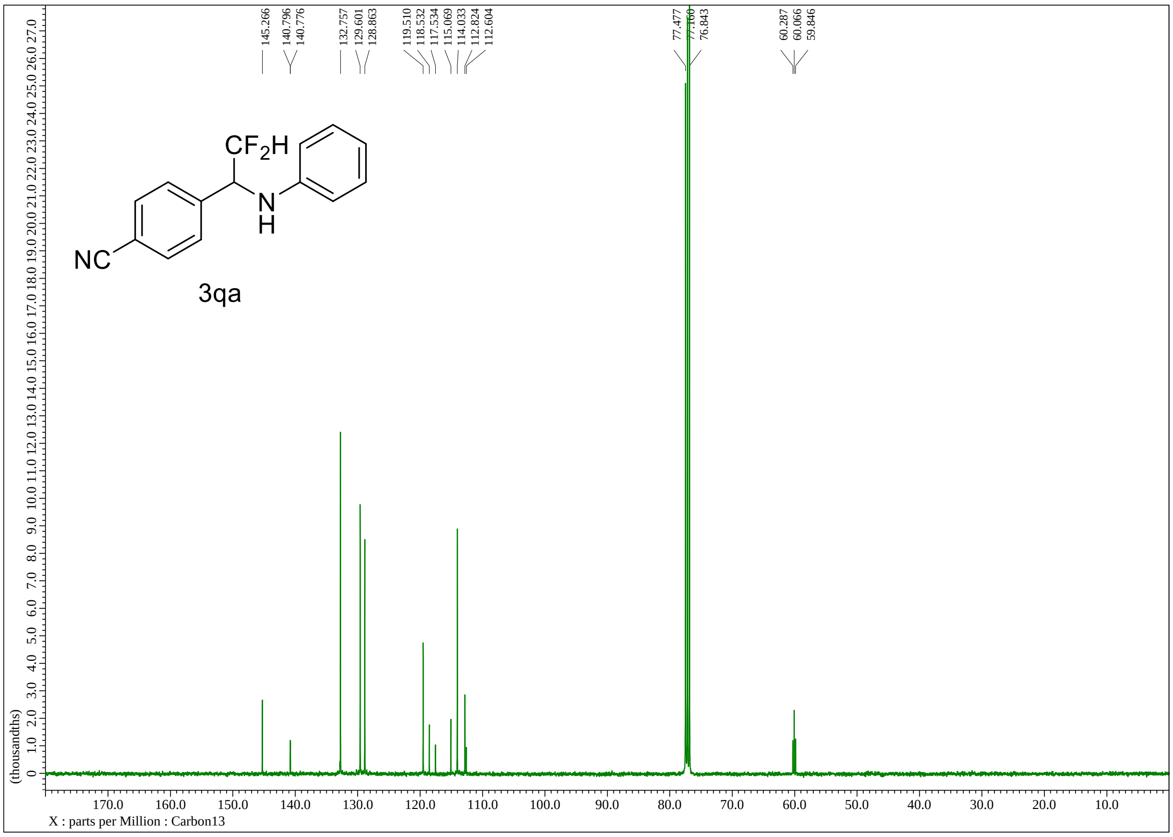

^{19}F NMR spectrum of **3qa** in $CDCl_3$. (376 MHz)

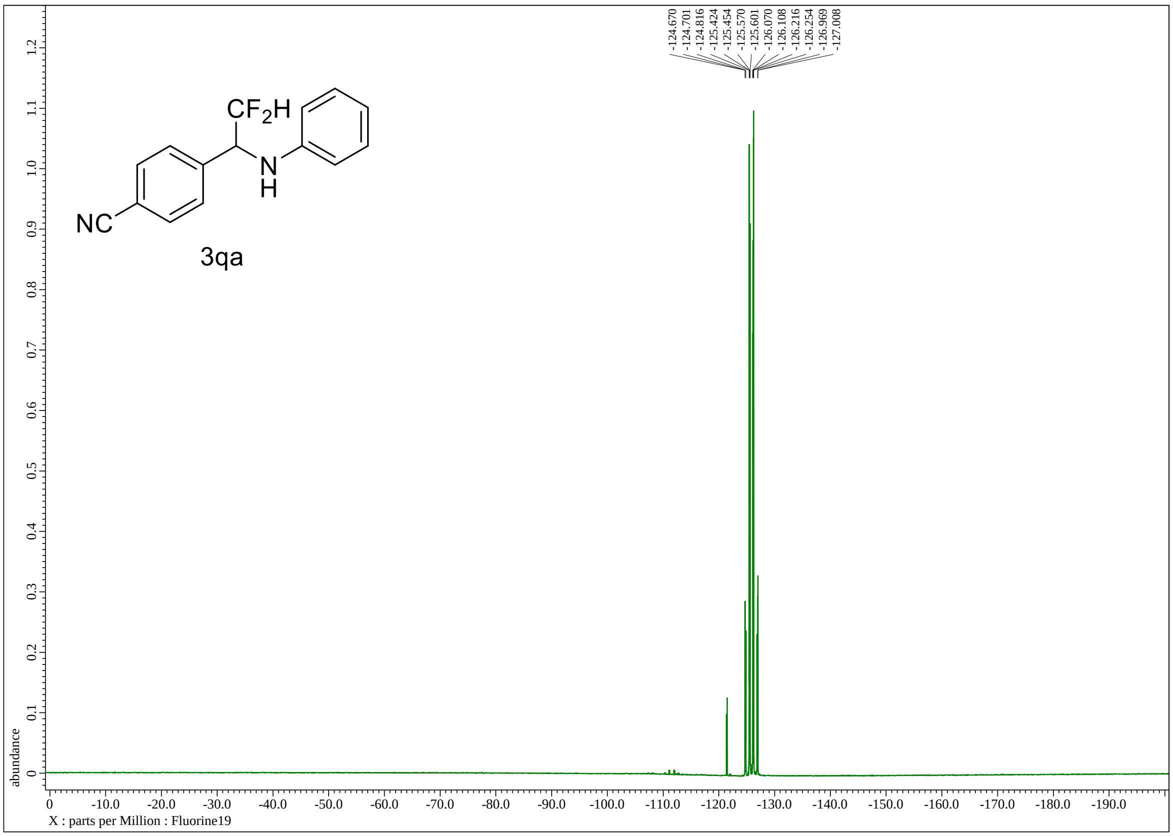

^{1}H NMR spectrum of **3ra** in $CDCl_3$. (400 MHz)

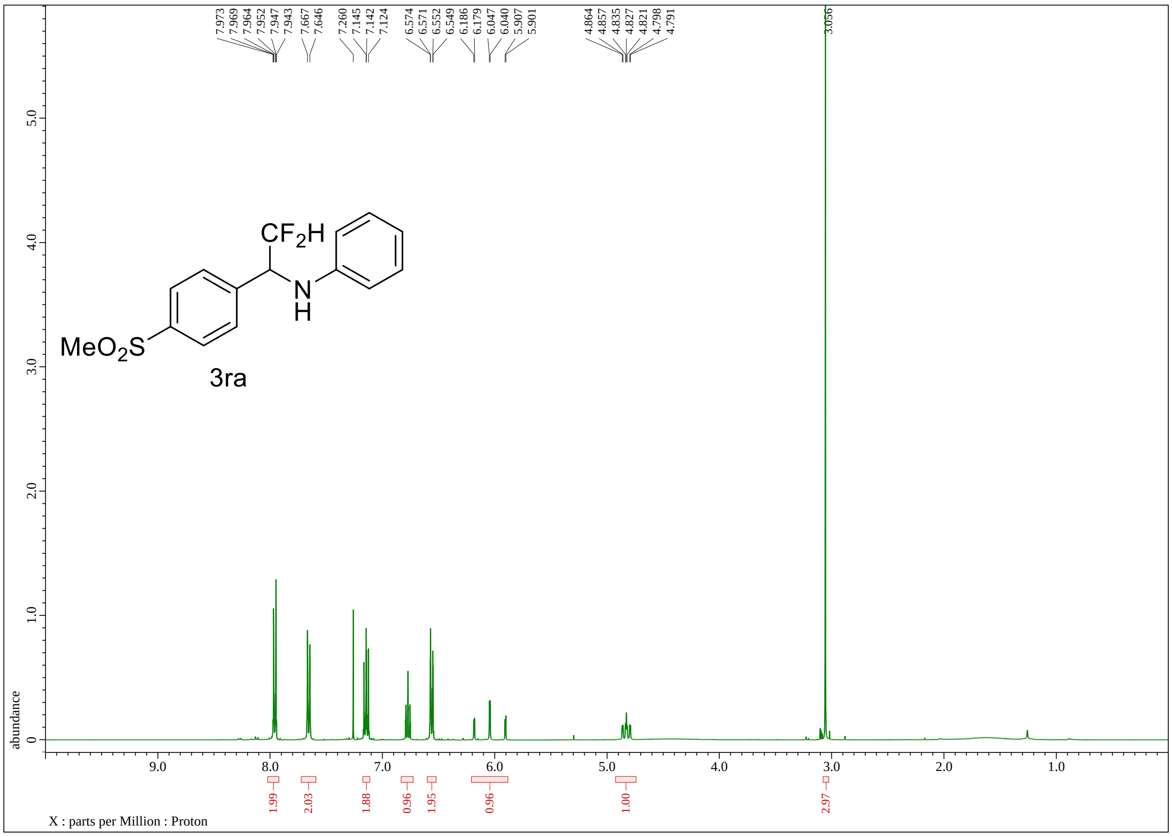

^{13}C NMR spectrum of **3ra** in $CDCl_3$. (100 MHz)

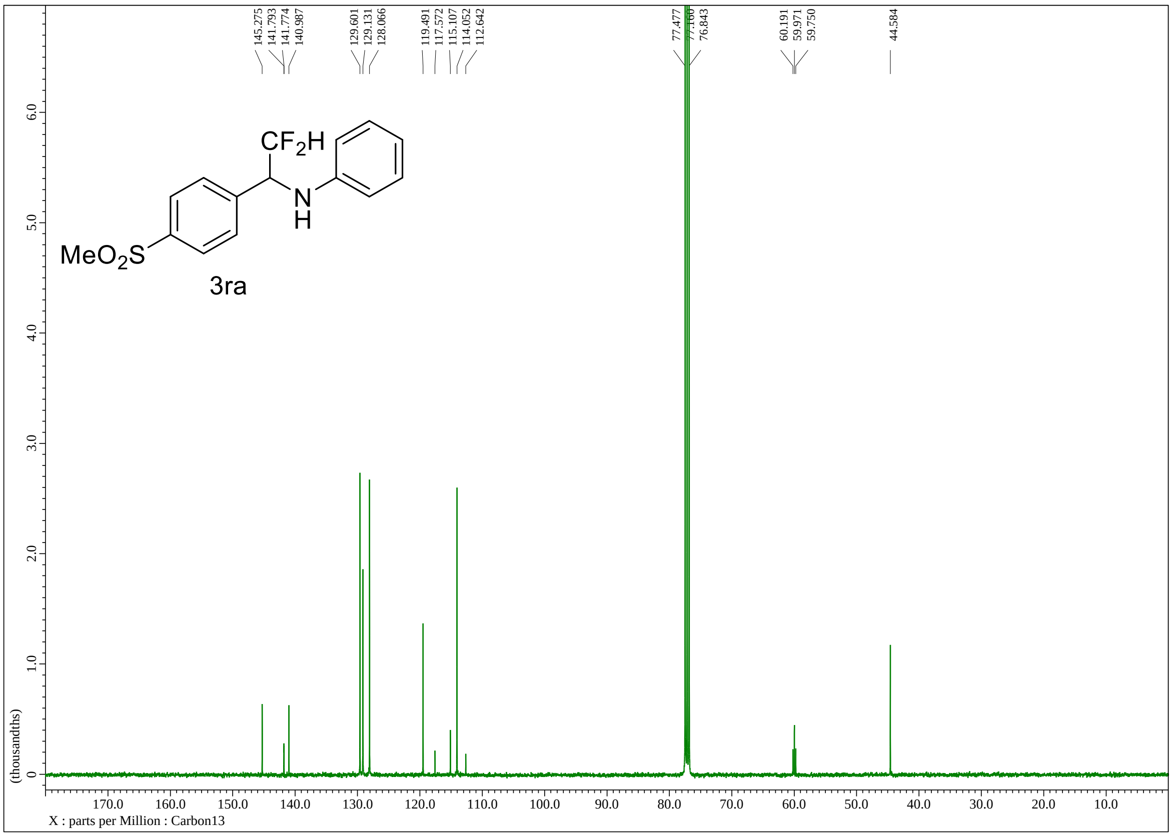

^{19}F NMR spectrum of **3ra** in $CDCl_3$. (376 MHz)

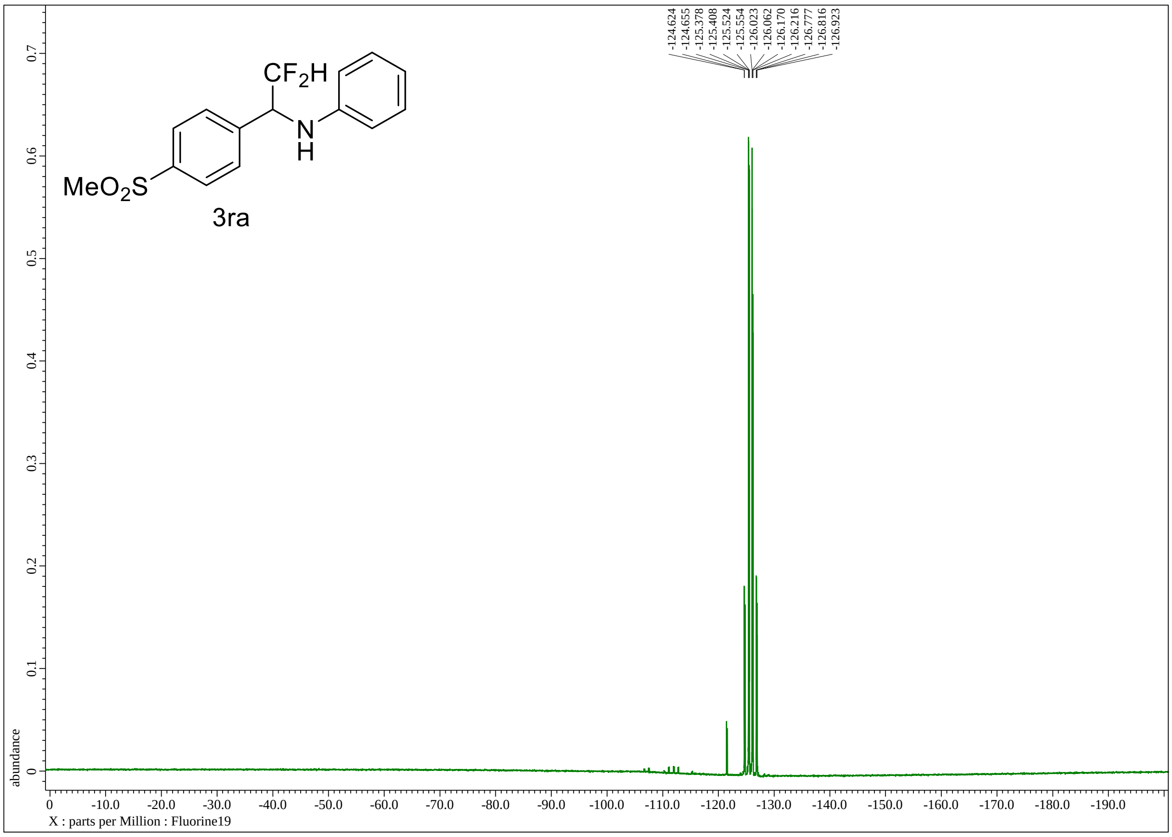

^{1}H NMR spectrum of **3sa** in $CDCl_3$. (400 MHz)

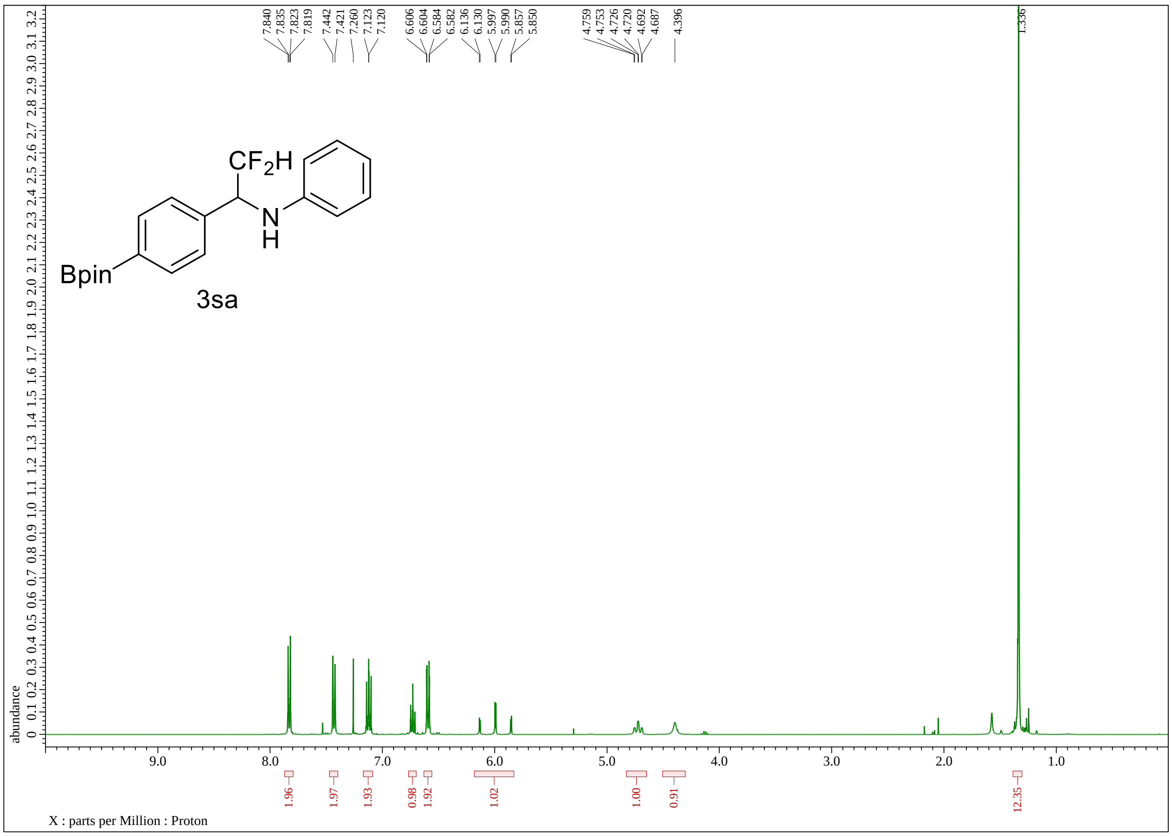

^{13}C NMR spectrum of **3sa** in $CDCl_3$. (100 MHz)

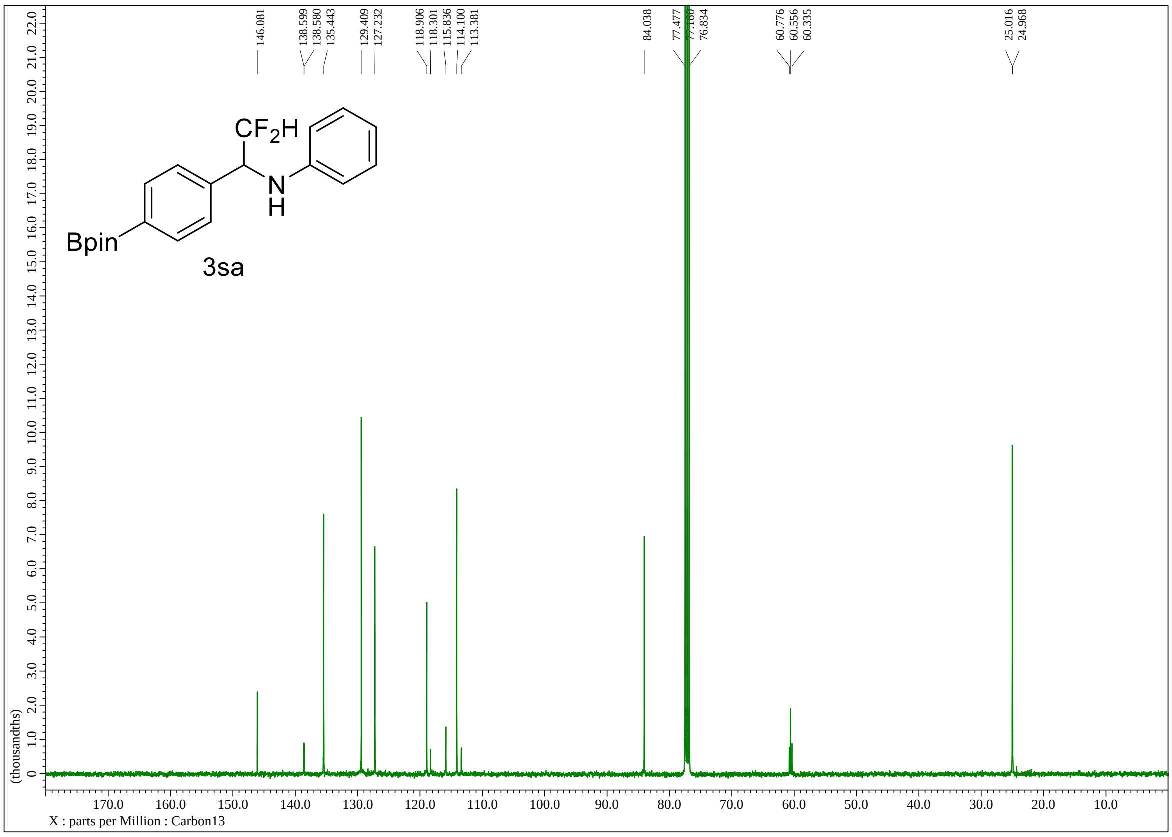

^{19}F NMR spectrum of **3sa** in $CDCl_3$. (376 MHz)

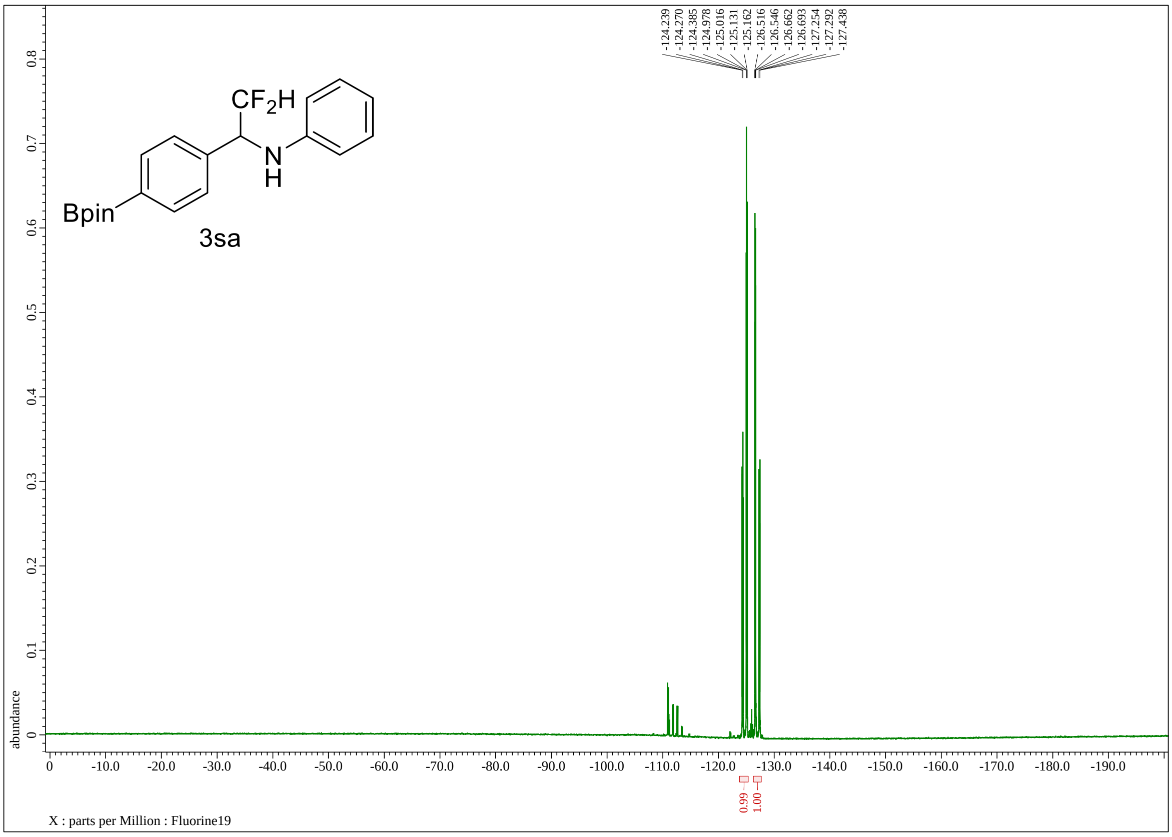

^{1}H NMR spectrum of **3ab** in $CDCl_3$. (400 MHz)

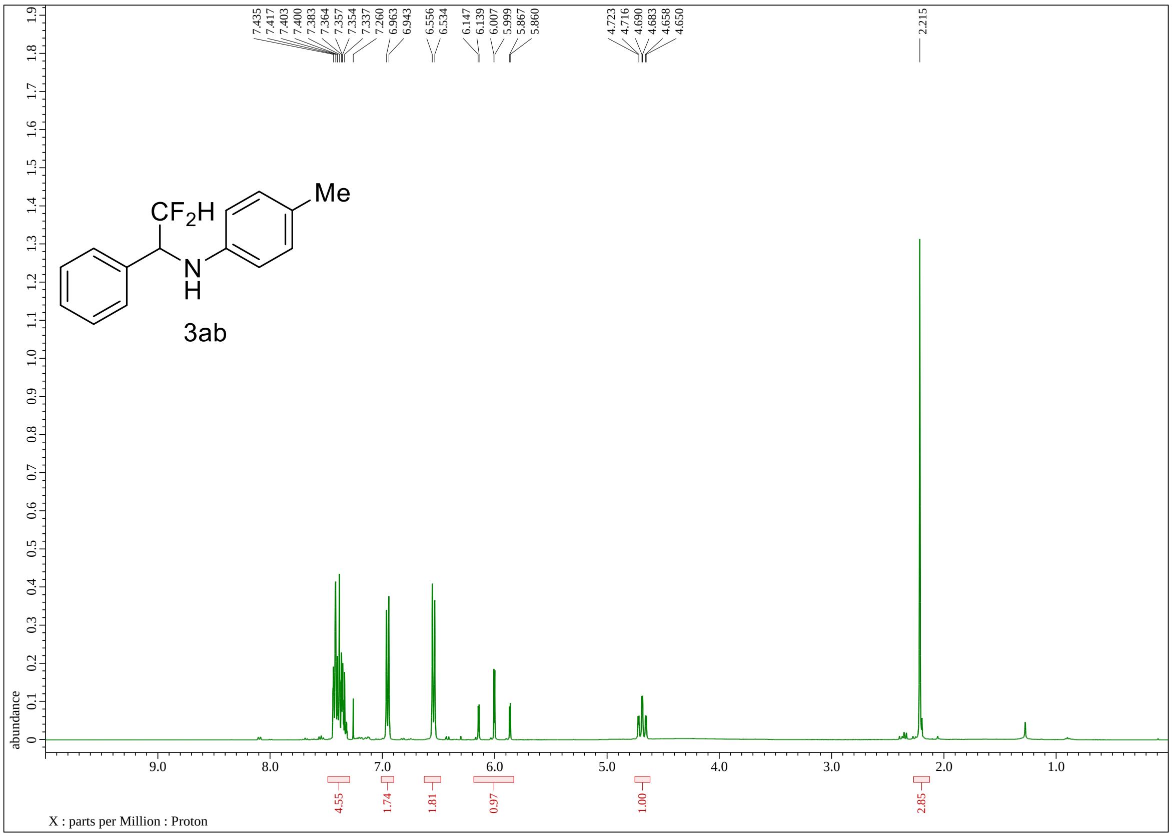

^{13}C NMR spectrum of **3ab** in $CDCl_3$. (100 MHz)

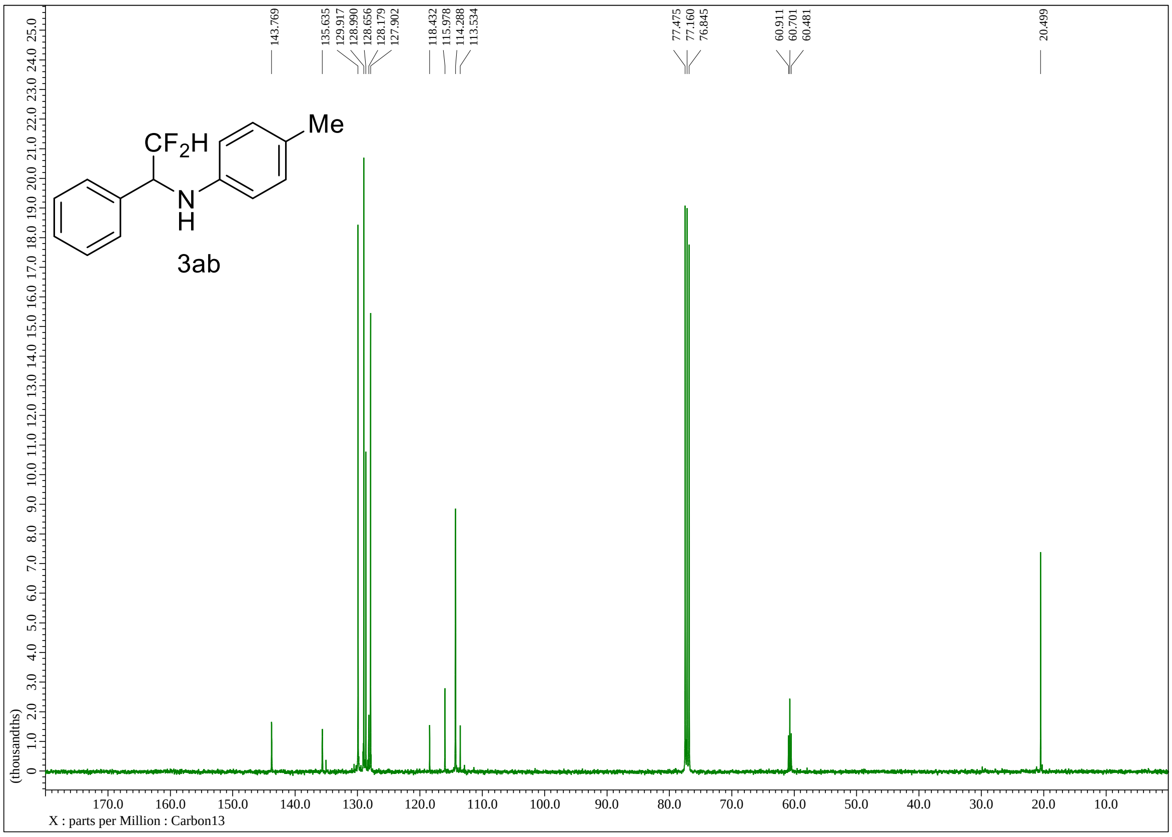

^{19}F NMR spectrum of **3ab** in $CDCl_3$. (376 MHz)

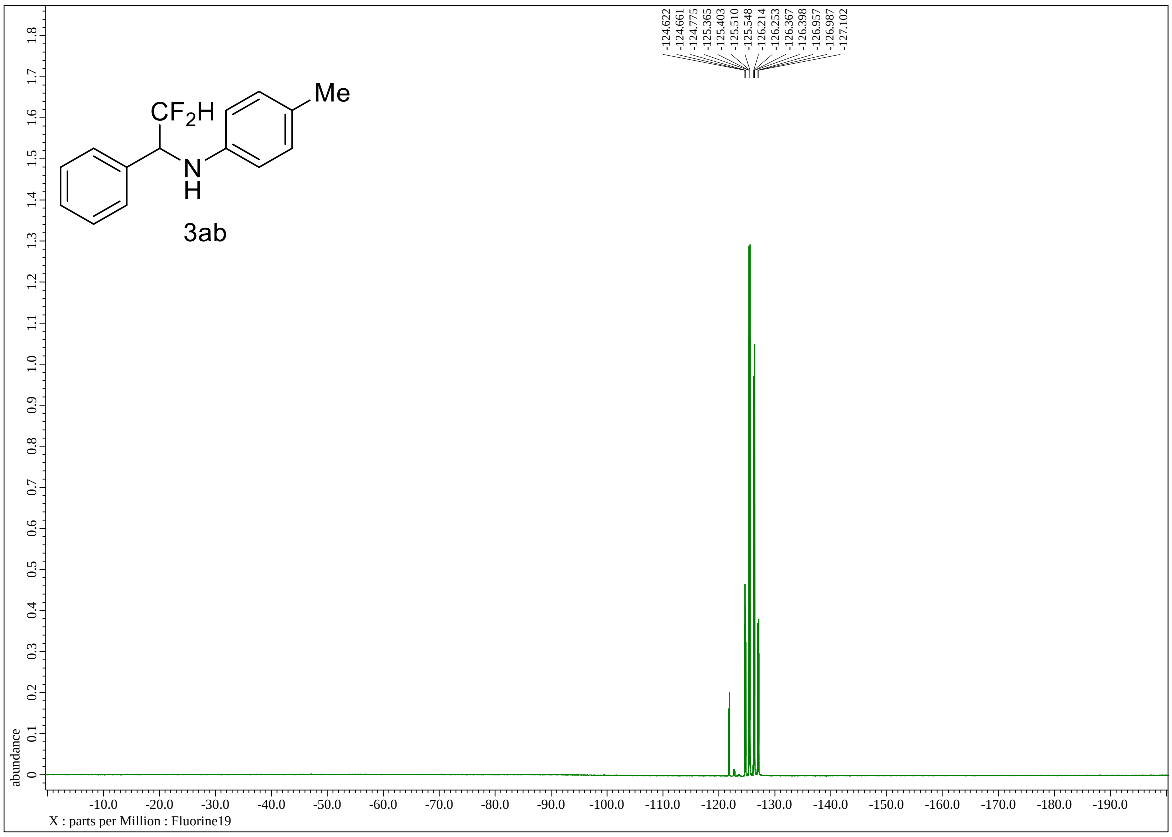

^{1}H NMR spectrum of **3ac** in $CDCl_3$. (400 MHz)

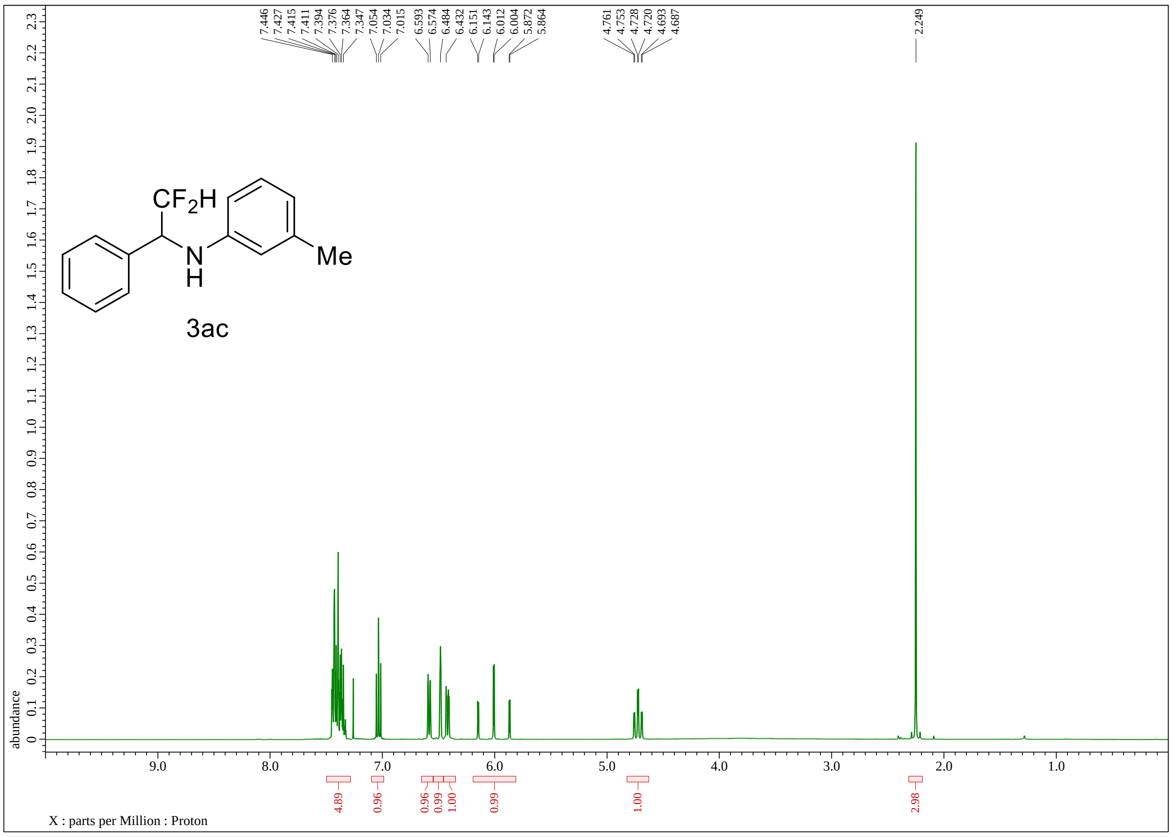

^{13}C NMR spectrum of **3ac** in $CDCl_3$. (100 MHz)

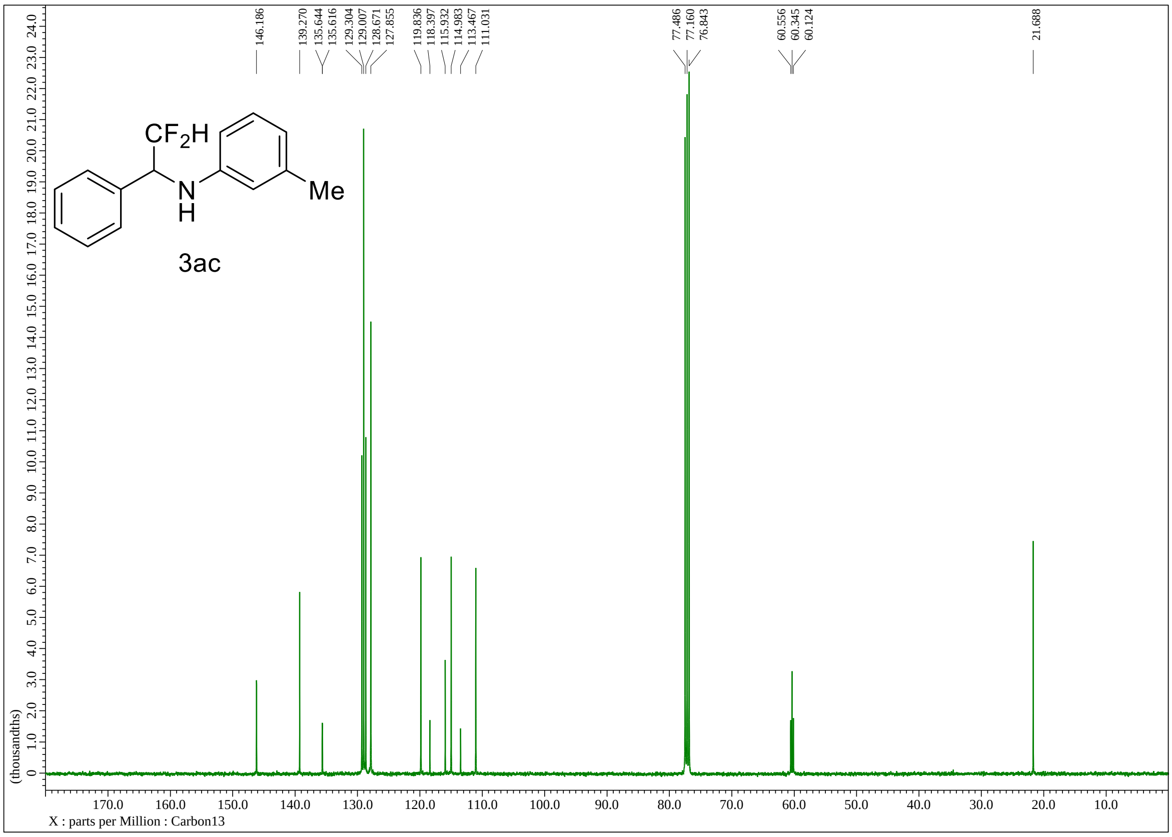

^{19}F NMR spectrum of **3ac** in $CDCl_3$. (376 MHz)

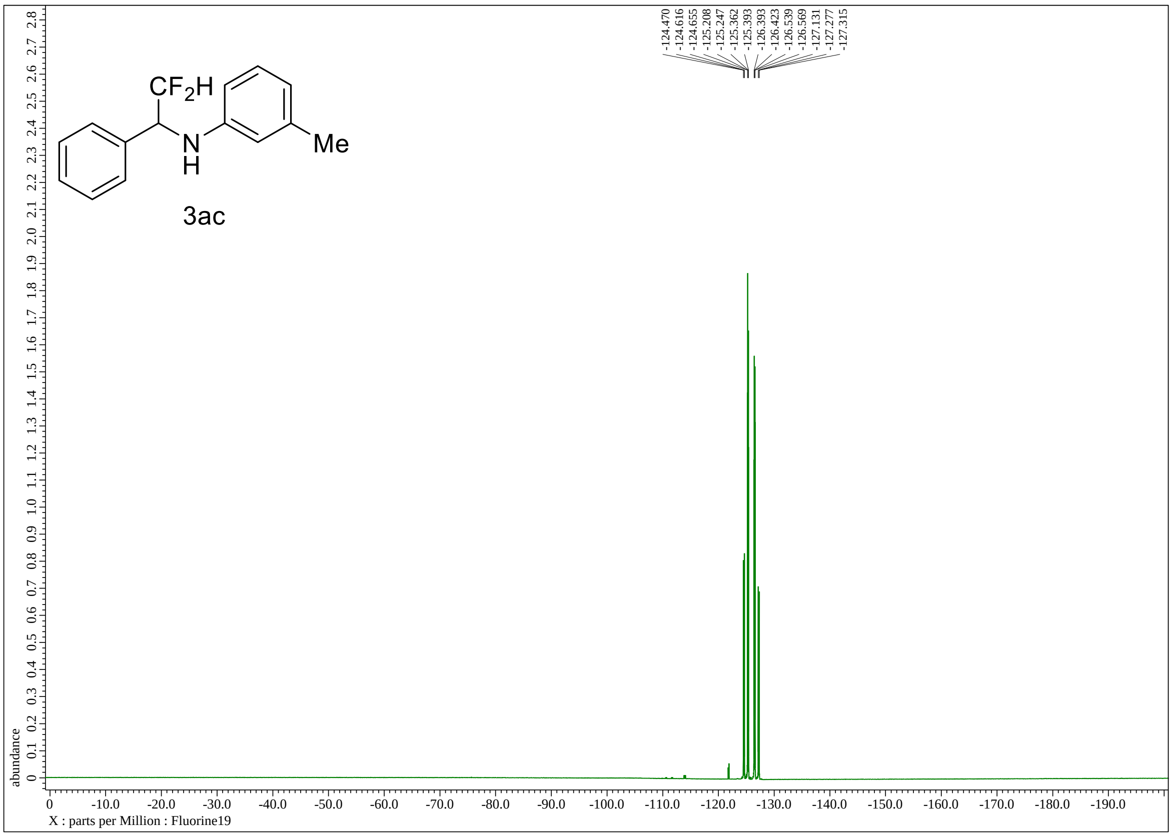

^{1}H NMR spectrum of **3ad** in $CDCl_3$. (400 MHz)

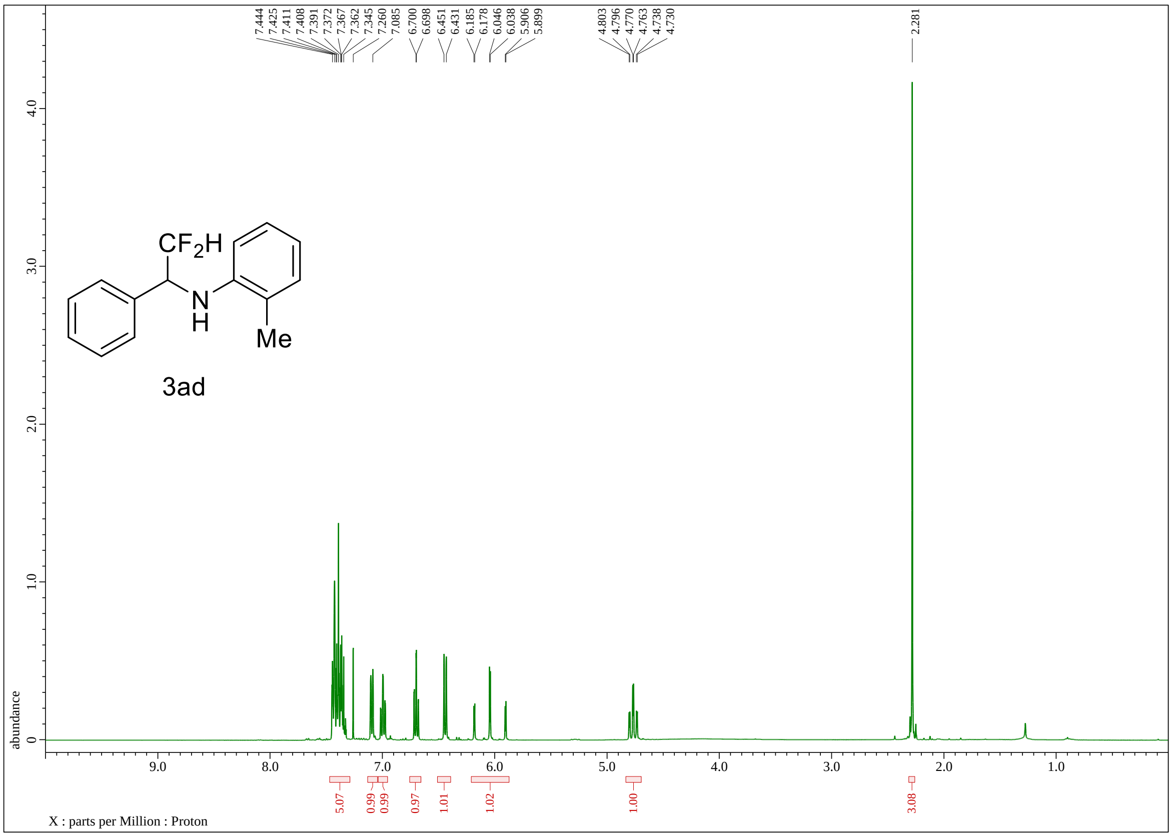

^{13}C NMR spectrum of **3ad** in $CDCl_3$. (100 MHz)

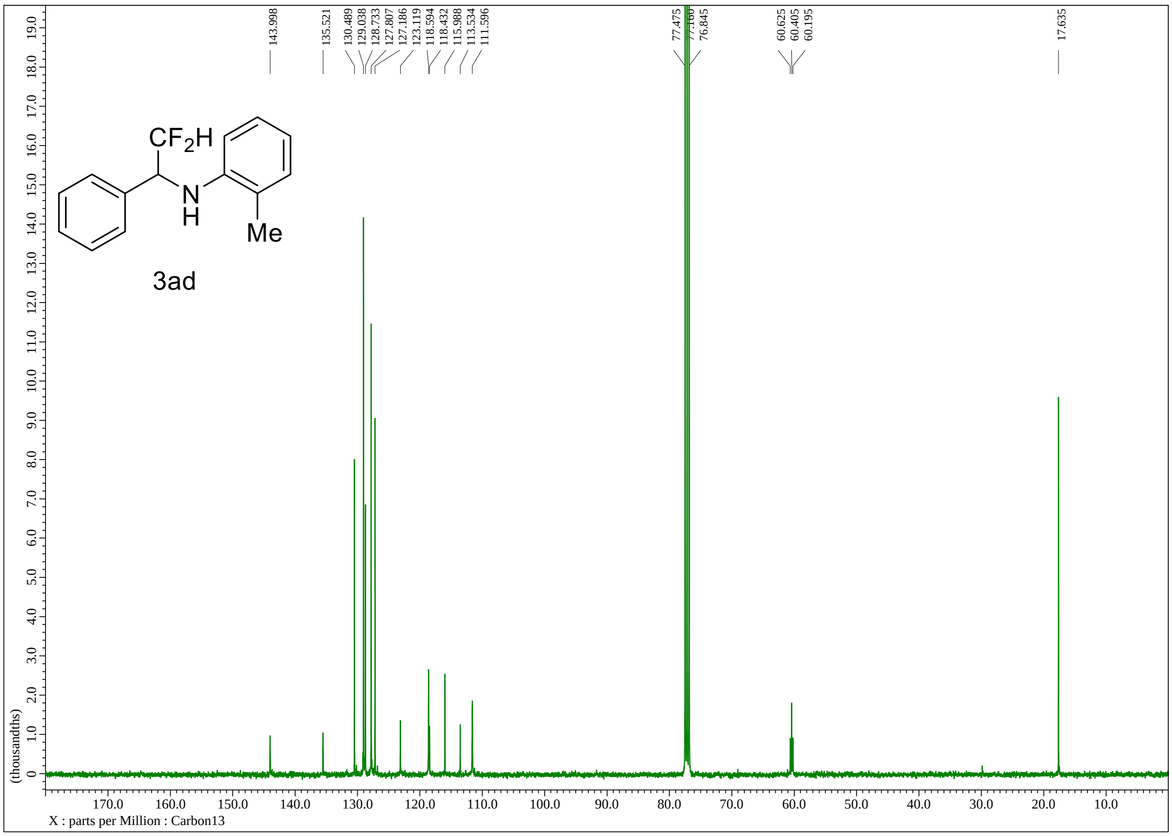

^{19}F NMR spectrum of **3ad** in $CDCl_3$. (376 MHz)

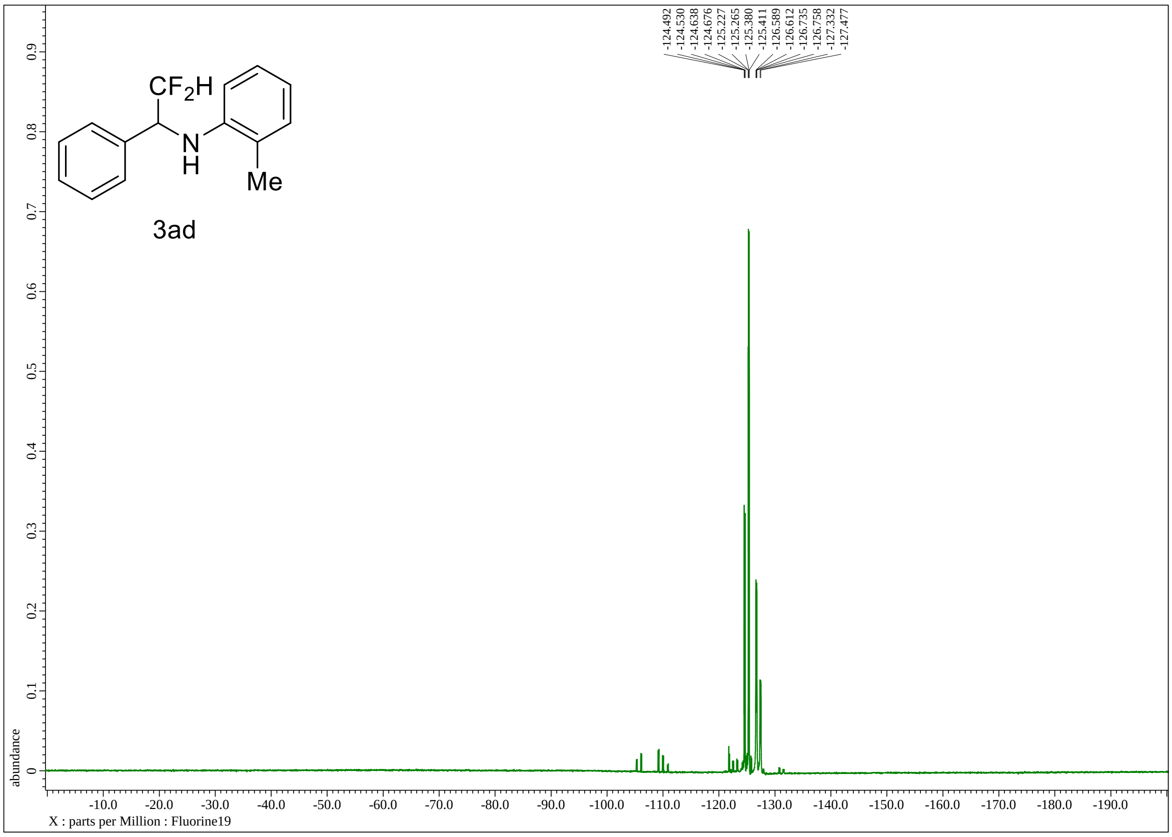

^{1}H NMR spectrum of **3ae** in $CDCl_3$. (400 MHz)

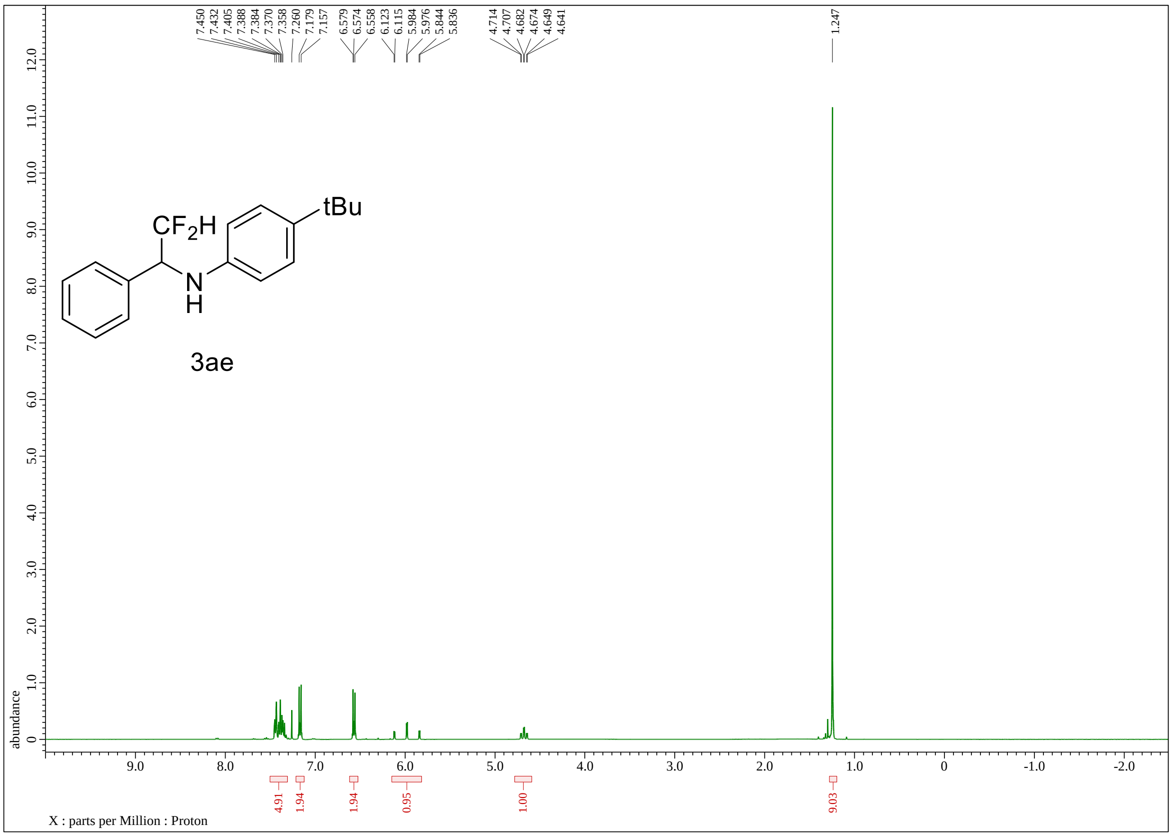

^{13}C NMR spectrum of **3ae** in $CDCl_3$. (100 MHz)

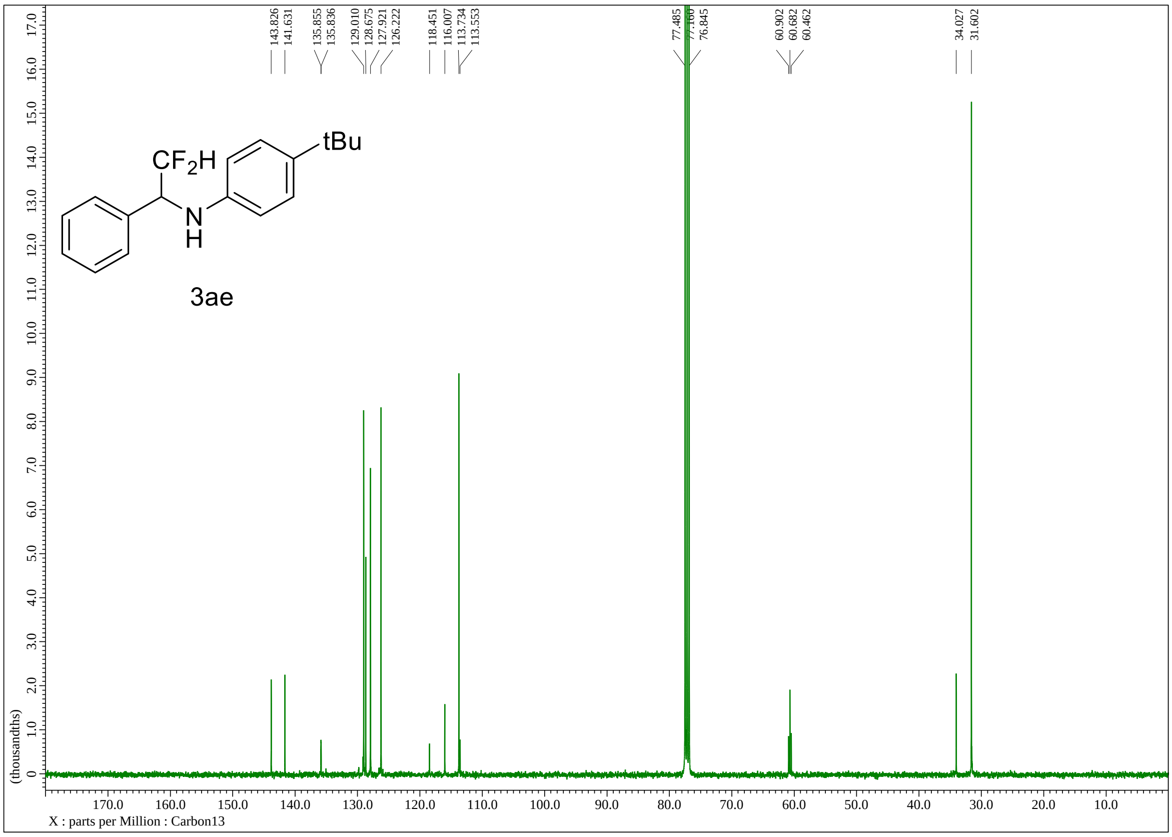

^{19}F NMR spectrum of **3ae** in $CDCl_3$. (376 MHz)

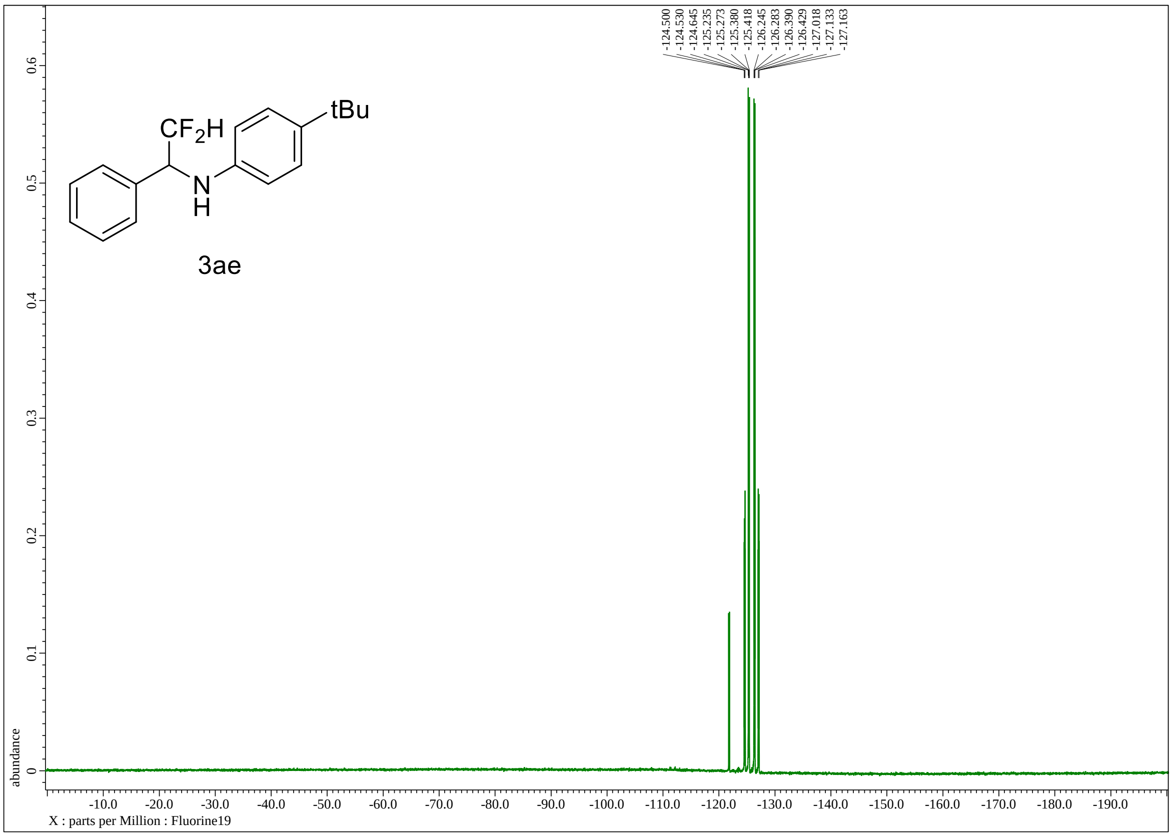

^{1}H NMR spectrum of **3af** in $CDCl_3$. (400 MHz)

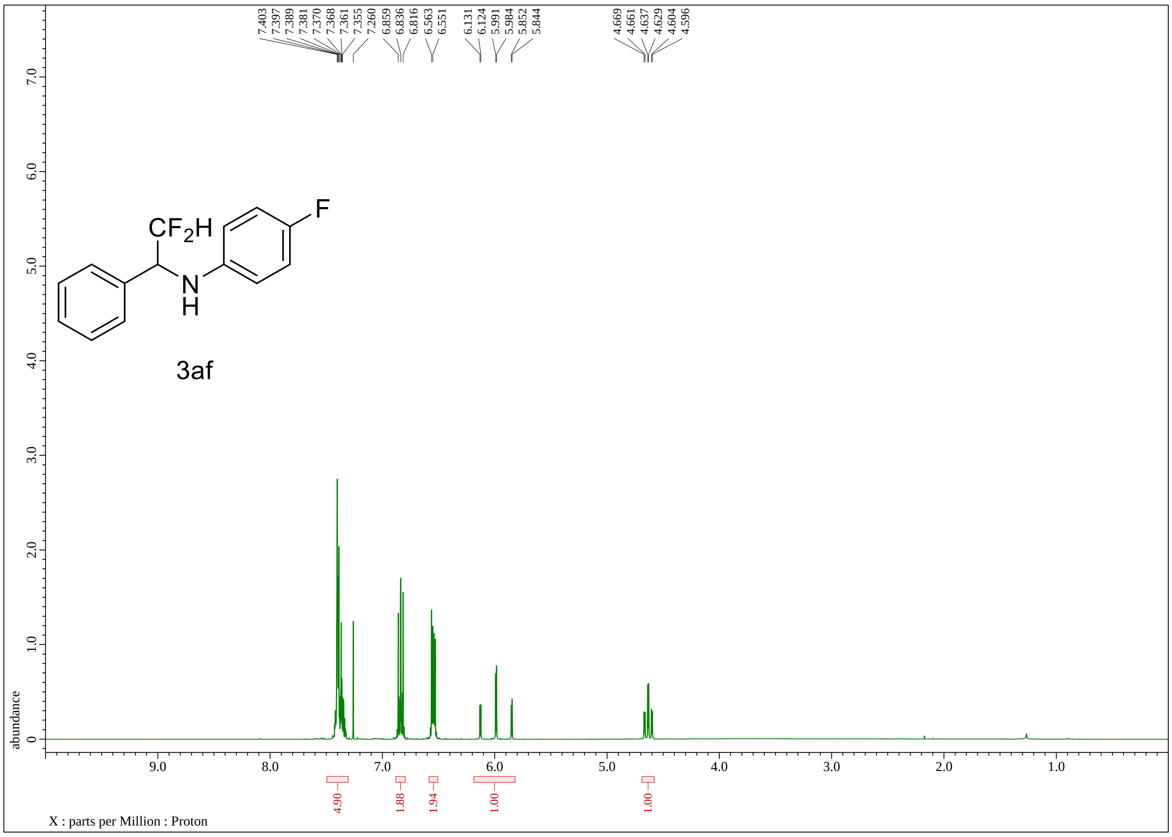

^{13}C NMR spectrum of **3af** in $CDCl_3$. (100 MHz)

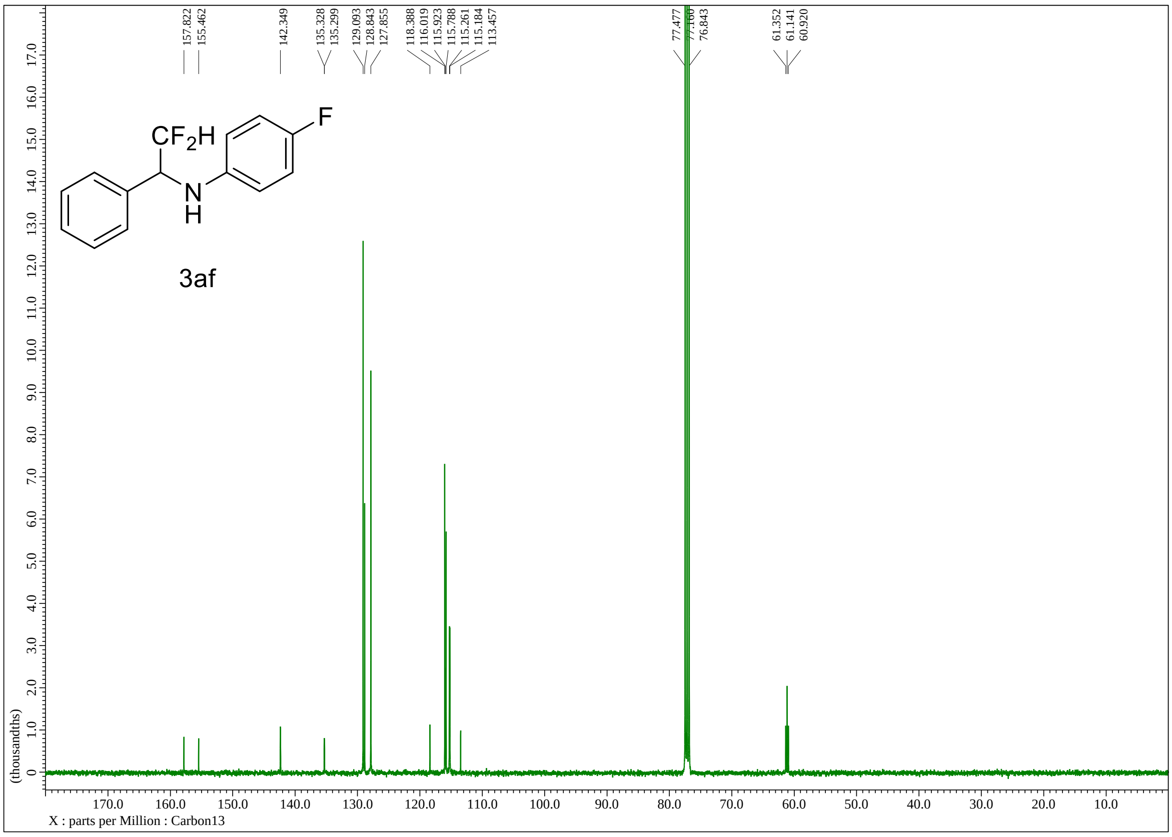

^{19}F NMR spectrum of **3af** in $CDCl_3$. (376 MHz)

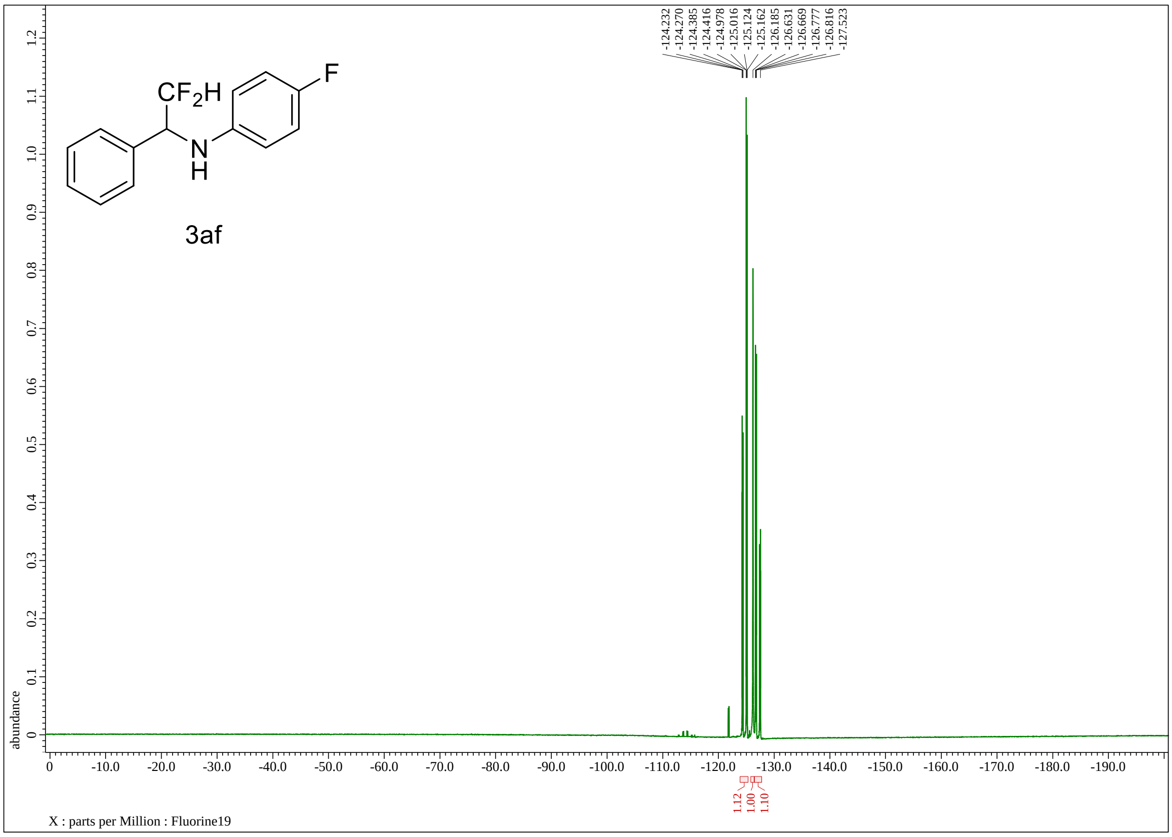

^{1}H NMR spectrum of **3ag** in $CDCl_3$. (400 MHz)

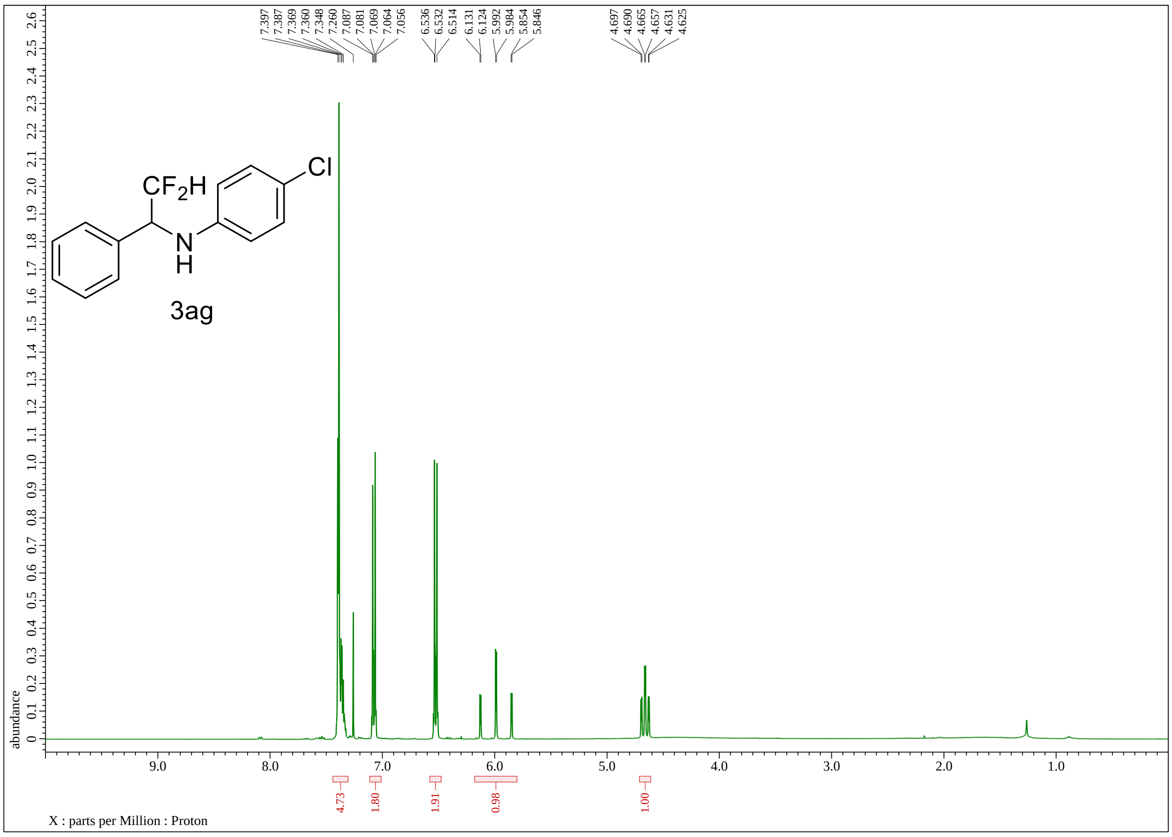

^{13}C NMR spectrum of **3ag** in $CDCl_3$. (100 MHz)

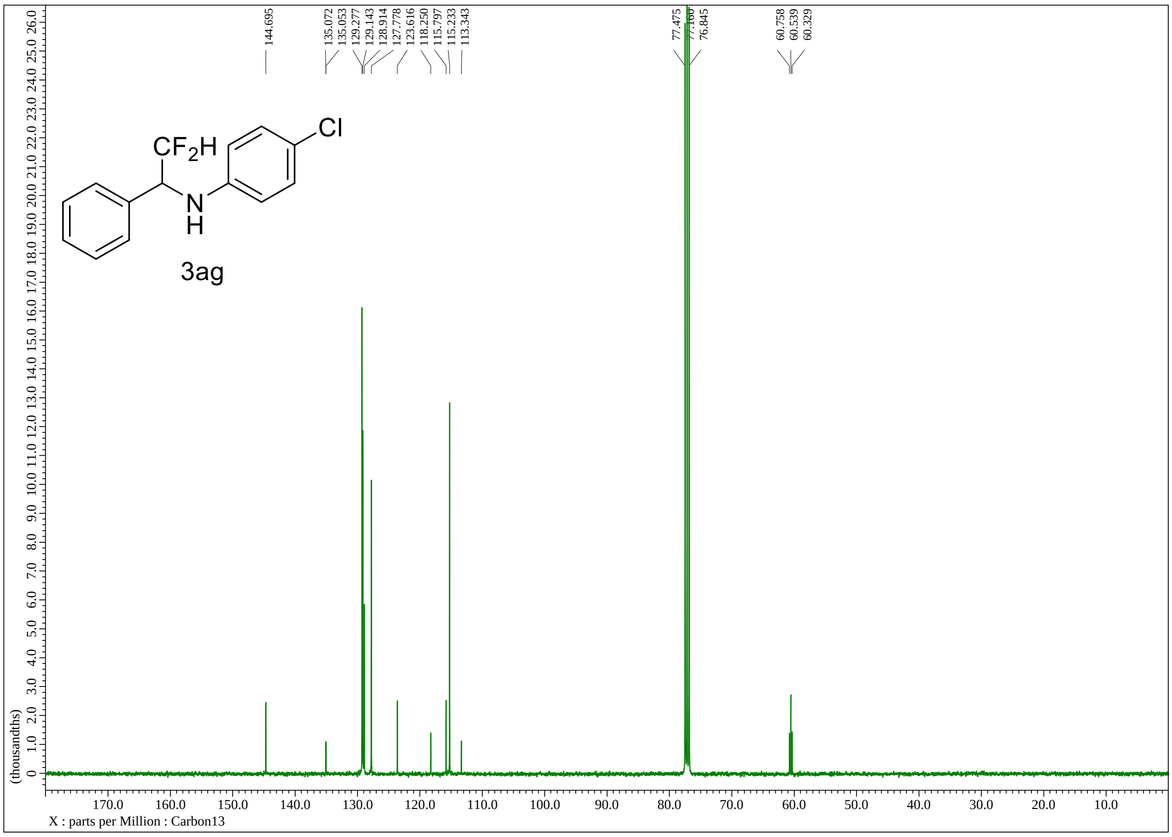

^{19}F NMR spectrum of **3ag** in $CDCl_3$. (376 MHz)

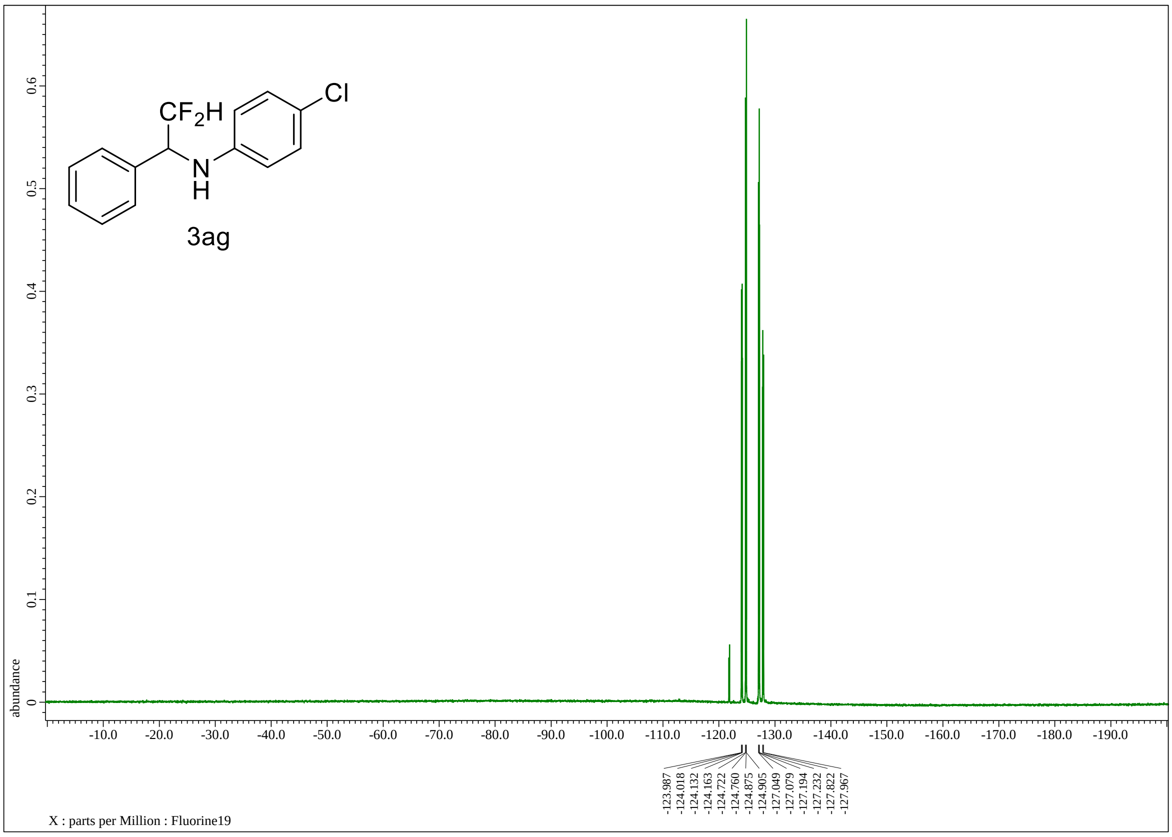

^{1}H NMR spectrum of **3ah** in $CDCl_3$. (400 MHz)

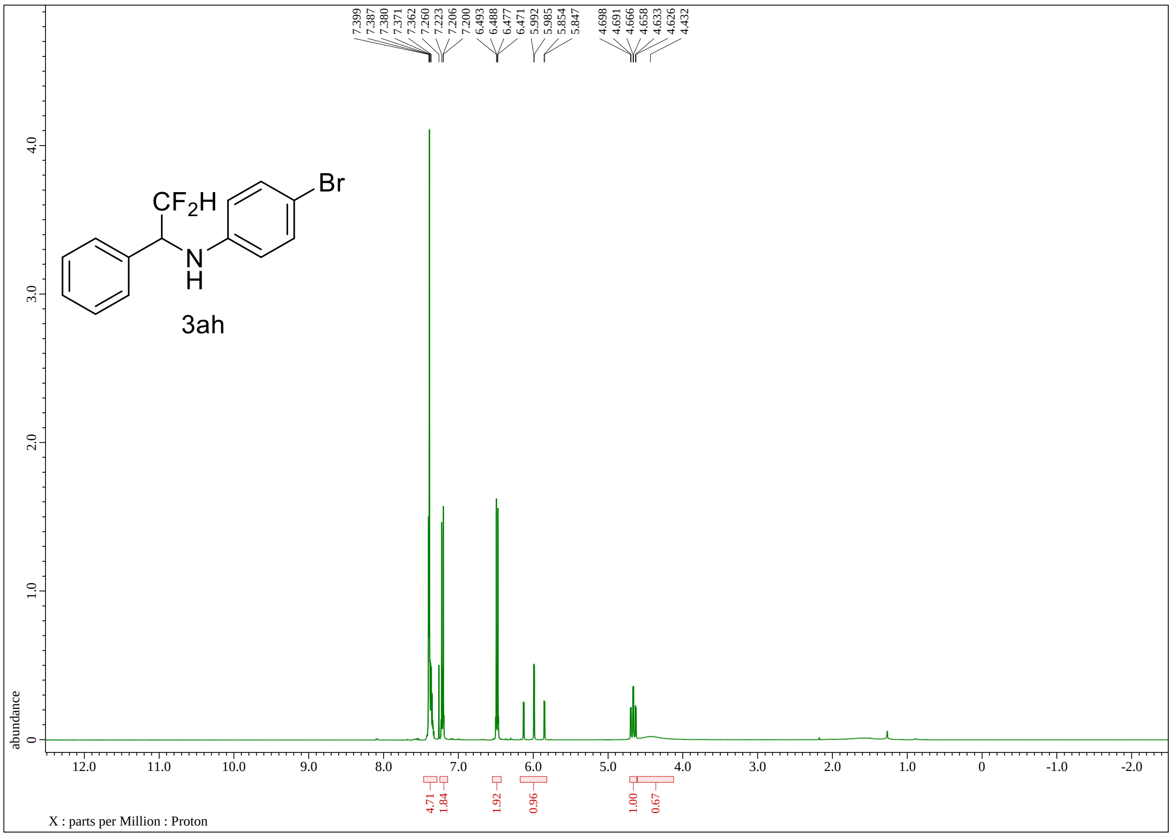

^{13}C NMR spectrum of **3ah** in $CDCl_3$. (100 MHz)

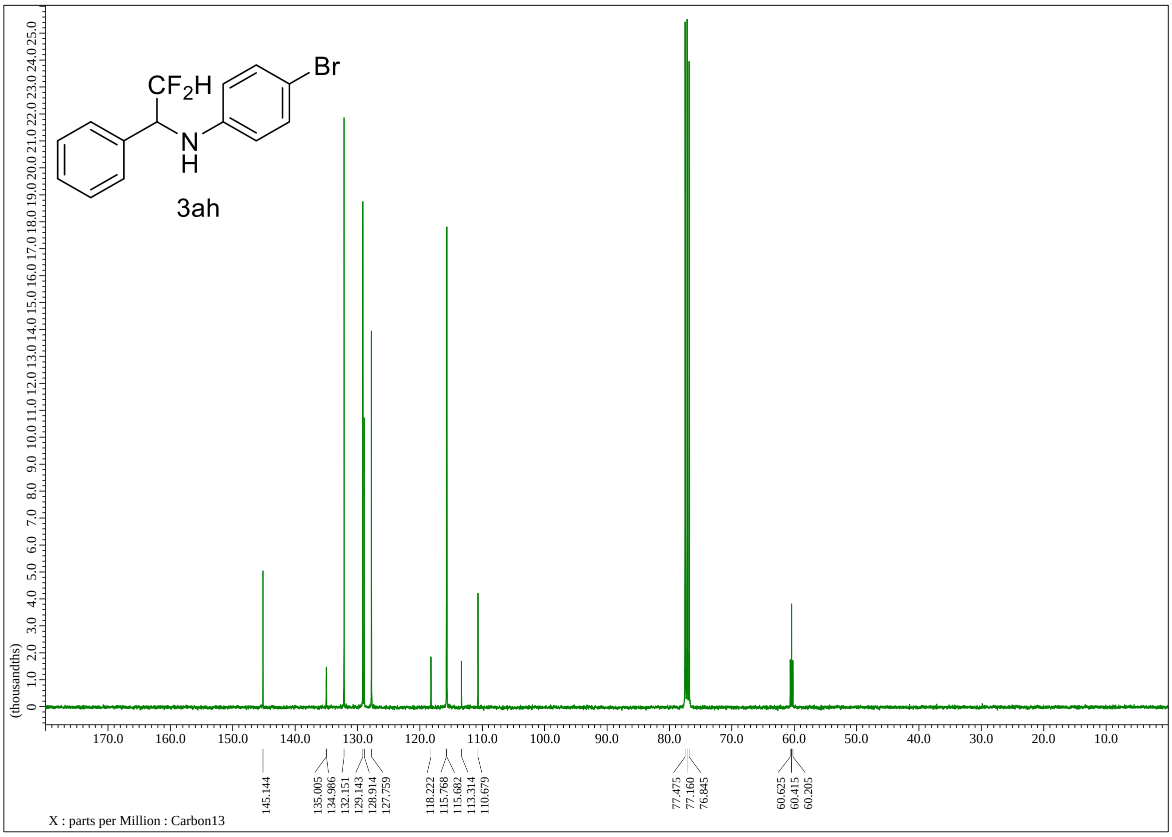

^{19}F NMR spectrum of **3ah** in $CDCl_3$. (376 MHz)

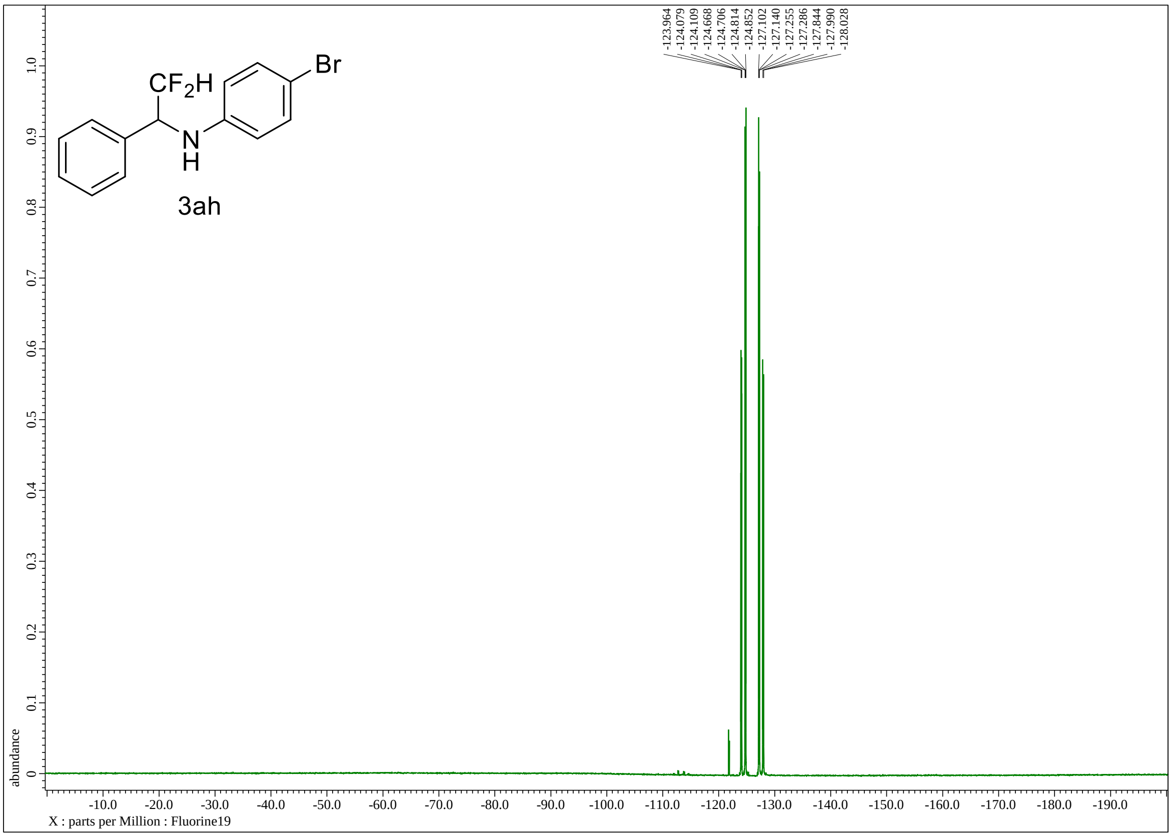

^{1}H NMR spectrum of **3ai** in $CDCl_3$. (400 MHz)

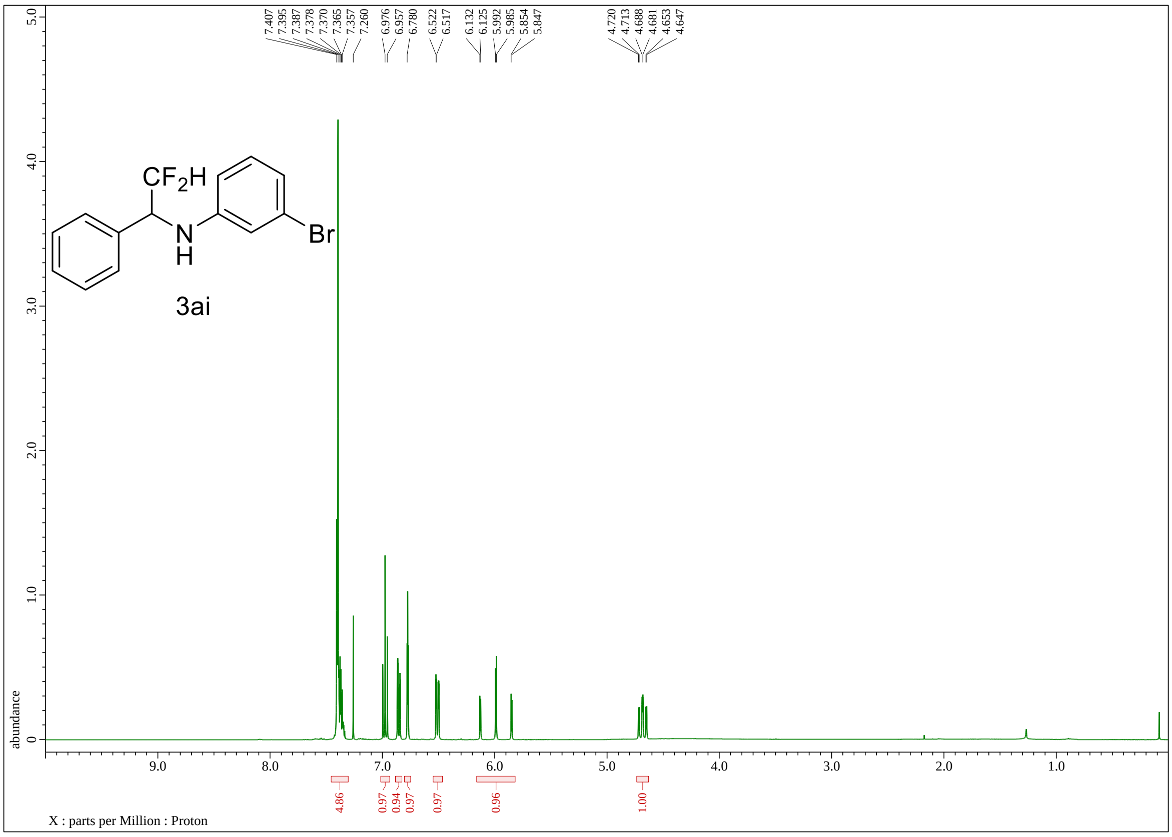

^{13}C NMR spectrum of **3ai** in $CDCl_3$. (100 MHz)

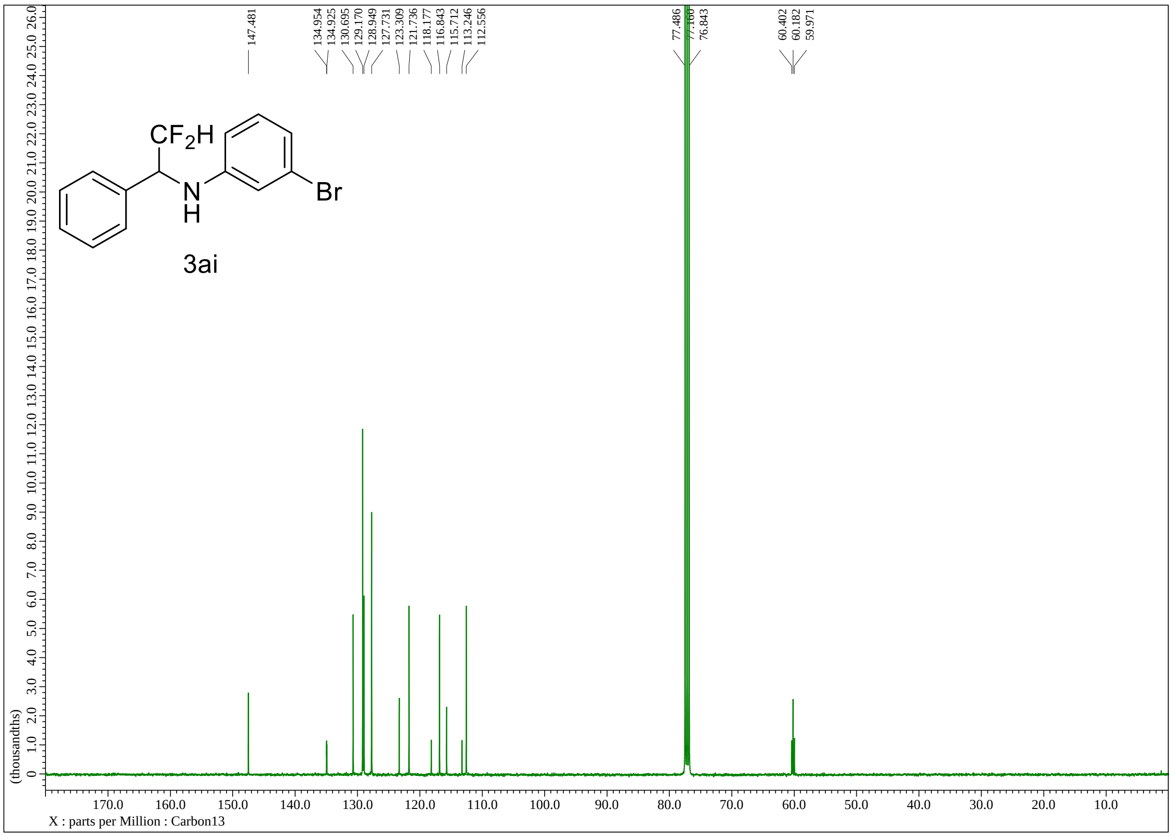

^{19}F NMR spectrum of **3ai** in $CDCl_3$. (376 MHz)

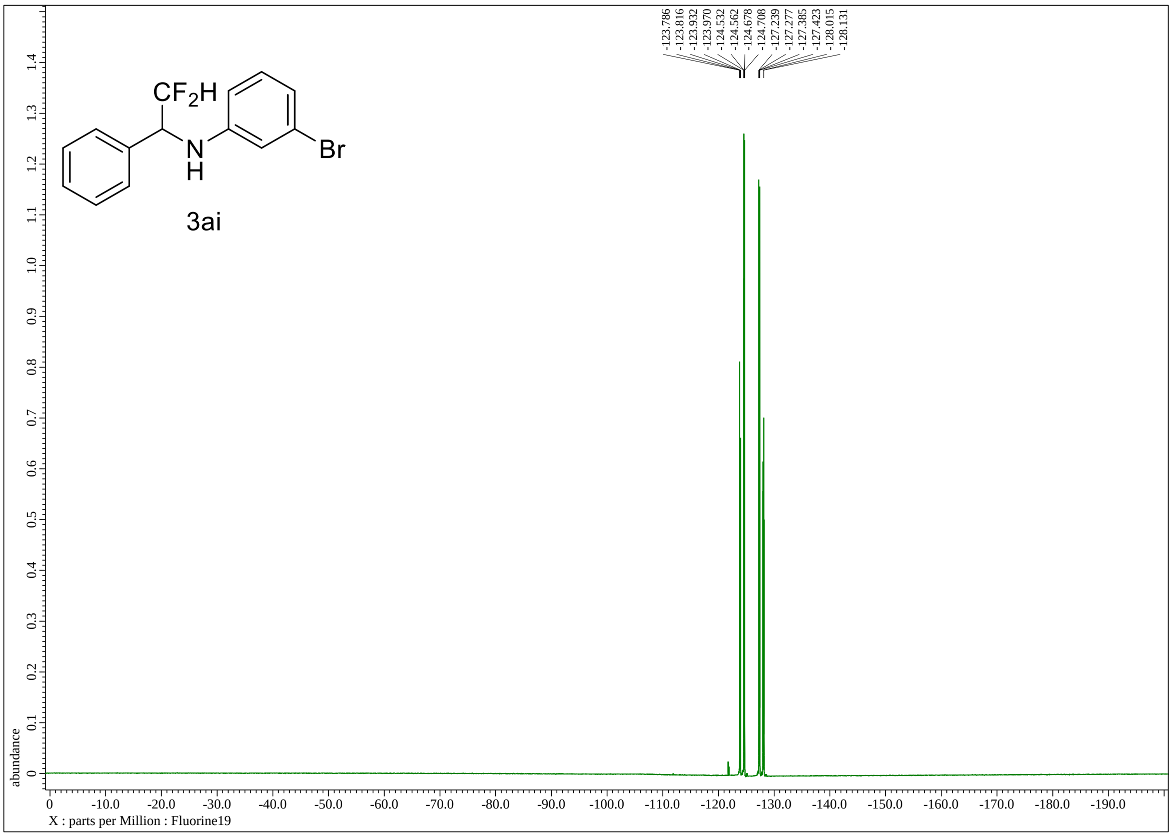

^{1}H NMR spectrum of **3aj** in $CDCl_3$. (400 MHz)

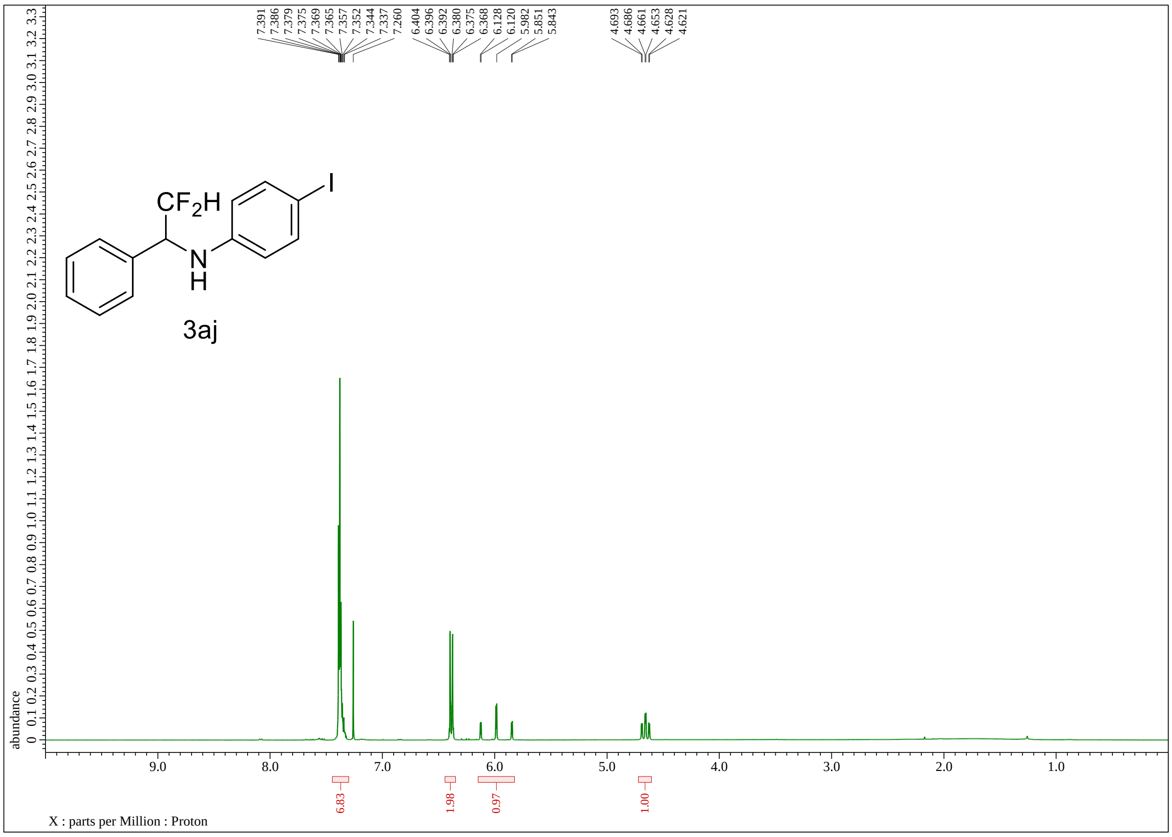

^{13}C NMR spectrum of **3aj** in $CDCl_3$. (100 MHz)

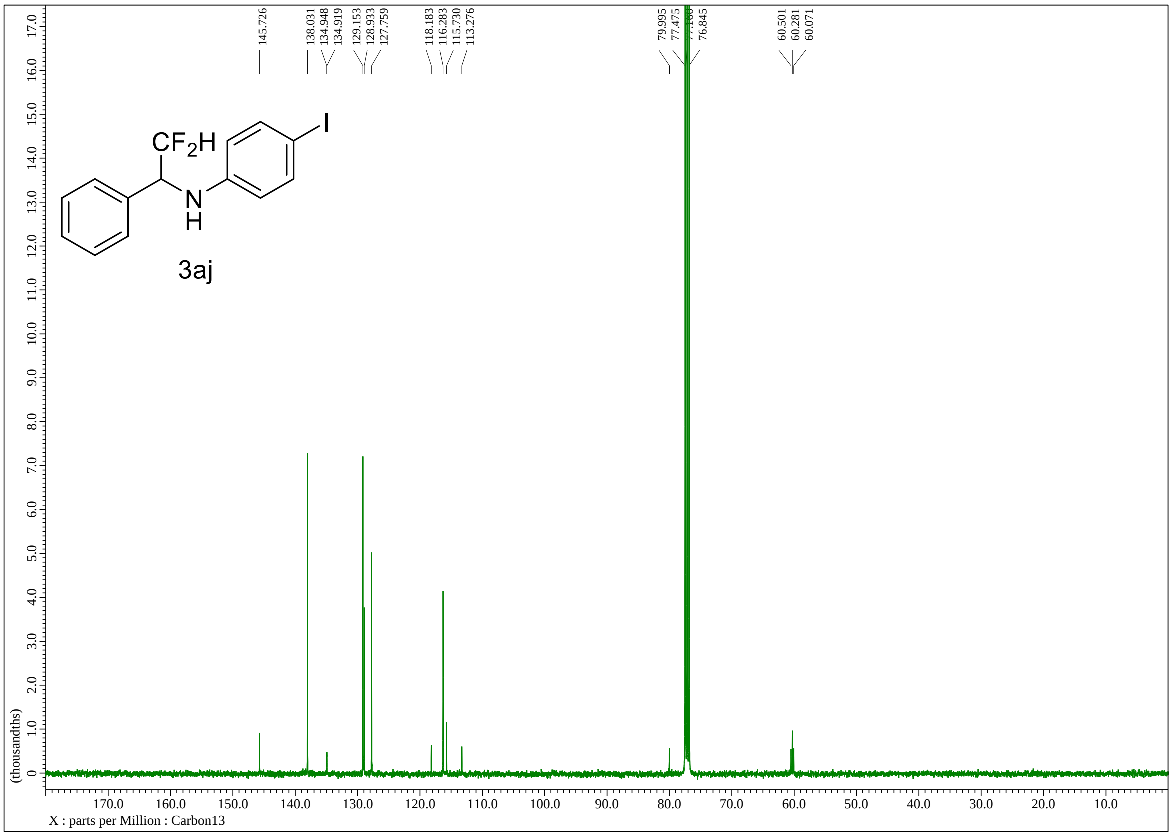

^{19}F NMR spectrum of **3aj** in $CDCl_3$. (376 MHz)

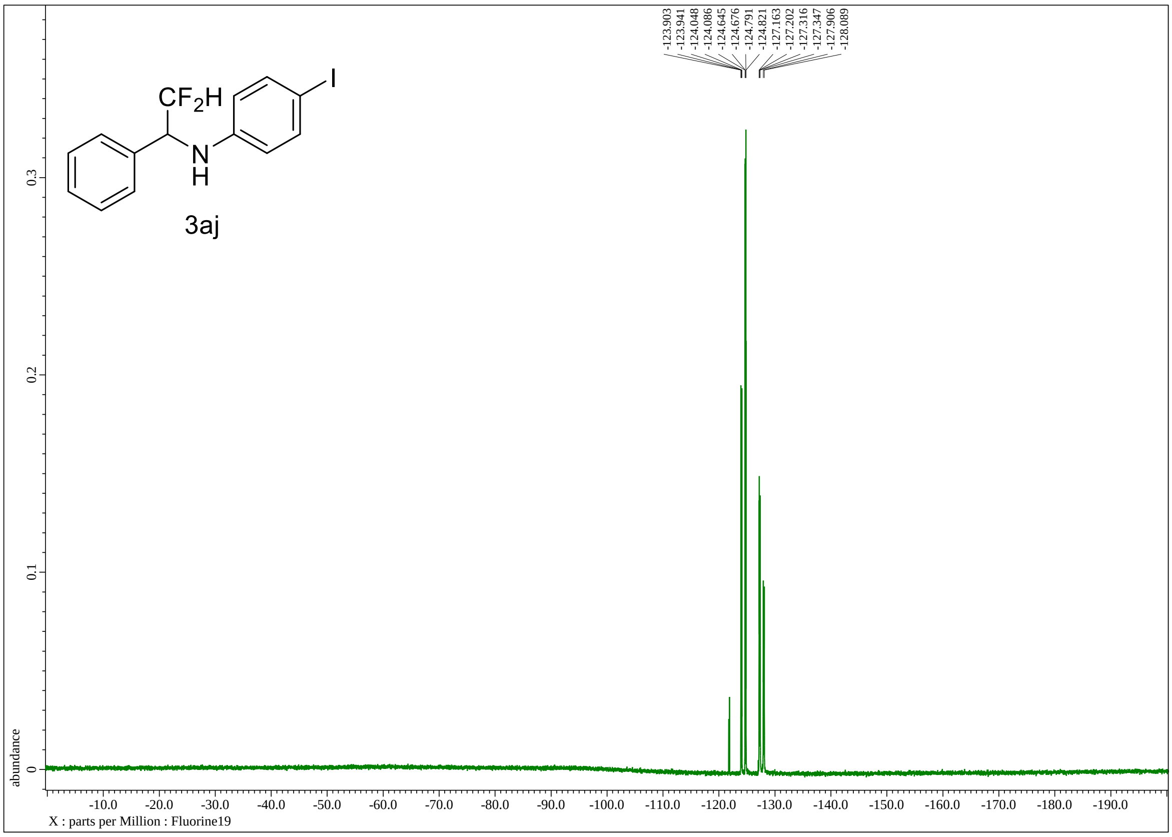

^{1}H NMR spectrum of **3ak** in $CDCl_3$. (400 MHz)

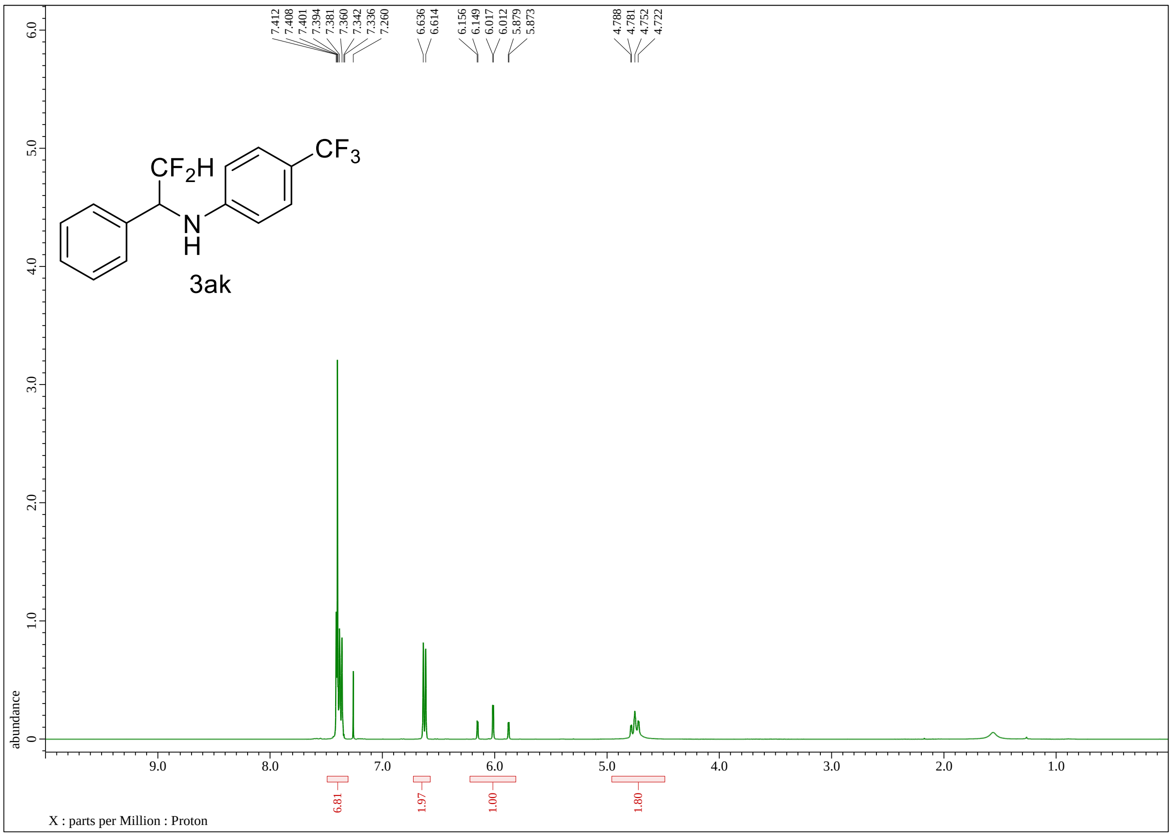

^{13}C NMR spectrum of **3ak** in $CDCl_3$. (100 MHz)

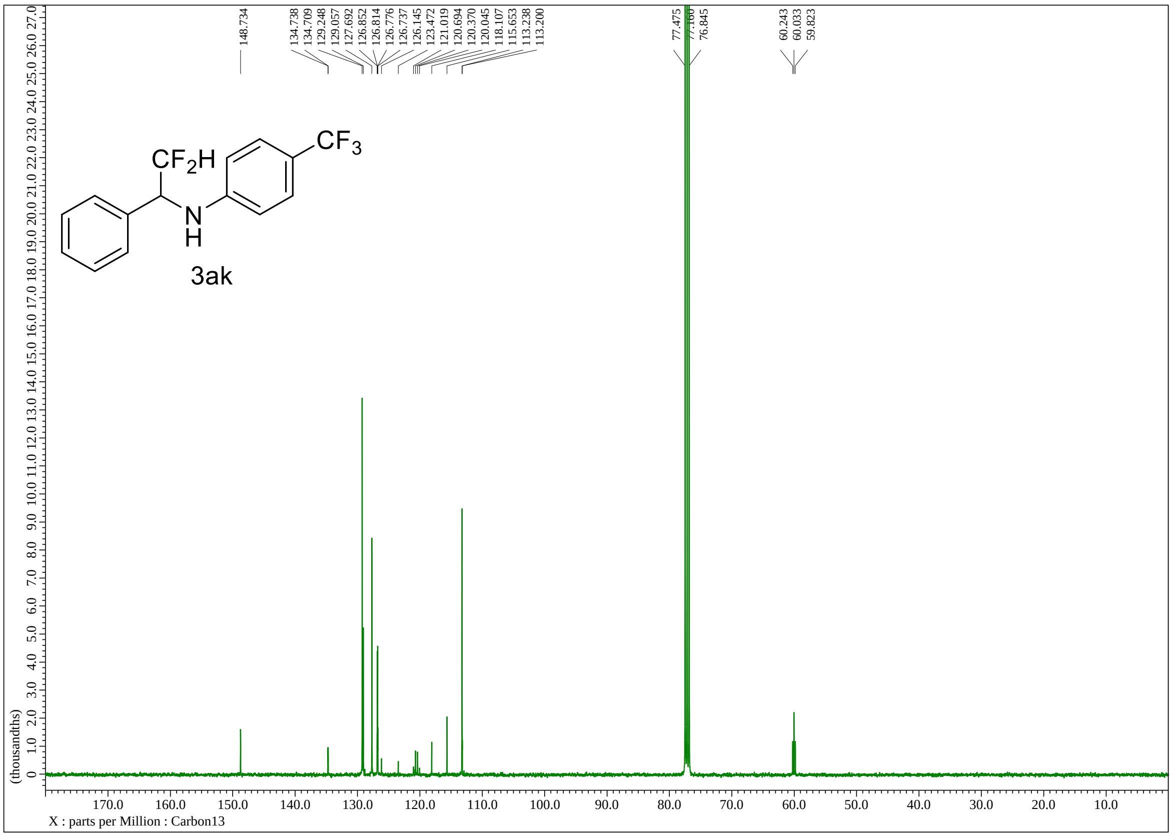

^{19}F NMR spectrum of **3ak** in $CDCl_3$. (376 MHz)

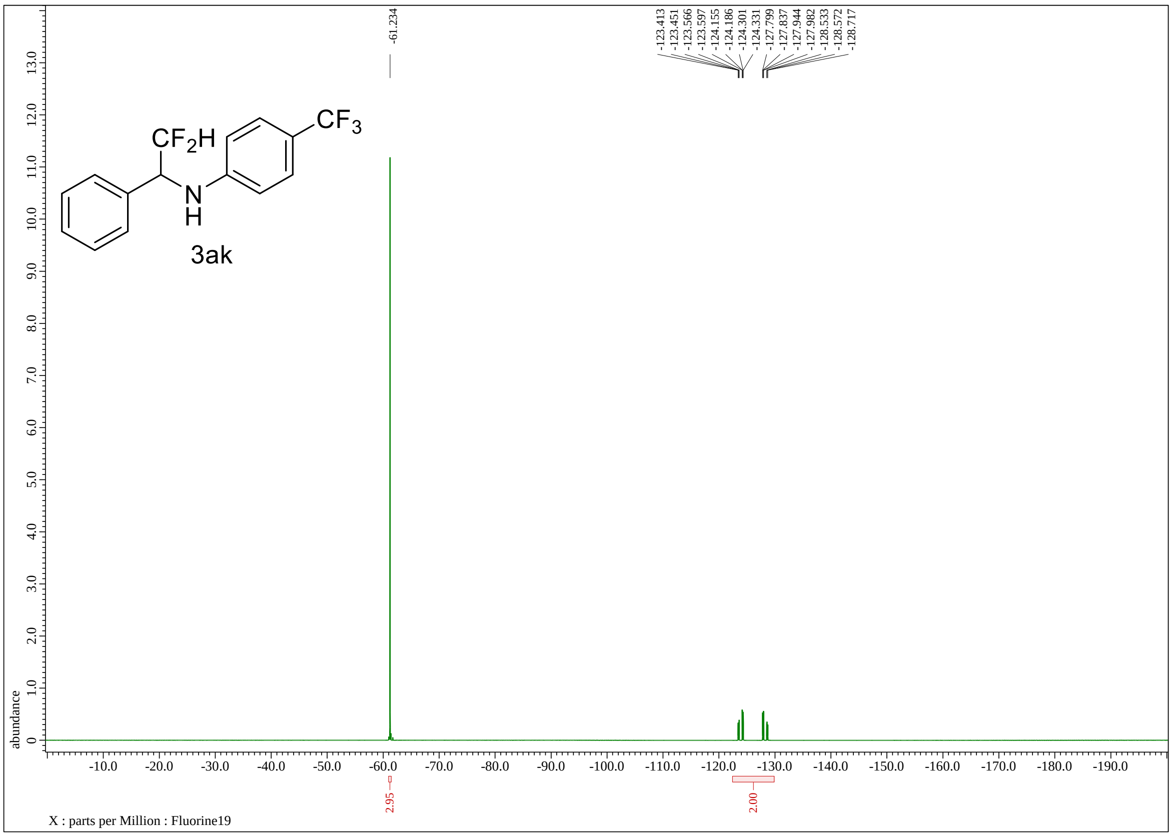

^{1}H NMR spectrum of **3al** in $CDCl_3$. (400 MHz)

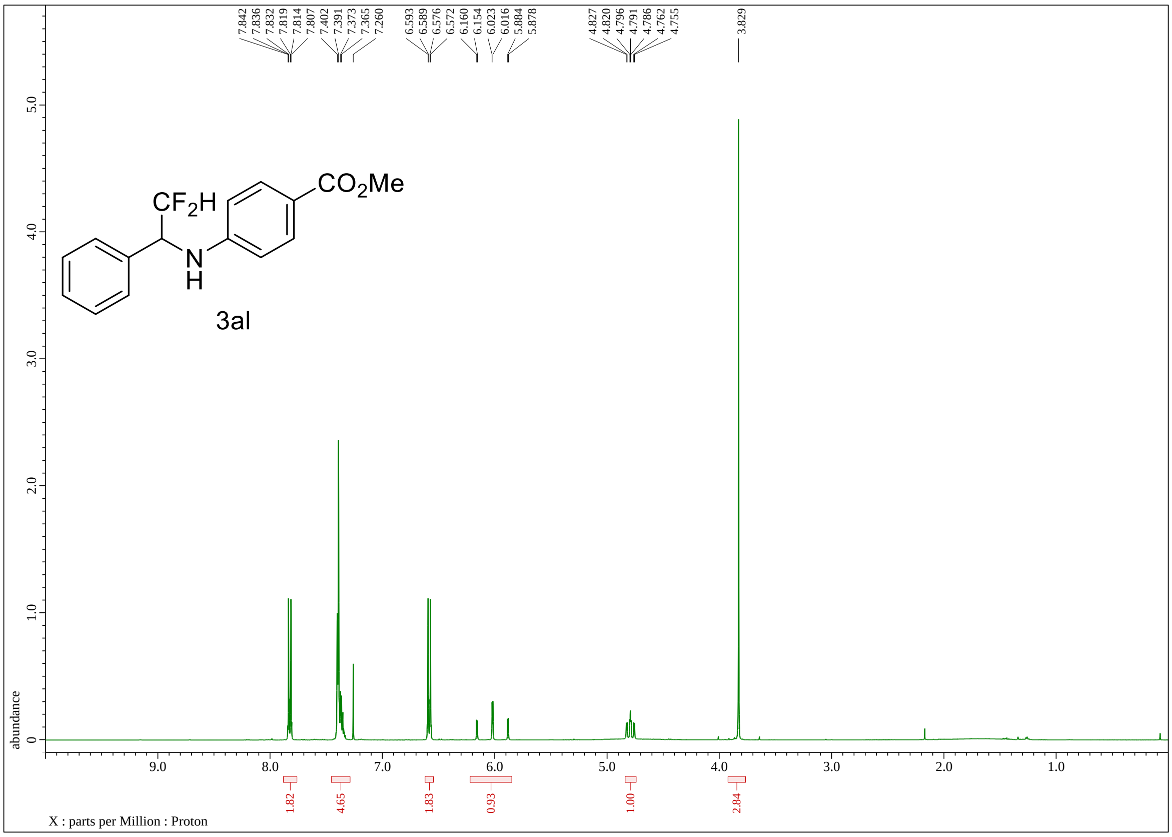

^{13}C NMR spectrum of **3al** in $CDCl_3$. (100 MHz)

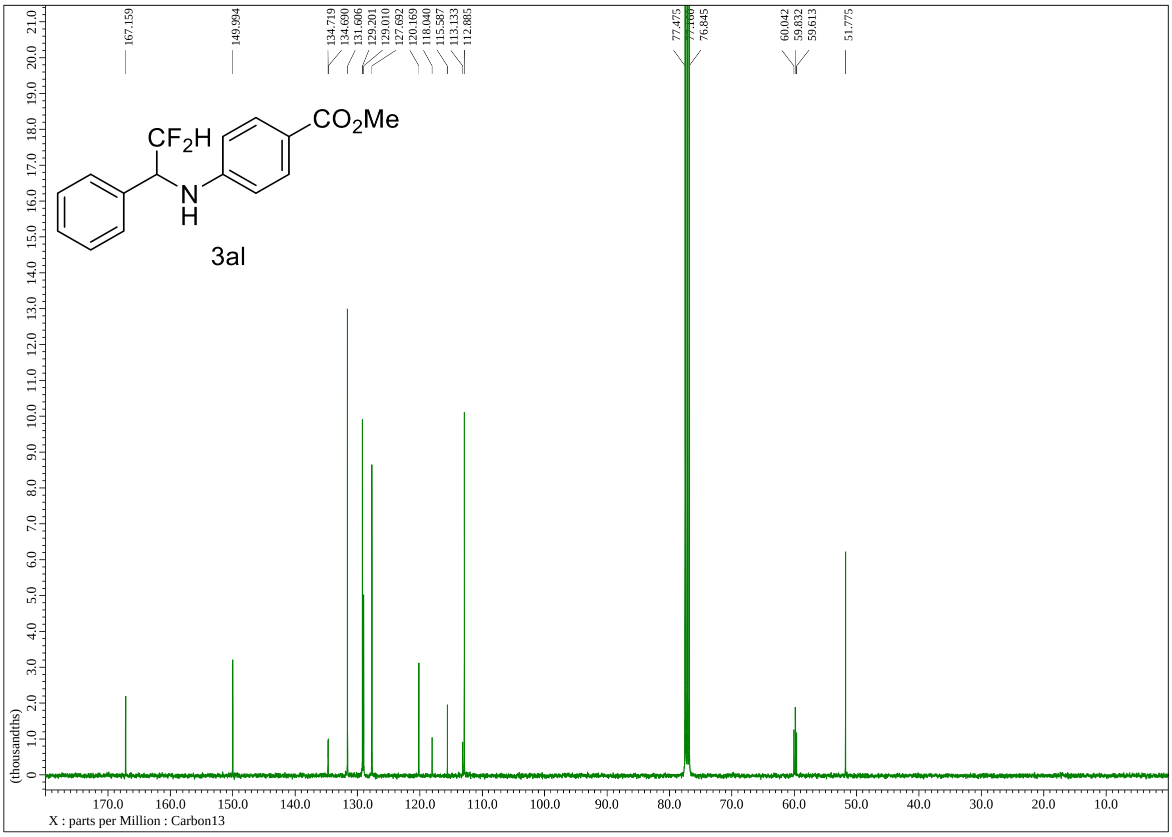

^{19}F NMR spectrum of **3al** in $CDCl_3$. (376 MHz)

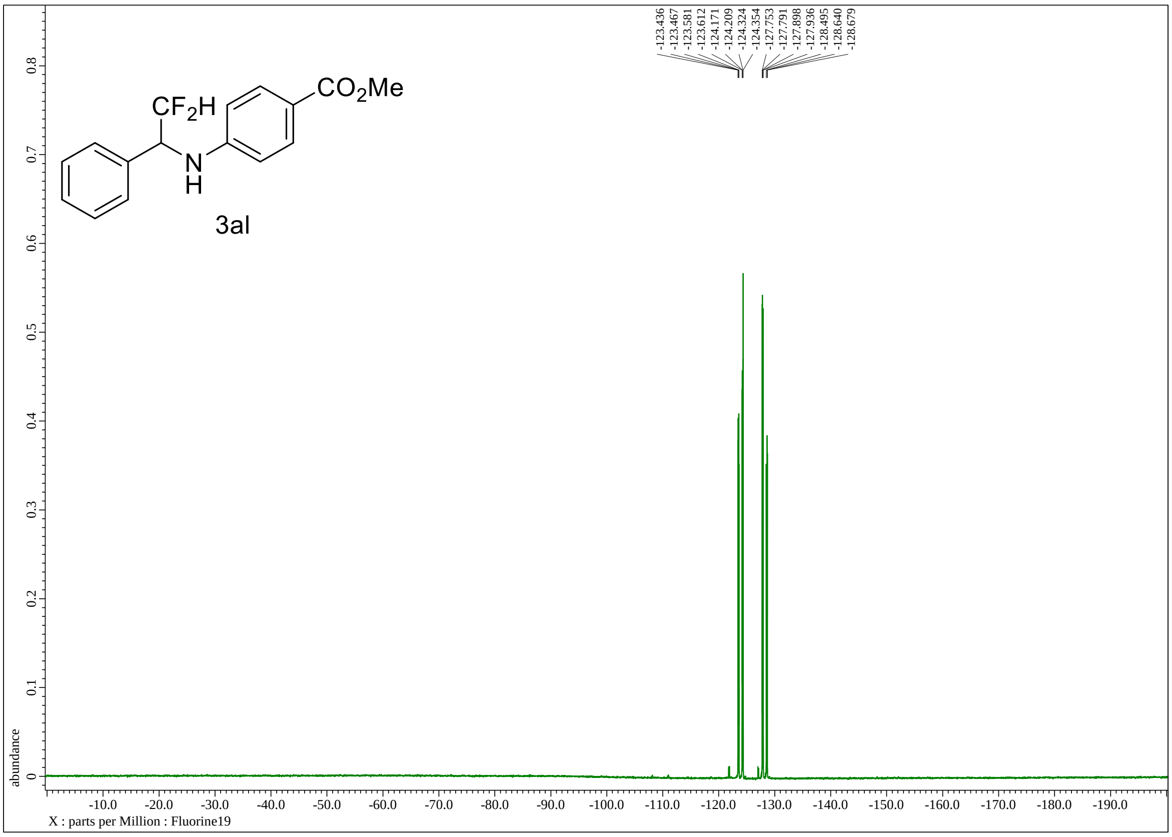

^{1}H NMR spectrum of **3am** in $CDCl_3$. (400 MHz)

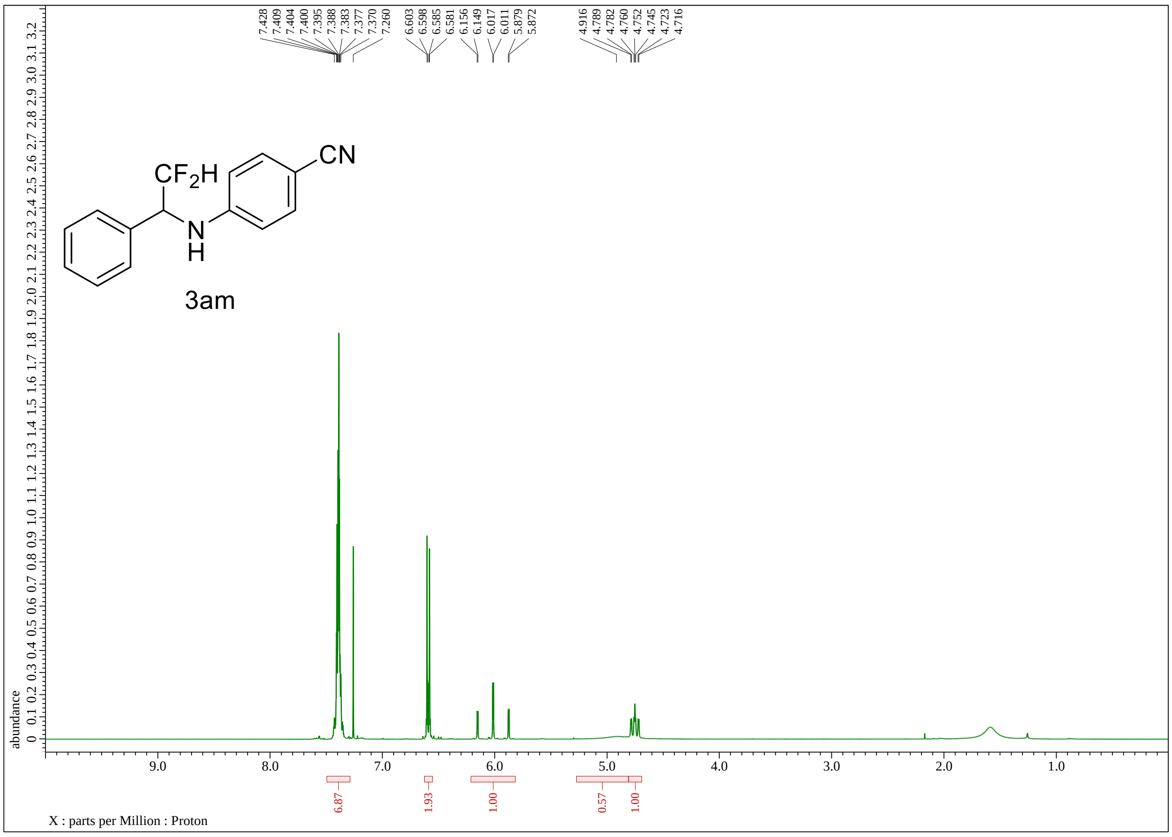

^{13}C NMR spectrum of **3am** in $CDCl_3$. (100 MHz)

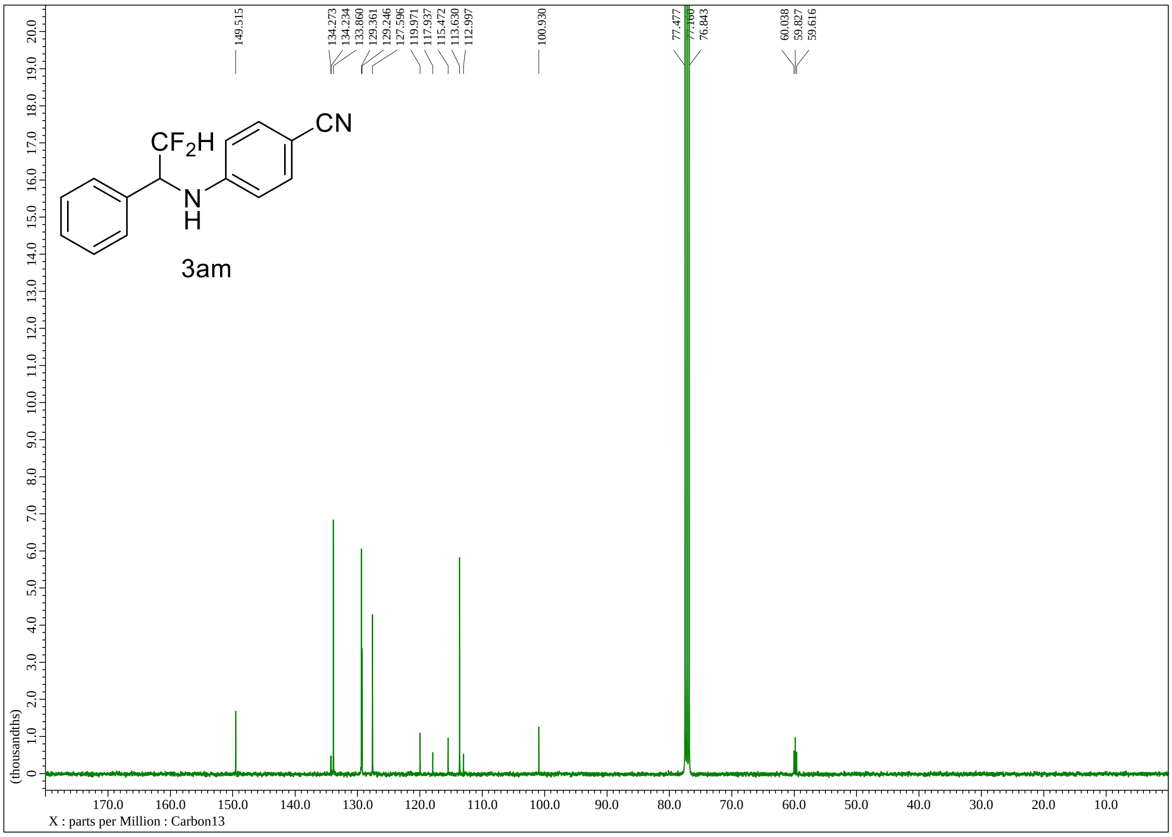

^{19}F NMR spectrum of **3am** in $CDCl_3$. (376 MHz)

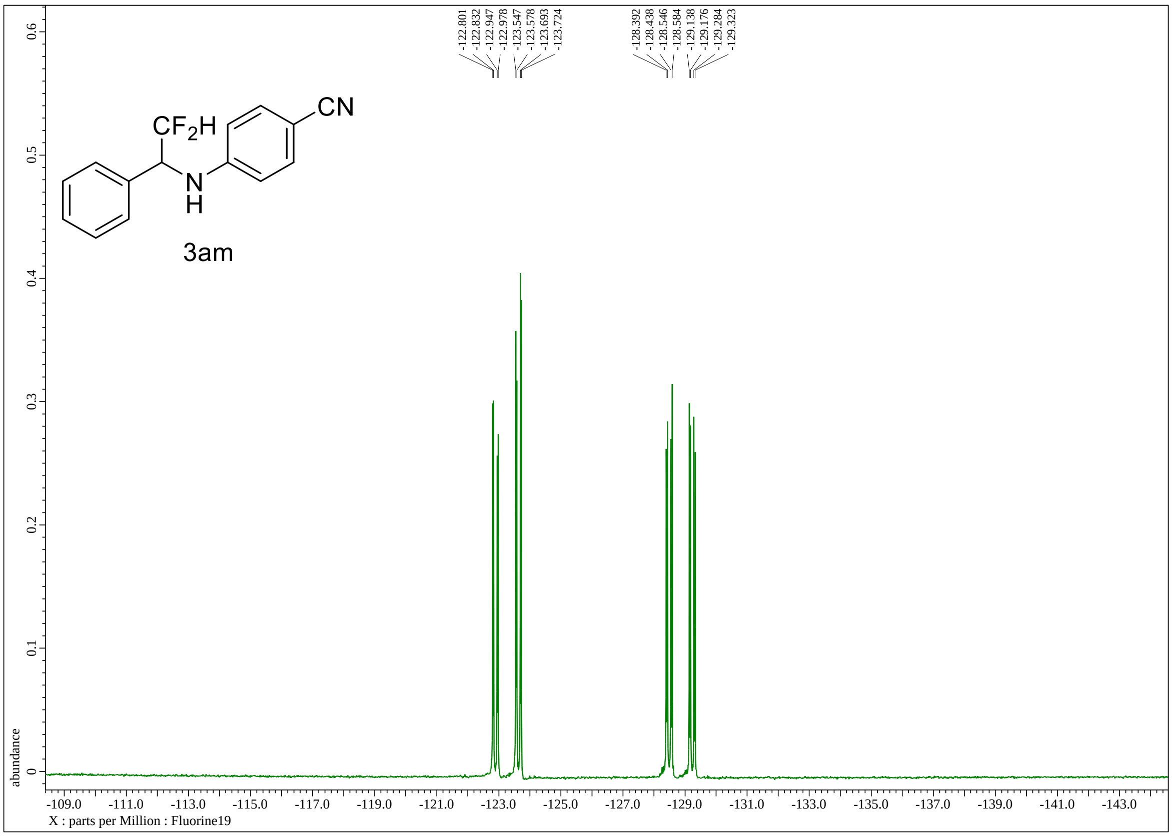

^{1}H NMR spectrum of **3an** in $CDCl_3$. (400 MHz)

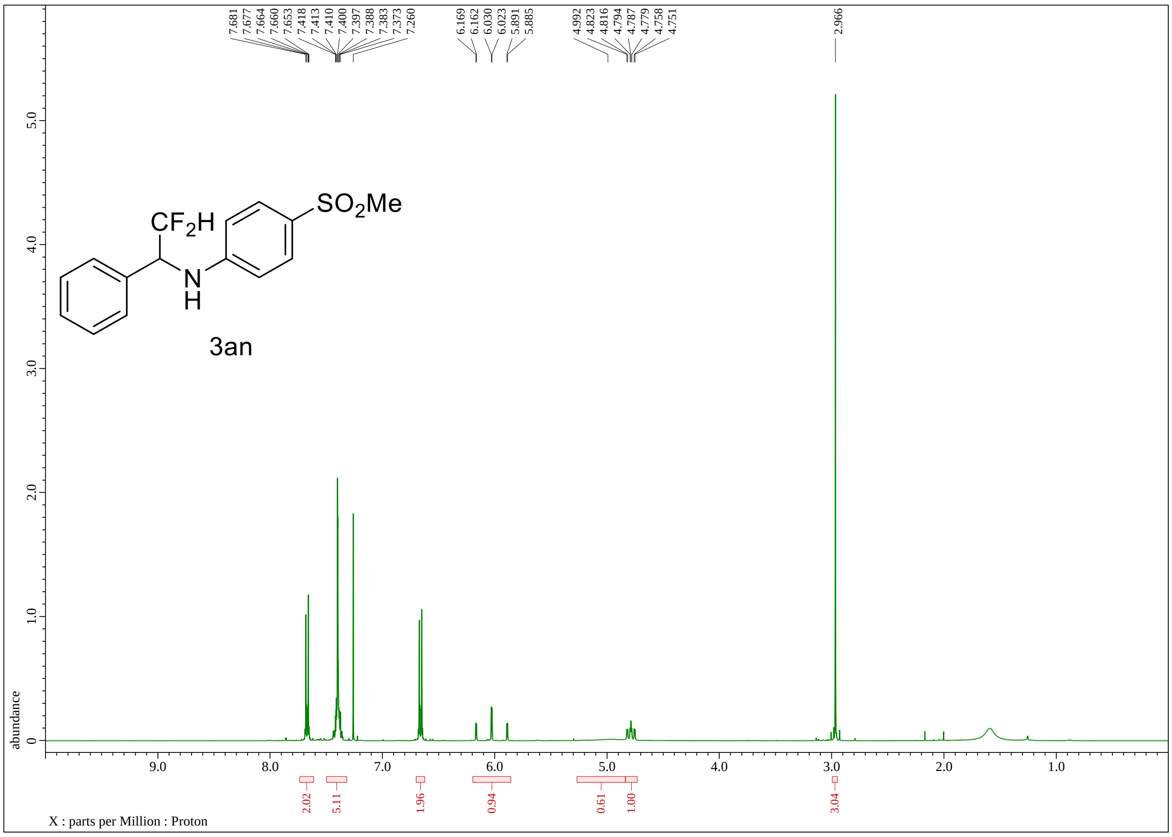

^{13}C NMR spectrum of **3an** in $CDCl_3$. (100 MHz)

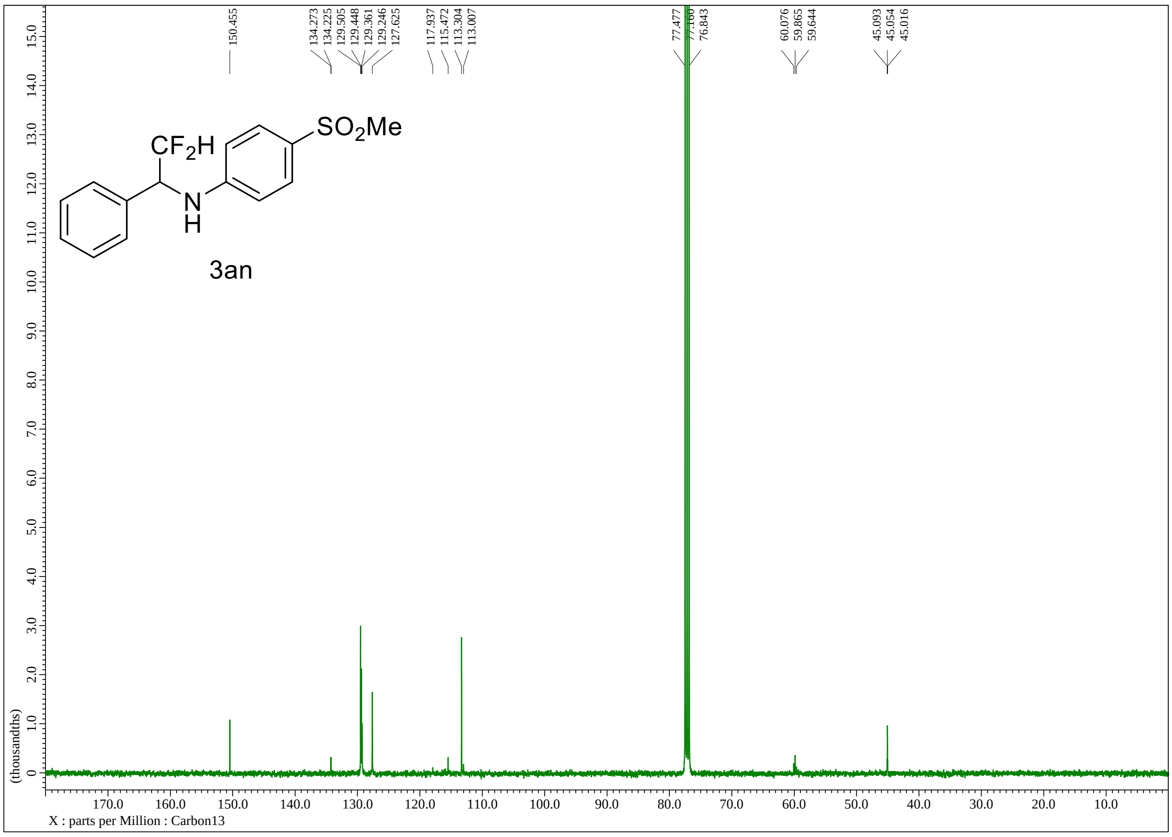

^{19}F NMR spectrum of **3an** in $CDCl_3$. (376 MHz)

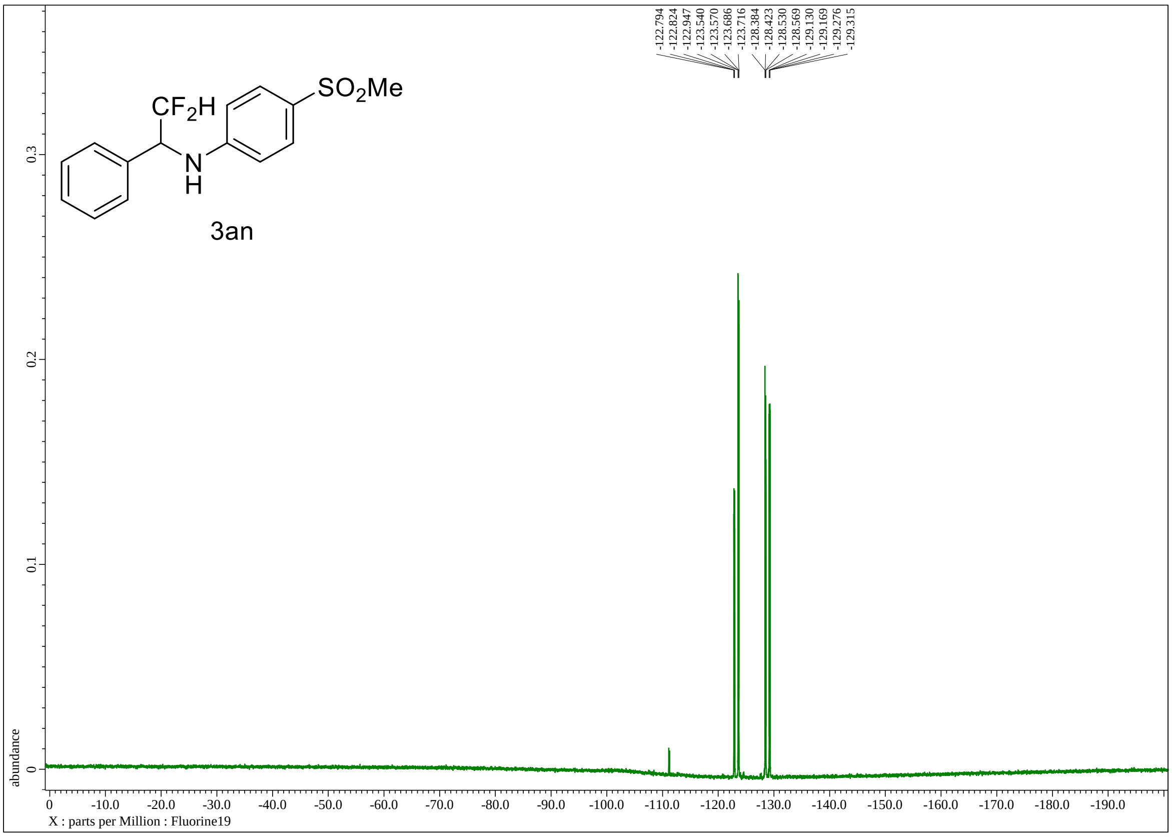

^{1}H NMR spectrum of **3ao** in $CDCl_3$. (400 MHz)

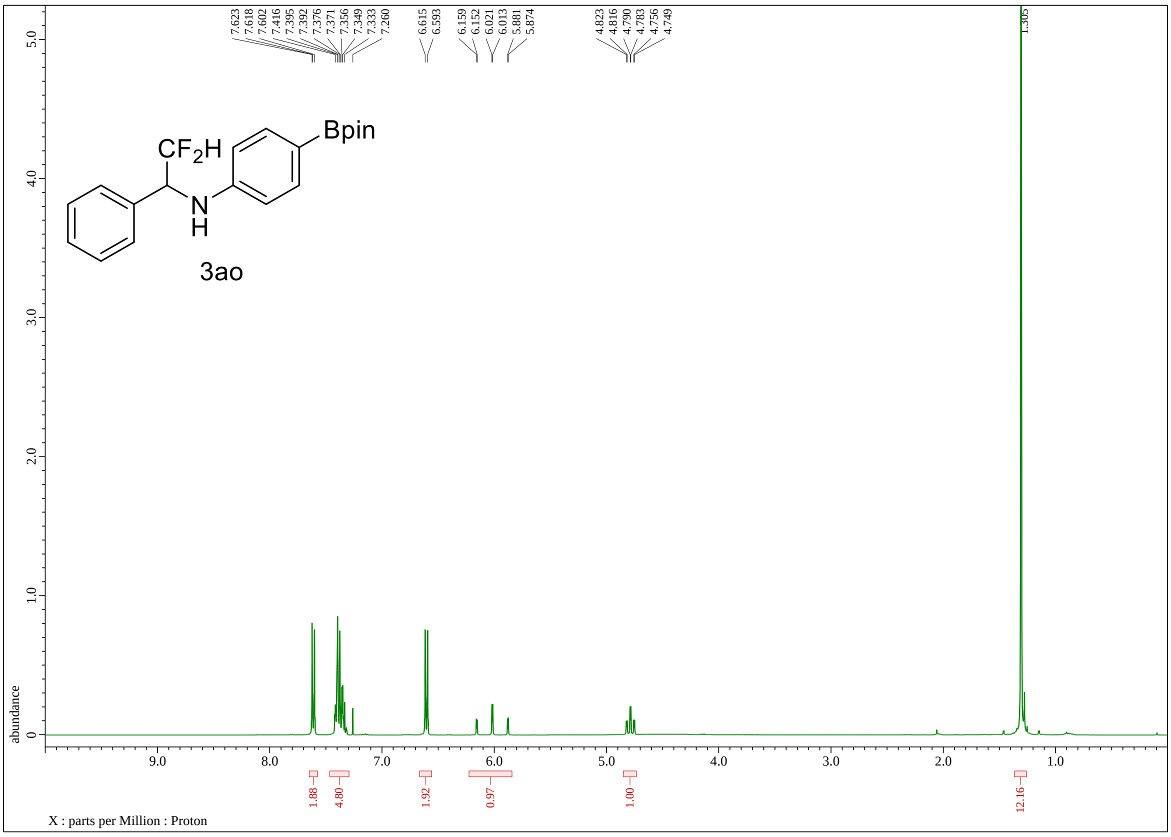

^{13}C NMR spectrum of **3ao** in $CDCl_3$. (100 MHz)

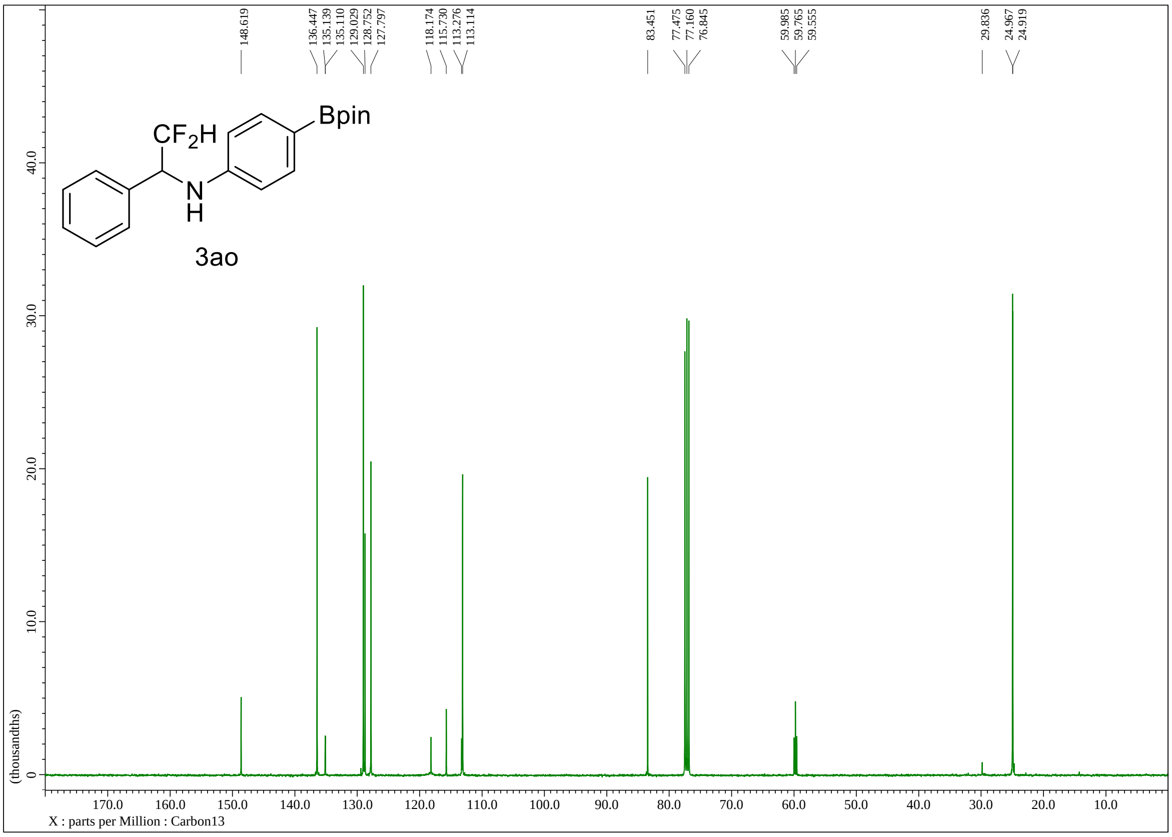

^{19}F NMR spectrum of **3ao** in $CDCl_3$. (376 MHz)

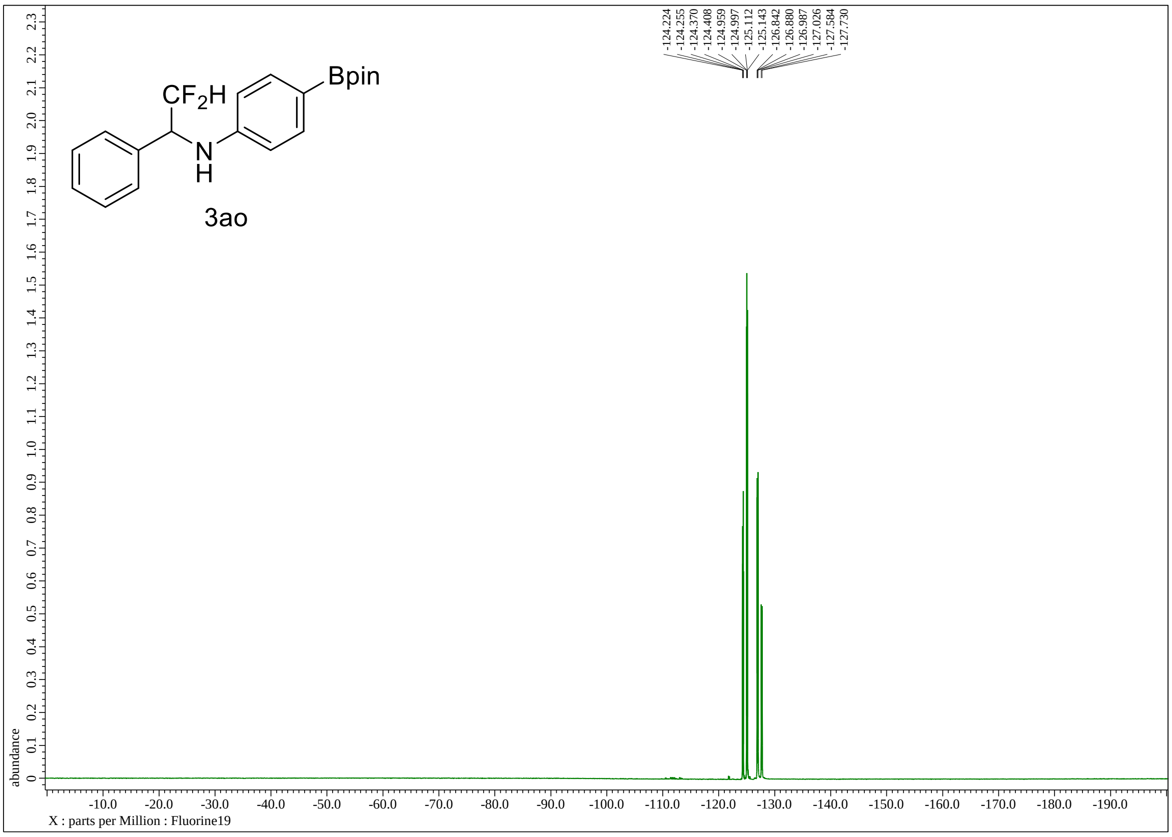

^{1}H NMR spectrum of **3ap** in DMSO-d6. (400 MHz)

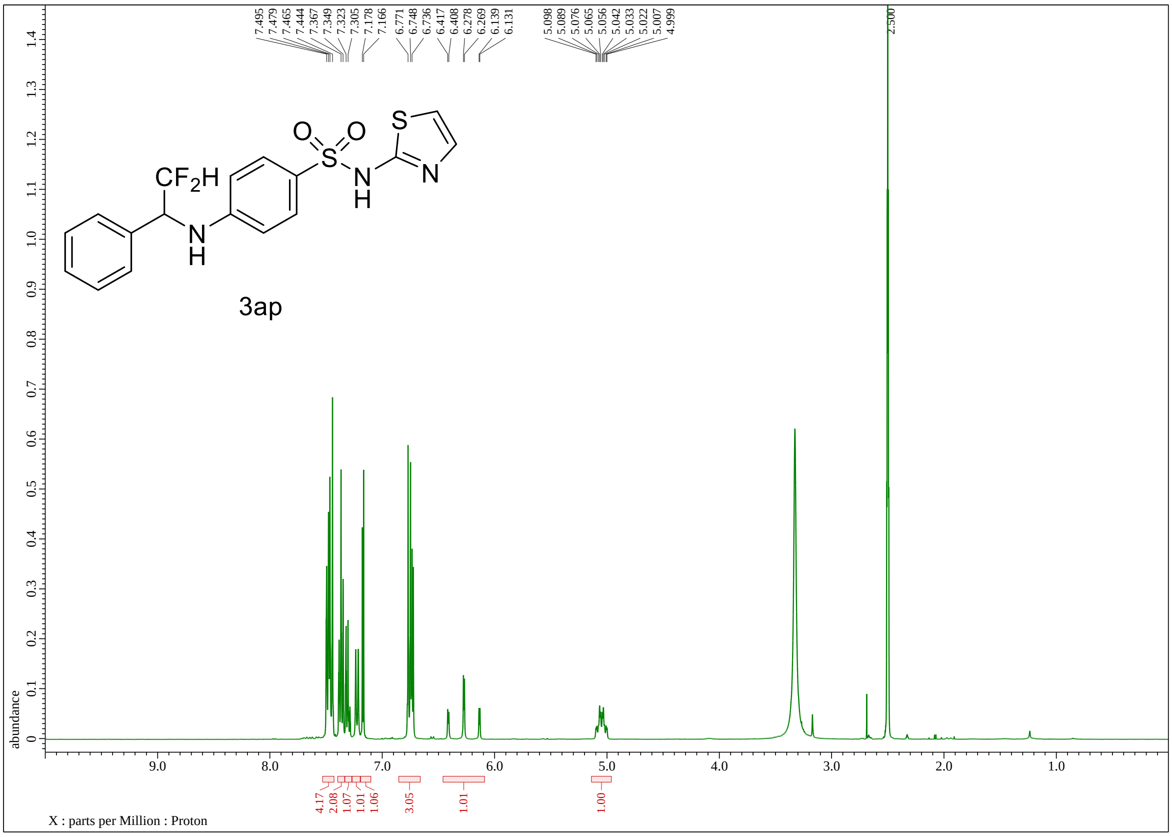

^{13}C NMR spectrum of **3ap** in DMSO-d6. (100 MHz)

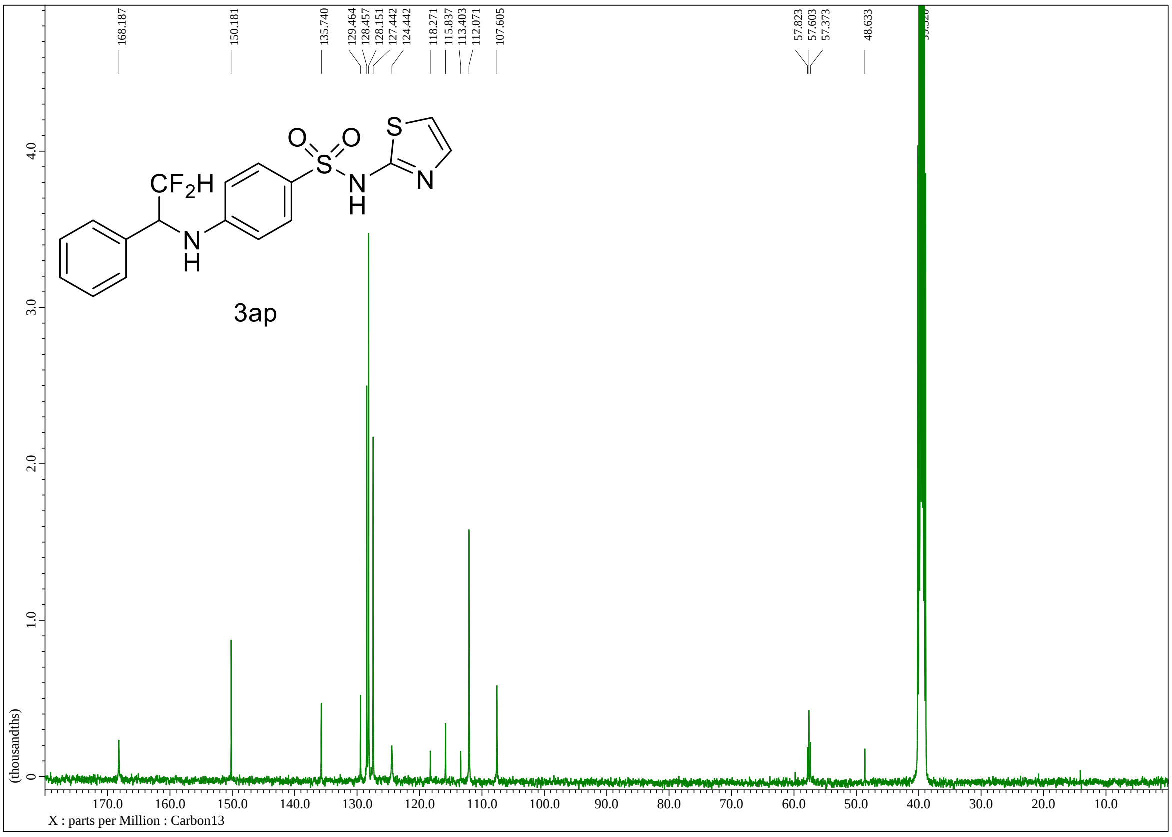

^{19}F NMR spectrum of **3ap** in $CDCl_3$. (376 MHz)

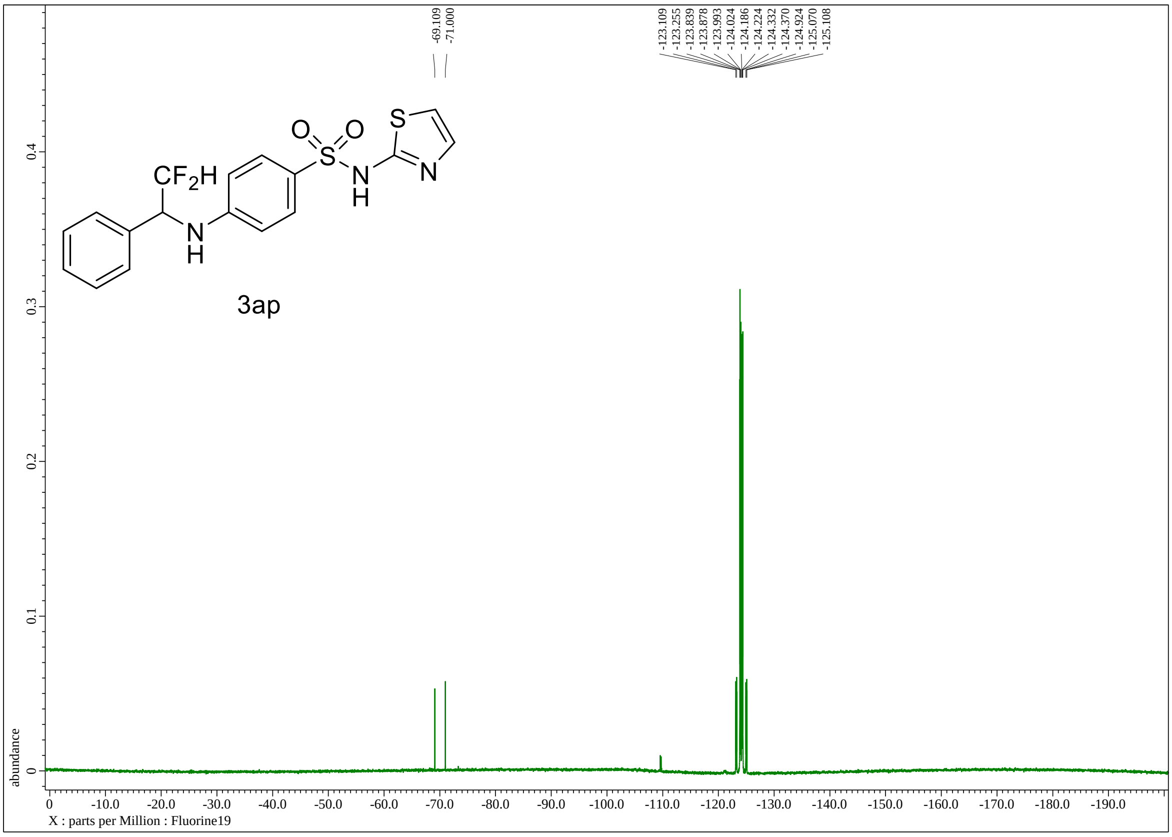

^{1}H NMR spectrum of **3aq** in $CDCl_3$. (400 MHz)

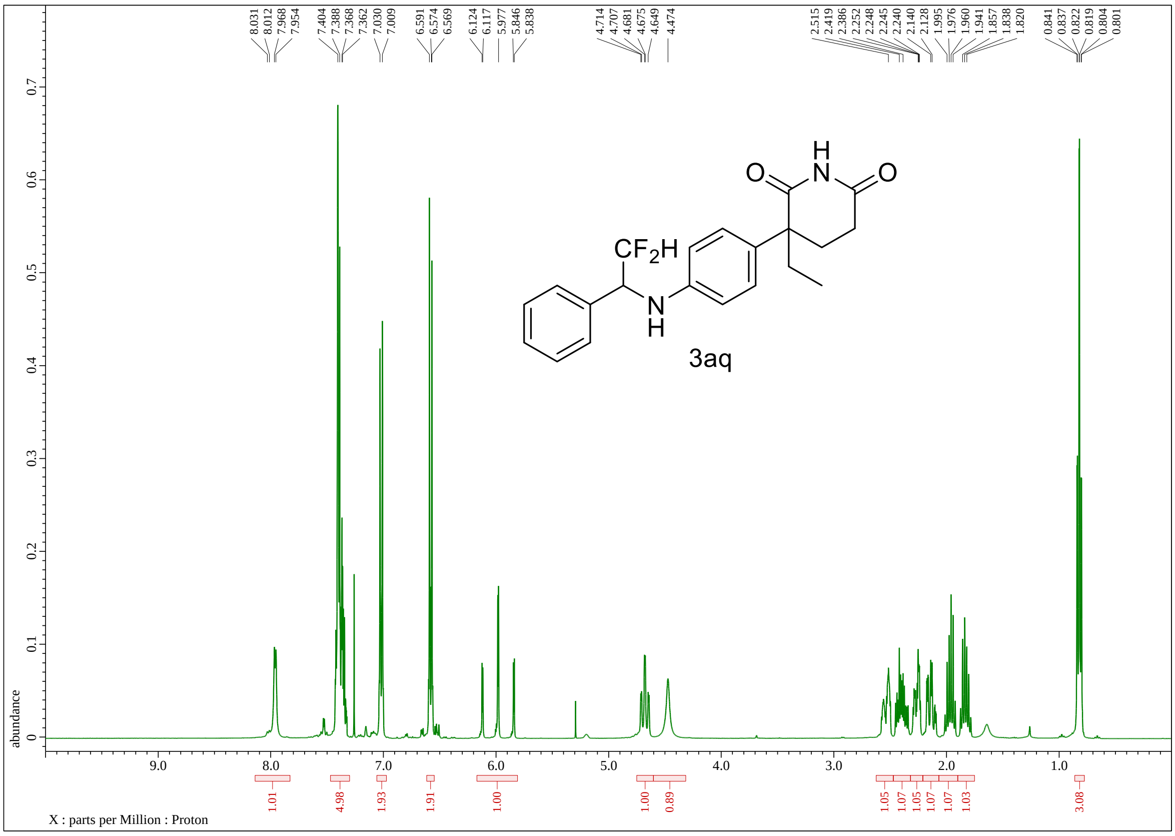

^{13}C NMR spectrum of **3aq** in $CDCl_3$. (100 MHz)

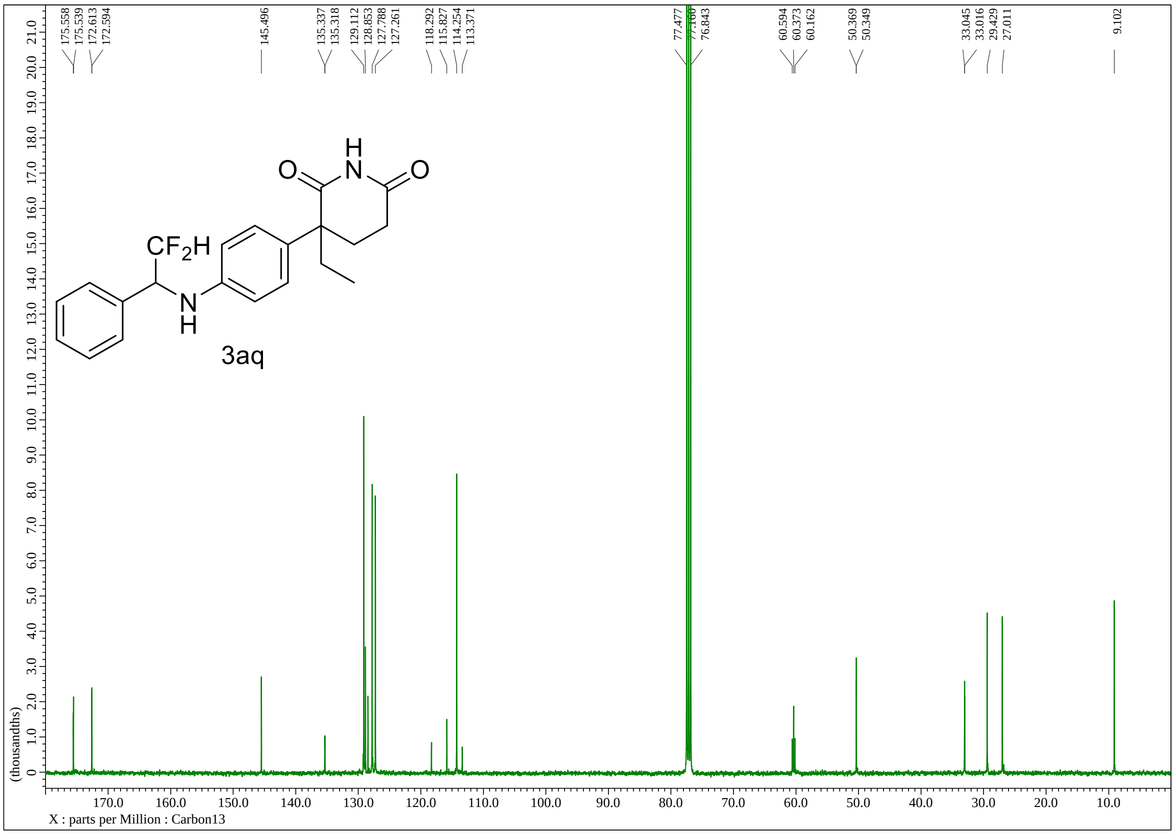

^{19}F NMR spectrum of **3aq** in $CDCl_3$. (376 MHz)

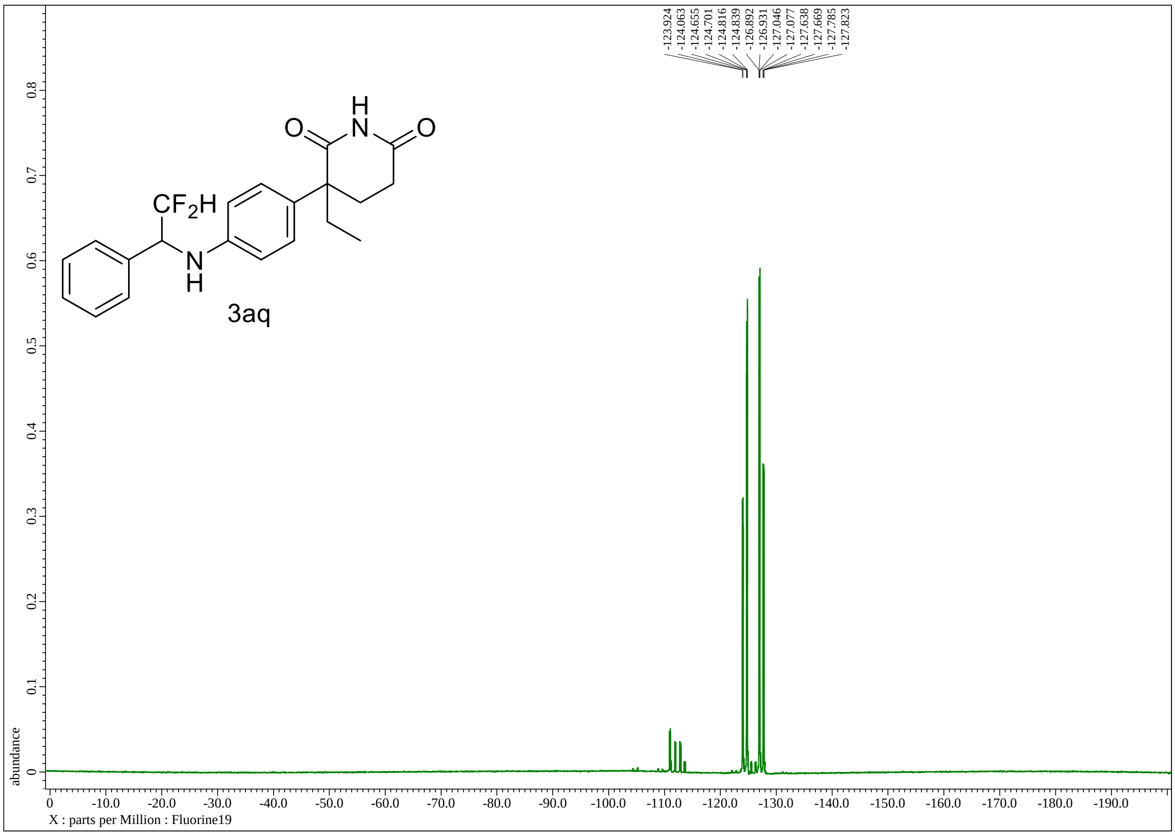

Supplement: Supplementary file 1 [file ol6c01025_si_001.pdf]
